# Yiqihuoxue formula improves atherosclerosis by regulating macrophage polarization associated with enhanced STAT6/PPARγ pathway

**DOI:** 10.3389/fcvm.2026.1896890

**Published:** 2026-09-22

**Authors:** Quan Xie, Binding Wang, Jinli Zhuang, Yi Guan, Xiaohong Yang, Jianfeng Li, Shengyu Cun, Yanting Chen, Ya Qin, Yanqing Li

**Affiliations:** 1 Department of Rehabilitation, Hospital of Chengdu University of Traditional Chinese Medicine, Chengdu, China; 2 Chengdu University of Traditional Chinese Medicine, Chengdu, China; 3 Department of TCM & Western Medicine, Affiliated Reproductive Maternity and Child Hospital of Chengdu University of Traditional Chinese Medicine, Chengdu, China

**Keywords:** atherosclerosis, high-fat diet, macrophage polarization, STAT6/PPARγ pathway, Yiqihuoxue formula

## Abstract

**Background:**

Atherosclerosis (AS) is a major contributor to deaths from cardiovascular diseases. Yiqihuoxue Formula (YQHXF) reflects the traditional Chinese medicine principle of “Qi-invigorating and blood-activating.” This study investigates the anti-atherosclerotic effects and mechanism of YQHXF through the signal transducer and activator of transcription 6 (STAT6)/ peroxisome proliferator-activated receptor gamma (PPARγ) signaling pathway and macrophage polarization.

**Methods:**

Ultra-high performance liquid chromatography-high resolution mass spectrometry (UHPLC-HRMS) was used to detect the components of YQHXF-containing serum (YQHXF-CS). Using oxidized low-density lipoprotein (ox-LDL), RAW264.7 macrophages were induced into foam cells *in vitro* and treated with YQHXF-CS. PPARγ knockdown was achieved using siRNA. Cell viability, lipid accumulation, macrophage polarization markers, and STAT6/PPARγ signaling were analyzed. *In vivo*, ApoE^−/−^ mice fed a high-fat diet (HFD) received low, medium, or high-dose YQHXF or atorvastatin. Serum lipids, aortic plaque area, histopathology, macrophage polarization, and pathway proteins were assessed.

**Results:**

The results showed that YQHXF-CS contained 2,317 components. *In vitro*, 5% YQHXF-CS enhanced cell viability, reduced lipid droplets, downregulated M1 markers (nitric oxide synthase 2 (*Nos2)*, *tumor necrosis factor (Tnf)*, *interleukin 1 beta (Il1b)*), upregulated M2 markers (*interleukin 4 (Il4)*, *arginase 1 (Arg1)*, *transforming growth factor-*β *(Tgfb)*, CD163, CD206), and this was accompanied by enhanced STAT6/PPARγ pathway. Moreover, YQHXF-CS partially counterbalanced siRNA-PPARγ-induced foam cells formation, and this effect was associated with M2 macrophages polarization. *In vivo*, YQHXF reduced body weight and serum lipid accumulation, diminished aortic plaque area and pathological damage, increased M2 macrophages (CD206^+^, IL-4, IL-10), and decreased M1 macrophages (CD86^+^, TNF-α, IL-1β). Mechanistically, YQHXF partially restored aortic p-STAT6 and PPAR*γ* expression.

**Conclusions:**

YQHXF ameliorates AS, as evidenced by reduced foam cell formation, alleviated vascular lesions, and promotion of anti-inflammatory M2 macrophage polarization; these effects were associated with enhanced STAT6/PPARγ signaling. This validates the theory of “Qi-invigorating and blood-activating” and highlights YQHXF's therapeutic potential.

## 1 Introduction

Atherosclerosis (AS) is a chronic vascular disease characterized by lipid deposition in the arterial intima, sustained inflammation, and fibrous plaque formation (1, 2). The pathogenesis involves dysregulated lipid metabolism, endothelial dysfunction, smooth muscle cell proliferation, and immune-mediated inflammatory responses. A key pathological feature is the oxidation of low-density lipoprotein (LDL) to oxidized LDL (ox-LDL) in the subendothelial space, promoting monocyte recruitment and their differentiation into macrophages. These macrophages engulf ox-LDL via scavenger receptors such as CD36, transforming into foam cells that form the earliest atherosclerotic lesions (3, 4). Progressive plaque inflammation and fibrous cap thinning may culminate in rupture, thrombosis, and severe clinical manifestations including myocardial infarction and stroke. Cardiovascular diseases continue to be the primary cause of mortality worldwide, accounting for 17.9 million deaths annually based on World Health Organization (WHO) statistics (5), with AS underpinning most cases (6). While statins, antiplatelet agents, and revascularization procedures have improved outcomes, limitations persist including treatment resistance, side effects, and restenosis (7, 8). Therefore, further investigation into the pathophysiology of AS and the development of multi-target, low-toxicity therapeutic strategies hold significant clinical relevance.

According to traditional Chinese medicine (TCM) theory, AS aligns with the diagnostic categories of “chest impediment”, “vessel impediment”, and “blood stasis syndrome”. The Yellow Emperor's Inner Canon identifies qi-blood disharmony is the core pathogenesis to AS. Contemporary TCM research establishes “qi deficiency with blood stasis” as the fundamental AS syndrome pattern, reflecting a pathological mechanism of “deficiency in origin and excess in superficiality” (9). “Qi deficiency” manifests through declining visceral functions, particularly spleen and lung qi deficiency, which impairs blood circulation and disrupts nutrient transport and qi-blood distribution, ultimately producing dyslipidemia. “Blood stasis” develops when inadequate qi propulsion causes blood stagnation and collateral obstruction, mirroring modern medical concepts of endothelial injury, lipid deposition, and inflammation. The “qi-supplementing and blood-activating” principle has emerged as a key strategy for AS prevention and treatment, restoring vascular homeostasis by enhancing circulation, resolving stasis, and rebalancing qi-blood and yin-yang (10, 11). Pharmacological evidence supports this approach. For example, Hua et al. (12) demonstrated that Yiqi Wenyang decoction attenuates vascular inflammation and AS progression by inhibiting the NF-*κ*B pathway. Bushen Huoxue Yiqi Formula attenuates myocardial fibrosis in ischemic heart failure by inhibiting endothelial-mesenchymal transition through modulation of the SIRT1/Notch1 pathway (13). Yiqihuoxue Formula (YQHXF) is a classical TCM prescription designed to tonify Qi and activate blood circulation. The YQHXF consists of *Ginseng Radix ET Rhizoma Rubra* (Hong-Shen, 6 g), *Atractylodis Rhizoma* (Bai-Zhu, 3 g), *Salviae Miltiorrhizae Radix ET Rhizoma* (Dan-Shen, 3 g), *Chuanxiong Rhizoma* (Chuan-Xiong, 3 g), and *Notoginseng Radix ET Rhizoma* (San-Qi, 3 g), which is taken three times a day in clinical practice. Previous pharmacological studies have demonstrated that YQHXF possesses multiple cardiovascular protective effects. Clinical trials and animal experiments have shown that YQHXF improves ischemic stroke, intracerebral hemorrhage, heart failure, and coronary atherosclerotic heart disease (14–18). Mechanistically, YQHXF has been reported to inhibit platelet aggregation, reduce oxidative stress, and suppress pro-inflammatory mediators. Furthermore, some clear components—such as ginsenosides from Hong-Shen, has been independently shown to regulate macrophage function and exhibit anti-atherosclerotic properties (19). Although YQHXF shows clinical promise for atherosclerosis treatment, its active constituents and mechanistic basis remain unclear.

Macrophages serve as the principal immune cells mediating the pathophysiology of atherosclerosis (20). These cells critically regulate inflammatory responses, intercellular signaling, and plaque development in atherosclerotic lesions. When exposed to pro-atherogenic stimuli, M1-polarized macrophages exacerbate inflammation through elevated secretion of interleukin-6 (IL-6), IL-1β, tumor necrosis factor-α (TNF-α), IL-12, and IL-23, thereby accelerating foam cell formation within plaques (21, 22). Conversely, IL-4 and IL-13-stimulated M2 macrophages produce anti-inflammatory mediators including IL-10, transforming growth factor-β (TGF-β), and arginase-1 (Arg1), which mitigate M1-driven inflammation while facilitating tissue repair, phagocytic clearance, angiogenesis, and fibrotic responses (23). Studies have demonstrated that early-stage atherosclerosis is predominantly characterized by M1 macrophage infiltration, whereas an increased proportion of M2 macrophages in advanced plaques is associated with plaque stabilization (24). Therefore, modulating the balance of macrophage polarization (M1/M2 ratio) is considered a key strategy for inhibiting the progression of atherosclerosis (25).

The STAT signaling pathway critically regulates macrophage polarization. The STAT family regulates macrophage polarization through the release of a broad spectrum of inflammatory mediators (26). Among these, signal transducer and activator of transcription 6 (STAT6) serves as a principal transcription factor in the IL-4/IL-13 receptor pathway. The peroxisome proliferator-activated receptor (PPAR) family comprises ligand-dependent nuclear transcription factors, among which PPAR-γ predominates alongside PPAR-α and PPAR-β (27). Macrophages exhibit high PPAR-γ expression, where its activation promotes M2 polarization while inhibiting M1 differentiation, effectively reversing the M1-to-M2 transition and mediating anti-inflammatory effects (28–30). Recent evidence establishes the IL-4R–STAT3/STAT6–PPAR-γ axis as a key regulator of M2 macrophage polarization during inflammatory responses (31–33). Despite these advances, no studies have examined whether YQHXF influences PPAR-dependent macrophage reprogramming to mitigate atherosclerosis. In particular, the involvement of the STAT6/PPARγ signaling axis, a well-established pathway driving M2 macrophage polarization, has never been investigated in the context of YQHXF-mediated atheroprotection.

This investigation aims to uncover the effects and mechanism by which YQHXF improves atherosclerosis through the regulation of macrophage polarization associated with the STAT6/PPARγ signaling pathway, using both *in vivo* and *in vitro* methods. The findings will provide modern scientific evidence for TCM in treating AS and offer theoretical support for developing novel multi-target therapeutics.

## 2 Materials and methods

### 2.1 Screening and analysis the core targets of atherosclerosis

To predict disease-related targets, the GeneCard (https://www.genecards.org/) and CTD (https://ctdbase.org/) databases were utilized. The search was conducted using the keyword “atherosclerosis.” Following the elimination of duplicate targets, the remaining targets were standardized using the Uniprot database. The STRING database (https://cn.string-db.org/) was used to import these standardized targets and retrieve protein-protein interaction data, which was then processed using Cytoscape software. The key atherosclerosis-related targets were analyzed using the DAVID database (https://davidbioinformatics.nih.gov/) for KEGG pathway enrichment. We visualized the top 20 enriched KEGG pathways with an online bioinformatics tool (http://www.bioinformatics.com.cn/).

### 2.2 Preparation of containing serum

Male Sprague-Dawley (SD) rats (8 weeks old, 200–220 g, purchased from Chengdu Dossy Experimental Animals Co., Ltd., License number: SCXK(Chuan)2020-030) were randomly allocated to two groups (*n* = 6): the blank serum group and the YQHXF-containing serum group. Rats were housed under standard conditions (temperature 22 ± 2 °C, humidity 50%–60%, 12 h light/dark cycle) with free access to standard chow and water for 7 days acclimatization. Following acclimatization, YQHXF group rats received daily gavage administration of YQHXF at 4.86 g/kg (clinically equivalent dose) for seven days, while blank serum group rats consumed standard diet and water for the same duration. The YQHXF solution was prepared by dissolving 4.86 g of empirical formula granules in 10 mL of normal saline with thorough mixing. Abdominal aorta blood collection occurred one hour post-final administration under anesthesia. Blood samples stood at room temperature for 2 h before centrifugation at 1,500 g for 10 min to isolate supernatant. To minimize individual variability, serum from six rats per group was pooled under aseptic conditions to form a homogeneous batch, which was then aliquoted and stored at −80 °C. Serum from each group was pooled, heat-inactivated at 56 °C for 30 min in a water bath, then sterilized through 0.22 μm membrane filtration to yield YQHXF-containing serum (YQHXF-CS) and blank serum (BS), respectively.

### 2.3 YQHXF-CS compounds analysis

Ultra-high performance liquid chromatography-high resolution mass spectrometry (UHPLC-HRMS) characterized the chemical constituents of YQHXF-CS. A Vanquish UHPLC system (Thermo Fisher Scientific, USA) interfaced with an Orbitrap Exploris 120 mass spectrometer (Thermo Fisher Scientific, USA) achieved chromatographic separation on a Phenomenex Kinetex C18 column (2.1 mm × 100 mm, 2.6 μm). The mobile phase consisted of (A) aqueous phase containing 0.01% acetic acid and (B) isopropanol: acetonitrile (1:1, v/v). The sample tray temperature was maintained at 4 °C with an injection volume of 2 μL. The Orbitrap Exploris 120 mass spectrometer operated with Xcalibur software (version 4.4, Thermo) acquired both full-scan and MS/MS data. Data-dependent acquisition parameters included a sheath gas flow rate of 50 Arb, auxiliary gas flow rate of 15 Arb, capillary temperature of 320 °C, full MS resolution of 60,000, and MS/MS resolution of 15,000, with stepped normalized collision energy (SNCE) set at 20/30/40 and spray voltages of 3.8 kV (positive) or −3.4 kV (negative). For sample preparation, we thawed aliquots from −80 °C storage on ice, vortexed them for 10 s, then transferred 50 μL to centrifuge tubes containing 300 μL of 20% acetonitrile-methanol with internal standard. The IS working solution (250 μg/mL) was prepared by diluting a 1,000 μg/mL stock solution in 20% acetonitrile-methanol. After vortexing for 3 min, samples were centrifuged at 13,800 g (4 °C, 15 min), and the supernatant underwent storage at −20 °C for 20 min. Following a second centrifugation under identical conditions, we filtered the supernatant through a 0.22 μm membrane into injection vials for UHPLC-HRMS analysis.

The metabolite features of components in YQHXF-CS were referenced using the Metabolomics Standards Initiative (MSI). Level 1: Confirmed by comparison with laboratory-built standard libraries under identical analytical conditions (retention time, precursor ion, fragment ions). Level 2: Putatively annotated compounds through MS/MS spectral matching with public databases (e.g., GNPS, HMDB, MassBank). Level 3: Characterized as compound classes or assigned molecular formulae based solely on accurate mass. The metabolite features tested were the results obtained after subtracting the corresponding chromatographic peaks in the “blank serum control”. This procedure eliminates potential interference from host endogenous metabolites present in the matrix background to the greatest extent.

### 2.4 Establishment of ox-LDL-induced macrophage foam cell

To establish a foam cell model, RAW264.7 cells were exposed to ox-LDL. The murine monocyte-macrophage leukemia cell line (RAW 264.7, Cat. No. iCell-m047) was obtained from iCell Bioscience Inc (Shanghai, China). Cells were maintained in iCell-m047-001b medium supplemented with 10% fetal bovine serum, 100 U/mL penicillin, and 100 μg/mL streptomycin at 37 °C with 5% CO₂. Upon reaching 70% confluence, cultures were incubated with 100 μg/mL ox-LDL (Cat. No. YB-002, Yiyuan biotechnology, Guangzhou, China) for 24 h. Lipid droplet formation was assessed by Oil Red O staining. The presence of prominent red-stained lipid droplets surrounding the cells indicated successful induction of RAW264.7 cell foam cell formation.

### 2.5 Cells grouping

Grouping 1: RAW 264.7 cells were divided into the blank control (BC) + YQHXF-CS (0%, 1%, 2%, 3%, 4%, and 5%), as well as ox-LDL + YQHXF-CS (0%, 1%, 2%, 3%, 4%, and 5%) groups.

Grouping 2: RAW 264.7 cells were divided into the BC, ox-LDL + blank serum (BS), ox-LDL + low-dose (1.25%) YQHXF-CS (YQHXF-CS-L), ox-LDL + middle-dose (2.5%) YQHXF-CS (YQHXF-CS-M), and ox-LDL + high-dose (5%) YQHXF-CS (YQHXF-CS-H) groups.

Grouping 3: RAW264.7 cells were first transfected with either siRNA-NC or siRNA-PPARγ using a commercial transfection reagent (No. 84753, Gimar Gene), followed by 24-hour incubation with 100 μg/mL ox-LDL. The cells were then allocated to six experimental groups: siRNA-NC, siRNA-NC + ox-LDL + BS, siRNA-NC + ox-LDL + YQHXF-CS (5%), siRNA-PPARγ, siRNA-PPARγ + ox-LDL + BS, and siRNA-PPARγ + ox-LDL + YQHXF-CS groups.

### 2.6 SiRNA transfection

RAW264.7 cells in the logarithmic growth phase were harvested and resuspended in culture medium to generate a single-cell suspension. The cell density was adjusted to 2 × 10^5^ cells/mL, and 2 mL of this suspension was seeded into each well of a 6-well plate. The cells were cultured at 37 °C in a 5% CO_2_ incubator. Cells were transfected with small interfering RNA (siRNA) targeting mouse *Pparg* or a negative control siRNA (GenePharma, Shanghai, China) using a commercial transfection reagent (No. 84753, GenePharma, Shanghai, China) according to the manufacturer's protocol. To prepare the transfection complexes, 75  pmol of siRNA was diluted in 42.5  μL of Buffer and mixed gently. Then, 7.5  μL of Plus transfection reagent was added to form the siRNA/Plus complex after gentle pipetting. This transfection complex was combined with an appropriate volume of complete antibiotic-free medium and mixed thoroughly. The resulting mixture was added directly to the culture wells. The plates were then incubated at 37 °C in 5% CO_2_. Transfection efficiency was confirmed by both real-time fluorescence quantitative polymerase chain reaction (RT-qPCR) (RT-qPCR) and Western blotting.

SiRNA sequence:

Negative control, sense-5′-UUCUCCGAACGUGUCACGUTT-3, antisence-5′-ACGUGACACGUUCGGAGAATT-3′;
*Pparg*-Mus-357, sense-5′-GCUCCAAGAAUACCAAAGUTT-3, antisence-5′-ACUUUGGUAUUCUUGGAGCTT-3′;
*Pparg*-Mus-1288, sense-5′-GACAGUGACUUGGCUAUAUTT-3, antisence-5′-AUAUAGCCAAGUCACUGUCTT-3′;
*Pparg*-Mus-560, sense-5′-CCAUCCGAUUGAAGCUUAUTT-3, antisence-5′-AUAAGCUUCAAUCGGAUGGTT-3′;
*Pparg*-Mus-850, sense-5′-GCGAUCUUGACAGGAAAGATT-3, antisence-5′-UCUUUCCUGUCAAGAUCGCTT-3′.

### 2.7 CCK-8 assay

RAW264.7 cells in the logarithmic growth phase were seeded into 96-well plates at 4 × 10^4^ cells per well (100 µL per well) and maintained at 37 °C with 5% CO_2_. After incubation, each well received 110 µL of CCK8 solution, prepared by diluting the reagent 1:10 in serum-free medium. After 2 h incubation under identical conditions, absorbance measurements at 450 nm were recorded using a BioTek® ELx800 microplate reader (USA).

### 2.8 Oil-red O staining

In the logarithmic growth phase, RAW264.7 cells were seeded into 96-well plates at a density of 1 × 10^5^ cells per well and maintained at 37 °C with 5% CO_2_. For vascular tissues, the entire aorta was opened longitudinally and stained en face. The The Oil Red O staining kit (No. G1262) was sourced from Beijing Solarbio Science & Technology Co., Ltd (China). After transfection and treatment, we fixed the cells with Oil Red O fixative using for 20 min and washed them twice with distilled water. The samples were briefly rinsed with 60% isopropanol for 30 s to ensure complete removal of residual stain. We repeated the isopropanol washes until the intercellular spaces became clear. Finally, the cells were stained with Oil Red O solution for 15 min and examined under a microscope (DMI1, LEICA, Germany). Quantitative analysis of Oil Red O staining was performed using ImageJ software. Oil Red O-positive area was quantified as the percentage of the total aortic luminal surface area. RGB images were first converted to 8-bit grayscale, and the threshold range was adjusted to distinguish positive staining (lipid droplets) from the background. The integrated density was then calculated. All image acquisition and quantitative analysis were conducted in a blinded manner.

### 2.9 Flow cytometry

Flow cytometry was employed to assess the surface expression levels of M1-associated markers (CD80^+^ and CD86^+^) and M2-associated markers (CD163^+^ and CD206^+^) on RAW264.7 cells. F4/80-positive macrophages were sorted using flow cytometry, and the relative percentages of M1 (CD86-positive) and M2 (CD206-positive) macrophages within the mouse aorta were determined. For cell staining, single-cell suspensions were prepared in 100 μL of PBS and incubated with the following antibodies: APC-conjugated anti-mouse CD80 (104713, BioLegend), APC-conjugated anti-mouse CD86 (105113, BioLegend), FITC-conjugated anti-mouse CD206 (141703, BioLegend), FITC-conjugated anti-mouse CD163 (TNKUPJ), PE-conjugated anti-mouse CD86 (159203, BioLegend), PE-conjugated anti-mouse CD80 (104707, BioLegend), PE-conjugated anti-mouse F4/80 (123109, BioLegend), and APC-conjugated anti-mouse CD206 (141707, BioLegend). Following antibody staining, cells were stored in the dark at 4 °C for 30 min, then centrifuged at 300 g for 5 min and rinsed with PBS. After a second centrifugation step at 300 g for 5 min, the cells were incubated at room temperature for 20 min, followed by centrifugation at 350 g for 5 min. The cells were then washed twice with 250 μL of Intracellular Staining Perm Wash Buffer, with a 350 g centrifugation step for 5 min between each wash, before being resuspended in 100 μL of the same buffer. Subsequently, the labeled cells were incubated for 30 min at 4 °C, centrifuged again at 350 g for 5 min, and finally washed with 250 μL of PBS. After a final centrifugation step at 350 g for 5 min, the cells were resuspended in 300 μL of PBS for flow cytometric (Cytoflex, Beckman, USA) analysis. The raw data of flow cytometry were shown in (Supplementary File 1).

### 2.10 RT-qPCR

Total RNA was isolated from RAW264.7 cells using the Total RNA Extraction Kit (BL1365A, Biosharp, China). Reverse transcription was conducted with the PrimeScript RT reagent Kit (RR047A, TaKaRa, China) to generate cDNA for subsequent RT-qPCR analysis. Amplification employed TB Green® Premix Ex Taq™ II (RR820A, TaKaRa, China) on a Quantstudio™ 3 real-time PCR system (Thermo Fisher, USA), with thermal cycling conditions of 95 °C for 1 min initial denaturation, followed by 40 cycles of 95 °C for 10 s, 55 °C for 30 s, and 72 °C for 10 s with fluorescence acquisition. The following primer sequences were used: β*-actin* (F: CTACCTCATGAAGATCCTGACC, R: CACAGCTTCTCTTTGATGTCAC); *Arg1* (F: CATATCTGCCAAAGACATCGTG, R: GACATCAAAGCTCAGGTGAATC); *Il1b* (F: CTCGCAGCAGCACATCAACAAG, R: CCACGGGAAAGACACAGGTAGC); *Il4* (F: AGTTGTCATCCTGCTCTTCTTTCTC, R: ATGGCGTCCCTTCTCCTGTG); *Nos2* (F: CAACTACTGCTGGTGGTGACAAG, R: CTGAAGGTGTGGTTGAGTTCTCTAAG); *Tgfb* (F: CCAGATCCTGTCCAAACTAAGG, R: CTCTTTAGCATAGTAGTCCGCT); *Tnf* (F: CCTGTAGCCCACGTCGTAG, R: GGGAGTAGACAAGGTACAACCC). CT values were determined using QuantStudio™ Design & Analysis SE Software (Thermo, USA). Using the 2^−△△CT^ approach, relative mRNA expression levels were computed.

### 2.11 Western blot

RAW264.7 cells and mouse aortic tissues were lysed for 30 min using cell lysis buffer (P0013, Beyotime, Shanghai, China). The tissues were homogenized for 2–5 min with a high-speed, low-temperature grinder, while cells underwent sonication in IP lysis buffer for 10–15 s. Following a 30 min incubation on ice, the samples were centrifuged at 13,800 g for 10 min at 4 °C. The protein content was measured using a BCA protein assay kit (BL521C, Biosharp, China). Proteins were then separated via SDS-PAGE (G2180-2L, Servicebio, China) and transferred onto PVDF membranes (ISEQ00010, Sigma Aldrich, USA). The membranes were incubated with a blocking solution containing 5% non-fat dry milk (GC310001, Servicebio, China) for one hour at room temperature, followed by probing with primary antibodies overnight at 4 °C. Afterward, the membranes were incubated with secondary antibodies for two hours at ambient temperature. The protein signals were detected using an ECL detection kit (BL520B, Biosharp, China) and captured using the Tanon 5200 Multi imaging system (Tanon, China). Antibodies information: β-actin, Abclonal, AC026, 1:50000; anti-PPARγ, Proteintech, 16643-1-AP, 1:5000; anti-p-STAT6, Huabio, HA722776, 1:2000; anti-STAT6, Affinity, AF6302, 1:2000; anti-CD80, Huabio, ER1905-99, 1:2000; anti-CD86, Huabio, ET1606-50, 1:5000; anti-CD163, Huabio, ER1804-03, 1:1000; anti-CD206, Cell Signaling Technology, 24595, 1:1000; Goat Anti-Rabbit IgG (H + L) HRP, Abclonal, AS014, 1:8000. The raw images of western blot were shown in (Supplementary File 2**)**.

### 2.12 Animals

Six male C57BL/6J mice (6–8 weeks old, SPF-grade) and thirty male APOE^−/−^ mice (6–8 weeks old) were obtained from SPF (Beijing) Biotechnology Co., Ltd. After one week of acclimatization with *ad libitum* access to food and water, the mice were maintained at 20–26 °C with 30%–70% relative humidity under a 12 h light/dark cycle. Housing density was standardized at five mice per cage. All procedures received ethical approval from the Laboratory Animal Ethics Committee of West China Hospital, Sichuan University (Approval No. 20250717014).

### 2.13 Animal experiments

Following 7 days of acclimatization, 30 ApoE-deficient (ApoE^−/−^) mice were randomly allocated into five groups (*n* = 6 each): the model group, low-dose YQHXF (3.51 g/kg, half the clinical equivalent), medium-dose YQHXF (7.02 g/kg, clinical equivalent), high-dose YQHXF (14.04 g/kg, twice the clinical equivalent), and positive control (atorvastatin, 5 mg/kg/d) using the random number table method. Dose selection is based on the conversion method between the body surface coefficients of mice and humans; mice dose is 9.1-fold higher than human therapeutic doses (34). Six C57BL/6J mice served as the control group. While the control group received a standard maintenance diet, all other groups were fed a high-fat diet for 6 weeks to induce hyperlipidemia. The high-fat diet and purified maintenance diet of SPF-grade were sourced from Jiangsu Xietong Pharmaceutical Bio-Engineering Co., Ltd. (Production License No.: Su Feed Certificate [2019] 01008). After the model was successfully established, as shown by notably higher serum total cholesterol (TC) and triglyceride (TG) levels in the model group compared to the control group, each groups received their respective treatments via daily intragastric gavage for 6 weeks. A similar volume of 0.9% saline was provided to the control group. Throughout the intervention period, the HFD was maintained for the treatment groups, while the control group continued with the standard diet. Atorvastatin (Batch No. 12311104) was purchased from Hunan Dino Pharmaceutical Co., Ltd. (Hunan, China). YQHXF was provided by the Affiliated Hospital of Chengdu University of Traditional Chinese Medicine (Chengdu, China), and identified according to the Chinese Pharmacopoeia 2025 version. Body weight, mental status, locomotor activity, coat luster, reaction speed, and fecal characteristics were recorded weekly. After 6 weeks, blood samples were obtained through retro-orbital bleeding before mice were anesthetized with isoflurane and euthanized. Major organs (heart, liver, spleen, lungs, and kidneys) were dissected, weighed, and fixed in 4% paraformaldehyde (PFA). The aortic arch was isolated and preserved in 4% PFA. Organ indices were determined by dividing organ weight (mg) by body weight (g). Throughout the entire experimental process, including drug intervention, sample collection, and the subsequent data analysis, blinding procedures was implemented, meaning that researchers were unaware of the specific groupings of the animals, in order to minimize human bias to the greatest extent.

### 2.14 Hematoxylin and eosin (H&E) staining

Histopathological alterations in key organs, including the heart, liver, spleen, lungs, and kidneys, were examined, with a focus on aortic abnormalities and lipid accumulation. Tissue samples were sectioned at a thickness of 5 μm and stained with hematoxylin (No. H9627, Sigma-Aldrich, USA) for 10 min, followed by a 3 min counterstain with eosin (No. YE2080, Hefei Bomei Biotechnology Co., Ltd., China). The sections were then dehydrated, cleared, and mounted using neutral balsam before being captured with a BA210 digital microscopy imaging system (Motic Industrial Group Co., Ltd., China). Evaluation of the aorta included (1) plaque, (2) endometrial thickening, degeneration and necrosis, (3) media degeneration and necrosis, (4) outer membrane dissolution, and (5) inflammatory cell infiltration. The degree of damage was expressed on a scale of 0–4 points: 0, normal; 1, slight damage; 2, mild damage; 3, moderate damage; and 4, severe damage. The total aortic injury score was calculated as the sum of the five individual scores, ranging from 0 to 20. Aortic histopathological changes were scored semiquantitatively by two independent pathologists who were blinded to the experimental groups.

### 2.15 Spectrophotometry

Serum total cholesterol (TC) and triglyceride (TG) levels were quantified using commercial assay kits provided by Nanjing Jiancheng Bioengineering Institute (China) (Total Cholesterol Assay Kit, No. A111-2-1; Triglyceride Assay Kit, No. A110-2-1). Following the manufacturer's protocol, samples underwent incubation at 37 °C for 10 min before absorbance measurements at 500 nm.

### 2.16 Enzyme-linked immunosorbnent assay (ELISA)

Mouse IL-1β ELISA Kit (No. ZC-37974), Mouse IL-4 ELISA Kit (No. ZC-37986), Mouse IL-10 ELISA Kit (No. ZC-37962), Mouse HDL ELISA Kit (No. ZC-38175), Mouse LDL ELISA Kit (No. ZC-38097), and Mouse TNF-α ELISA Kit (No. ZC-39024) were purchased from Shanghai ZCIBIO Technology Co., Ltd. (Shanghai, China). Following the manufacturer's protocol, we added 100 μL of sample or standard to each well in technical replicates, including blank controls. After incubating at 37 °C for 1–2 h, we washed the wells five times with 300 μL buffer, allowing 1 min between washes. We then added 100 μL of HRP-conjugated secondary antibody and incubated the plates for 1 h at 37 °C. Subsequently, 100 μL of TMB substrate was introduced, and the plates were protected from light for 15–30 min to facilitate color development before adding 50 μL of stop solution. Finally, absorbance at 450 nm was measured using a SpectraMAX Plus384 microplate reader (Molecular Devices, USA).

### 2.17 Immunofluorescent staining

Co-immunofluorescence staining of aortic sections detected the expression of CD86/F4/80, CD206/F4/80, p-STAT6/F4/80, and PPARγ/F4/80 proteins. After dewaxing and rehydrating paraffin-embedded sections, antigen retrieval was performed by microwave heating for 20 min. The sections were then treated with 3% hydrogen peroxide at room temperature for 25 min in the dark and washed three times with PBS (5 min per wash). Blocking was performed using bovine serum albumin (BSA, No. GC305010, Wuhan Servicebio Technology Co., Ltd.) at room temperature for 30 min. Primary antibodies were incubated overnight at 4 °C, followed by three PBS washes (5 min each). Secondary antibodies were applied at room temperature for 50 min, with three additional PBS washes (5 min each). After incubating with tyramide signal amplification (TSA) reagent in the dark for 10 min at room temperature, a second set of primary antibodies was applied overnight at 4 °C. Corresponding secondary antibodies were then incubated at 37 °C for 30 min. Finally, nuclei were counterstained with DAPI (No. G1012, Servicebio) at room temperature for 10 min. Finally, the sections were mounted with an anti-fade mounting medium, and images were acquired using an OLYMPUS VS200 scanner. The number of positively stained cells was quantified using Image-Pro Plus software. Antibodies information: anti-CD86, ER1906-01, 1:200, HUABIO; anti-CD206, ab64693, 1:200, Abcam; F4/80, 28463-1-AP, 1:400, Proteintech; anti-p-STAT6, HA722776, 1:200, HUABIO; anti-PPARγ, 16643-1-ap, 1:200, Proteintech; FITC labeled goat anti-rabbit, GB22303, 1:100, Servicebio; HRP labeled goat anti-rabbit, GB23303, 1:100, Servicebio. Tyramide signal amplification (TSA) (FITC-Tyramide), G1222, 1:500, Servicebio; TSA (CY3-Tyramide), G1223, 1:500, Servicebio.

### 2.18 Statistical analysis

Statistical analyses were performed using SPSS 25.0 (SPSS Inc., USA), with data presented as mean ± standard deviation (SD). Data normality was verified using the Shapiro–Wilk test, and variance homogeneity was assessed via Levene's test. One-way analysis of variance (ANOVA) with LSD (homogeneous variances) or Tamhane (unequal variances) *post-hoc* tests was employed for multi-group comparisons where appropriate. When variances proved heterogeneous, the Kruskal–Wallis nonparametric test was applied instead. For longitudinal data (body weight) collected repeatedly from the same animals, two-way repeated-measures ANOVA with Bonferroni multiple comparisons was employed. For cell experiments, all assays were performed using at least three independent biological replicates. Statistical significance was defined as *p* < 0.05.

## 3 Results

### 3.1 The core targets and pathways of atherosclerosis

As shown in Supplementary Figure S1A, a total of 142 atherosclerosis-related targets with degree values above 110 were retrieved from the database. The PPI network revealed that key targets (including *Il6*, *Tnf*, *Akt1*, *Il1b*, *Alb*, *Ins*, *Tp53*, *Stat3*, *Mmp9*, *Pparg*, *Tgfb1*, *Il10*, *Il4*, *etc*) exhibited strong associations with macrophage-related inflammatory responses. KEGG pathway analysis demonstrated that the “Lipid and atherosclerosis” signaling pathway was most prominently enriched (Supplementary Figure S1B). Notably, the mechanistic diagram highlights that ox-LDL can upregulate CD36 expression via PPARγ, thereby enhancing macrophage uptake of ox-LDL and promoting foam cell formation (Supplementary Figure S2) (35). These findings suggest the potential role of PPARγ signaling in macrophage polarization and lipid handling during atherosclerosis pathogenesis, providing a foundation for subsequent investigations.

### 3.2 Compounds analysis of YQHXF-CS

Using UHPLC-HRMS technology, a total of 2,317 components were detected in YQHXF-CS, including 1,272 compounds in positive ion mode (Supplementary Figure S3A) and 1,045 compounds in negative ion mode (Supplementary Figure S3B). Among these, 807 were identified as level 1, 696 were putatively annotated as level 2, and the remaining 814 were classified as level 3, referencing the Metabolomics Standards Initiative (MSI). The primary constituents comprised terpenoids, flavonoids, phenolic acids, alkaloids, lignans, and coumarins. Hesperetin 7-O-glucoside, senkyunolide C, tangeretin, salvianolic acid E, senkyunolide Q, tanshindiol B, tanshindiol C, angelicin, nobiletin, ginsenoside C, ginsenoside Rg1, ginsenoside Rb1, atractylone and other important components were detected. Detailed information on these compounds, including name, formula, molecular weight, and ionization mode, is provided in (Supplementary Table S1**)**.

### 3.3 Effects of YQHXF on body weight and organ pathology in HFD-treated mice

Mice fed a high-fat diet (HFD) showed significantly greater body weight than controls (*p* < 0.001; Figure 1A), while YQHXF treatment markedly attenuated this weight gain relative to untreated HFD mice (*p* < 0.001). The HFD group displayed elevated organ indices for liver, spleen, lungs, and kidneys (*p* < 0.05) but showed no cardiac hypertrophy. YQHXF administration specifically lowered liver and spleen indices (*p* < 0.05) without affecting lung or kidney measurements (Figure 1B). Histopathological assessment via H&E staining showed that no obvious pathological alterations were observed in the heart, spleen, or lung tissues across all groups (Figure 1C). The YQHXF-treated mice displayed no significant tissue damage or lesions in these organs, consistent with the control group. However, in the model group, hepatocytes exhibited small vacuoles of varying sizes in the cytoplasm, along with vacuolization of glomerular cells, cellular swelling, and foamy cytoplasm in the kidneys. These pathological changes were alleviated following YQHXF treatment. The results indicate that YQHXF did not cause obvious histopathological abnormalities in major organs.

**Figure 1:**
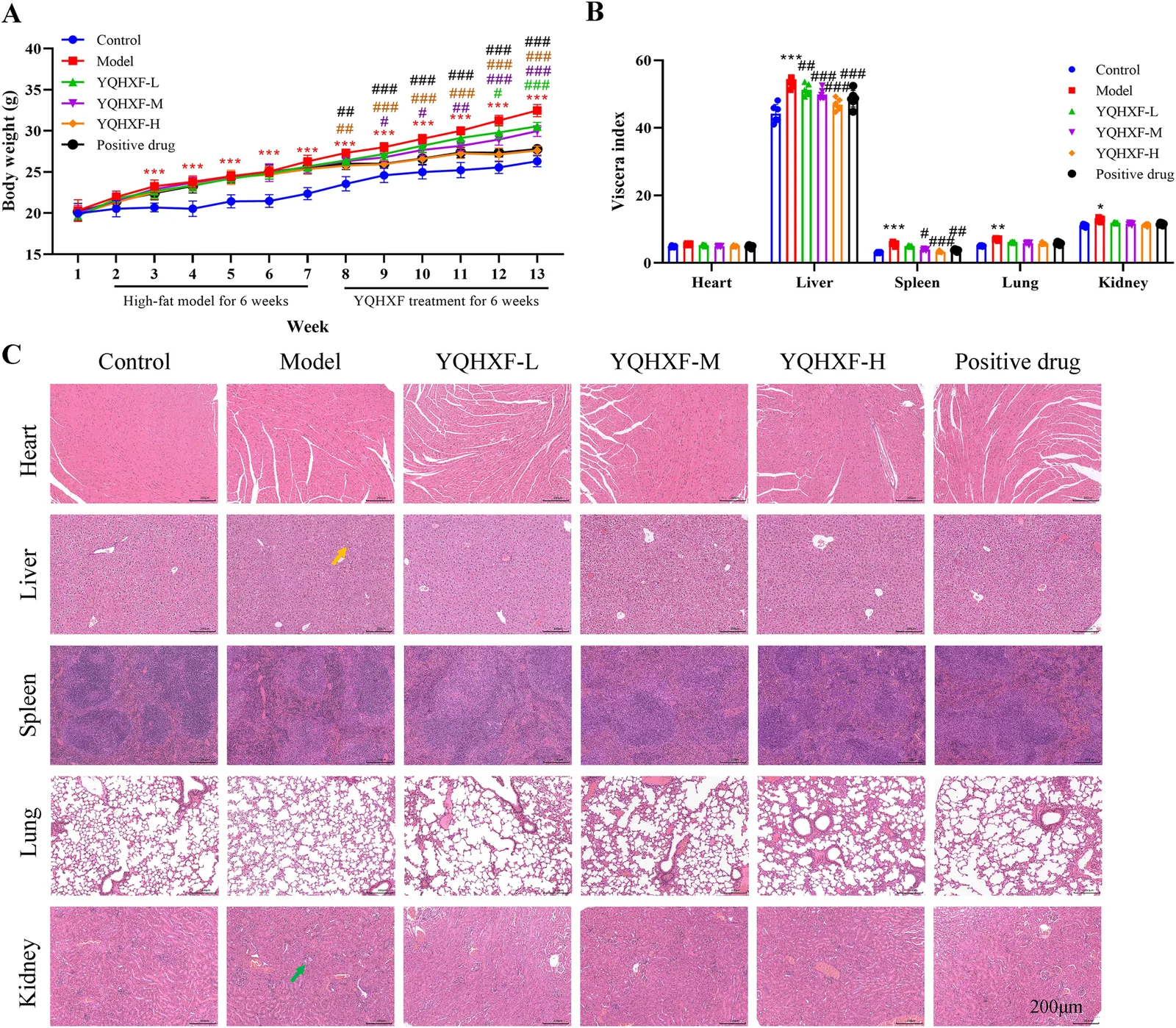
Effects of YQHXF on body weight and organ pathology in HFD-treated ApoE^−/−^ mice. **(A)** Body weight of mice. **(B)** The viscera indexes of heart, liver, spleen, lung, and kidney of mice. **(C)** H&E staining was used to observe the histopathological changes of various organs (heart, liver, spleen, lung and kidney). Scale bar = 200 μm. The yellow arrow indicates hepatocyte steatosis; green arrows indicate vacuolization of cells within the glomerulus. Values are expressed as mean ± SD (*n* = 6). Data for body weight were analyzed using two-way repeated-measures ANOVA; Data for viscera indexes were analyzed using one-way ANOVA. ^*^p < 0.05, ^**^p < 0.01, ***p < 0.001, compared with the control group; ^#^*p* < 0.05, ^##^*p* < 0.01, ^###^*p* < 0.001, compared with the model group. HFD: high-fat diet; YQHXF: Yiqihuoxue Formula.

### 3.4 YQHXF alleviated atherosclerosis in HFD-treated mice

To assess YQHXF's impact on atherosclerosis progression in mice, we quantified serum TC, TG, LDL, and HDL concentrations with commercial assay kits. Figures 2A–D demonstrates that the model group showed substantially higher TC, TG, and LDL levels (*p* < 0.001) alongside significantly lower HDL concentrations (*p* < 0.001) relative to controls. These changes were significantly reversed by treatment with low-, medium-, and high-dose YQHXF as well as the positive control drug atorvastatin (*p* < 0.05). Notably, no significant difference was observed between the high-dose YQHXF group and the atorvastatin group. As illustrated in Figure 2E,F, Oil Red O staining was performed to assess aortic plaque area. The results demonstrated that medium-, high-dose YQHXF, and atorvastatin significantly reduced the proportion of Oil Red O-positive areas (*p* < 0.05), indicating the beneficial role of YQHXF in ameliorating atherosclerotic lesions. Furthermore, H&E staining revealed distinct aortic pathological alterations and lipid deposition. The model group exhibited pronounced histopathological damage compared with the control group (*p* < 0.001), characterized by varying degrees of sclerotic plaques accompanied by fibroblast proliferation and inflammatory cell infiltration, primarily foam cells and lymphocytes. The internal elastic lamina exhibited intimal thickening, degeneration, and necrosis, with blurred cellular structures. Additionally, the medial elastic fibers were disorganized and necrotic. Treatment with medium- and high-dose YQHXF and atorvastatin led to notable improvements in these pathological alterations (*p* < 0.001) (Figure 2G,H). These findings suggest that YQHXF exerts therapeutic effects against atherosclerosis in mice by ameliorating abnormal lipid metabolism and reducing aortic lesions and lipid deposition.

**Figure 2:**
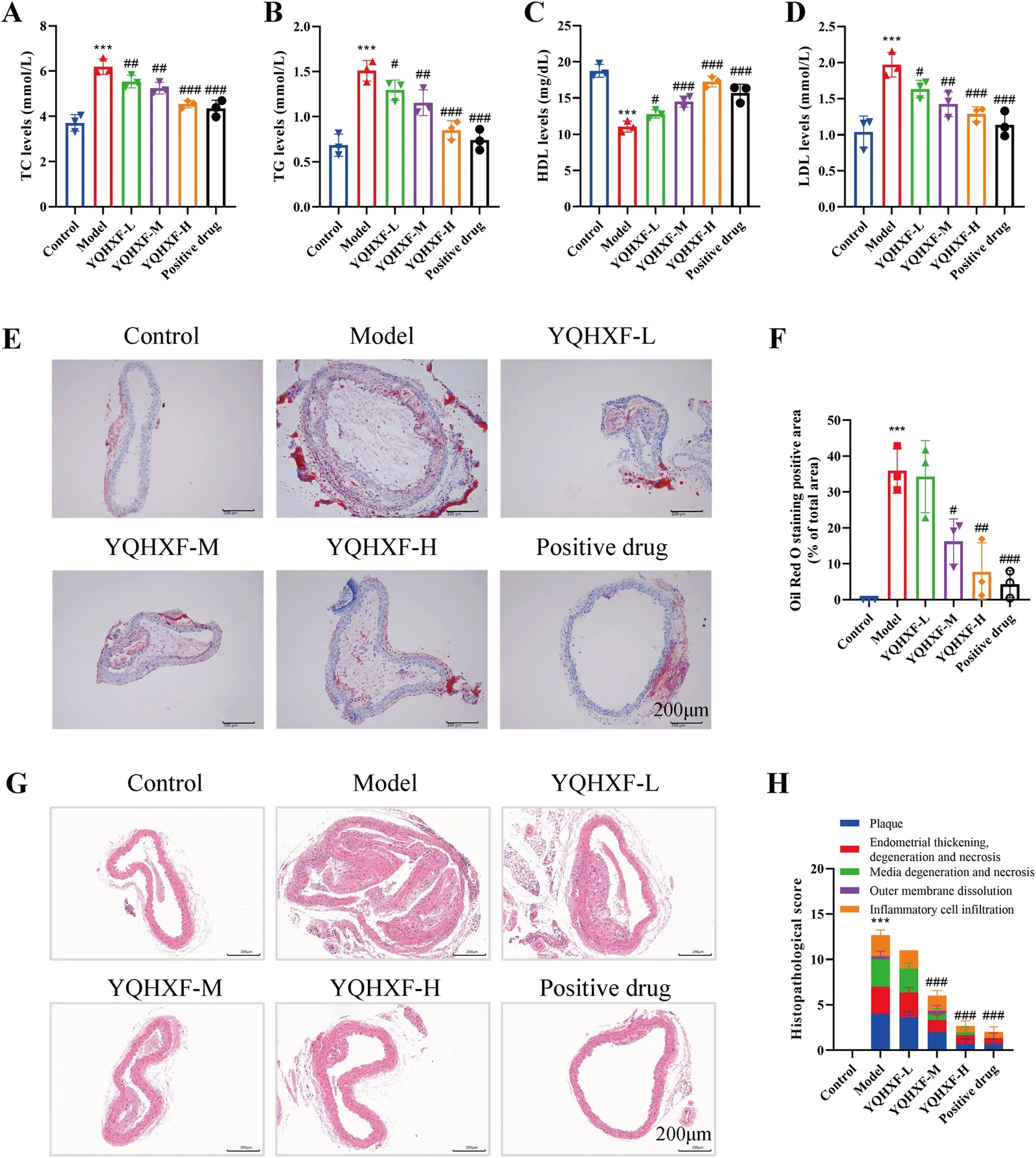
YQHXF alleviated atherosclerosis in HFD-treated ApoE^−/−^ mice. **(A)** serum TC level; **(B)** serum TG level; **(C)** serum HDL level; **(D)** serum LDL level; **(E)** Representative picture of Oil red O staining of aortic tissue under microscope, scale bar = 200 μm; **(F)** Plaque area as a percentage of total area; **(G)** Representative picture of H&E staining of aortic tissue under microscope, scale bar = 200 μm; **(H)** histopathological scores for H&E staining. The lesions were graded as normal (0 points), slight (1 point), mild (2 points), moderate (3 points) and severe (4 points). Values are expressed as mean ± SD (*n* = 3). Data were analyzed using one-way ANOVA. ^***^p < 0.001, compared with the control group; ^#^*p* < 0.05, ^##^*p* < 0.01, ^###^*p* < 0.001, compared with the model group. YQHXF, Yiqihuoxue formula; TC, total cholesterol; TG, triglycerides; HDL, high density lipoprotein; LDL, low density lipoprotein.

### 3.5 YQHXF promoted M2 macrophage polarization in HFD-treated mice, which is associated with the STAT6/PPARγ signaling pathway

To elucidate how YQHXF modulates macrophage polarization, we analyzed macrophage subsets in mouse aortic tissue using flow cytometry. The model group showed a higher proportion of CD86^+^ M1 macrophages (*p* < 0.01), a lower proportion of CD206^+^ M2 macrophages (*p* < 0.01), and an elevated M1/M2 ratio (*p* < 0.001) compared with controls (Figures 3A–D). YQHXF administration at all doses increased CD206^+^ M2 macrophage populations (*p* < 0.001) while decreasing the M1/M2 ratio (*p* < 0.001). Western blot analysis of M1 polarization markers (CD86 and CD80) and M2 polarization markers (CD163 and CD206) in aortic tissues revealed that, compared with the control group, the model group showed significantly elevated expression of CD86 and CD80 (*p* < 0.001) and significantly reduced expression of CD163 and CD206 (*p* < 0.05). YQHXF treatment reversed these changes (*p* < 0.05) (Figure 3E–H, Supplementary Figure S7). Serum levels of inflammatory cytokines (IL-1β, TNF-α, and IL-10) were quantified by ELISA. YQHXF administration at all doses significantly elevated anti-inflammatory cytokines IL-10 (*p* < 0.05) while suppressing pro-inflammatory cytokines (IL-1β and TNF-α, *p* < 0.01) relative to the model group (Figure 3I–K). Immunofluorescence co-staining revealed YQHXF markedly decreased F4/80 ^+^ CD86^+^ cell counts (*p* < 0.01) and increased F4/80^+^CD206^+^ cells (*p* < 0.01) (Figure 4A–D), indicating M1-to-M2 macrophage polarization. Western blot analysis of aortic tissues showed YQHXF modulated the STAT6/PPARγ pathway, as evidenced by altered expression of STAT6, p-STAT6, and PPARγ (Figure 5A–D). The model group displayed significantly lower p-STAT6 and PPARγ expression than controls (*p* < 0.05). YQHXF treatment at all doses effectively reversed the HFD-induced decrease in p-STAT6 and PPARγ expression (*p* < 0.01). Immunofluorescence co-staining revealed that HFD-induced reductions in F4/80 ^+^ p-STAT6^+^ and F4/80 ^+^ PPARγ^+^ cell populations in aortic tissues were significantly restored following YQHXF treatment (*p* < 0.01) (Figure 5E–H). Overall, these observations reveal that YQHXF modulates the M1/M2 balance, an effect associated with enhanced STAT6/PPARγ signaling pathway, which in turn mitigates HFD-induced atherosclerosis in ApoE^−/−^ mice.

**Figure 3:**
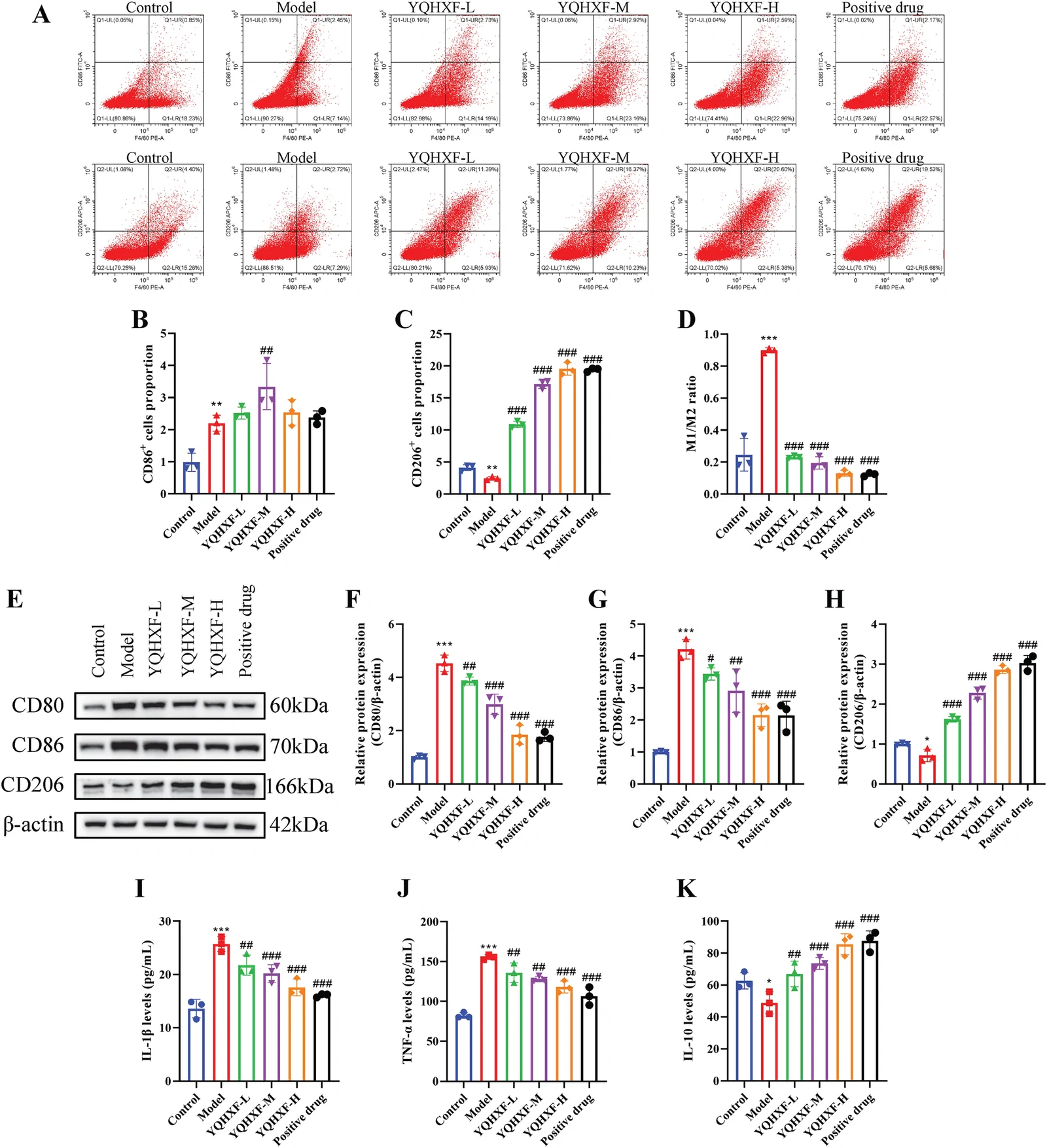
YQHXF regulated macrophage polarization in HFD-treated ApoE^−/−^ mice. **(A)** M1 (CD86^+^) and M2 (CD206^+^) macrophages in mouse aorta were detected by flow cytometry. **(B)** Proportion of CD86^+^ cells; **(C)** Proportion of CD206^+^ cells; **(D)** Proportion of M1/M2 macrophages; **(E)** Representative pictures of CD80, CD86, and CD206 protein expression detected by Western blot; β-actin was used to normalize the data. **(F–H)** Quantitative analysis of relative CD80 **(F)**, CD86 **(G)**, and CD206 **(H)** proteins expression. **(I)** Serum IL-1β level; **(J)** Serum TNF-α level; **(K)** Serum IL-10 level. Values are expressed as mean ± SD (*n* = 3). Data were analyzed using one-way ANOVA. *p < 0.05, ^**^p < 0.01, ***p < 0.001, compared with the control group; ^#^*p* < 0.05, ^##^*p* < 0.01, ^###^*p* < 0.001, compared with the model group. HFD: high-fat diet; YQHXF: Yiqihuoxue Formula.

**Figure 4:**
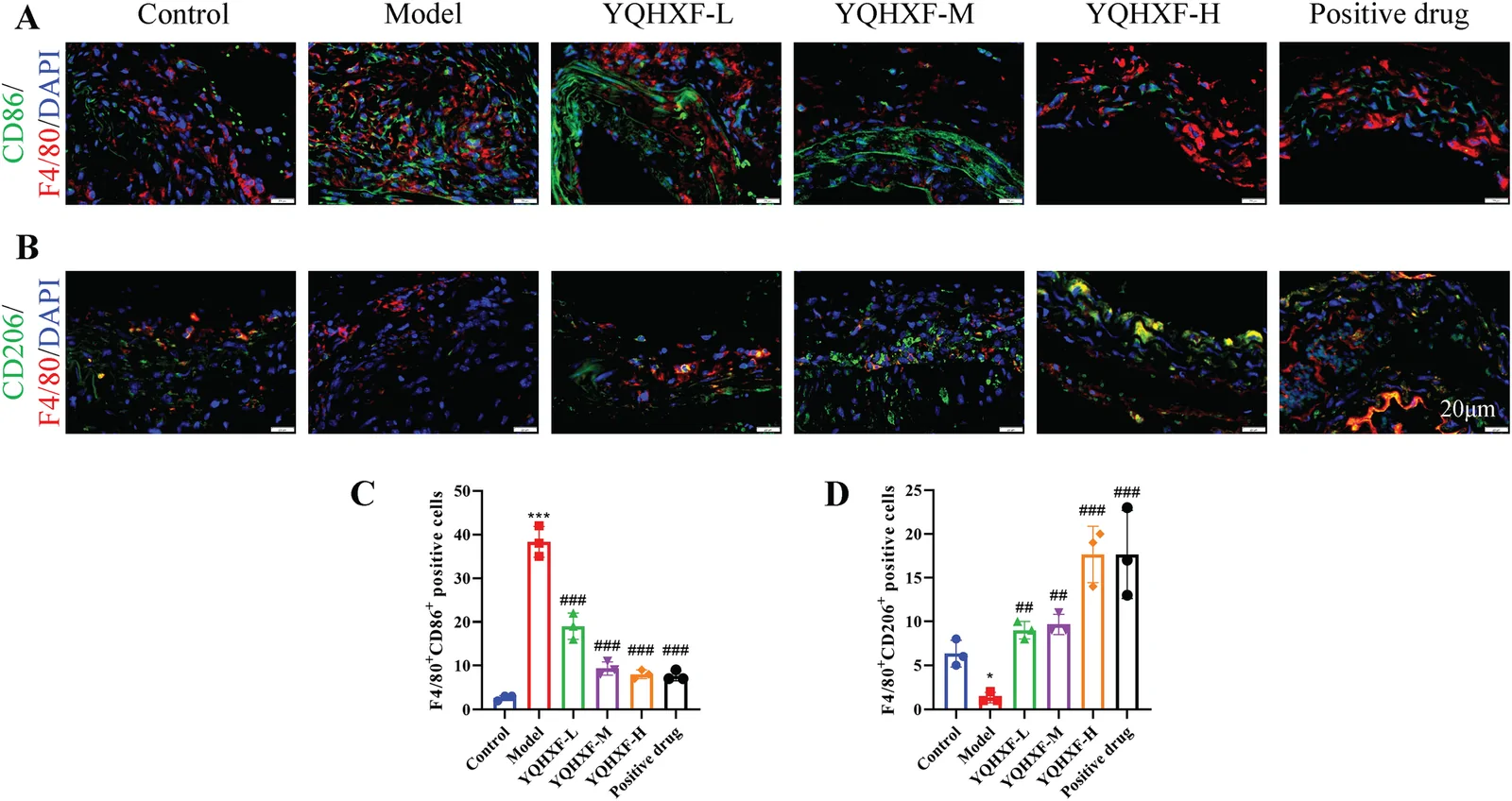
YQHXF promoted M2 macrophage polarization in mice. (**A and B)** Representative immunofluorescence images of macrophage distribution. **(C)** Immunofluorescence co-staining for detection the number of F4/80^+^ CD86^+^ positive cells (M1 macrophages). **(D)** Immunofluorescence co-staining for detection the number of F4/80^+^CD206^+^ positive cells (M2 macrophages). Scale bar = 20 μm. Values are expressed as mean ± SD (*n* = 3). Data were analyzed using one-way ANOVA. *p < 0.05, ^***^p < 0.001, compared with the control group; ^##^*p* < 0.01, ^###^*p* < 0.001, compared with the model group. YQHXF, Yiqihuoxue formula.

**Figure 5:**
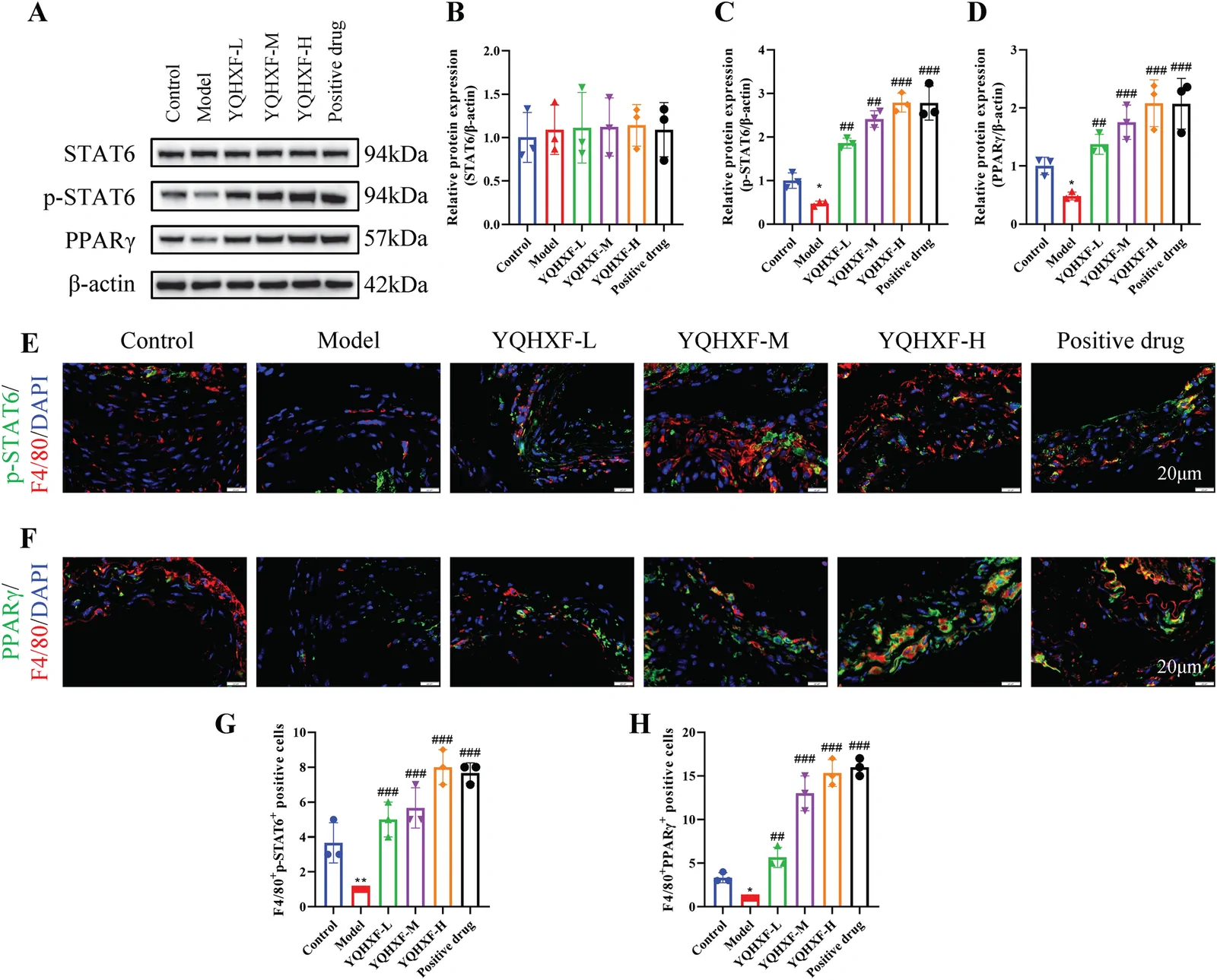
YQHXF promoted M2 macrophage polarization in HFD-treated mice is associated with STAT6/PPARγ signaling pathway. **(A)** Representative picture of STAT6, p-STAT6, and PPARγ proteins expression detected by Western blot; **(B–D)** Quantification of STAT6 **(B)**, p-STAT6 **(C)**, and PPARγ **(D)** proteins relative expression. β-actin was used to normalize the data. **(E,F)** Representative immunofluorescence images of F4/80 ^+^ p-STAT6^+^ **(E)** and F4/80 ^+^ PPARγ^+^ **(F)** cells distribution. (**G,H)** Quantitative analysis of F4/80 ^+^ p-STAT6^+^ **(G)** and F4/80 ^+^ PPARγ^+^ **(H)** positive cells. Scale bar = 20 μm. Values are expressed as mean ± SD (*n* = 3). Data were analyzed using one-way ANOVA. ^*^p < 0.05, ^**^p < 0.05, compared with the control group; ^##^*p* < 0.01, ^###^*p* < 0.001, compared with the model group. HFD: high-fat diet; YQHXF: Yiqihuoxue formula.

### 3.6 YQHXF-CS enhanced cell viability of ox-LDL-exposed RAW264.7 cells

Firstly, a foam cell model was established in RAW264.7 cells using ox-LDL. The cells underwent treatment with YQHXF-CS at varying concentrations of 0%, 1%, 2%, 3%, 4%, and 5% for 24 h, and cell viability was then evaluated using the CCK-8 assay. Compared with the BC + 0% YQHXF-CS group, the ox-LDL+0% YQHXF-CS group exhibited a significant reduction in cell viability (*p* < 0.05). Similar trends were observed between the ox-LDL + 1% YQHXF-CS and BC + 1% YQHXF-CS groups, as well as between the ox-LDL + 2% YQHXF-CS and BC + 2% YQHXF-CS groups (*p* < 0.05). Notably, treatment with 5% YQHXF-CS markedly improved viability relative to the ox-LDL + 0% control (*p* < 0.001) (Supplementary Figure S4). These results indicate that YQHXF-CS could enhance cell viability of RAW264.7 cells.

### 3.7 YQHXF-CS promoted macrophages polarization to M2 type and alleviated foam cell formation *in vitro*

Based on the aforementioned findings, 5% YQHXF-CS was selected as the optimal concentration. RAW264.7 cells were treated with low (1.25%), medium (2.5%), and high (5%) concentrations of YQHXF-CS for 24 h. CCK-8 assay demonstrated that the high-dose YQHXF-CS group significantly reversed the decreased cell viability compared with the ox-LDL + BS group (*p* < 0.05) (Figure 6A,B). Oil Red O staining revealed a marked increase in red-stained lipid droplets distributed throughout the cytoplasm in the ox-LDL + BS group compared with the BC group, with some cells exhibiting lipid droplet aggregation, indicating foam cell formation in RAW264.7 cells (*p* < 0.05) (Figure 6C). Treatment with high-dose YQHXF-CS significantly reduced the number of red lipid droplets in ox-LDL-induced RAW264.7 cells (*p* < 0.05) (Figure 6D). To examine how YQHXF influences macrophage polarization, we measured mRNA expression of M1 markers (*Nos2*, *Tnf*, and *Il1b*) and M2 markers (*Arg1* and *Tgfb*) using RT-qPCR. Ox-LDL markedly increased *Nos2*, *Tnf*, and *Il1b* expression in RAW264.7 cells (*p* < 0.001; Figure 6E), while YQHXF-CS at all tested doses suppressed these M1 markers (*p* < 0.05). Although the ox-LDL + BS group showed no significant changes relative to baseline controls, YQHXF-CS treatment elevated *Arg1* and *Tgfb* expression across all doses (*p* < 0.05). Flow cytometry revealed that YQHXF-CS decreased CD80/CD86-positive M1 macrophages while increasing CD163/CD206-positive M2 populations (Supplementary Figure S5A–E), consistent with its polarization effects. These findings indicate that YQHXF-CS attenuates foam cell formation, and this effect is associated with a shift from M1 to M2 macrophage phenotypes.

**Figure 6:**
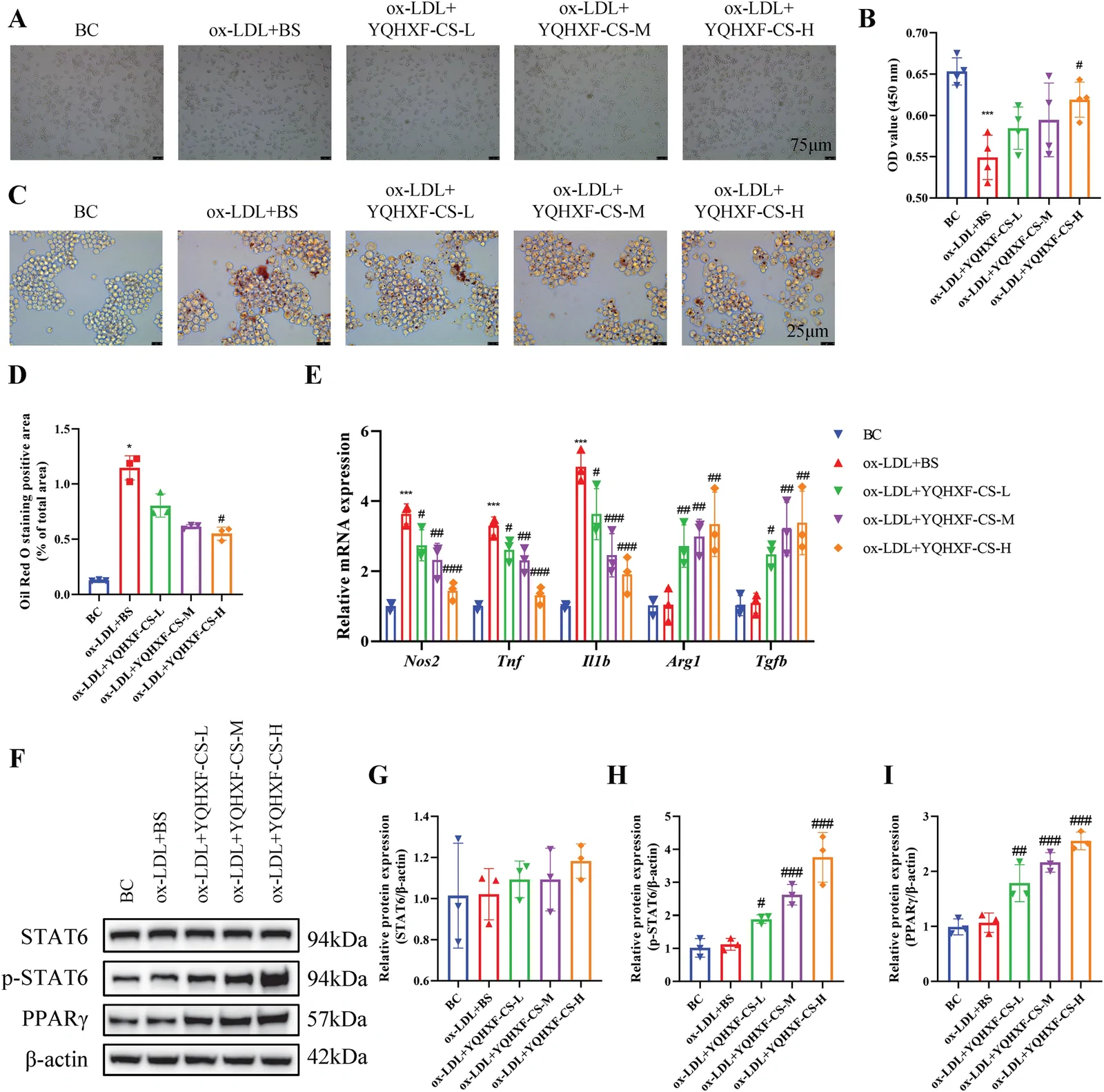
YQHXF-CS regulated macrophage differentiation and alleviated macrophage foam cell formation. **(A)** Morphology of RAW264.7 cells under microscopy. **(B)** Absorbance (OD) at 450 nm was determined using a microplate reader (*n* = 4). Higher OD values indicate stronger cell viability. Scale bar = 75 μm. **(C)** Representative images of Oil Red O-stained RAW264.7 cells under microscopy. Scale bar = 25 μm. **(D)** Proportion of Oil Red O-positive area (*n* = 3). **(E)** RT-qPCR analysis of M1-like macrophage activation markers (*Nos2*, *Tnf*, and *Il1b*) and M2-like macrophage activation markers (*Arg1* and *Tgfb*) (*n* = 3). **(F)** Representative Western blotting images of STAT6, p-STAT6, and PPARγ protein expression. β-actin was used to normalize the data. **(G–I)** Quantitative analysis of relative STAT6 **(G)**, p-STAT6 **(H)**, and PPARγ **(I)** proteins expression (*n* = 3). Values are expressed as mean ± SD. Data were analyzed using one-way ANOVA. *p < 0.05, ***p < 0.001 *vs.* BC group; ^#^*p* < 0.05, ^##^*p* < 0.01, ^###^*p* < 0.001 vs. ox-LDL + BS group. BC, blank control; BS: blank serum; YQHXF-CS, Yiqihuoxue formula containing serum; ox-LDL, oxidized low-density lipoprotein.

### 3.8 YQHXF-CS promoted macrophage M2 polarization, an effect associated with enhanced STAT6/PPARγ signaling pathway *in vitro*

To explore the molecular mechanism by which YQHXF-CS promotes M2 polarization, we examined the STAT6/PPARγ signaling. Western blot analysis of the STAT6/PPARγ pathway demonstrated that ox-LDL did not affect STAT6, p-STAT6, or PPARγ protein levels (Figure 6F–I). In contrast, YQHXF-CS treatment upregulated p-STAT6 and PPARγ expression (*p* < 0.05), suggesting a possible association between this pathway and YQHXF-CS treatment. To further confirm the role of PPARγ in this process, RAW264.7 cells were transfected with either siRNA-NC or siRNA-PPARγ, followed by incubation with 100 μg/mL ox-LDL for 24 h. PPARγ mRNA and protein expression levels decreased significantly following siRNA transfection (Figure 7A–C). The CCK-8 assay showed that siRNA-PPARγ substantially reduced cell viability, an effect partially counteracted by YQHXF-CS (*p* < 0.001) (Figure 7D,E). YQHXF-CS also partially mitigated the siRNA-PPARγ and ox-LDL-induced foam cell formation observed in Oil Red O staining (Figure 7F,G). Flow cytometry revealed that siRNA-PPARγ and ox-LDL elevated CD80^+^ and CD86^+^ cell populations while reducing CD163^+^ and CD206^+^ populations, changes partially reversed by YQHXF-CS treatment (both *p* < 0.05) (Supplementary Figure S6). Quantitative RT-qPCR analysis demonstrated that siRNA-PPARγ transfection alone upregulated M1 markers (*Nos2*, *Tnf*, and *Il1b*) and downregulated M2 markers (*Arg1* and *Tgfb*) compared to the siRNA-NC group (*p* < 0.001 and *p* < 0.05, respectively) (Figure 8A–E). This pattern persisted in the siRNA-PPARγ + ox-LDL + BS group vs. siRNA-NC + ox-LDL + BS controls (*p* < 0.001). Ox-LDL treatment exacerbated these transcriptional shifts (*p* < 0.05), confirming its role in M1 polarization, whereas YQHXF-CS partially reversed these effects (*p* < 0.05), indicating promotion of M2 polarization. Western blot analysis showed siRNA-PPARγ reduced p-STAT6 and PPARγ protein levels relative to siRNA-NC (*p* < 0.05), and YQHXF-CS partially upregulated ox-LDL-suppressed p-STAT6 and PPARγ expression (*p* < 0.05) (Figure 8F–I). In summary, these findings suggest that YQHXF-CS promotes macrophage polarization toward the M2 phenotype, and this effect is associated with enhanced STAT6/PPARγ signaling. However, PPARγ knockdown only partially reversed these effects.

**Figure 7:**
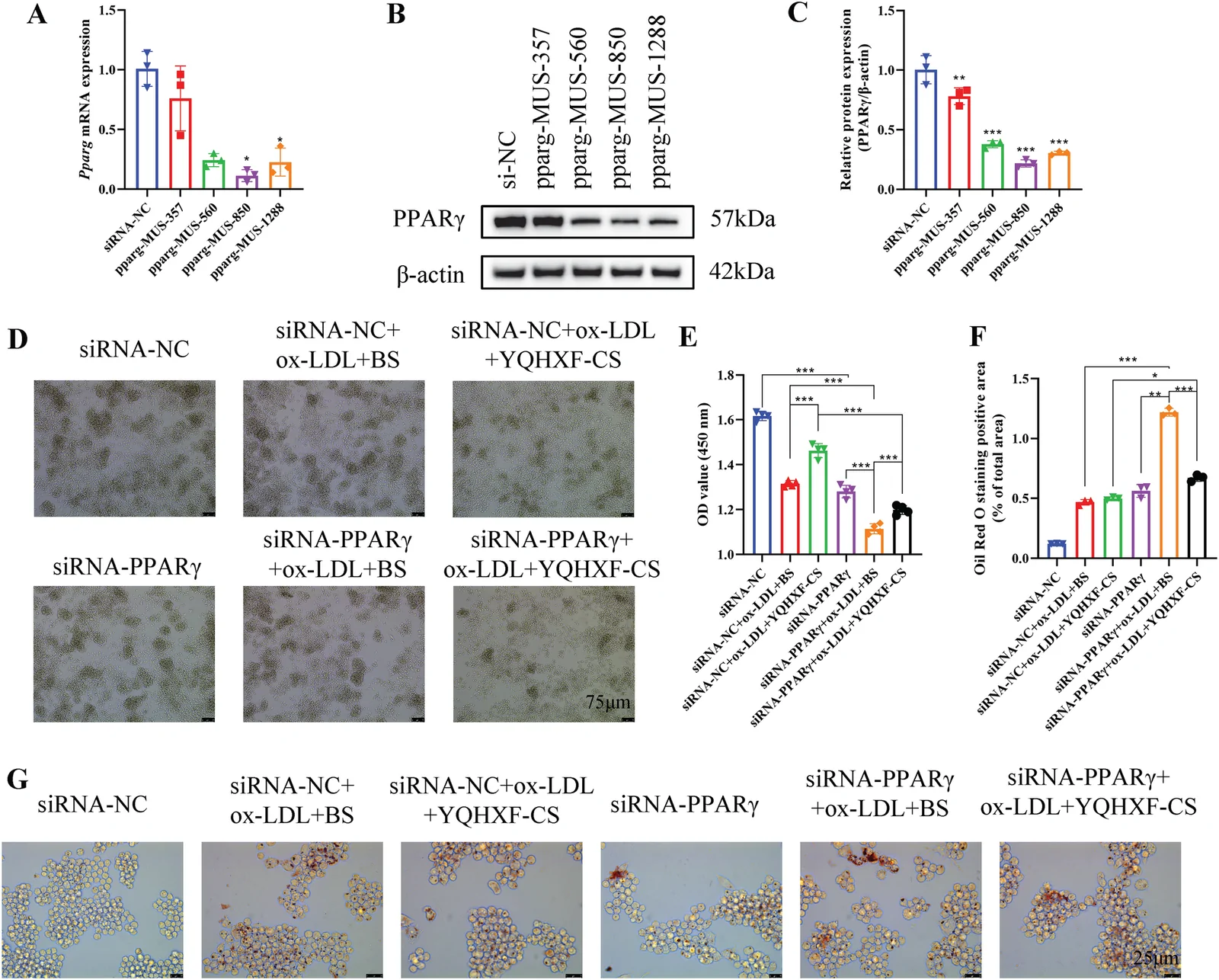
YQHXF-CS alleviated macrophage foam cell formation and lipid deposition *in vitro*. **(A–C)** The transfection efficiency of siRNA-PPARγ was detected by RT-qPCR **(A)** and **(B,C)** Western blotting (*n* = 3). ^*^p < 0.05, ^**^p < 0.01, ***p < 0.001 vs. siRNA-NC group. **(D,E)** Cell viability was detected by CCK8 assay (*n* = 4). Scale bar = 75 μm. (**F,G)** Representative pictures of Oil red O staining of macrophage and quantification of Oil Red O-stained positive area (*n* = 3). Scale ba*r* = 25 μm. Values are expressed as mean ± SD. Data were analyzed using one-way ANOVA. *p < 0.05, ^**^p < 0.01, ***p < 0.001.

**Figure 8:**
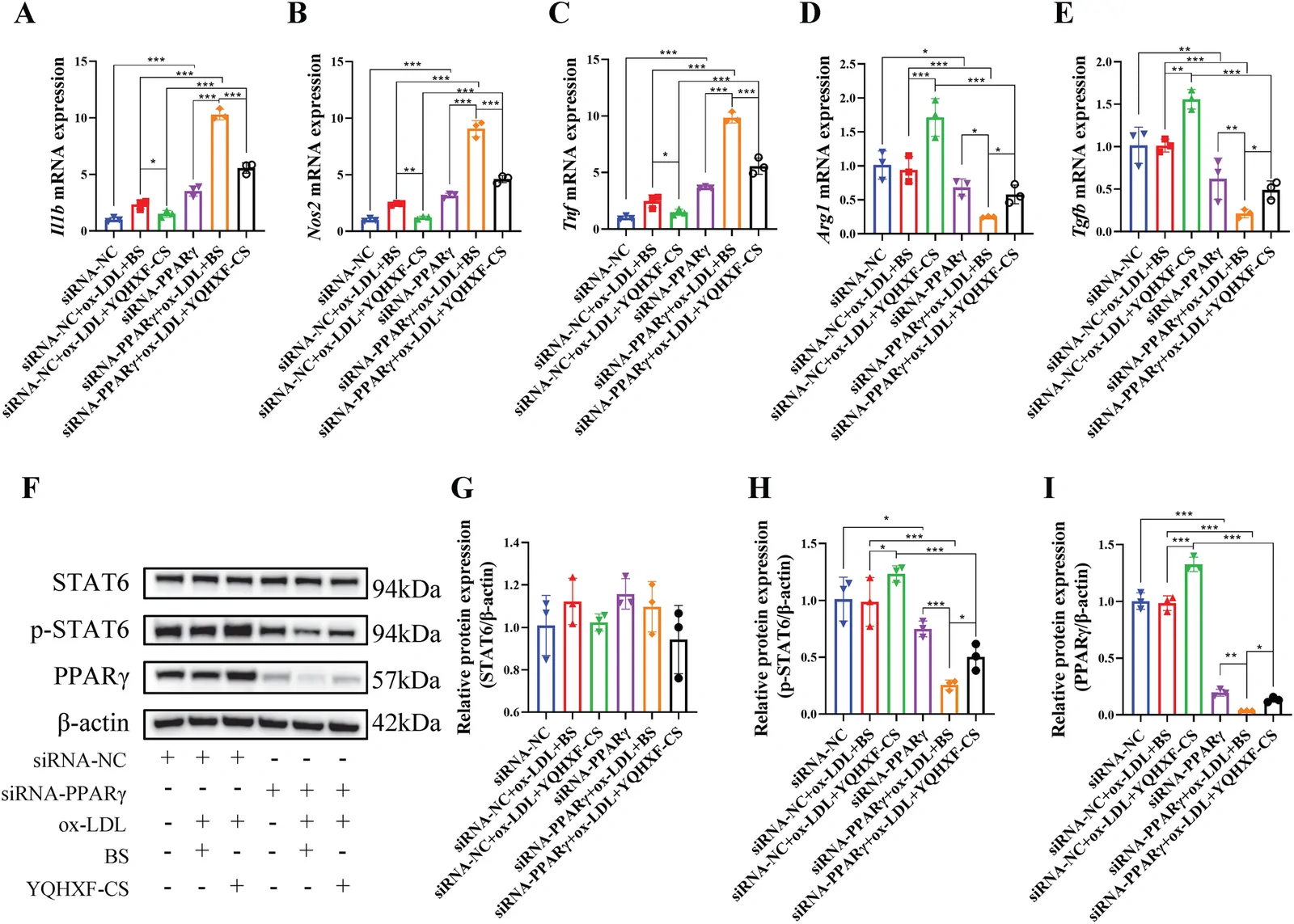
YQHXF-CS promoted macrophage M2 differentiation and alleviate foam cell formation is associated with PPARγ signaling pathway. **(A–E)** *Il1b* **(A)**, *Nos2* **(B)**, *Tnf* **(C)**, *Arg1* **(D)**, and *Tgfb* **(E)** mRNA expression levels were detected by RT-qPCR. **(F)** Representative picture of STAT6, p-STAT6 and PPARγ protein expression detected by Western blotting. β-actin was used to normalize the data. **(G–I)** Quantitative analysis of STAT6 **(H)**, p-STAT6 **(I)**, and PPARγ **(J)** proteins expression. Values are expressed as mean ± SD (*n* = 3). Data were analyzed using one-way ANOVA. *p < 0.05, ^**^p < 0.01, ***p < 0.001. BS, blank serum; YQHXF-CS, Yiqihuoxue formula containing serum; ox-LDL, oxidized low-density lipoprotein.

## 4 Discussion

This study employed systems biology, pharmacological approaches, and molecular biology techniques to elucidate the multi-level mechanism by which YQHXF treats AS. Network analysis identified *Il6*, *Tnf*, *Akt1*, *Stat3*, and *Pparg* and others as core targets modulating the “lipids and AS” pathway (KEGG enrichment), with *Pparg* emerging as a critical node for ox-LDL involvement in lipid metabolism and AS pathogenesis through macrophage polarization regulation. Beyond *Pparg*, several other core targets are intimately linked to inflammation and macrophage polarization. *Il6* and *Tnf* are prototypical pro-inflammatory cytokines that not only promote M1 polarization but also drive endothelial activation and plaque instability. *Akt1*, a key node in the PI3K/Akt signaling axis, regulates macrophage survival, metabolism, and the balance between M1 and M2 phenotypes. *Stat3*, as a member of the STAT family, can be activated by *Il6* and other cytokines, and its sustained activation is associated with chronic inflammation and impaired M2 polarization. Although the present study focused on the STAT6/PPARγ pathway, the network-derived targets suggest that YQHXF may simultaneously modulate these intersecting pro-inflammatory signals. This is consistent with the multi-component, multi-target nature of traditional Chinese medicine formulas. Future studies are warranted to experimentally verify whether YQHXF regulates the IL-6/STAT3 or PI3K/AKT1 axis to fine-tune macrophage polarization and plaque inflammation. Chemical characterization revealed 2,317 bioactive constituents in YQHXF, including ginsenosides, salvianolic acid, and ligusticolactone, which known as their blood-activating properties. *In vitro* experiments demonstrated that YQHXF stimulates M2 macrophage polarization and suppresses ox-LDL-induced foam cell formation, an effect is associated with enhanced STAT6/PPARγ pathway. The ApoE^−/−^ mouse model confirmed YQHXF's lipid-lowering and anti-atherosclerotic effects, which were associated with M1/M2 balance modulation and inflammatory response inhibition. These findings establish a pharmacological foundation for YQHXF's therapeutic application while bridging the principle of “qi-tonifying and blood-activating” in TCM with macrophage polarization mechanisms.

TCM attributes AS pathogenesis to “qi deficiency and blood stasis,” with “qi-tonifying and blood-activating” as the primary therapeutic strategy. The HFD-induced ApoE^−/−^ mouse model successfully recapitulated key pathological features including dyslipidemia, endothelial dysfunction, and plaque formation. YQHXF treatment significantly reduced TC, TG, and LDL-C while elevating HDL-C, diminished aortic plaque area, and improved vascular wall pathology, and these results aligned with previous clinical observations. Notably, high-dose YQHXF exhibited efficacy comparable to atorvastatin. At the cellular level, YQHXF-CS preferentially induced ox-LDL-stimulated RAW264.7 macrophages toward the M2 phenotype, evidenced by elevated CD206^+^/CD163^+^ populations and reduced M1 markers (CD80^+^, CD86^+^). Mechanistic studies verified STAT6 phosphorylation (p-STAT6) and PPARγ upregulation by YQHXF-CS, and these effects were partially attenuated by PPARγ silencing (siRNA-PPARγ), suggesting possible PPARγ-independent mechanisms rather than complete pathway dependence.

Macrophage polarization critically influences the pathogenesis of AS through its immunomodulatory functions (36, 37). M1-polarized macrophages release proinflammatory cytokines including IL-6, IL-1β, TNF-α, IL-12, and IL-23, intensifying vascular inflammation and promoting foam cell development (21). Conversely, IL-4 and IL-13 stimulation induces M2 macrophages to generate anti-inflammatory factors such as IL-10, TGF-β, and Arg1, which counteract M1-mediated inflammation while enhancing plaque lipid metabolism and tissue regeneration (20). Studies have shown that the combination of CD206, Arg1, and IL-10 is widely accepted in the field for defining murine M2 polarization (38). Our *in vitro* experiments demonstrated that YQHXF-CS treatment markedly decreased mRNA levels of M1 markers (*Nos2*, *Tnf*, and *Il1b*) while elevating M2 markers (*Arg1* and *Tgfb*) in RAW264.7 cells. Flow cytometry revealed YQHXF-CS reduced CD80^+^/CD86^+^ M1 macrophages while increasing CD163^+^/CD206^+^ M2 populations. In ApoE^−/−^ mice, YQHXF treatment elevated aortic CD206^+^ M2 macrophage prevalence and lowered the M1/M2 ratio. These findings indicate YQHXF facilitates macrophage repolarization from proinflammatory M1 to anti-inflammatory M2 states, potentially slowing AS. It should be noted that the significant improvement in serum lipid profiles achieved by YQHXF may partially explain the shift in macrophage phenotype observed *in vivo*. A reduction in circulating LDL-C and an increase in HDL-C lower the lipid burden within the arterial wall, which can attenuate foam cell formation and create a less inflammatory microenvironment that naturally favors M2 polarization. Therefore, the anti-atherosclerotic effect of YQHXF likely involves both lipid-lowering-dependent and STAT6/PPARγ pathway-associated mechanisms. This mechanism aligns with the theory of “phlegm-blood stasis accumulation” in TCM, where M1 overactivation mirrors “toxic pathogen accumulation” and M2 transformation reflects “blood circulation activation and stasis resolution” therapeutic effects (39). This study evaluated M2 polarization mainly based on morphological changes, surface markers, and cytokine alterations. Although these results are highly consistent with an M2 phenotype, future studies incorporating functional assays—such as direct detection of efferocytosis and cholesterol efflux capacity—would provide more direct evidence for the anti-atherosclerotic efficacy of YQHXF-CS.

Regarding the role of PPARγ in lipid handling, it is important to note that PPARγ exerts dual function on macrophage lipid metabolism. On the one hand, PPARγ can upregulate CD36 and promote ox-LDL uptake, thereby potentially favoring foam cell formation (40). On the other hand, PPARγ may enhance cholesterol efflux through the LXR*α*–ABCA1/ABCG1 pathway and simultaneously promote anti-inflammatory M2 polarization, which can limit foam cell formation (41). In this study, we did not directly assess CD36, ABCA1, ABCG1, or LXR*α* expression, nor did we perform ox-LDL uptake or cholesterol efflux assays. Therefore, the reduction in foam cell formation observed after YQHXF-CS treatment should not be solely attributed to PPARγ-mediated suppression of lipid accumulation. Instead, it may reflect the combined effects of enhanced M2 polarization, possible lipid handling modulation, and other PPARγ-independent mechanisms. Further experiments are needed to clarify the contribution of PPARγ to foam cell formation.

STAT6 and PPARγ serve as core transcription factors regulating macrophage polarization. IL-4/IL-13 signaling induces M2 polarization through STAT6 activation, whereas PPARγ, a nuclear receptor superfamily member, directly suppresses proinflammatory pathways like NF-*κ*B while upregulating M2 marker expression (32, 42). Our experiments demonstrated that YQHXF treatment partially reversed the decreased p-STAT6 and PPARγ levels observed in the AS model, with PPARγ knockdown partially abolishing YQHXF's anti-foam cell effects, suggesting that the STAT6/PPARγ axis may be an important contributor to the effects of YQHXF rather than an established primary target. Mechanistic studies revealed that STAT6 activation creates a positive feedback loop by binding to the PPARγ promoter and enhancing its transcription (43, 44). Activated PPARγ subsequently suppresses STAT1/3-mediated M1 pathways while reinforcing M2 polarization, illustrating the bidirectional regulation underlying YQHXF's multi-target therapeutic effects. However, this study did not directly test this cascade, and the hierarchical relationship between STAT6 and PPARγ remains to be experimentally established. Additionally, our study highlighted the differences in the effects of acute *in vitro* stimuli vs. chronic *in vivo* disease environments on STAT6/PPARγ signaling. *In vitro*, acute treatment with ox-LDL for 24 h may be insufficient to directly suppress STAT6/PPARγ protein expression within such a short timeframe. Under these conditions, the p-STAT6/PPARγ pathway may remain in an “un-activated” or “basal” state, rather than being suppressed. The role of YQHXF-CS in this context is to “activate” this pathway, thereby promoting macrophage polarization toward the M2 phenotype. *In vivo* experiments, the high-fat diet represents a chronic process lasting several months. This leads to a complex, systemic pathological environment characterized by persistent hyperlipidemia, chronic inflammation, and abundant pro-inflammatory cytokines. In such an environment, prolonged inflammatory signaling may suppress anti-inflammatory pathways such as STAT6/PPARγ, resulting in the downregulation of their protein expression and phosphorylation levels. Therefore, the role of YQHXF in this context is a genuine “restoration”, and elevating the suppressed pathway activity. Interestingly, although our data strongly suggest that the STAT6/PPARγ axis is a key mediator of YQHXF–CS-regulated M2 polarization, the partial protective effects observed in PPARγ-knockdown cells indicate the potential involvement of other mechanisms. This is consistent with the multi-component nature of YQHXF–CS, which may simultaneously modulate other signaling pathways to achieve its overall anti-atherosclerotic therapeutic effects. Future studies incorporating PPARγ overexpression or agonists/antagonists are warranted to further validate the role of PPARγ.

YQHXF consists of Hong-Shen, Bai-Zhu, Dan-Shen, Chuan-Xiong, and San-Qi, embodying the traditional principle of “fortifying qi and resolving blood stasis.” Hong-Shen and Bai-Zhu tonify the qi of the spleen and lungs; Dan-Shen and Chuan-Xiong promote blood circulation; San-Qi addresses both stasis and hemorrhage. Modern pharmacological studies have shown that YQHXF contains a variety of active ingredients, with anti-inflammatory, anti-oxidative, and lipid-regulating properties. In particular, tanshindiol C has been reported to inhibit ox-LDL-induced macrophage foam cell formation, thereby intervening in early AS (45). Salvianolic acid B targets PPAR*α* and participates in lipid metabolism regulation while alleviating oxidative stress and inflammatory responses (46). Ginsenosides can activate PPARγ, regulate oxidative stress, and reduce blood lipids, thus improving AS (47, 48). Ligustrazine activates PPARγ and contributes to vascular remodeling (49, 50). Nobiletin modulates macrophage inflammation via PPARγ signaling and demonstrates hepatoprotective and lipid-lowering properties (51, 52). Collectively, these compounds, reported to target inflammation, lipid metabolism, or PPARγ signaling, provide a plausible pharmacological foundation for the observed association between YQHXF treatment and enhanced M2 macrophage polarization/STAT6/PPARγ signaling.

However, there are some limitations to this study. First, classic positive control drugs were not set in the *in vitro* phenotype experiments, which limited the ability to evaluate the effect of YQHXF-CS to some extent. Although the YQHXF-CS provides a holistic perspective, subsequent studies will focus on identifying and purifying key active monomers from YQHXF-CS, or isolating active fractions using preparative chromatography, followed by functional validation in the same model system to further verify the core efficacious constituents. The direct binding interactions between YQHXF's components and the STAT6/PPARγ pathway require further validation, given herbal medicine's multi-target nature. While ApoE^−/−^ mice represent a standard AS model, their high-fat-diet-induced pathology differs from human AS progression, necessitating large-animal verification. We also note that future studies using ApoE⁻/⁻ mice on a chow diet as an additional control would be beneficial to completely rule out strain-specific effects. Although the involvement of STAT6/PPARγ is suggested by the present findings, potential crosstalk with NF-*κ*B or PI3K/Akt pathways warrants investigation using inhibitors or knockout models. Although we have confirmed that YQHXF can upregulate p-STAT6 and PPARγ, and that the knockout of PPARγ can partially reduce the level of p-STAT6, the direct dependence relationship between PPARγ and STAT6 has not yet been verified through experiments. In the future, *in vivo* studies on STAT6 inhibition or PPARγ antagonists, or siRNA-mediated knockout should be carried out to clarify the regulatory cascade of STAT6/PPARγ and its role in M2 polarization. Future clinical studies should explore correlations between qi deficiency/blood stasis patterns and macrophage polarization, alongside evaluating YQHXF's combinatorial potential with statins or anti-inflammatory agents. YQHXF is expected to provide an alternative or complementary therapeutic strategy for the development of a novel TCM preparation targeting atherosclerosis through immunomodulatory pathways. However, its translation into clinical practice still requires further large-scale clinical studies, standardized quality control, and long-term safety evaluations.

## 5 Conclusions

Based on the aforementioned findings, our study demonstrates that YQHXF facilitates macrophage polarization from M1 to M2 phenotype, an effect associated with STAT6/PPARγ pathway *in vitro*, this phenotypic shift may contribute to the mitigation of macrophage foam cell formation. Furthermore, YQHXF ameliorates dysregulated lipid metabolism *in vivo*, attenuates aortic lesions and lipid deposition, and alleviates atherosclerosis in ApoE^−/−^ mice, these beneficial effects were accompanied by enhanced STAT6/PPARγ signaling and increased M2 macrophage polarization. This study clarifies the therapeutic effects and molecular mechanisms of YQHXF against atherosclerosis from a contemporary medical standpoint. The results offer substantial experimental evidence for employing TCM atherosclerosis management, with both important theoretical implications and clinical potential.

## Data Availability

The datasets presented in this study can be found in online repositories. The names of the repository/repositories and accession number(s) can be found in the article/Supplementary Material.
