# Supplementary material for: Yiqihuoxue formula improves atherosclerosis by regulating macrophage polarization associated with enhanced STAT6/PPARγ pathway
Source: Front Cardiovasc Med. 2026 Sep 22;13:1896890. doi: 10.3389/fcvm.2026.1896890 (PMC13638390; doi:10.3389/fcvm.2026.1896890)

Supplementary file 1. Raw data of flow cytometry

(1) The raw data of flow cytometry in Figure 3A

Figure 3A Blank

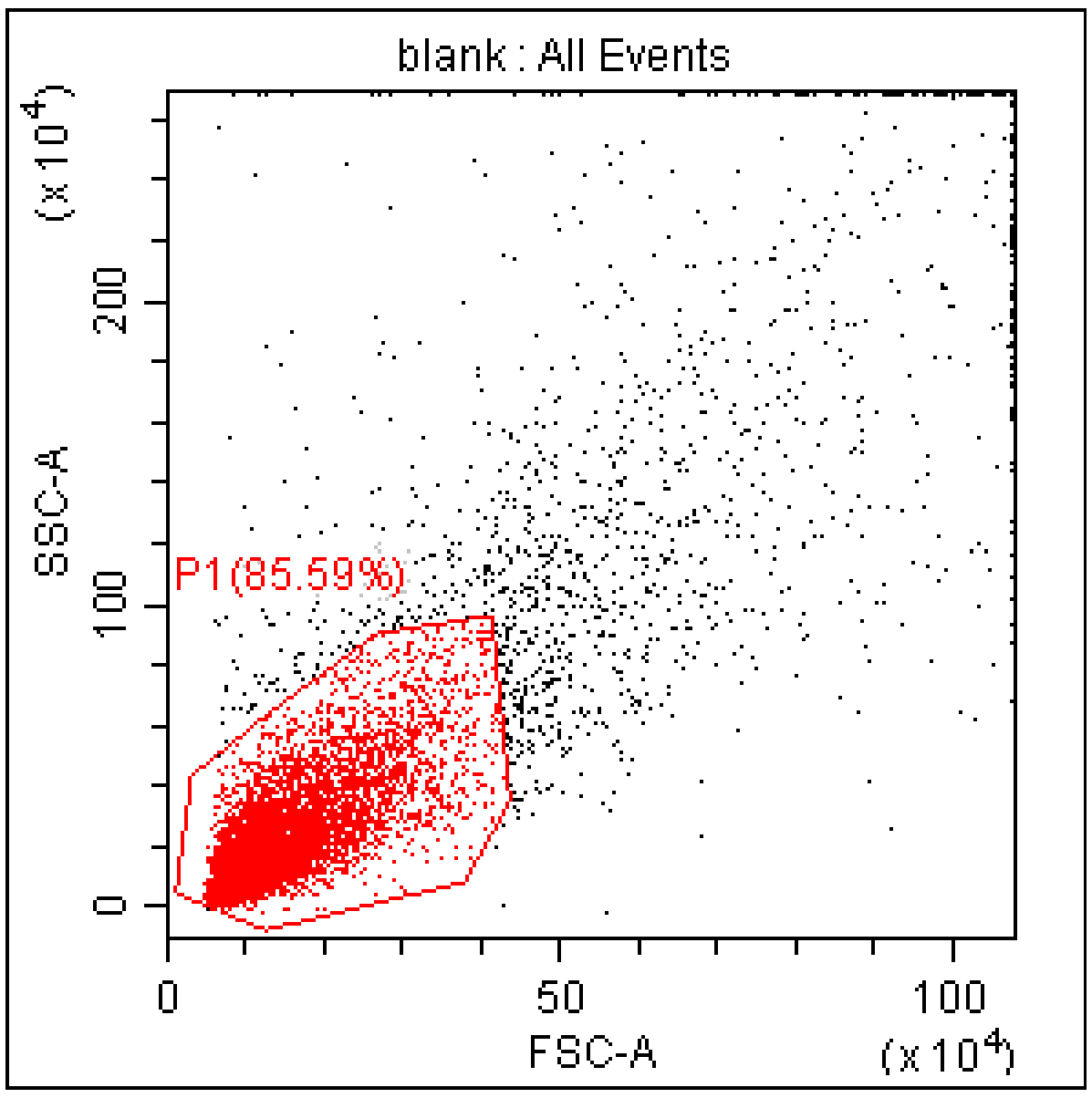

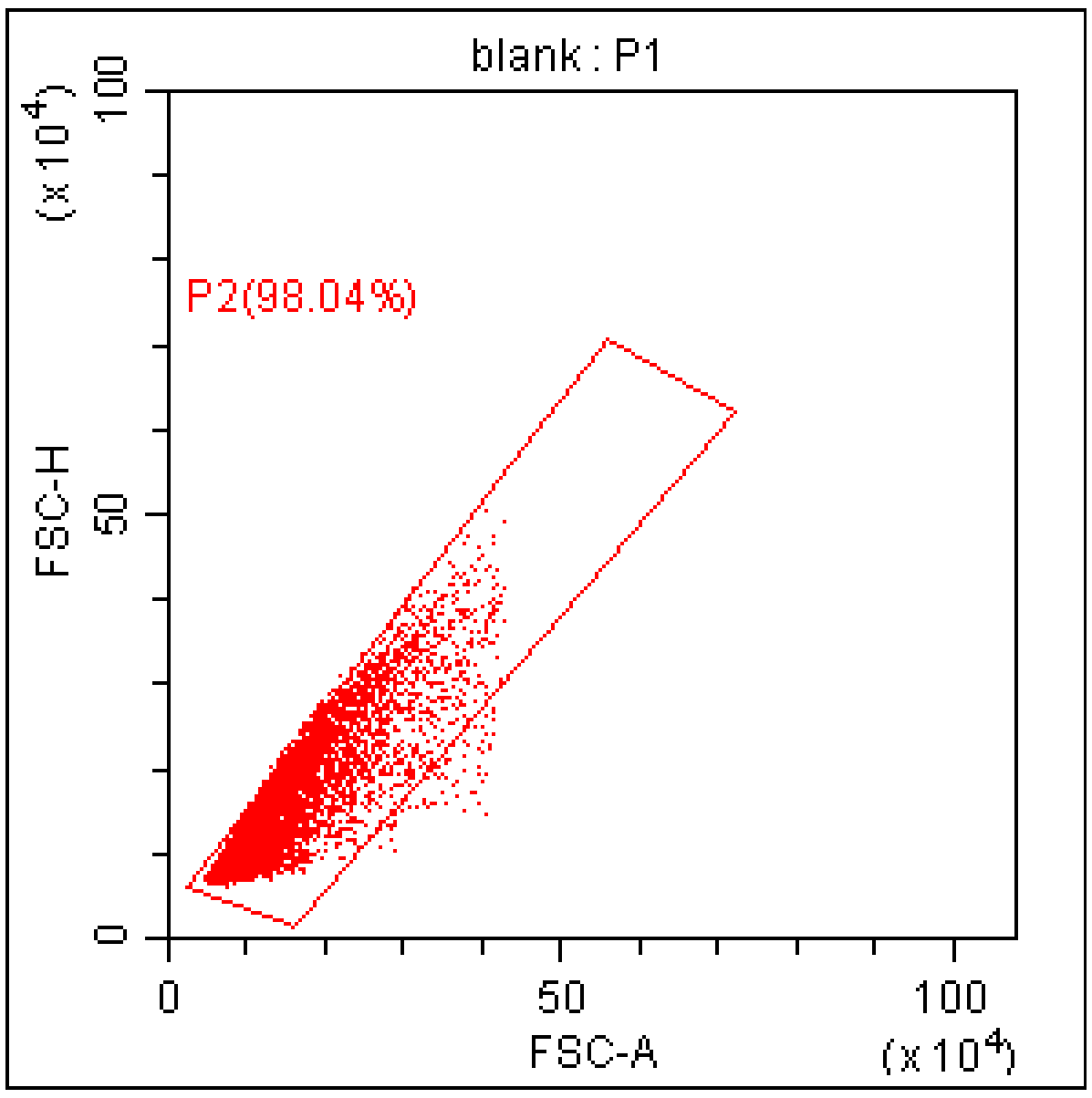

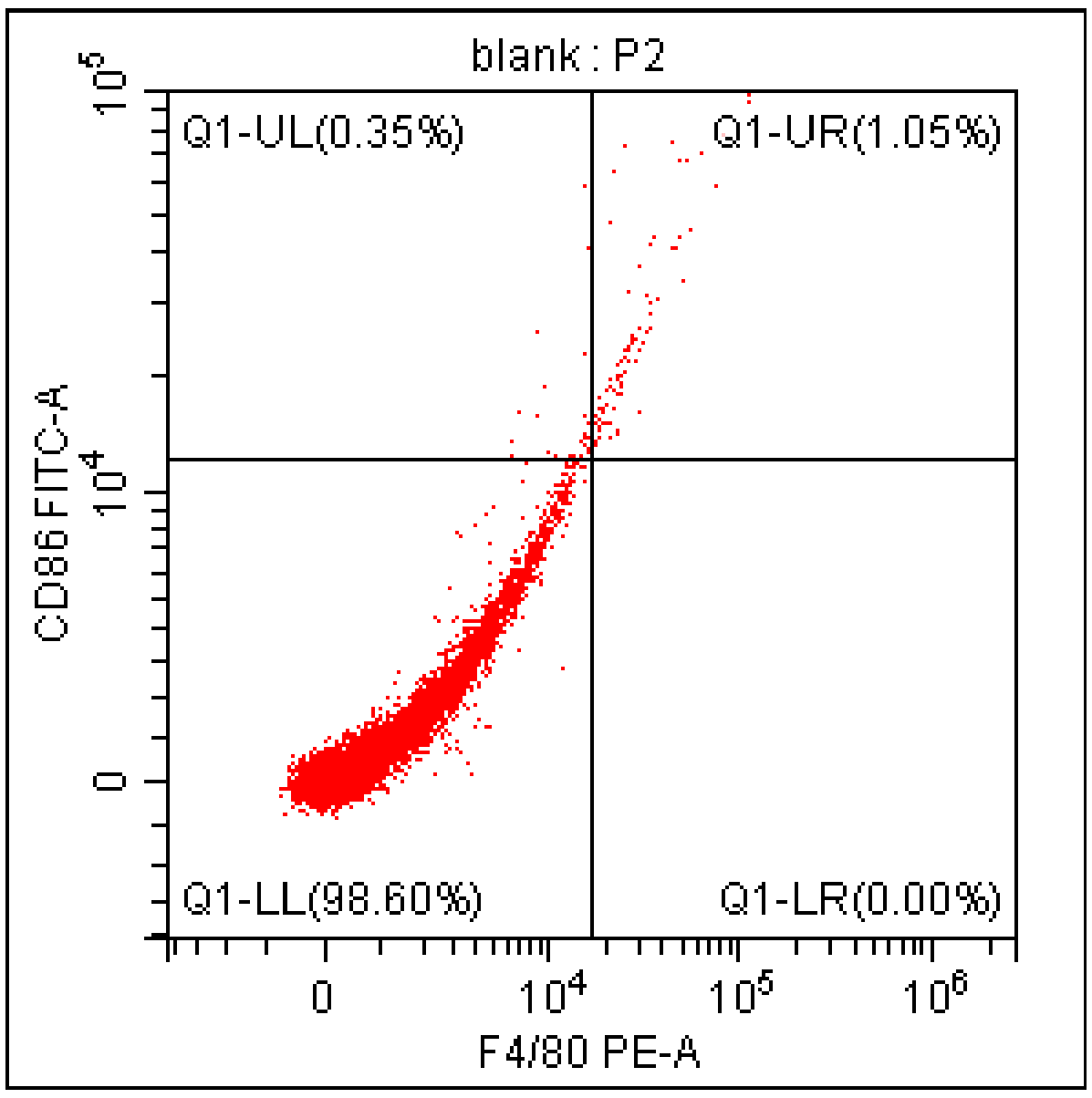

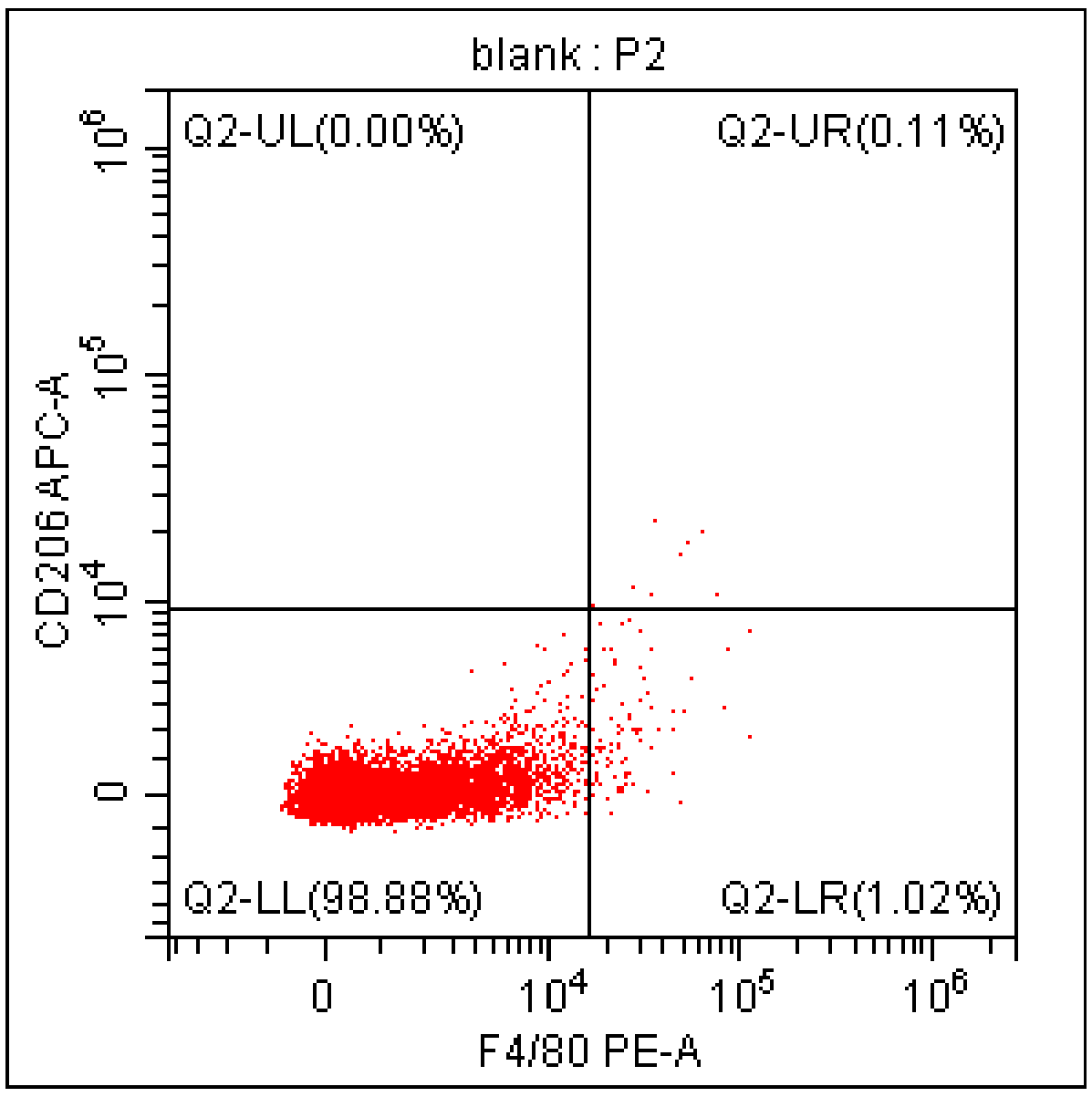

Figure 3A F4-80

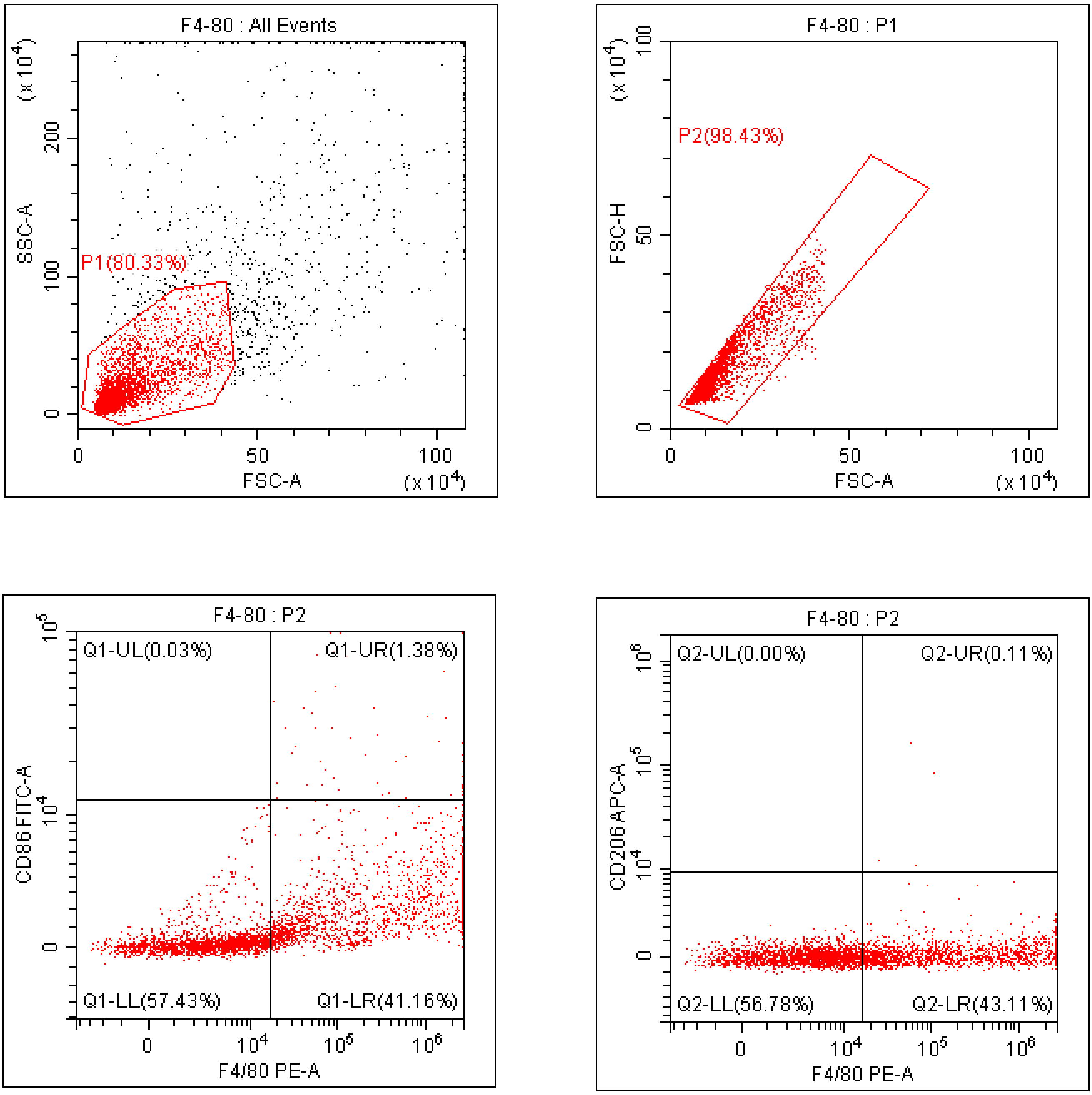

Figure 3A CD206

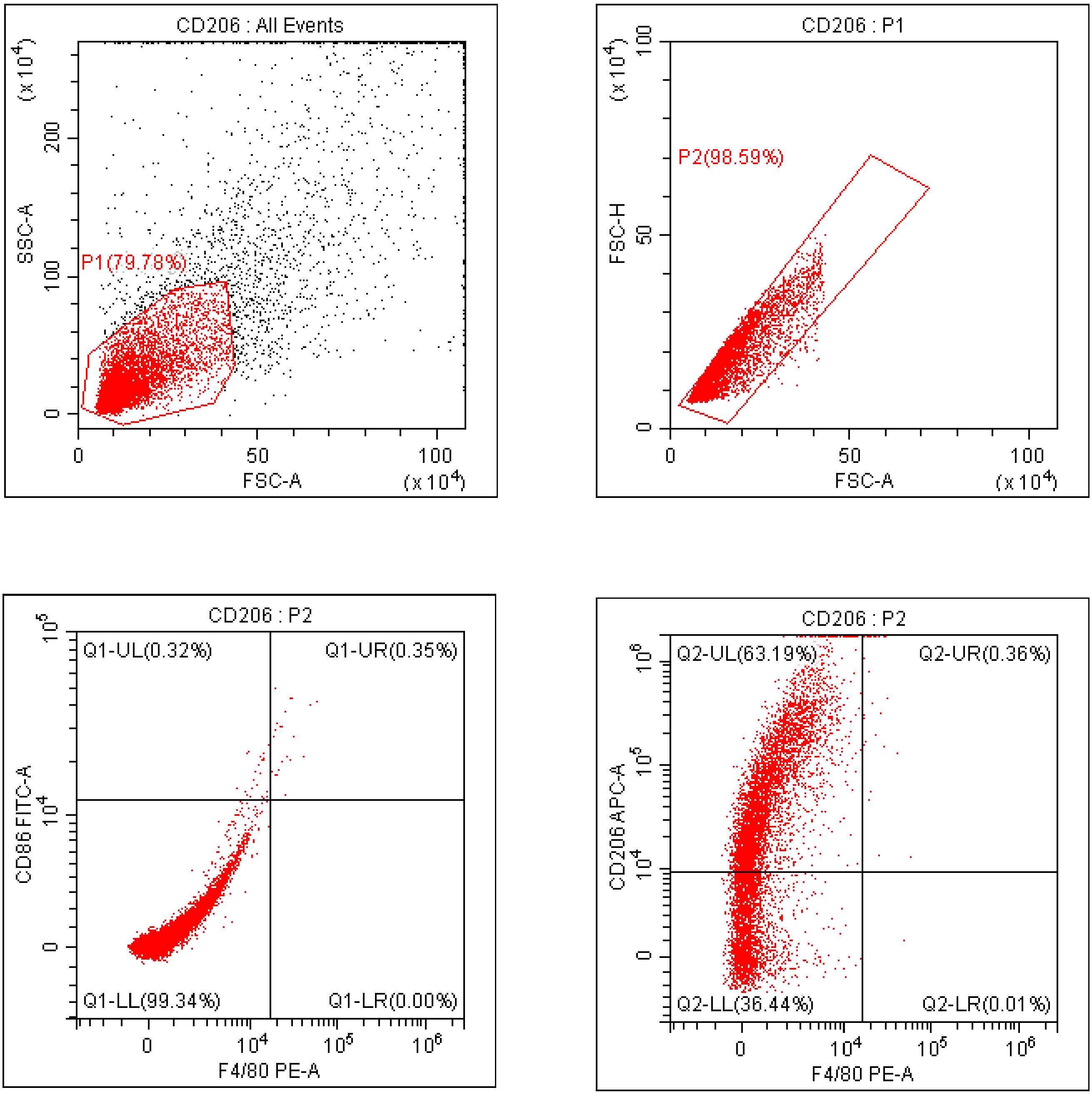

Figure 3A CD86

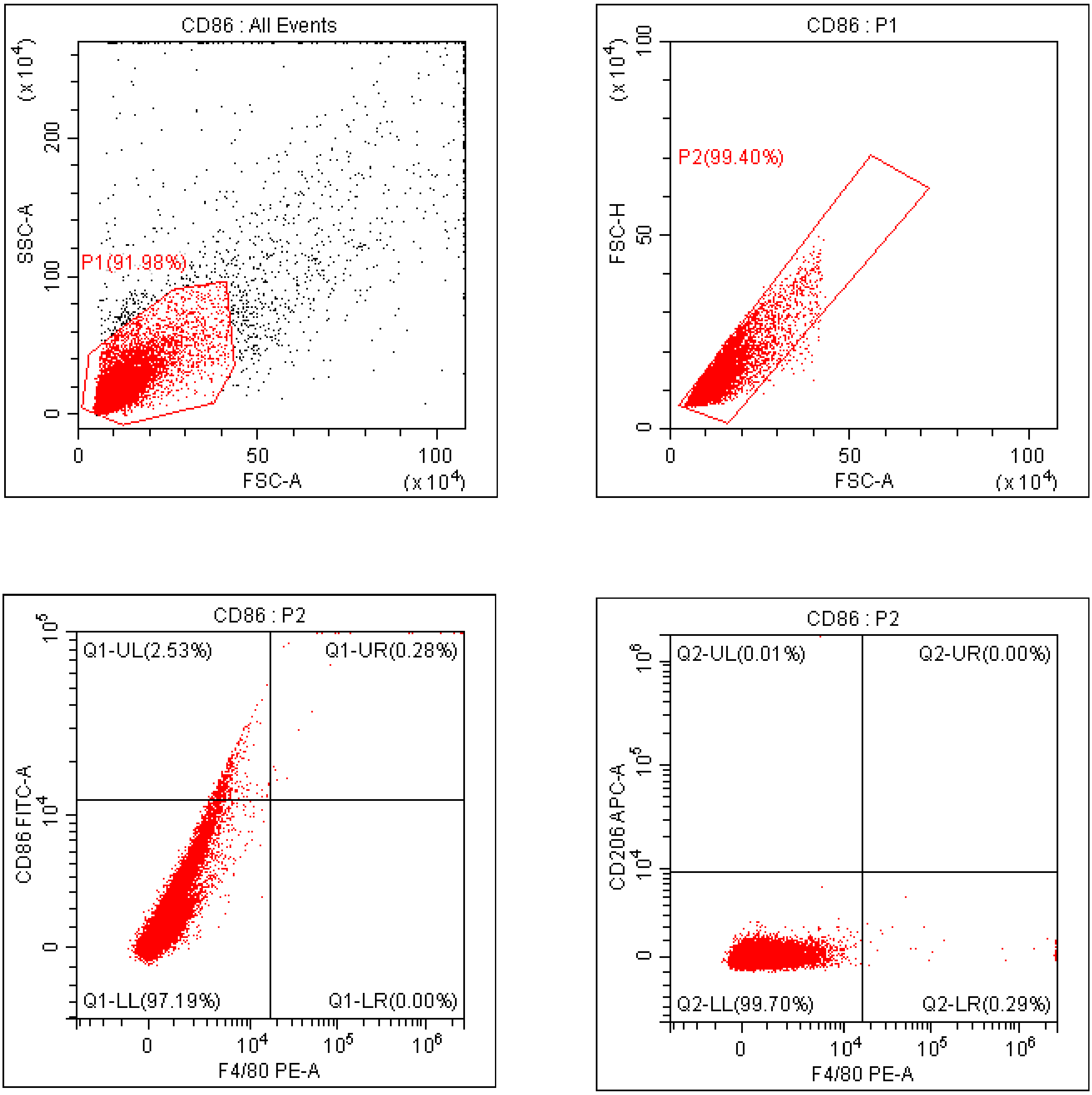

Figure 3A Positive drug-1

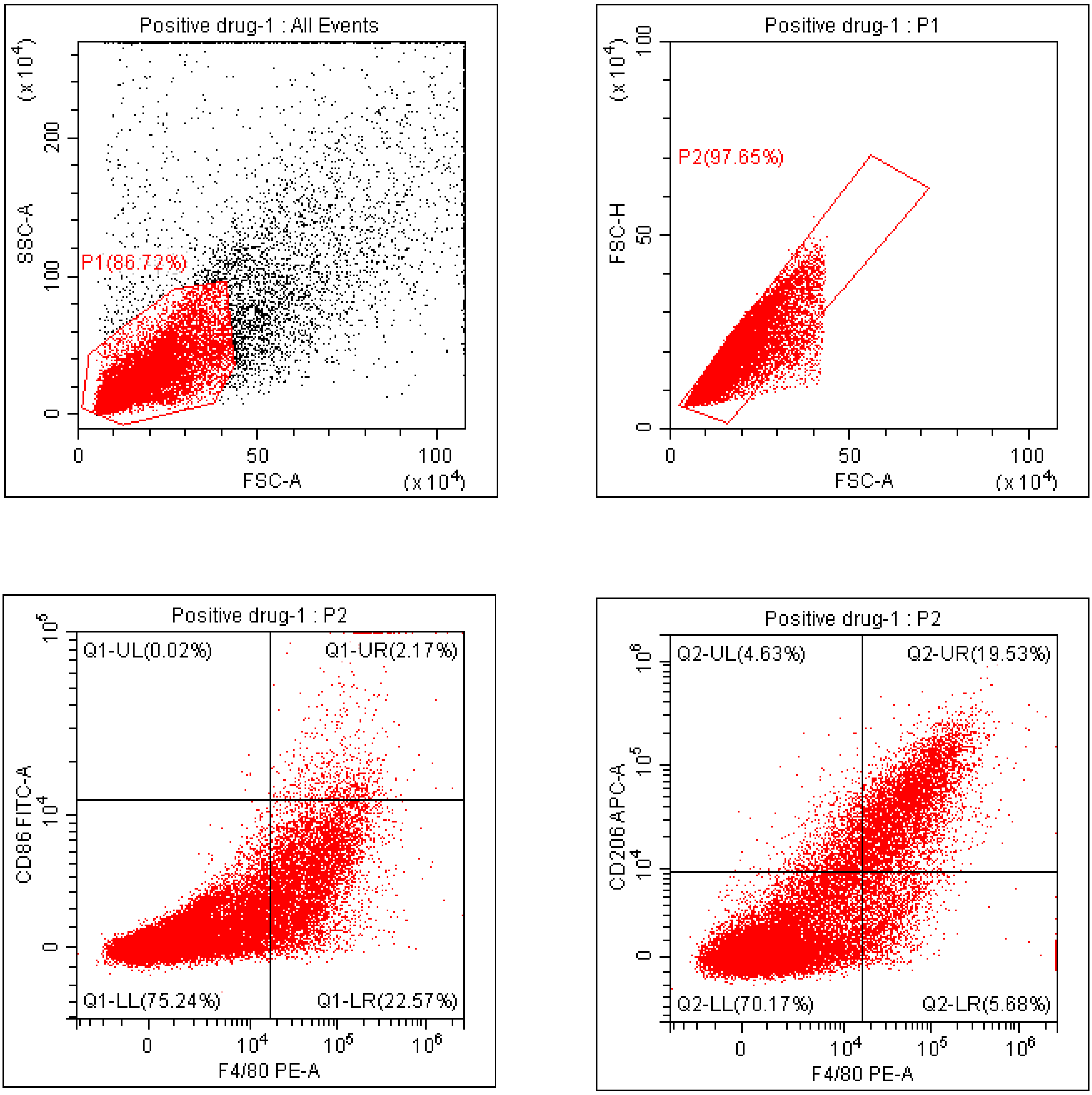

Figure 3A Positive drug-2

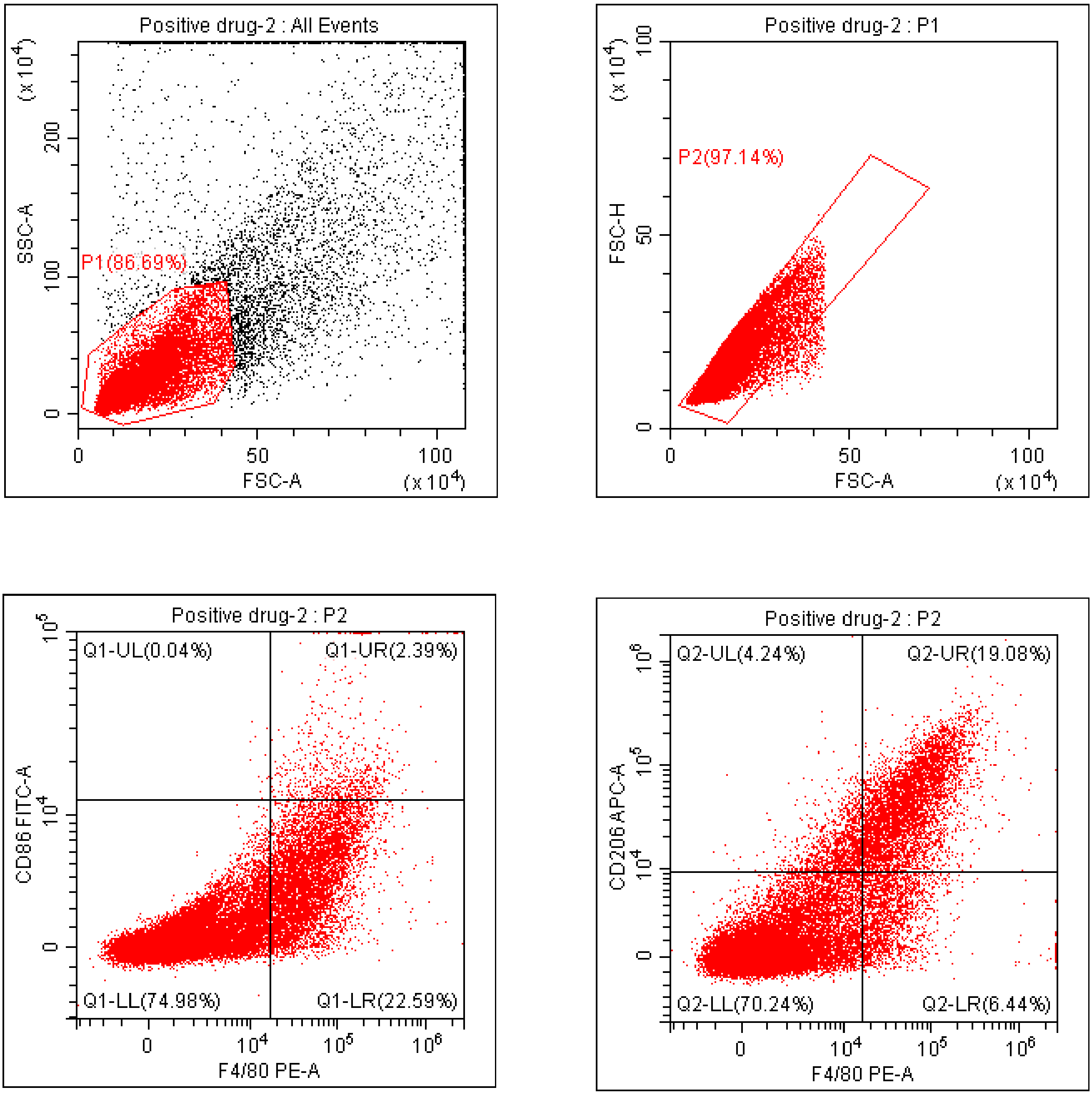

Figure 3A Positive drug-3

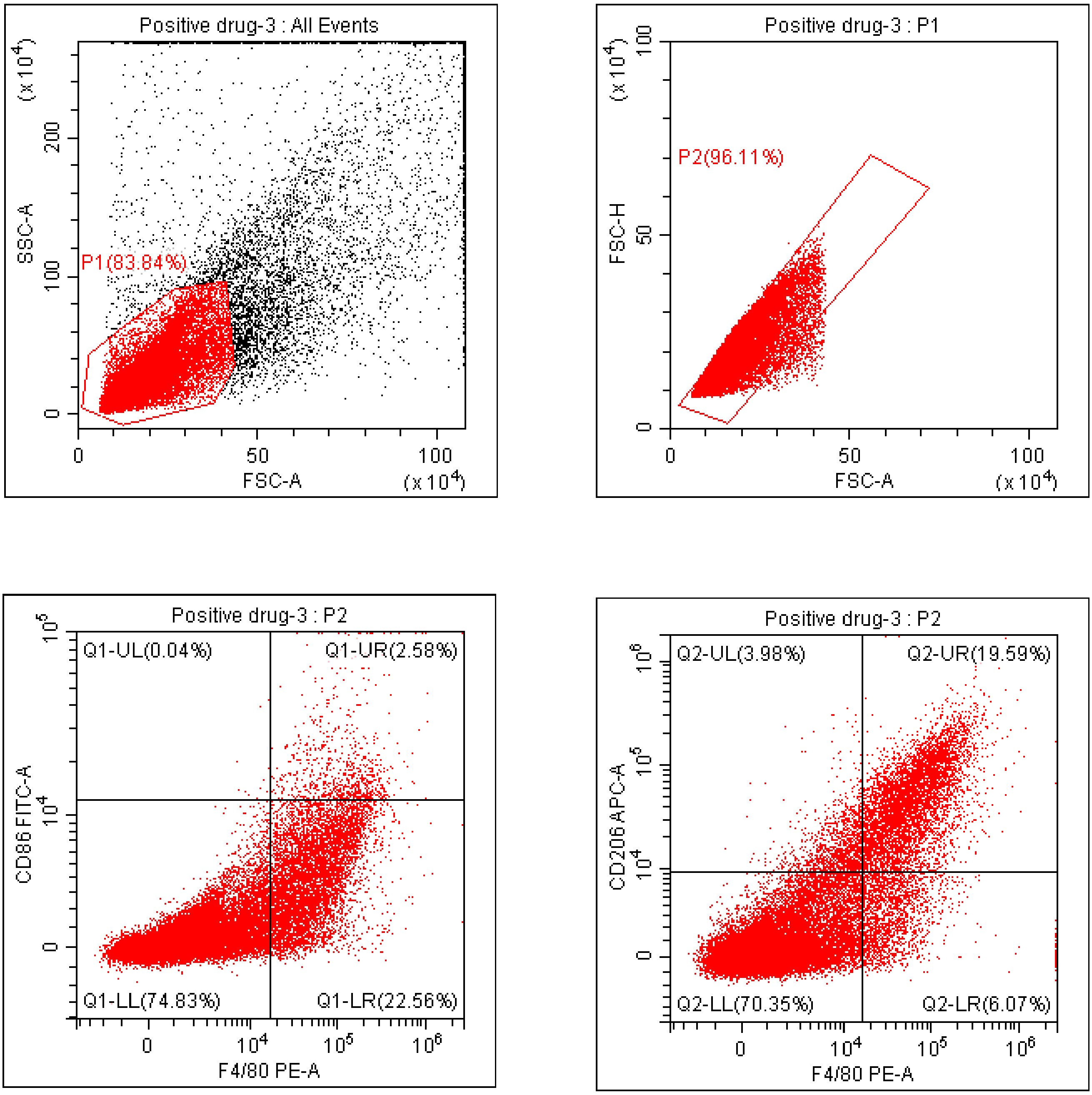

Figure 3A YQHXF-H-1

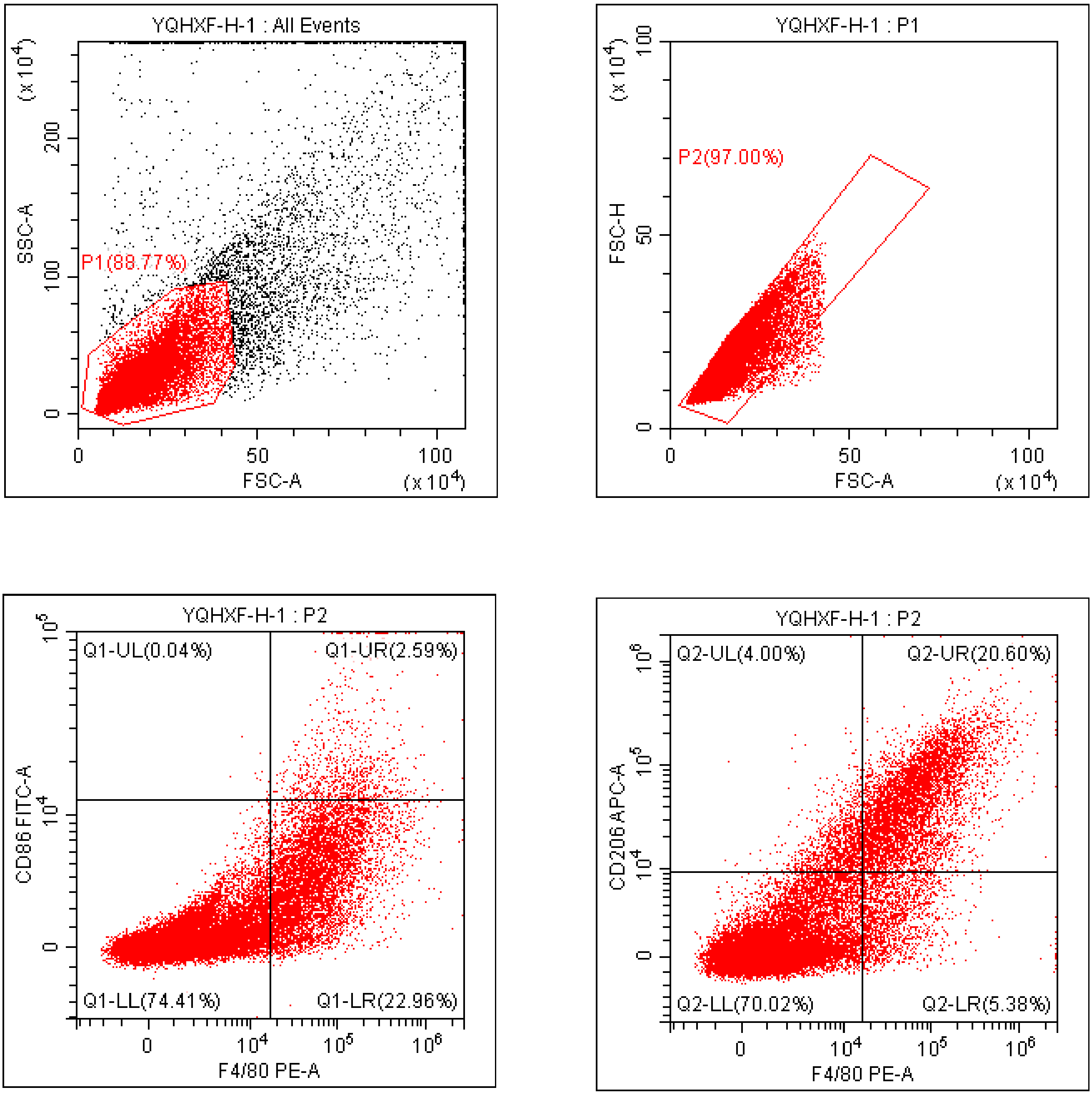

Figure 3A YQHXF-H-2

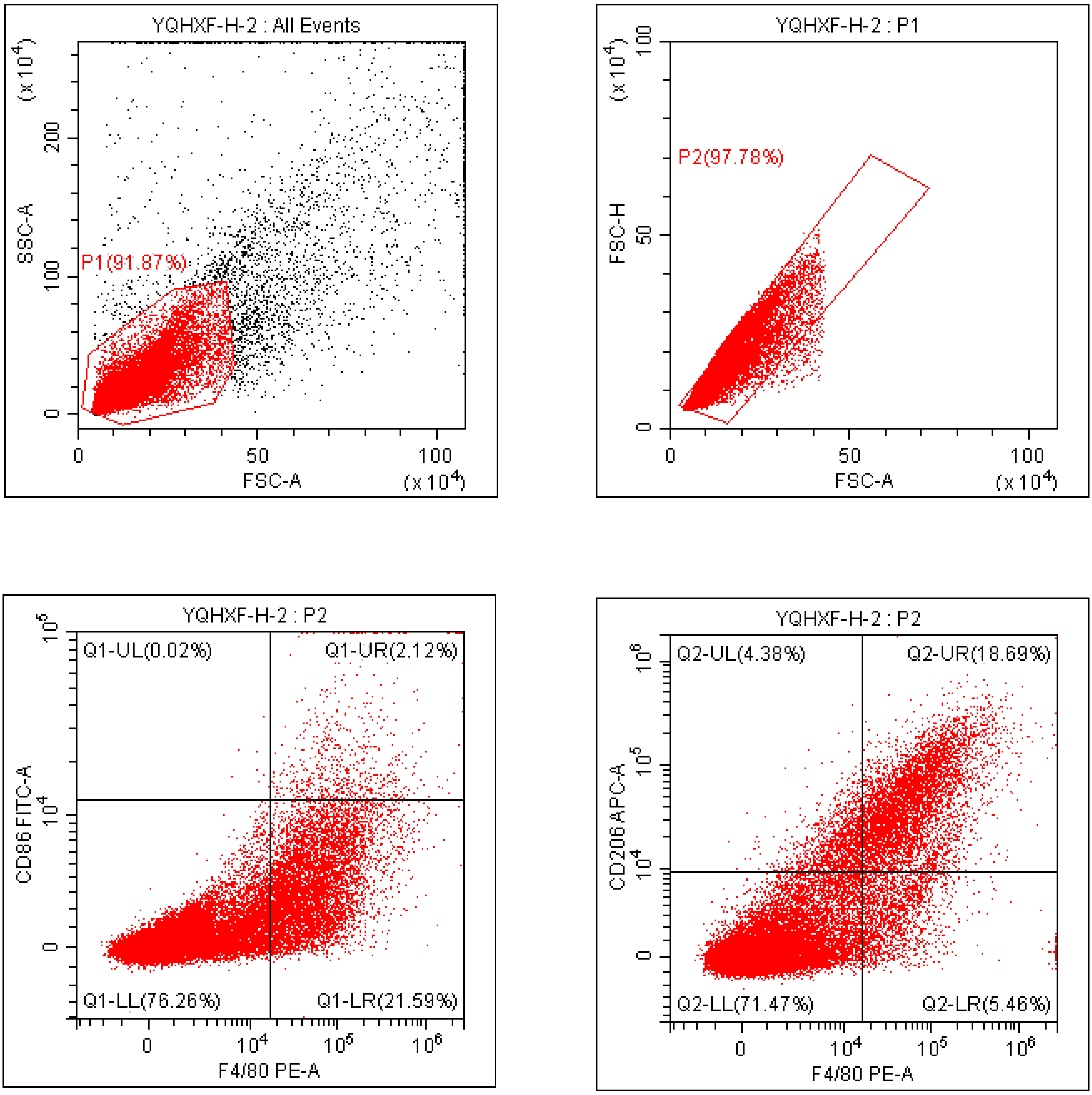

Figure 3A YQHXF-H-3

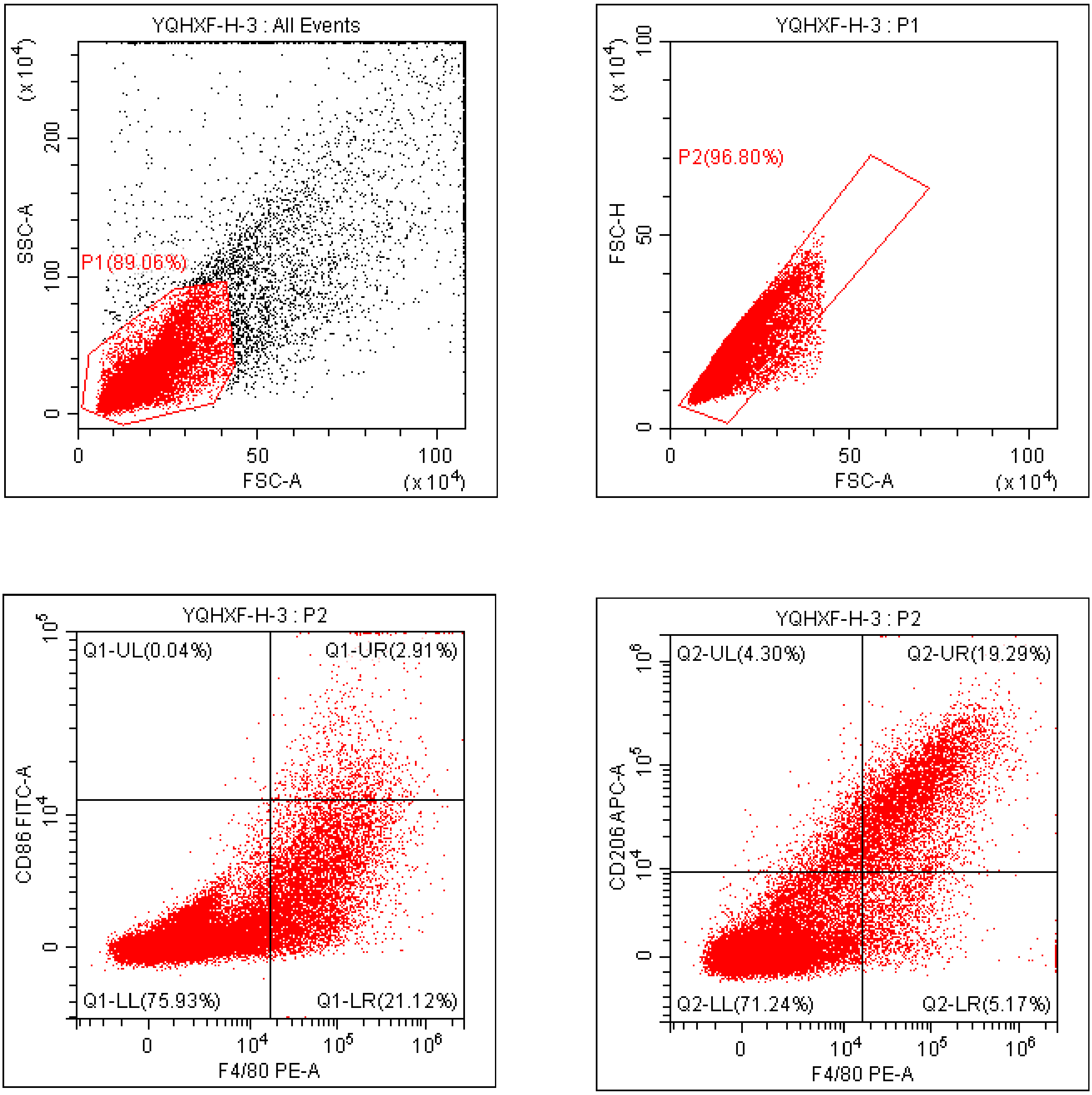

Figure 3A YQHXF-L-1

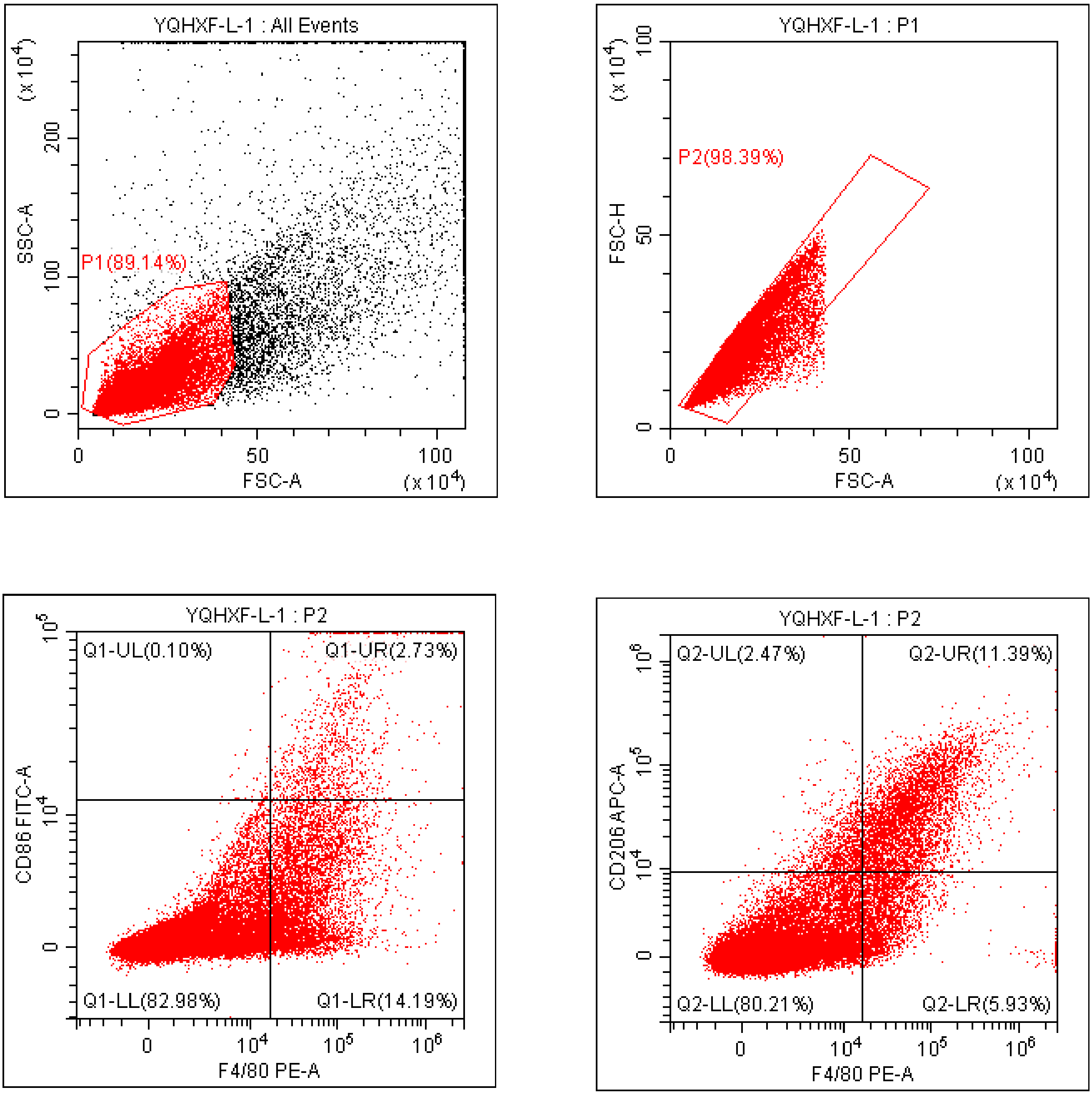

Figure 3A YQHXF-L-2

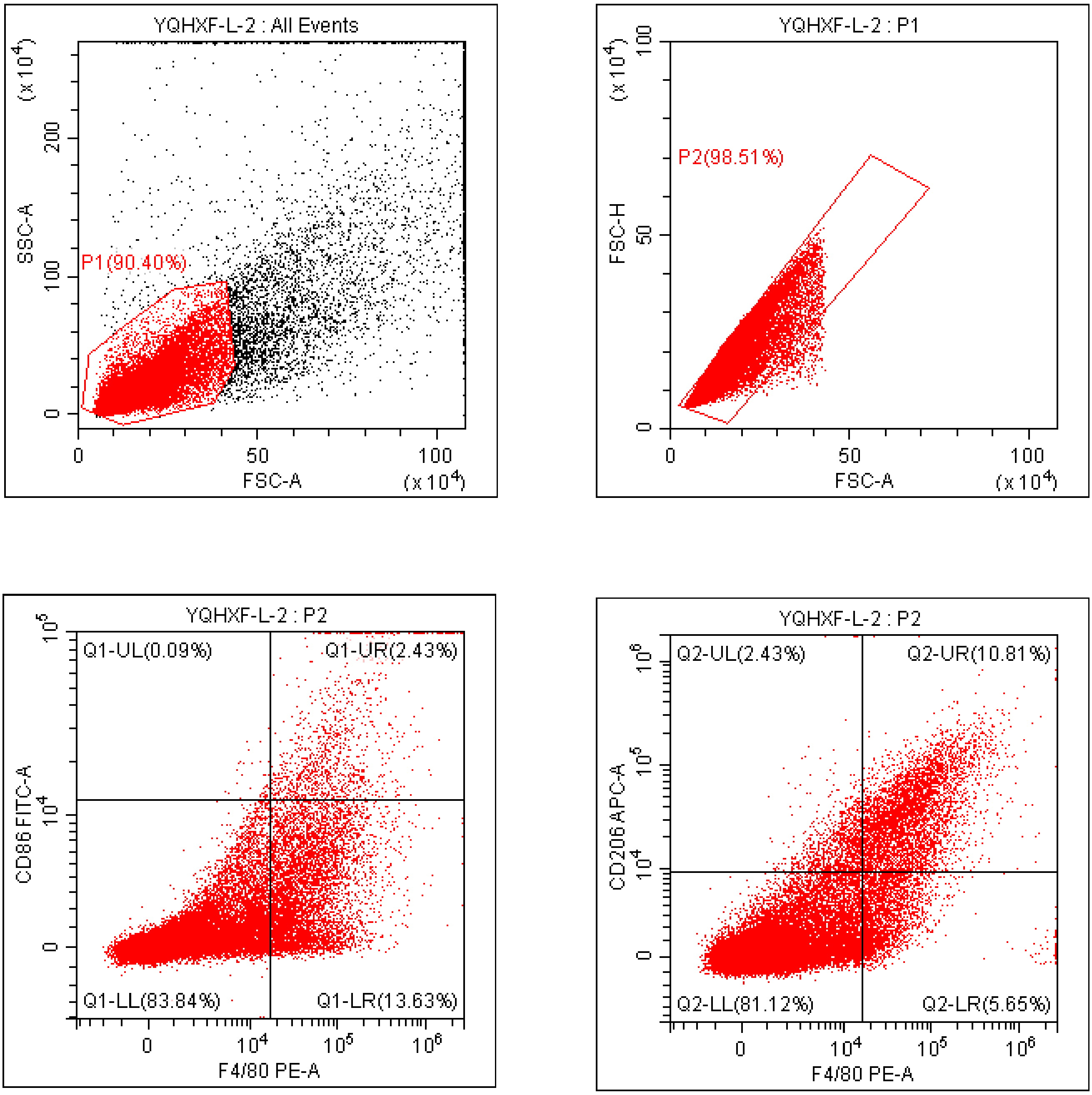

Figure 3A YQHXF-L-3

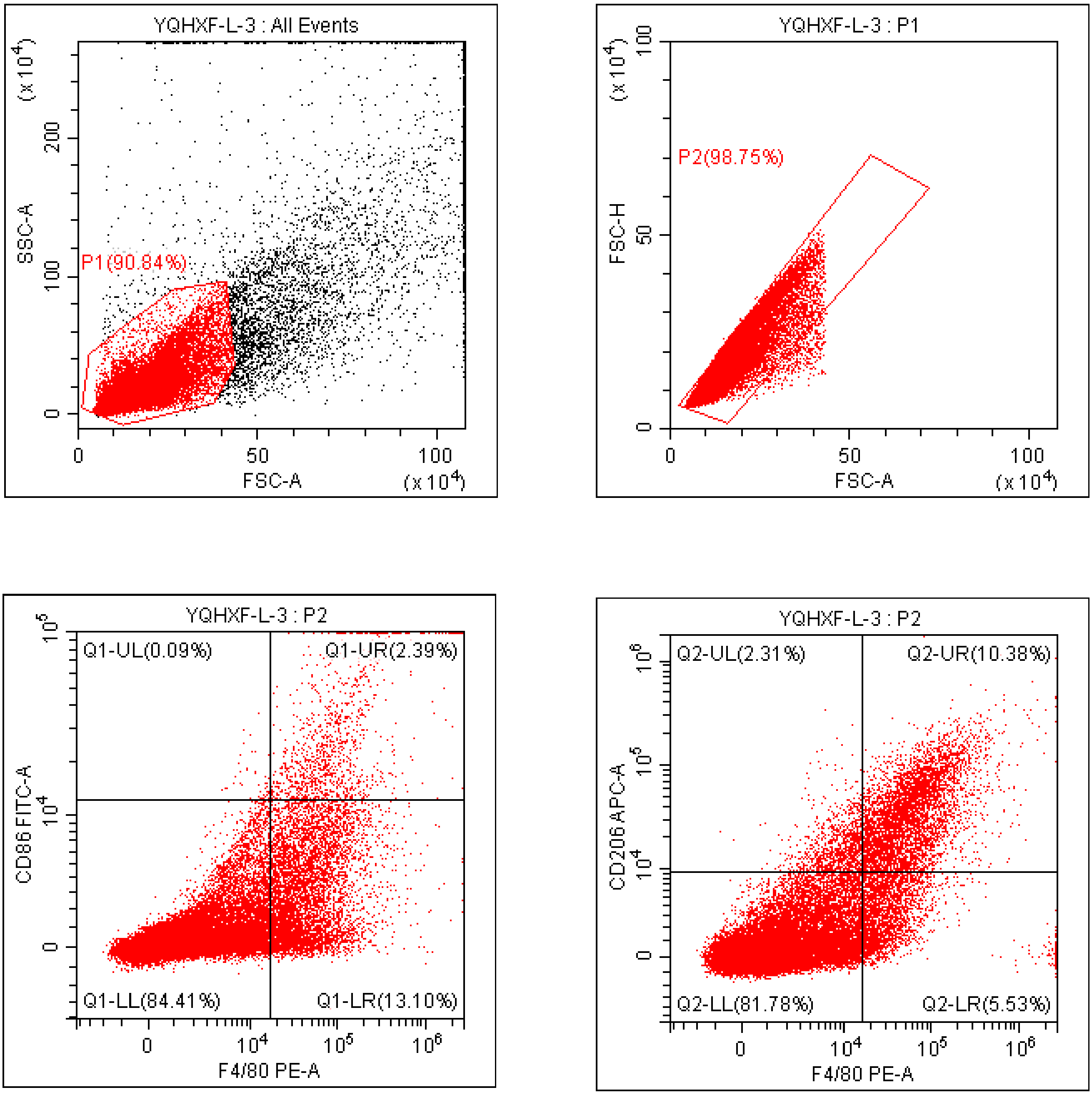

Figure 3A Model-1

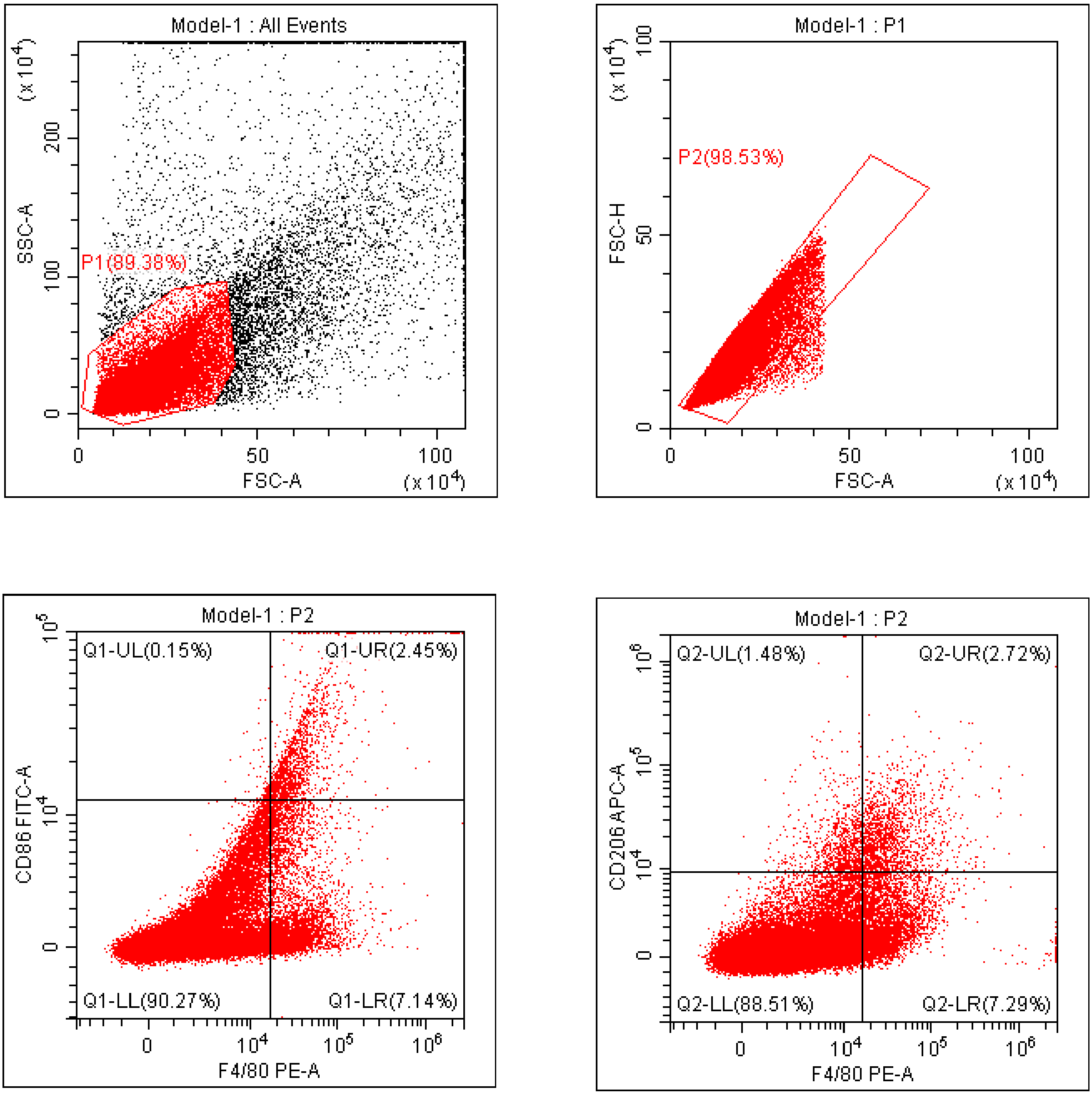

Figure 3A Model-2

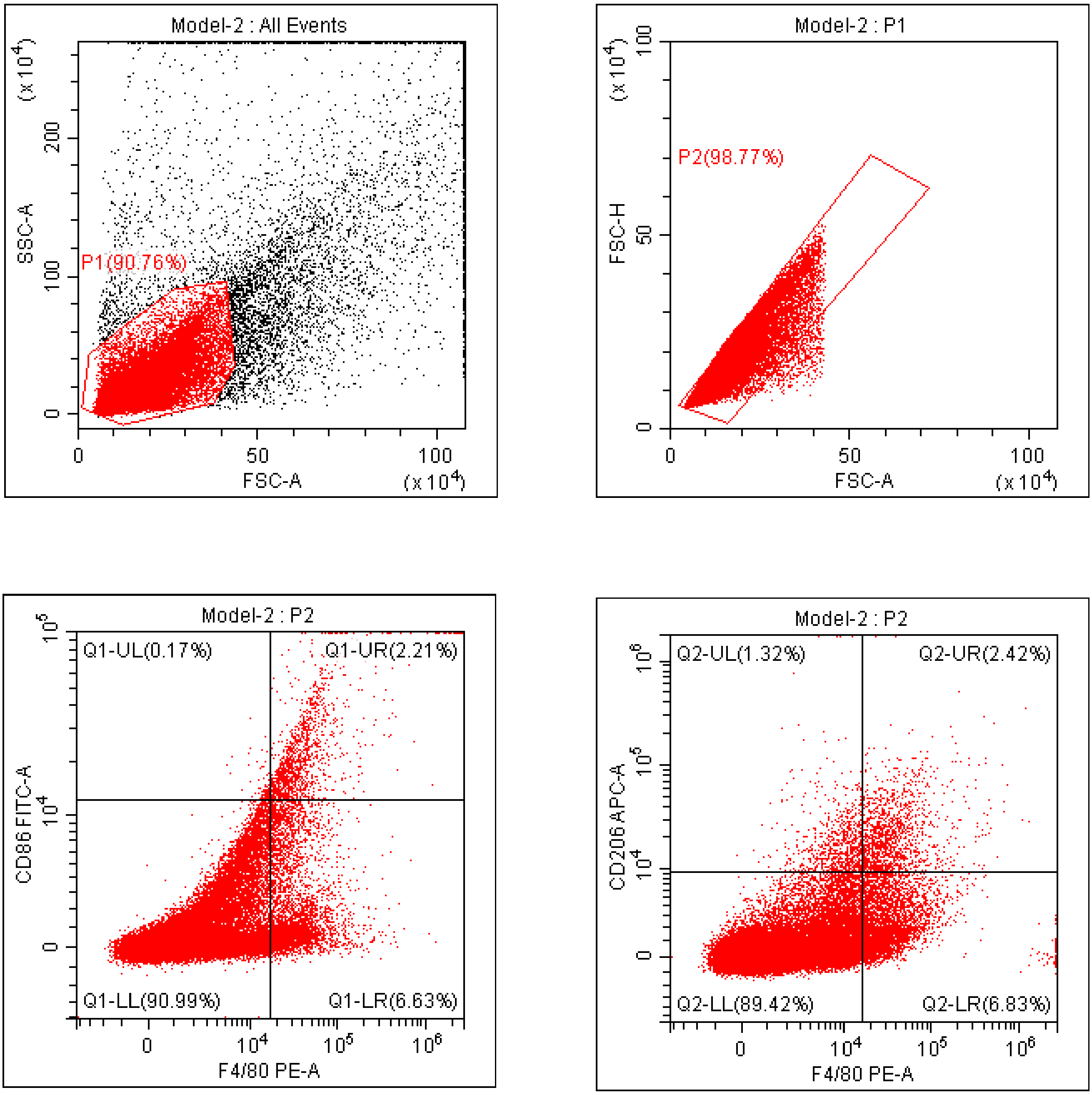

Figure 3A Model-3

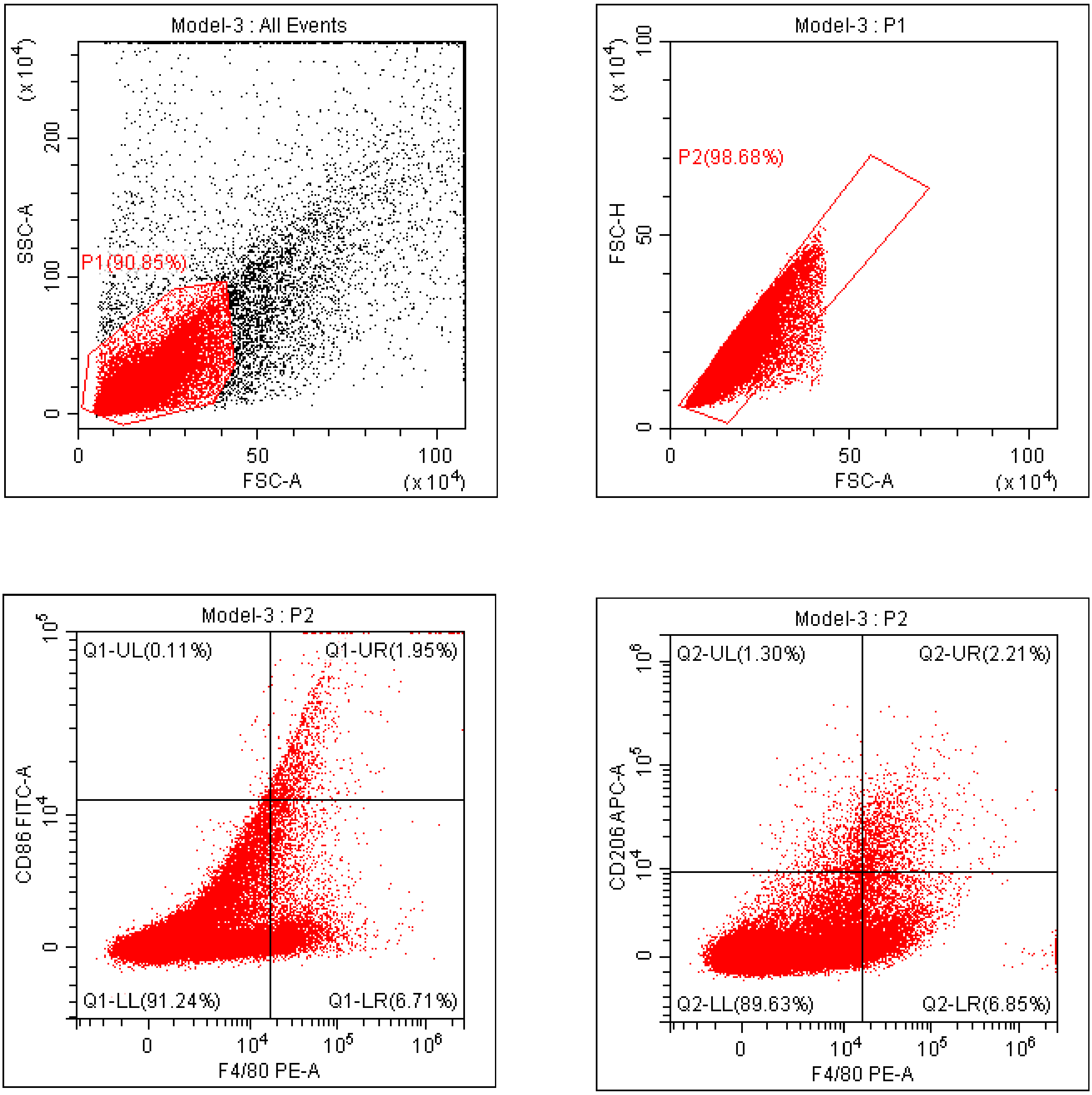

Figure 3A YQHXF-M-1

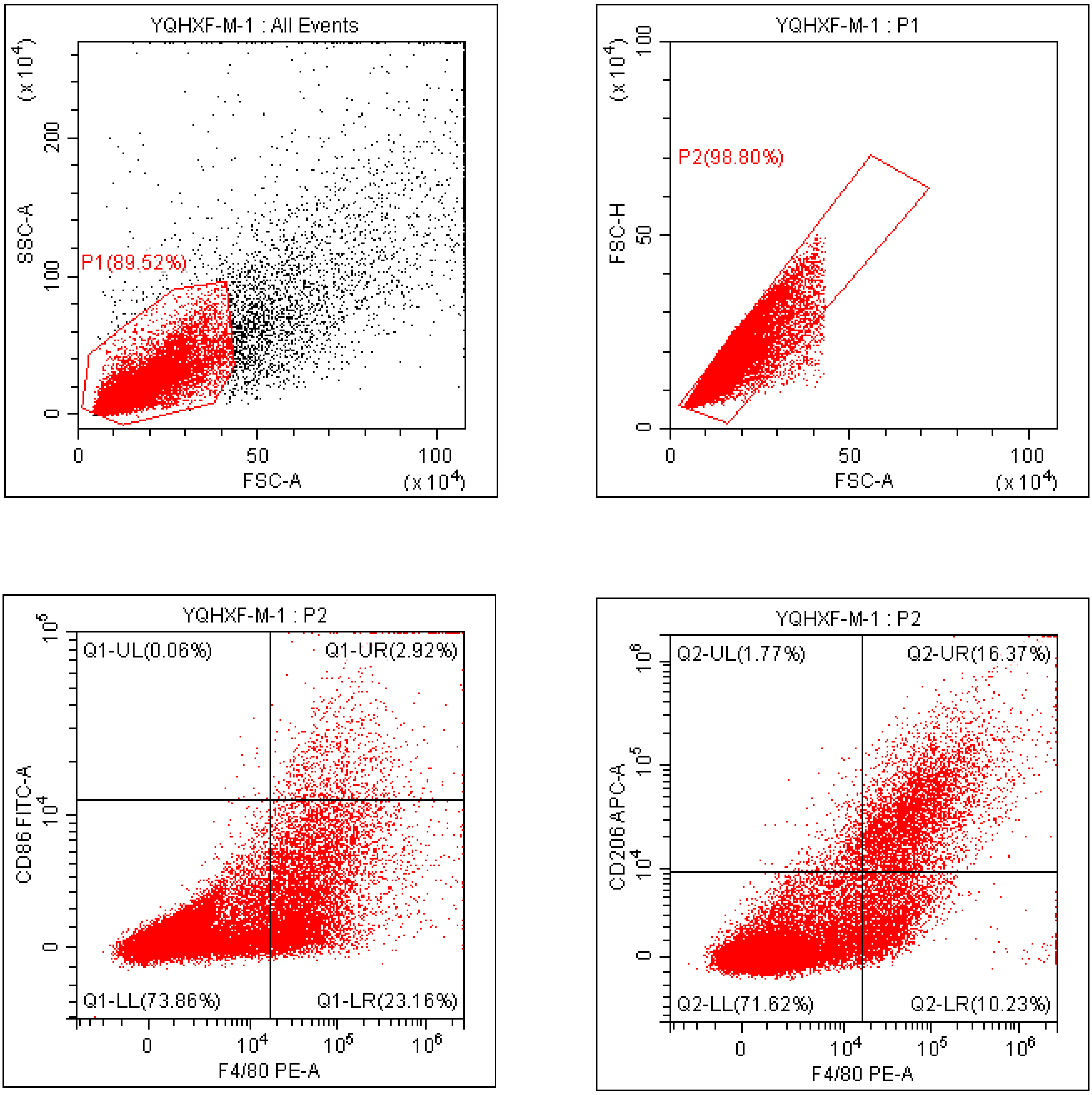

Figure 3A YQHXF-M-2

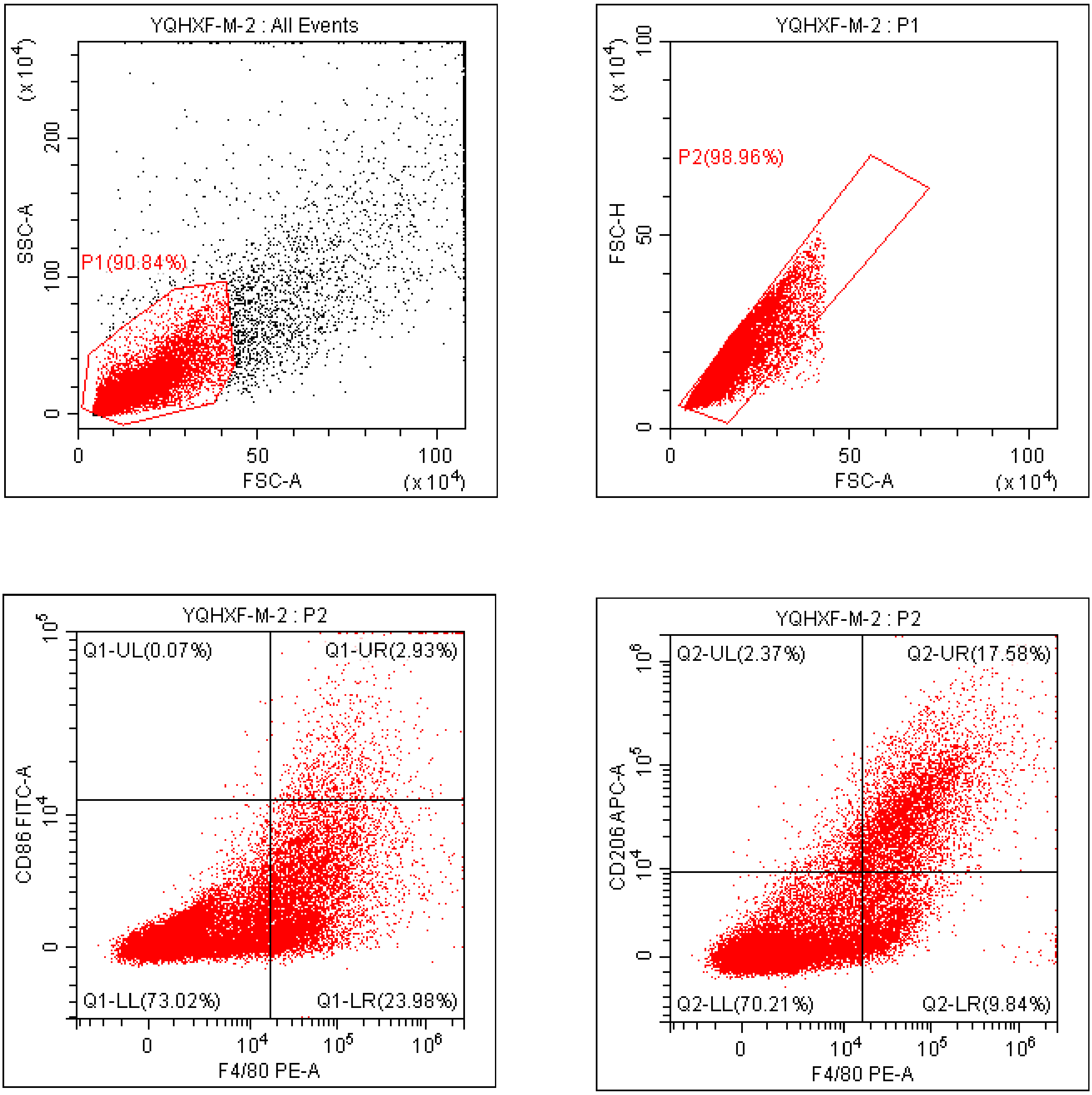

Figure 3A YQHXF-M-3

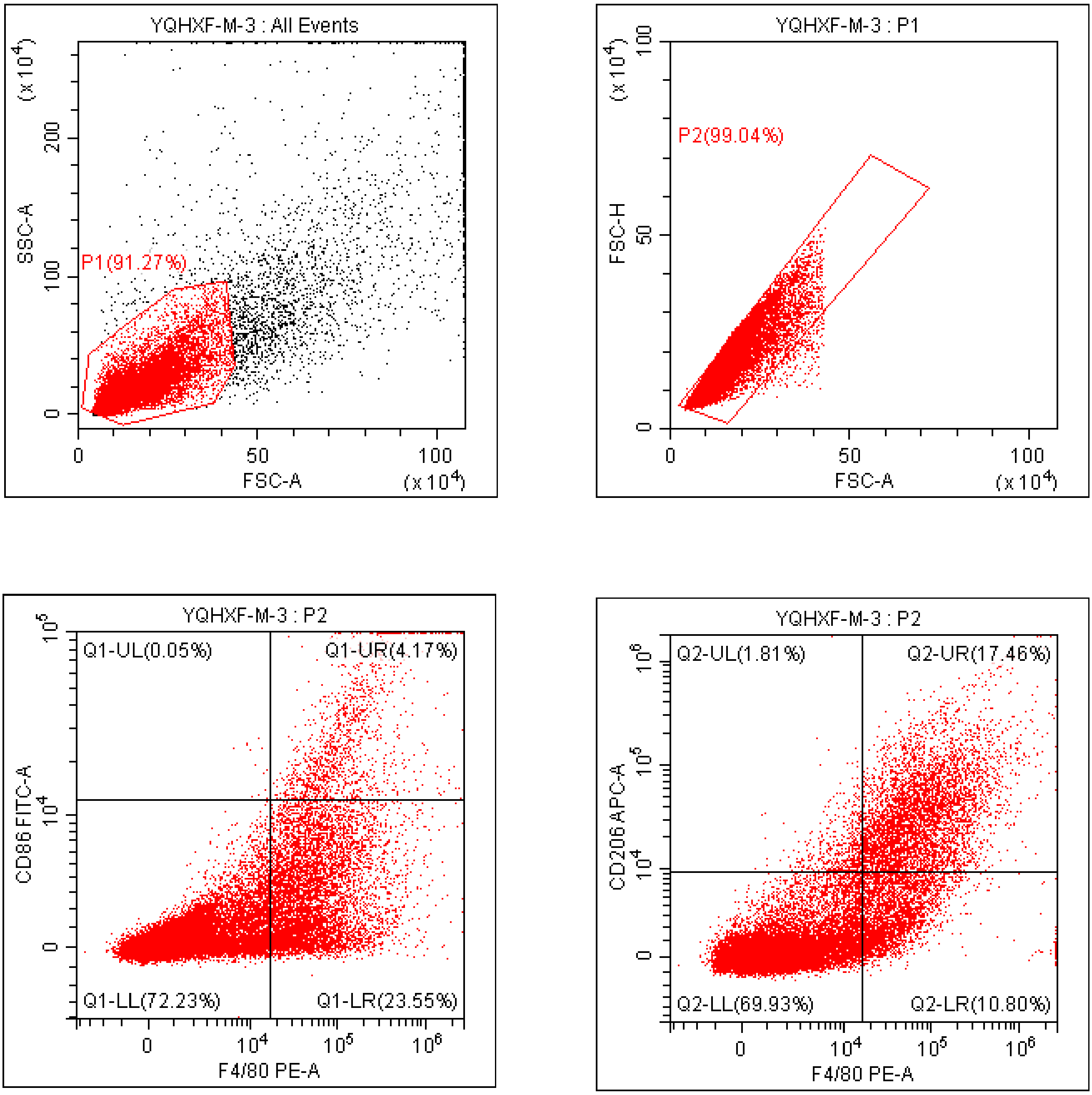

Figure 3A Control-1

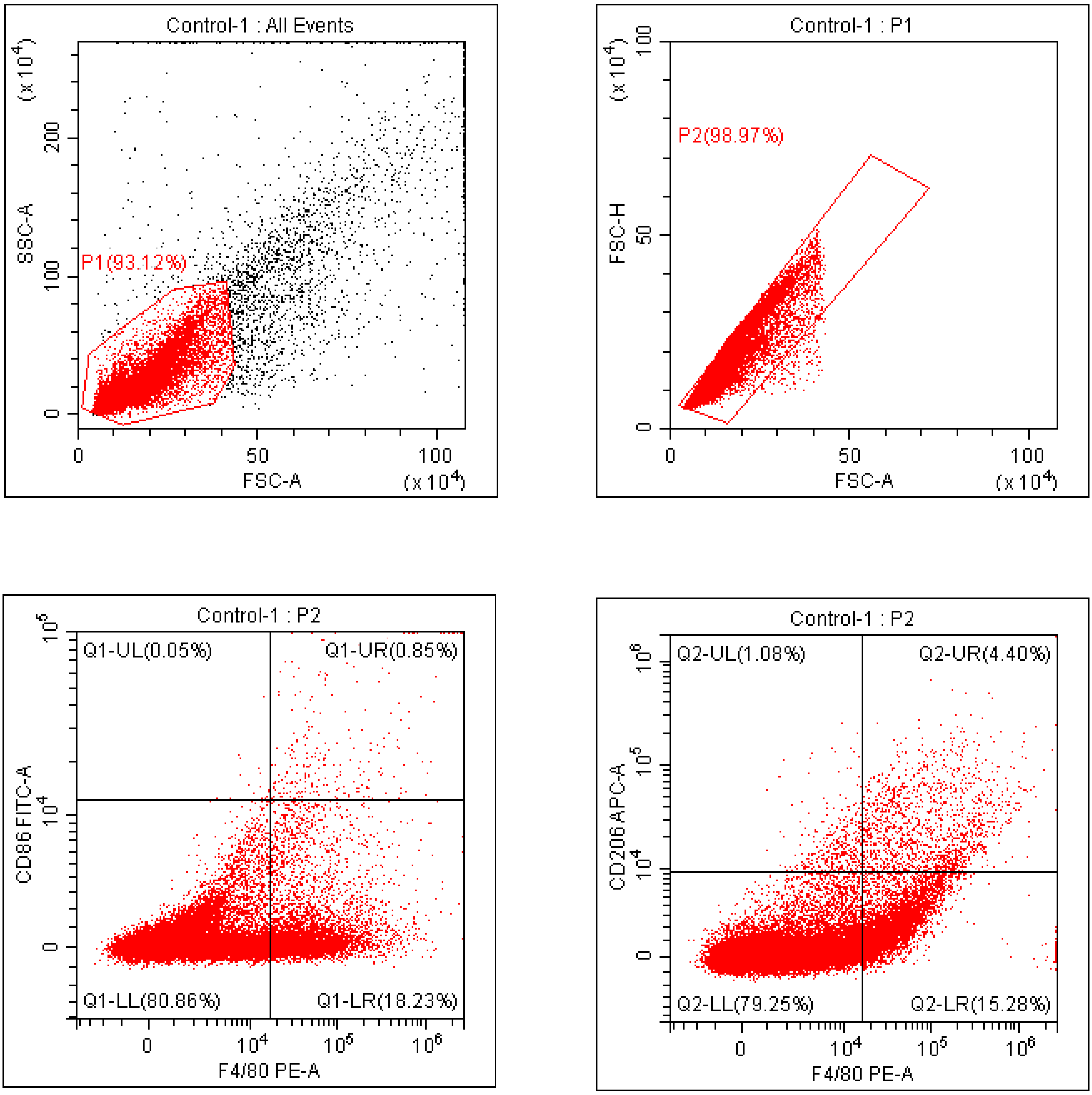

Figure 3A Control-2

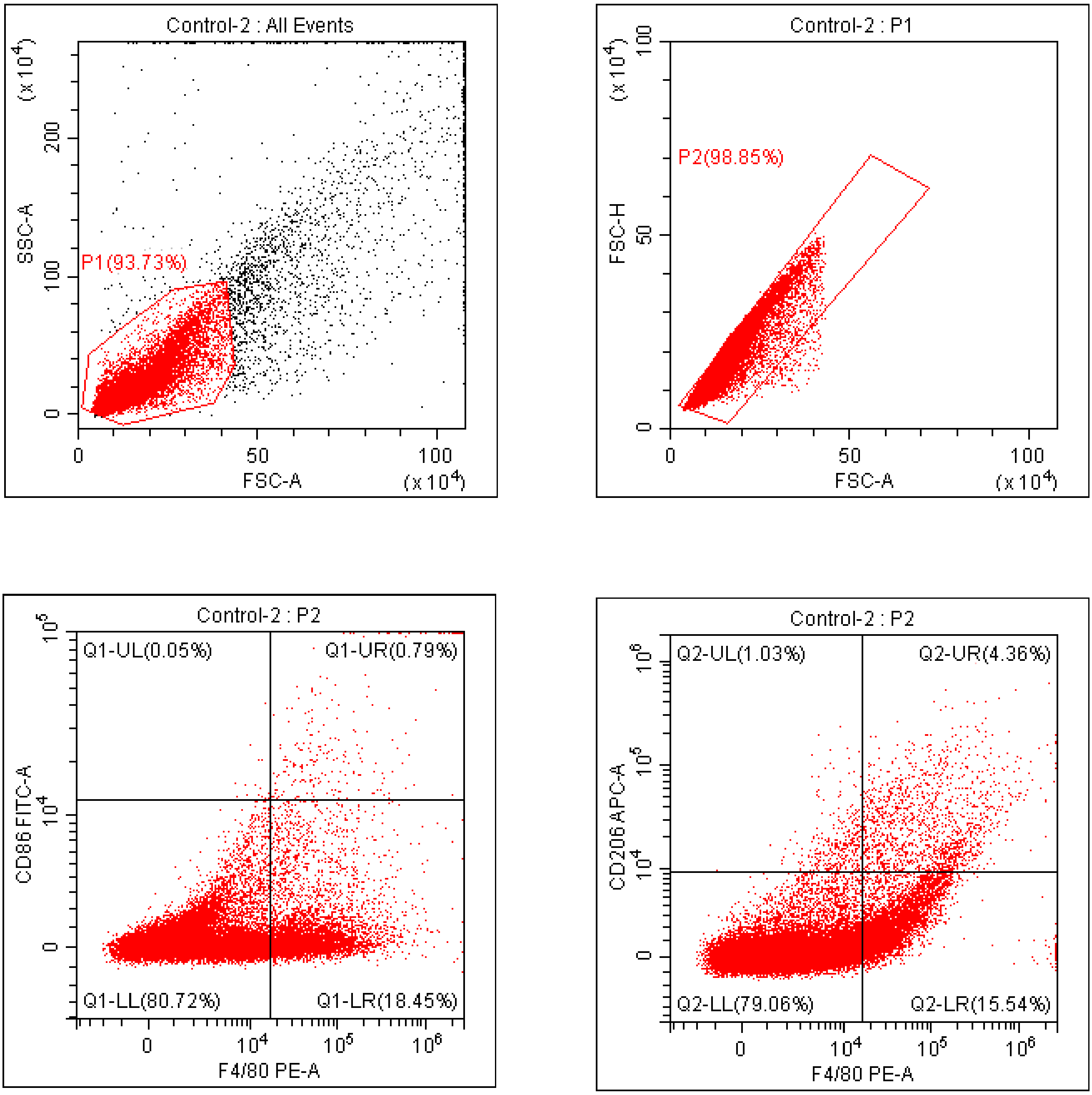

Figure 3A Control-3

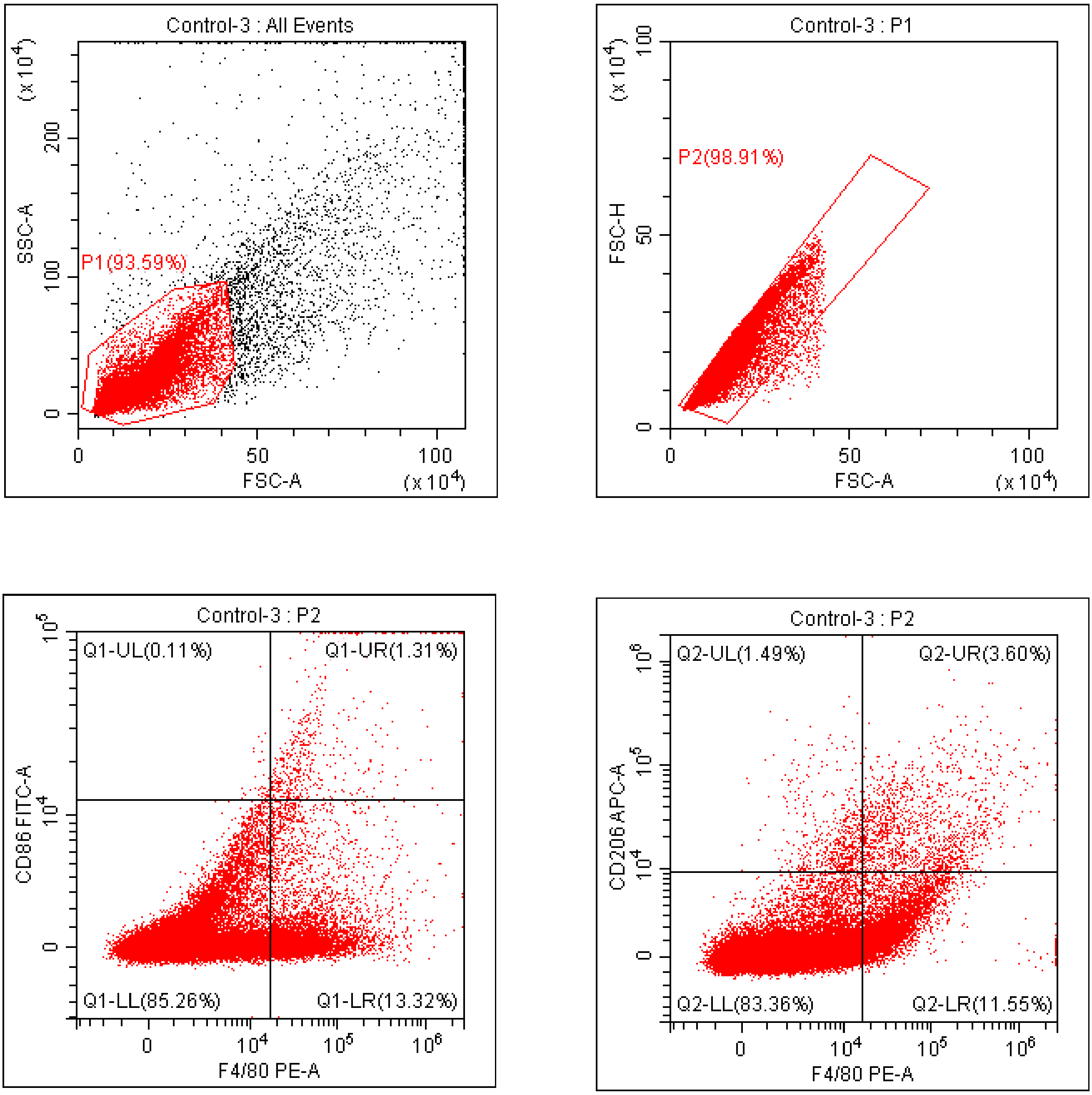

(2) The raw data of flow cytometry in Figure S5

Figure S5 BLANK CD80/CD163

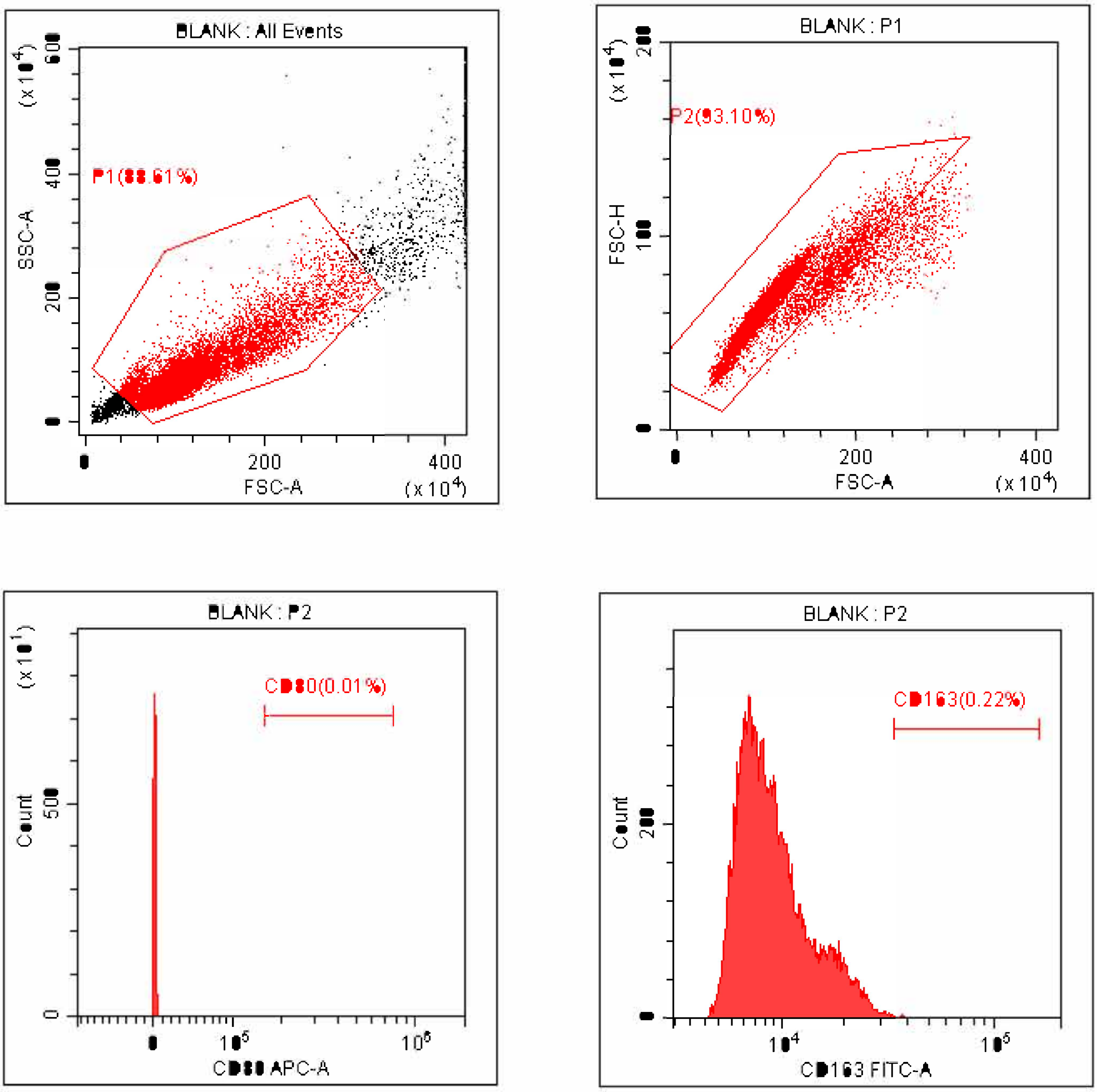

Figure S5 CD80

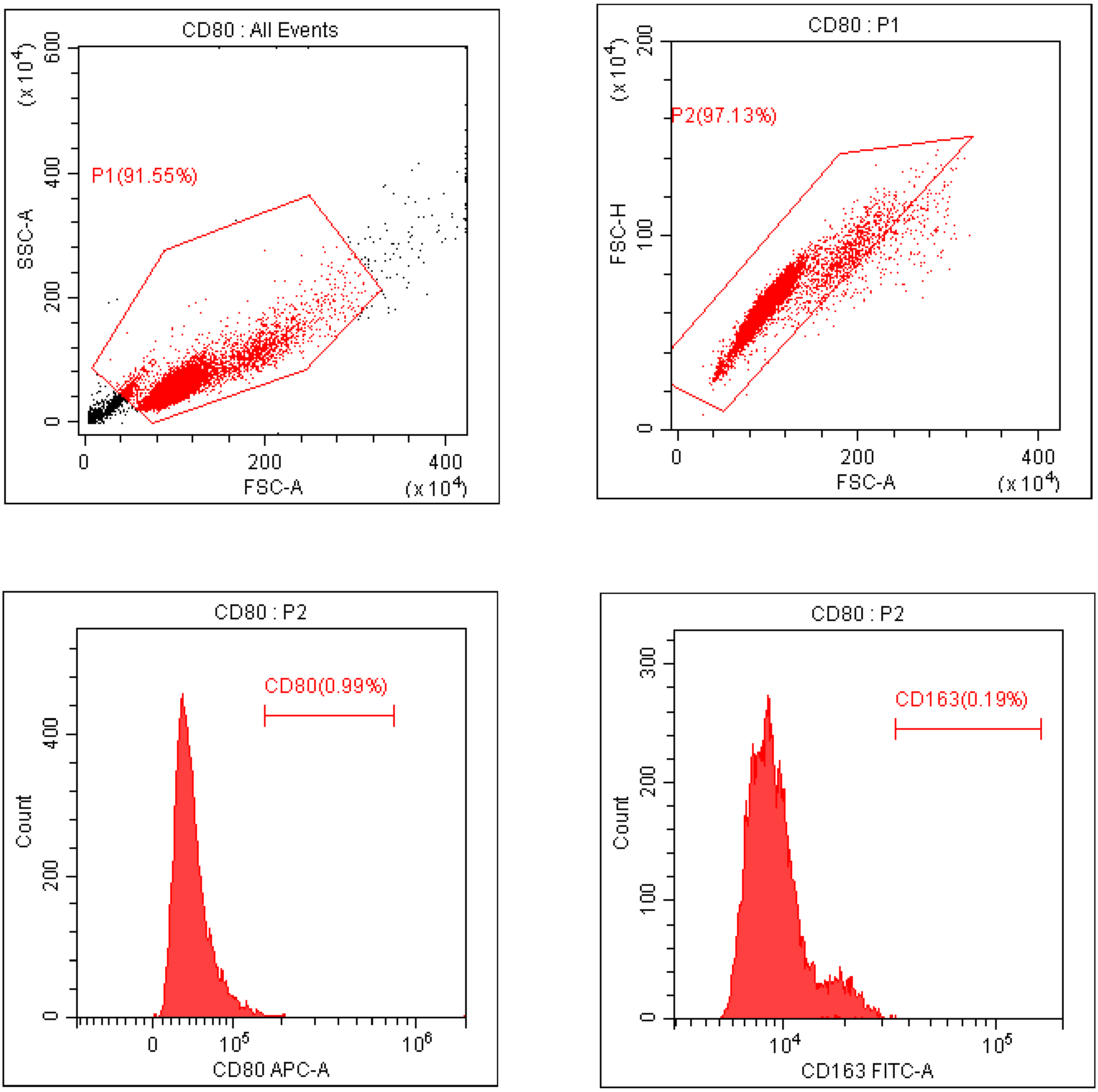

Figure S5 CD163

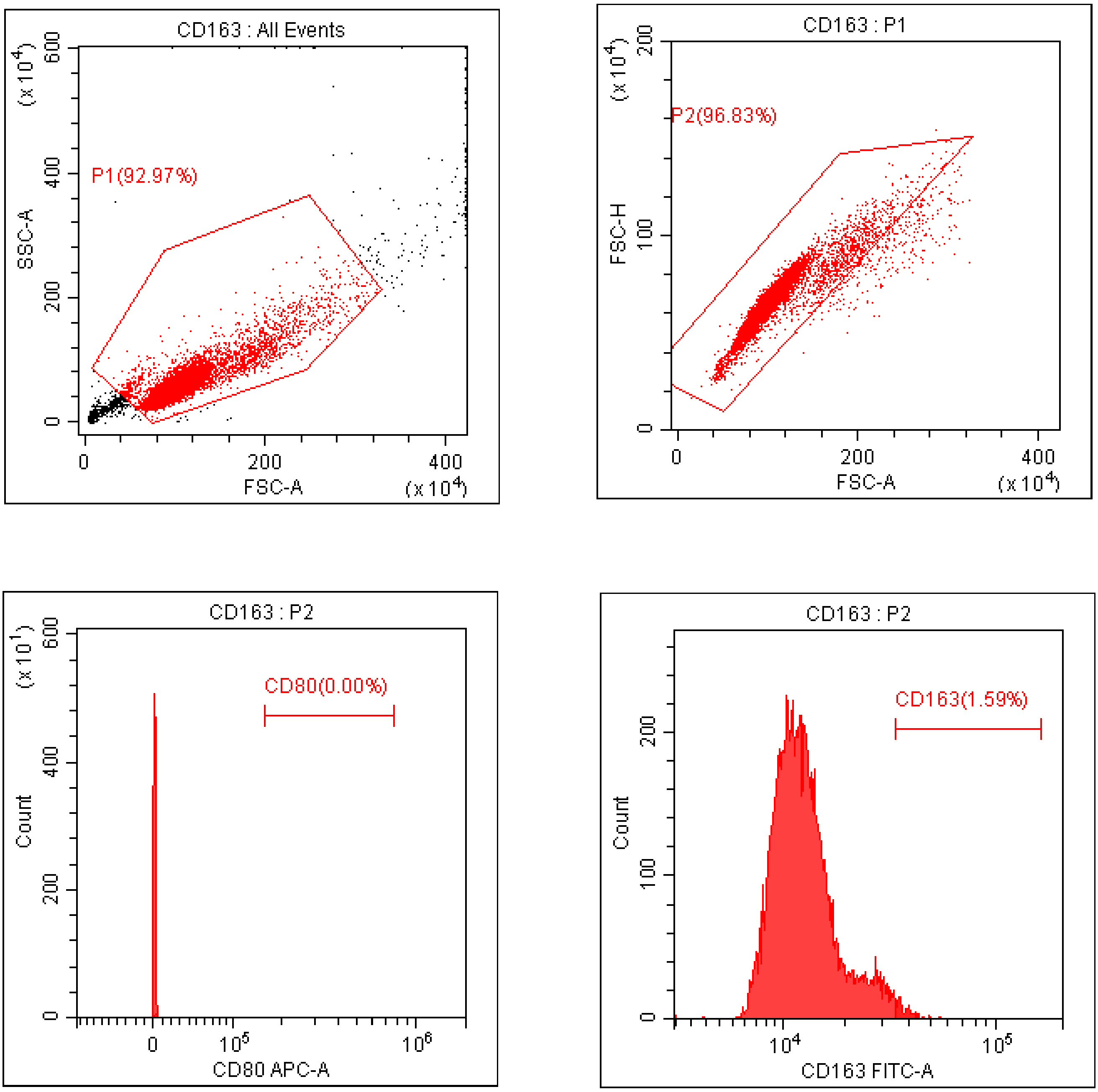

Figure S5   BC-1 CD80/CD163

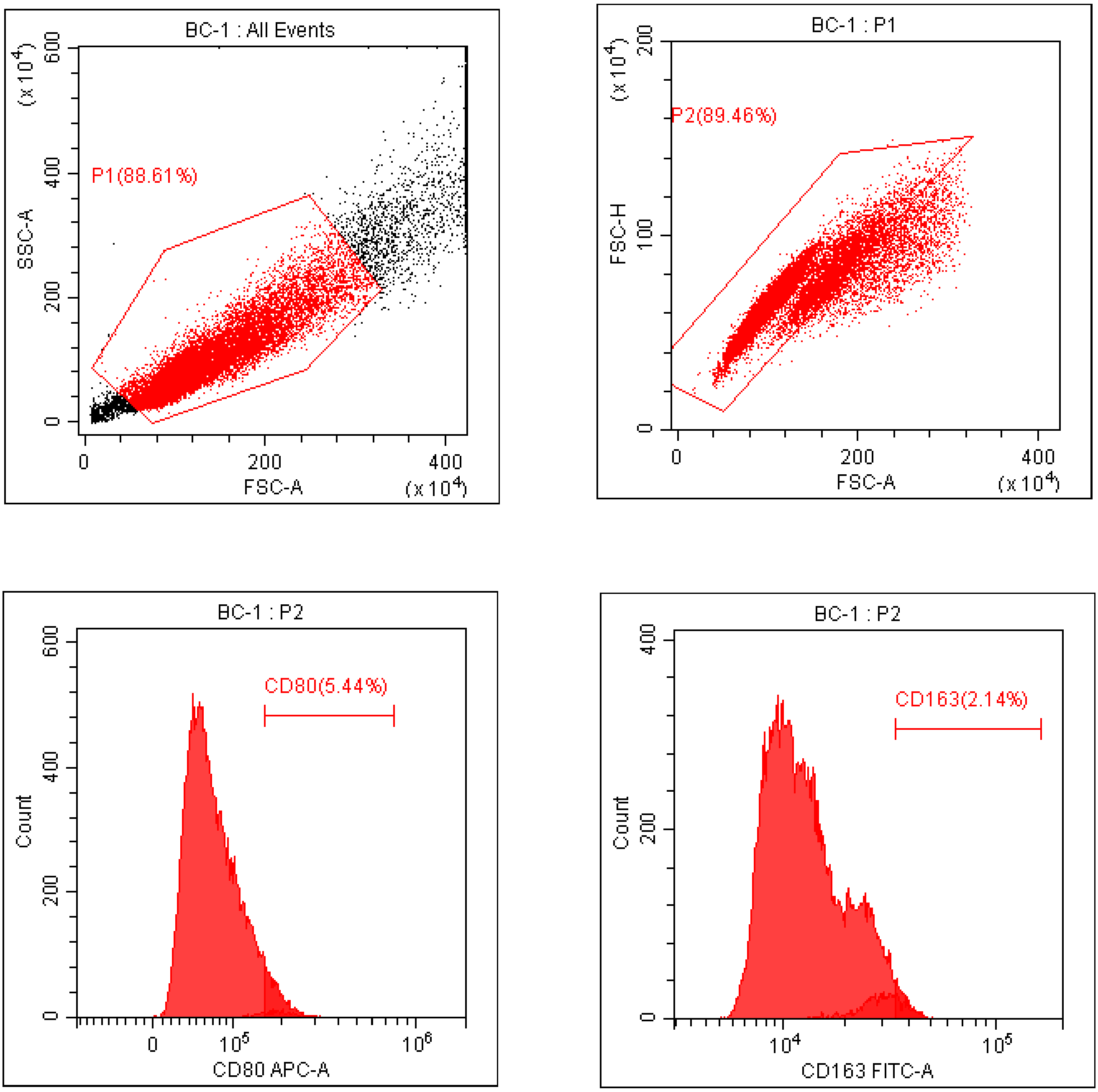

Figure S5   BC-2 CD80/CD163

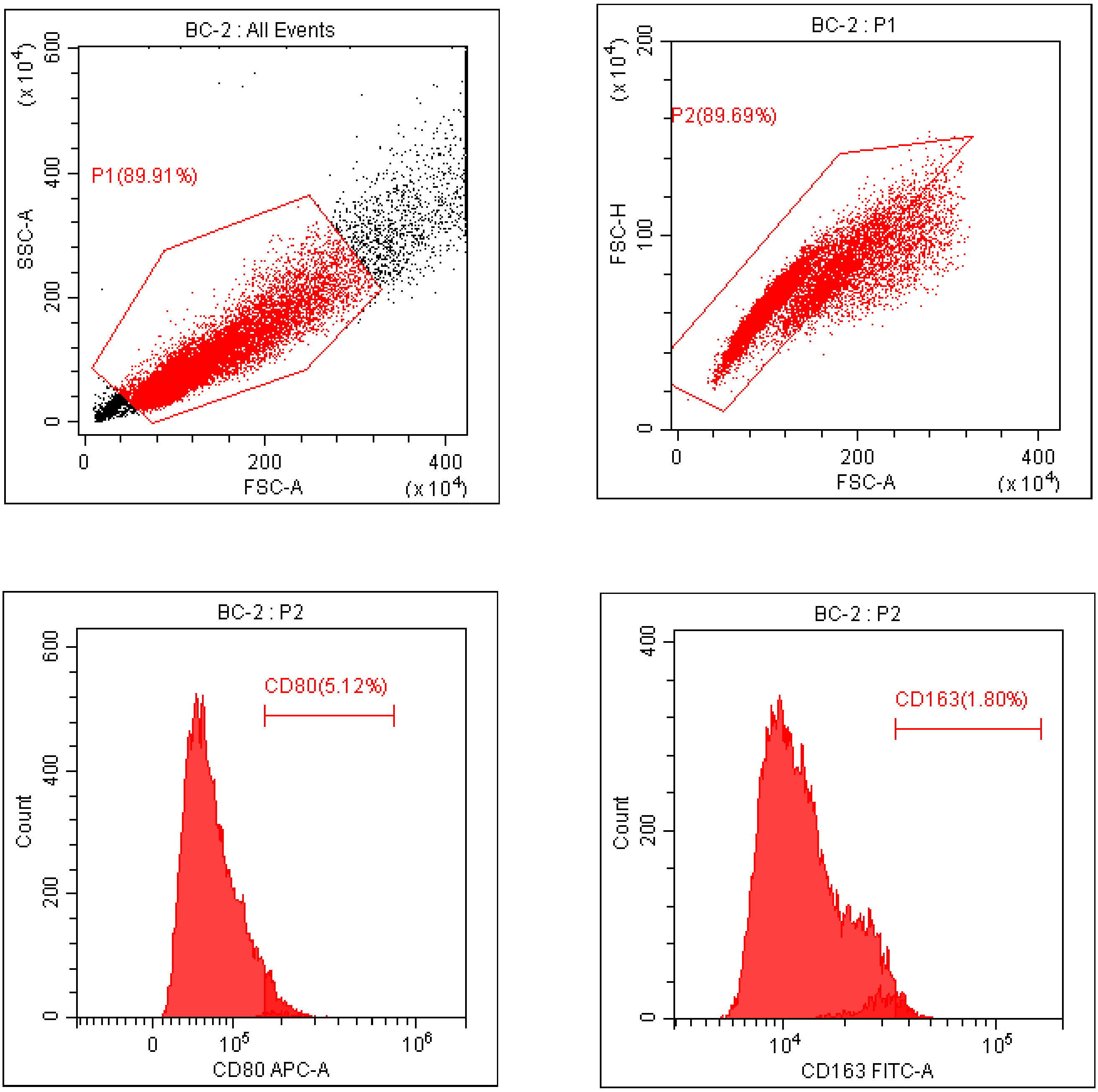

Figure S5 BC-3 CD80/CD163

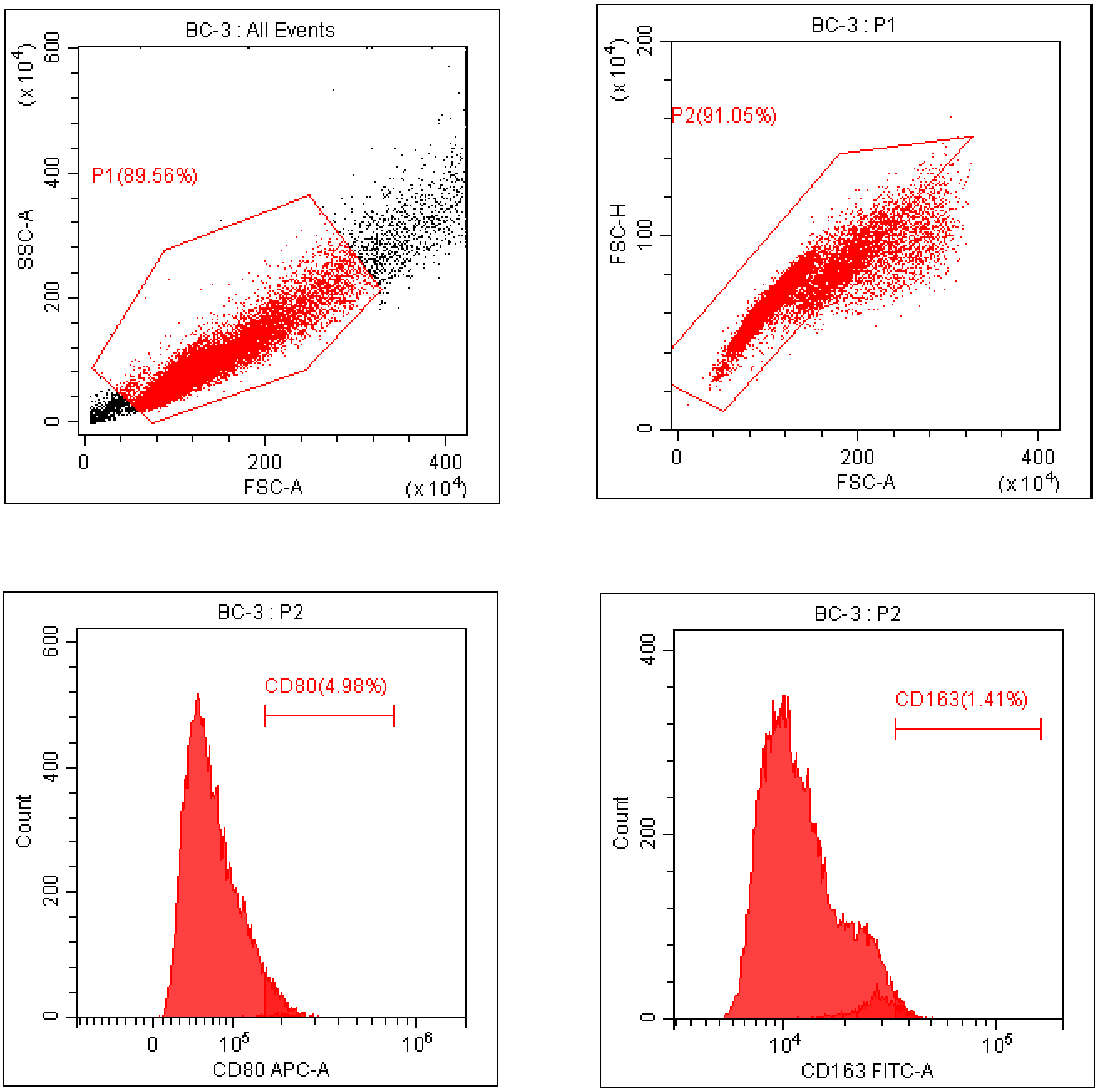

Figure S5 ox-LDL+BS-1 CD80/CD163

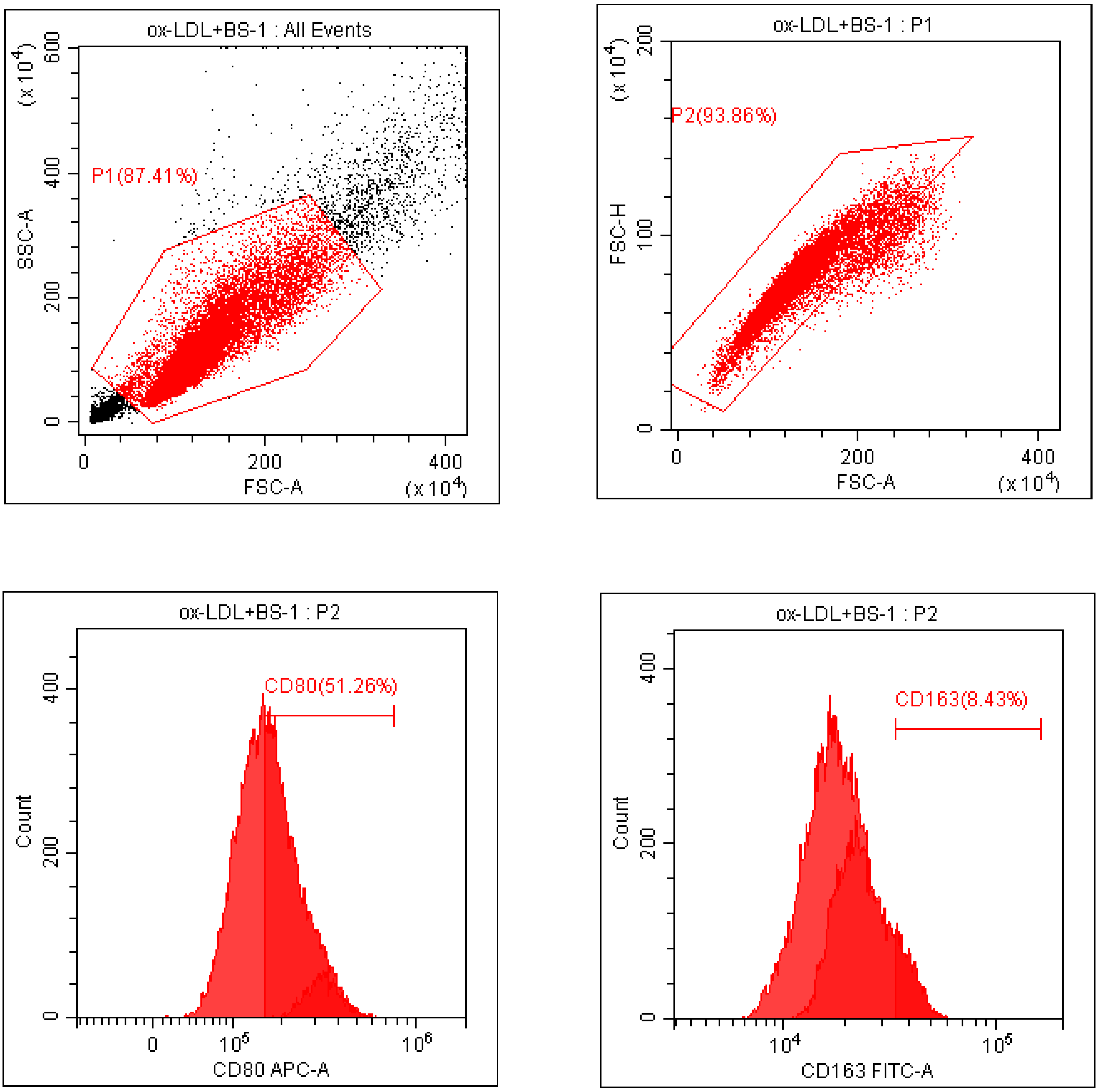

Figure S5 ox-LDL+BS-2 CD80/CD163

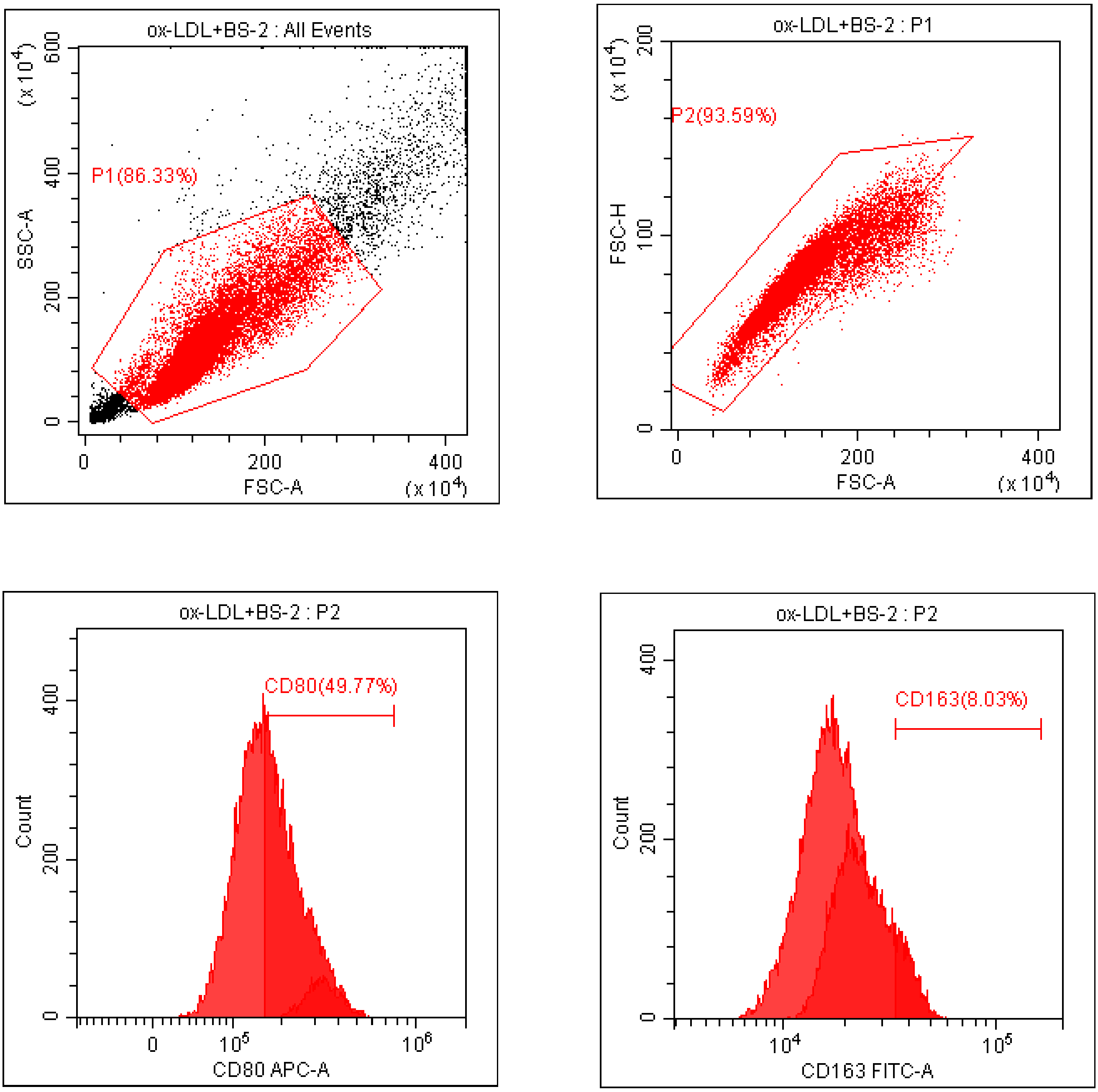

Figure S5 ox-LDL+BS-3 CD80/CD163

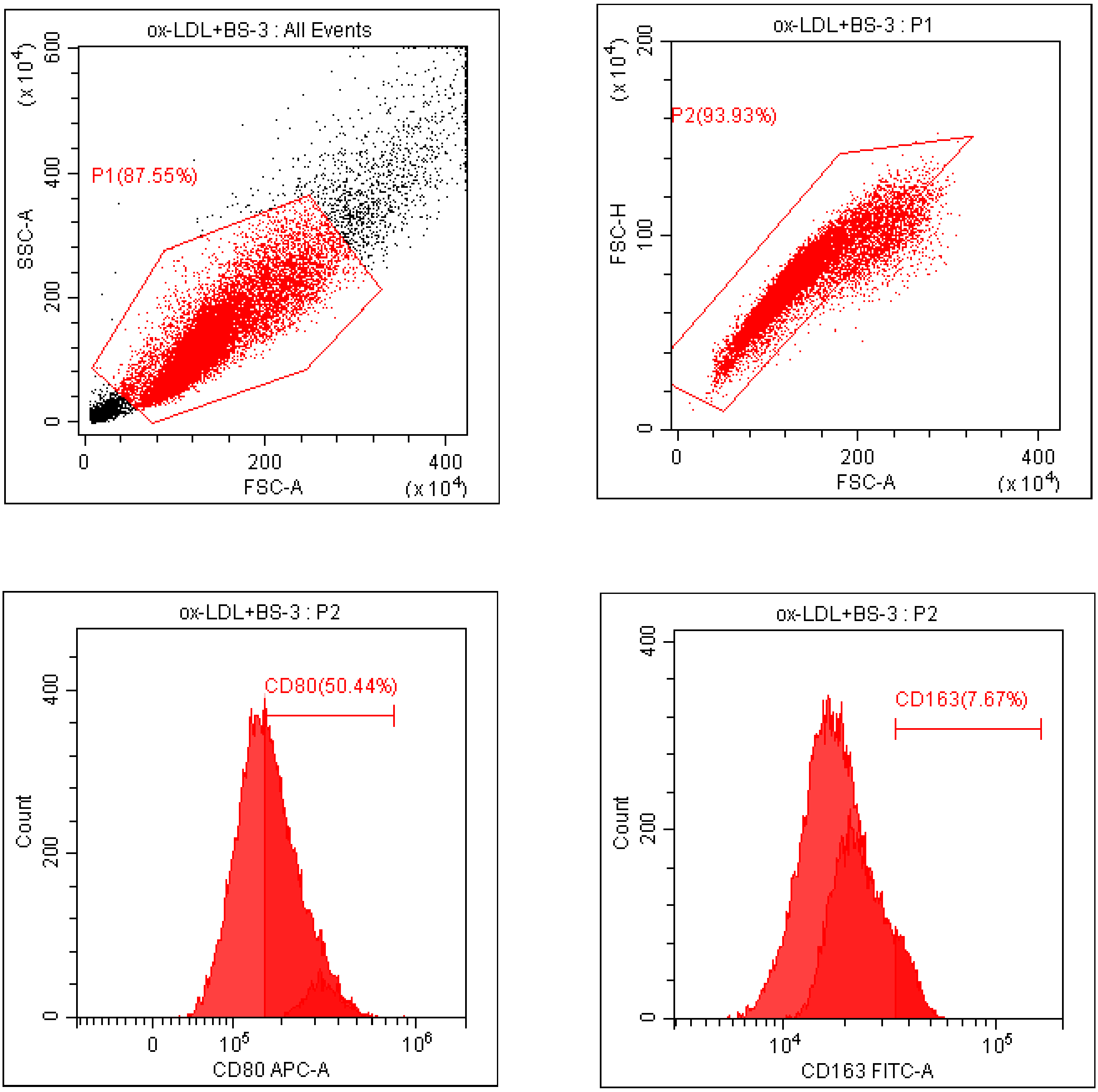

Figure S5 ox-LDL+YQHXF-CS-L-1 CD80/CD163

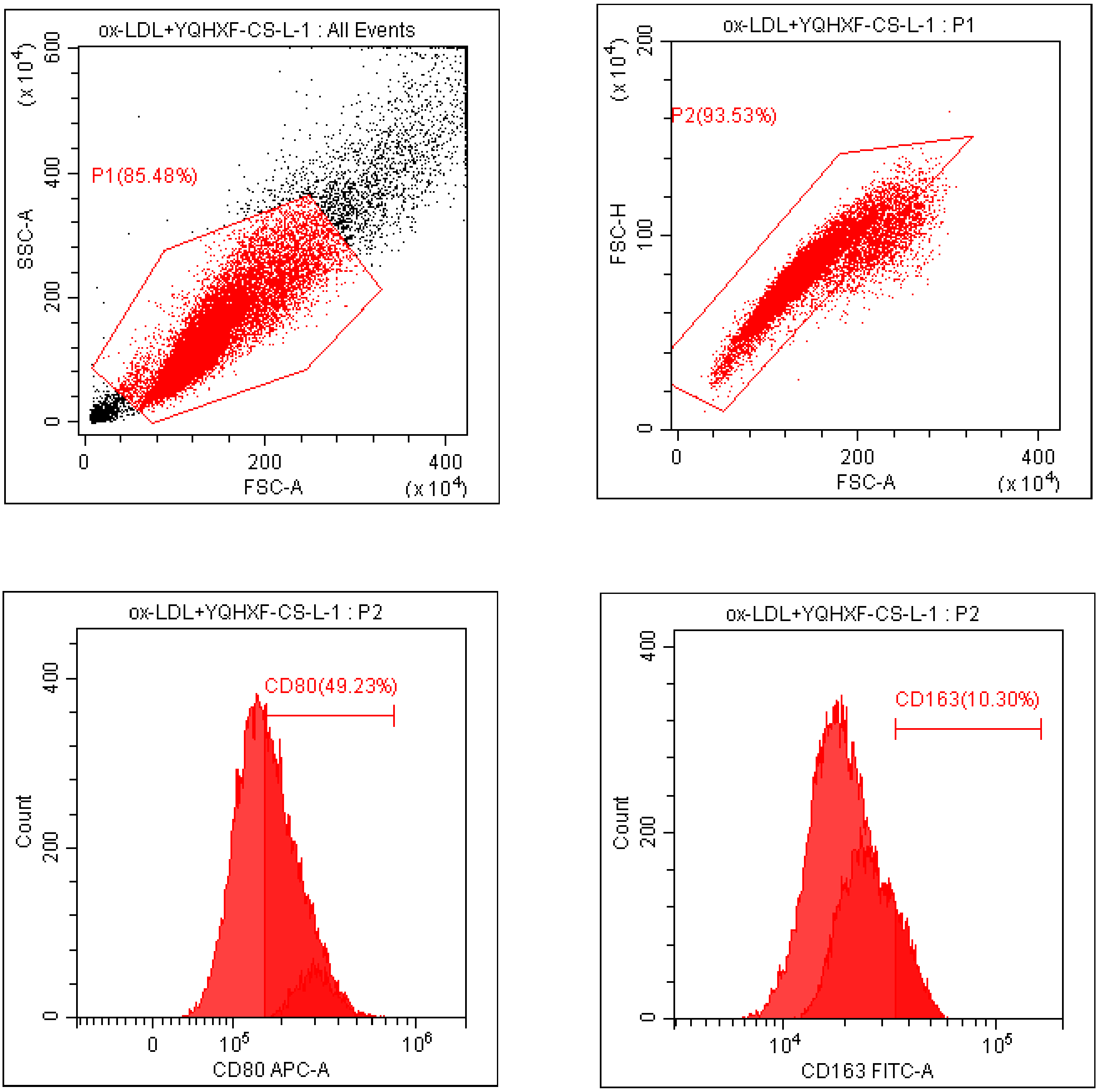

Figure S5 ox-LDL+YQHXF-CS-L-2 CD80/CD163

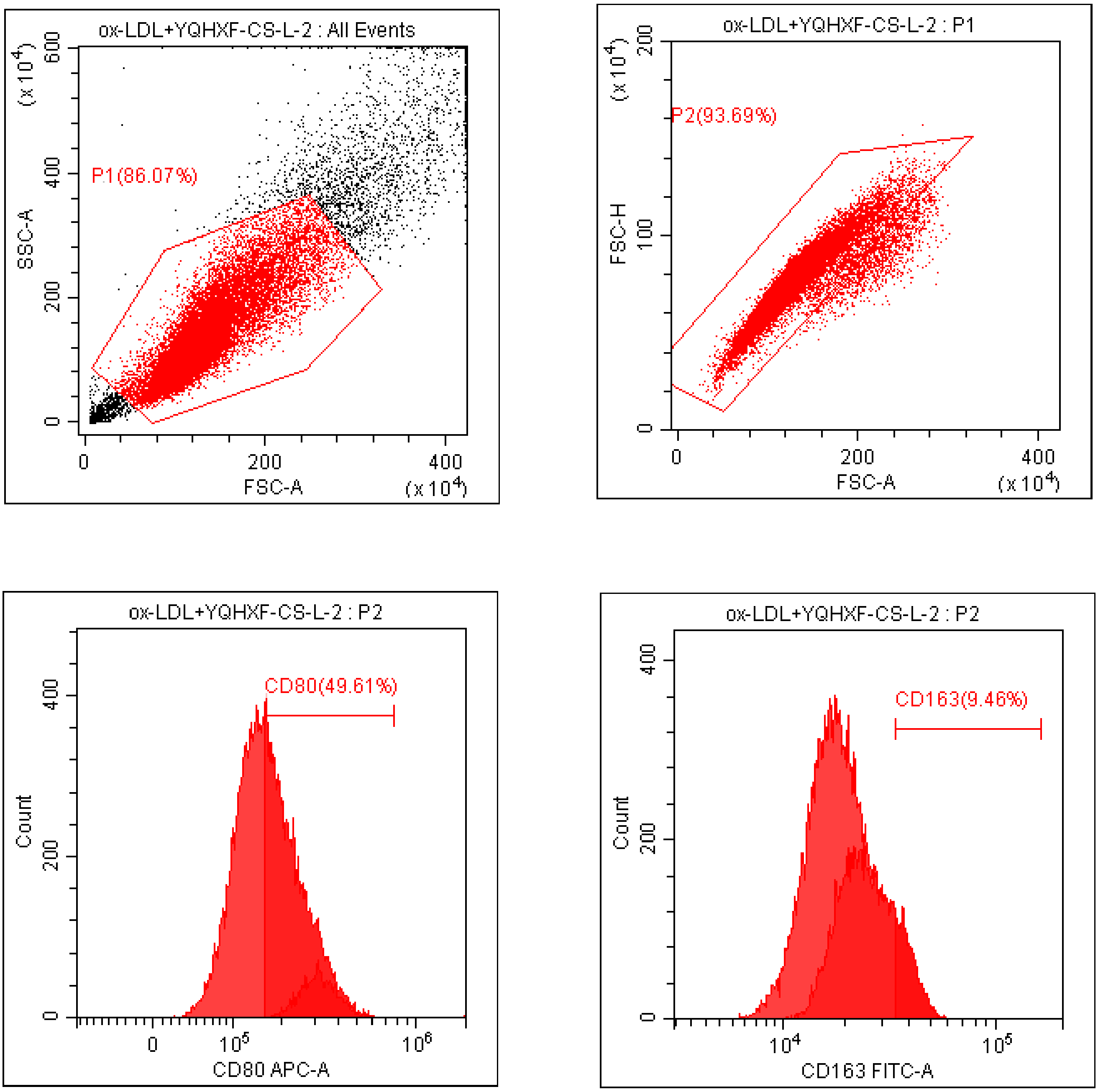

Figure S5 ox-LDL+YQHXF-CS-L-3 CD80/CD163

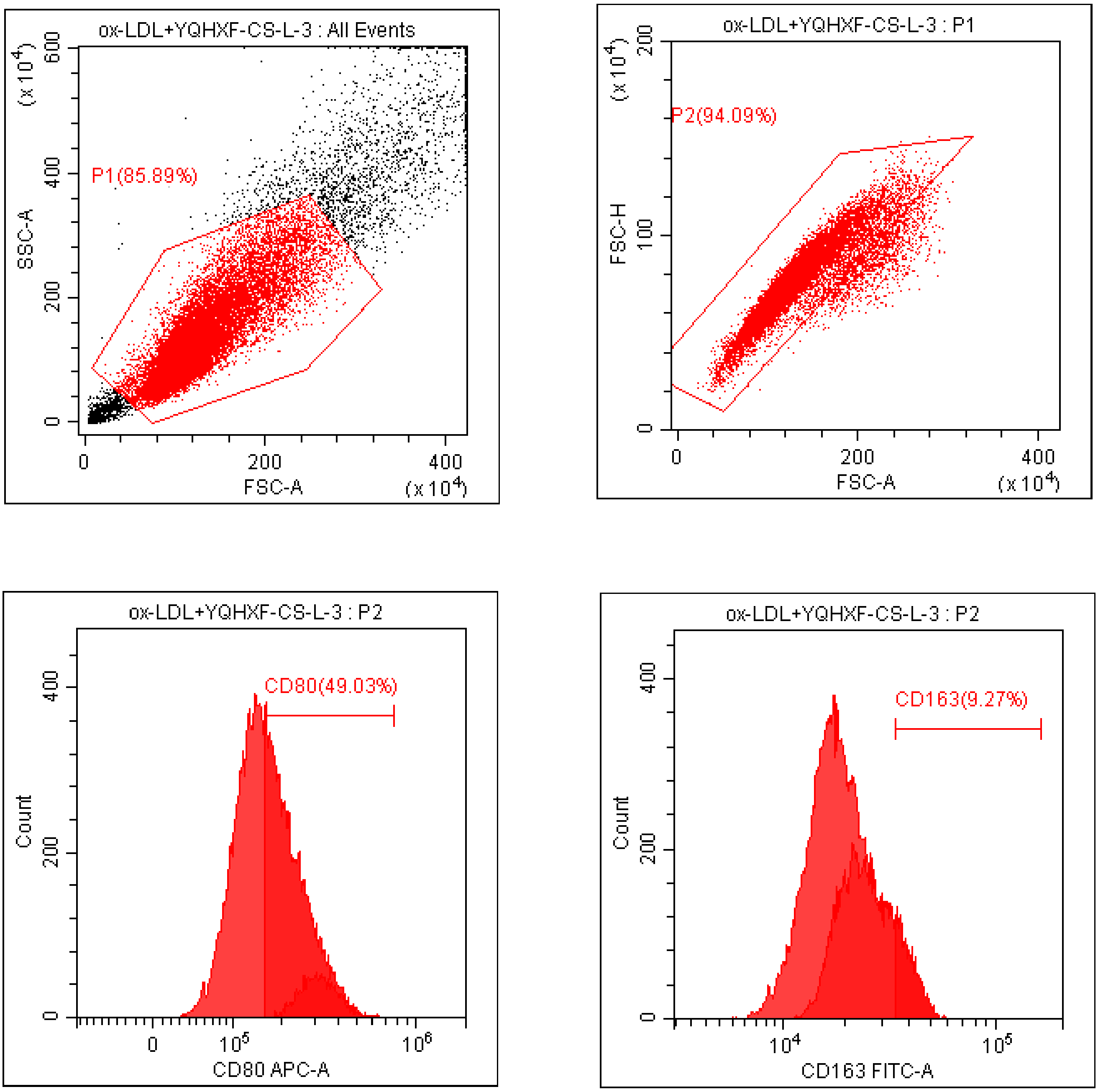

Figure S5 ox-LDL+YQHXF-CS-H-1 CD80/CD163

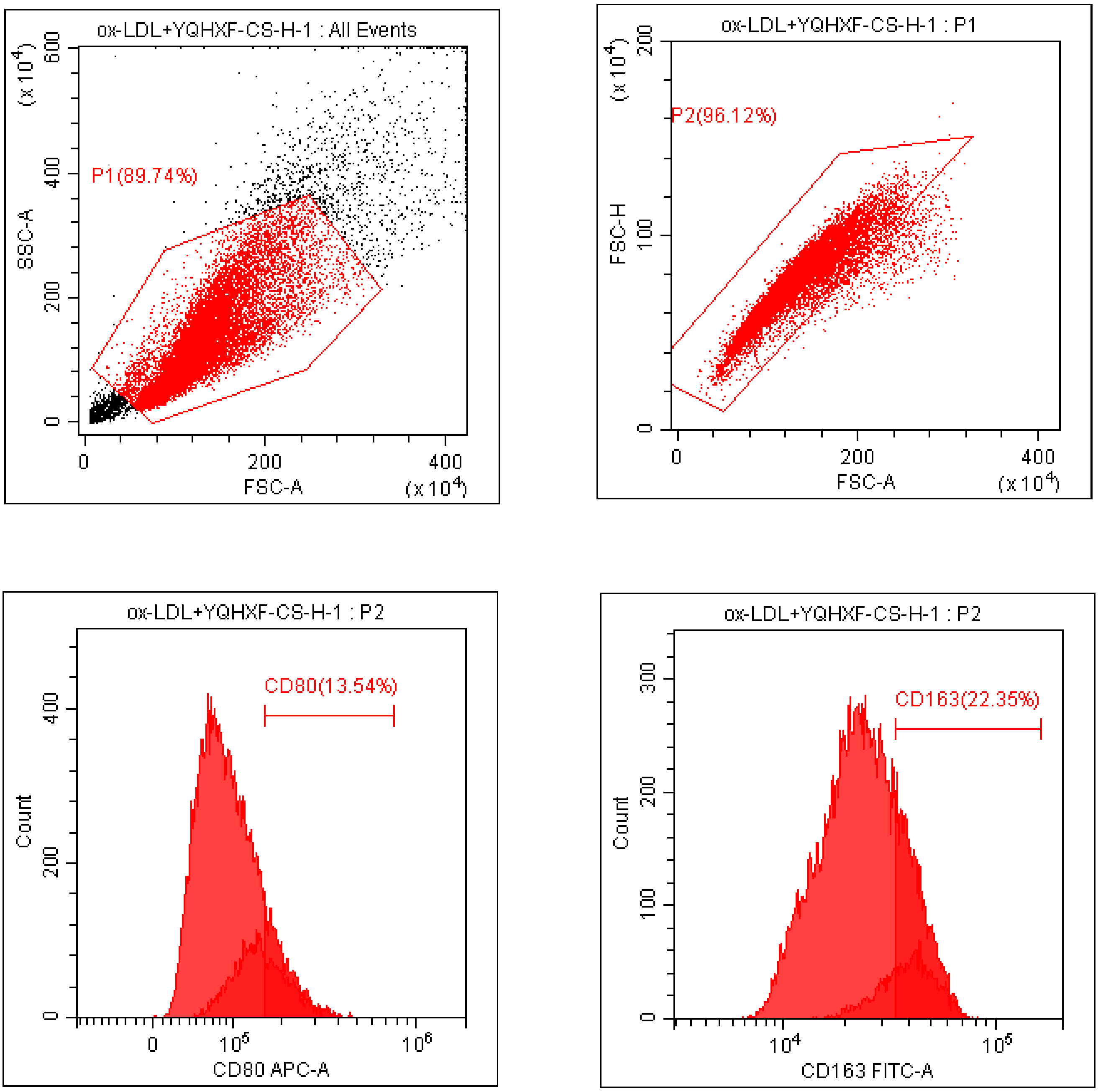

Figure S5 ox-LDL+YQHXF-CS-H-2 CD80/CD163

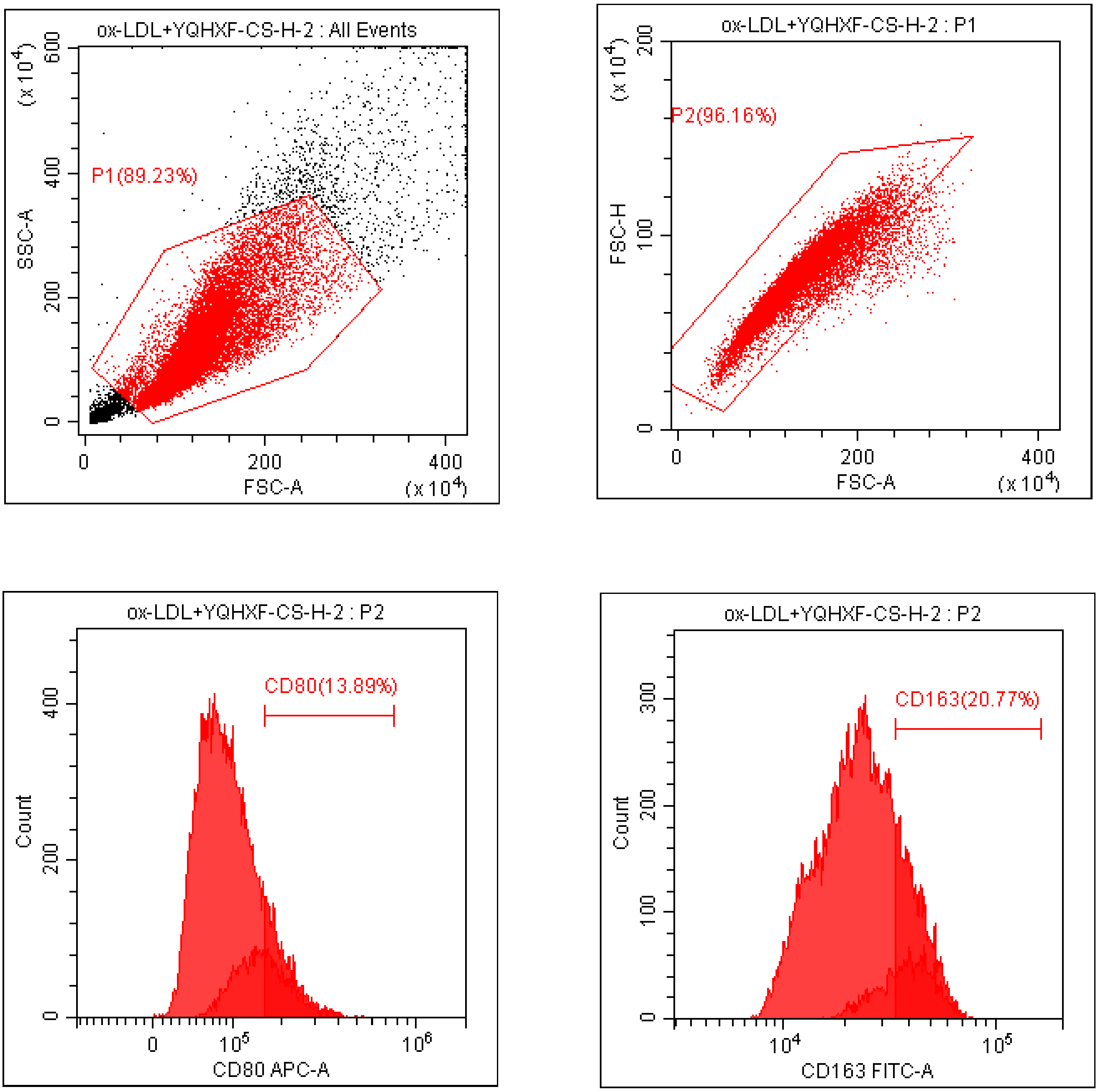

Figure S5 ox-LDL+YQHXF-CS-H-3 CD80/CD163

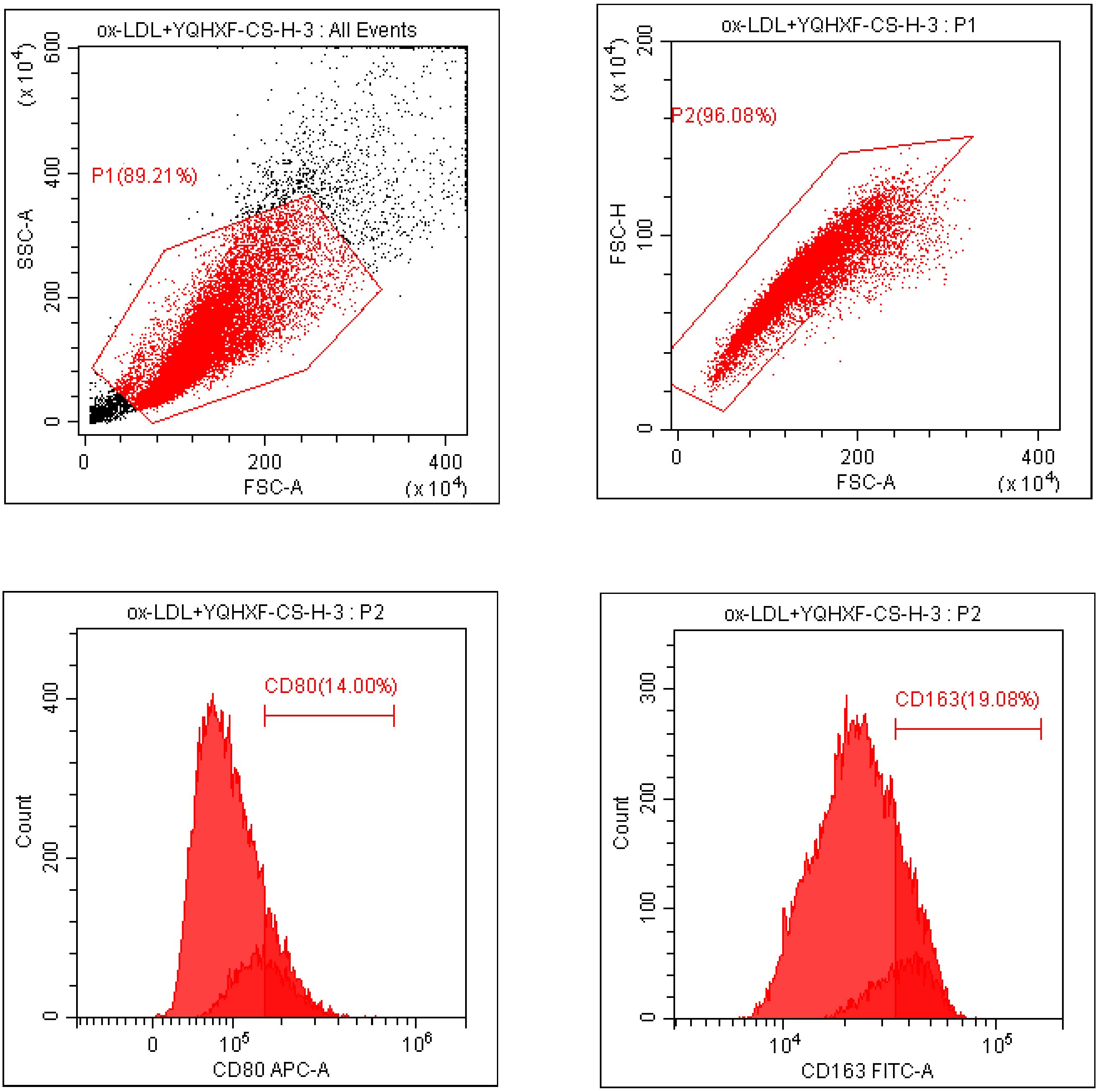

Figure S5 ox-LDL+YQHXF-CS-M-1 CD80/CD163

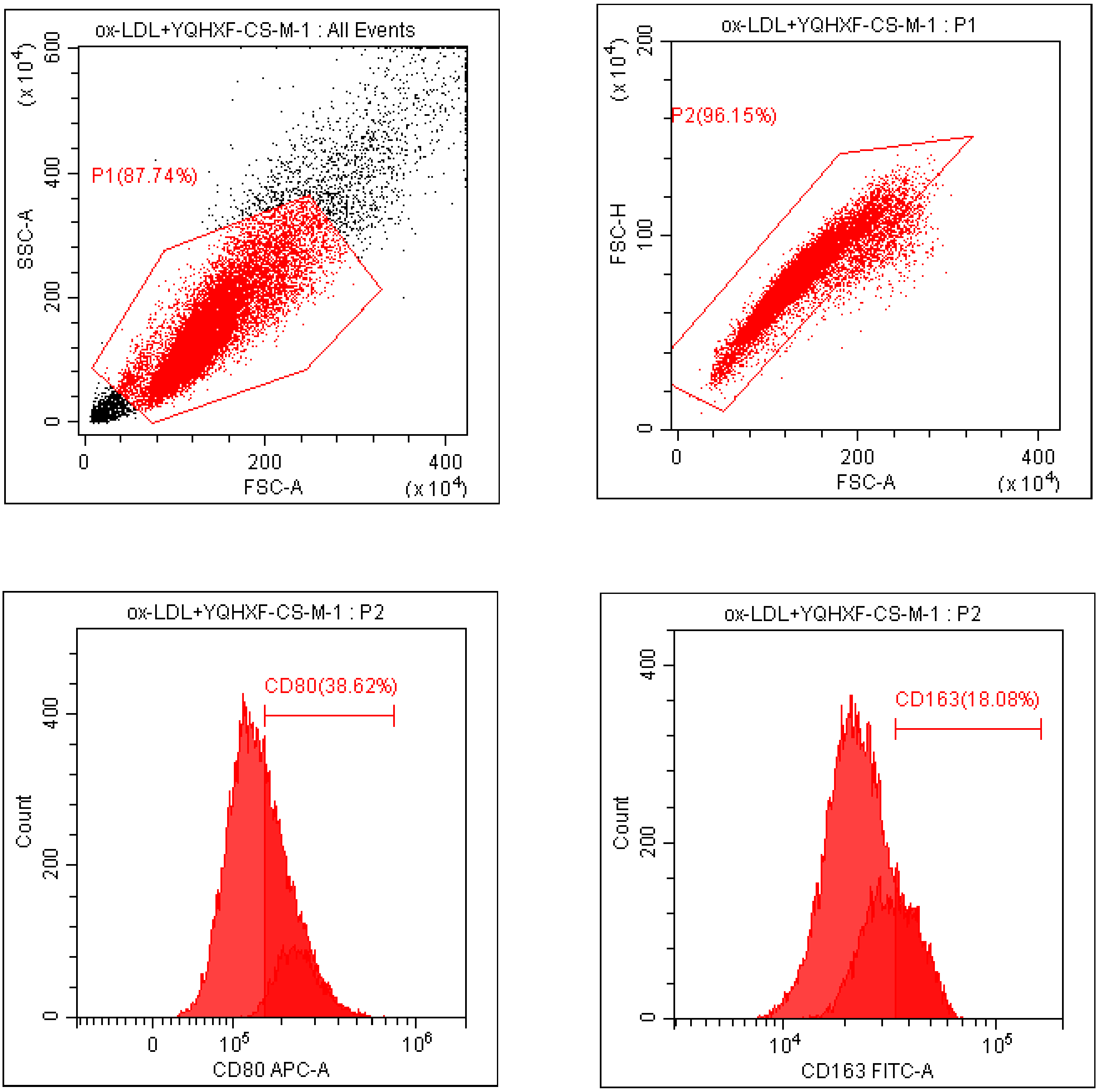

Figure S5 ox-LDL+YQHXF-CS-M-2 CD80/CD163

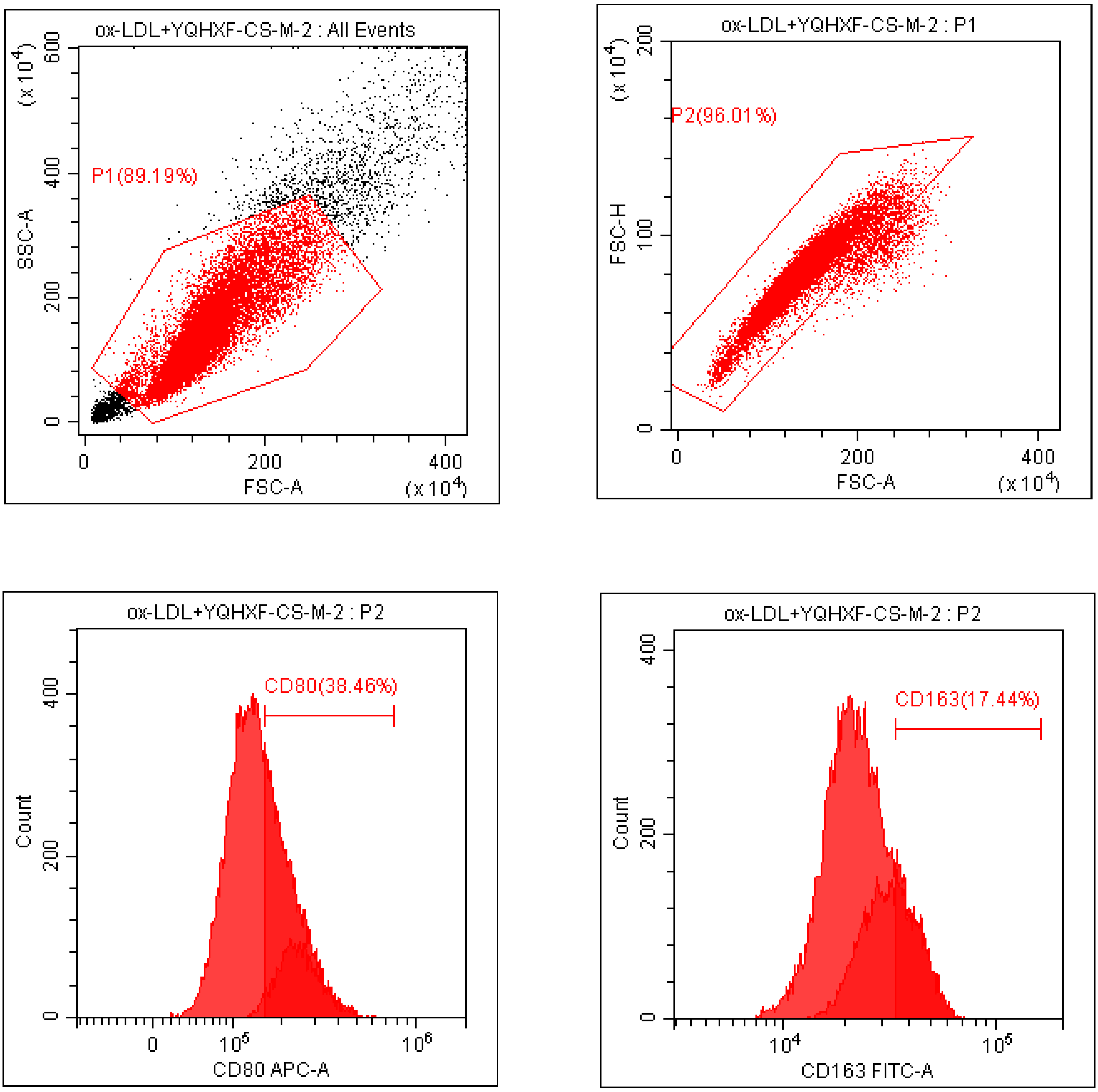

Figure S5 ox-LDL+YQHXF-CS-M-3 CD80/CD163

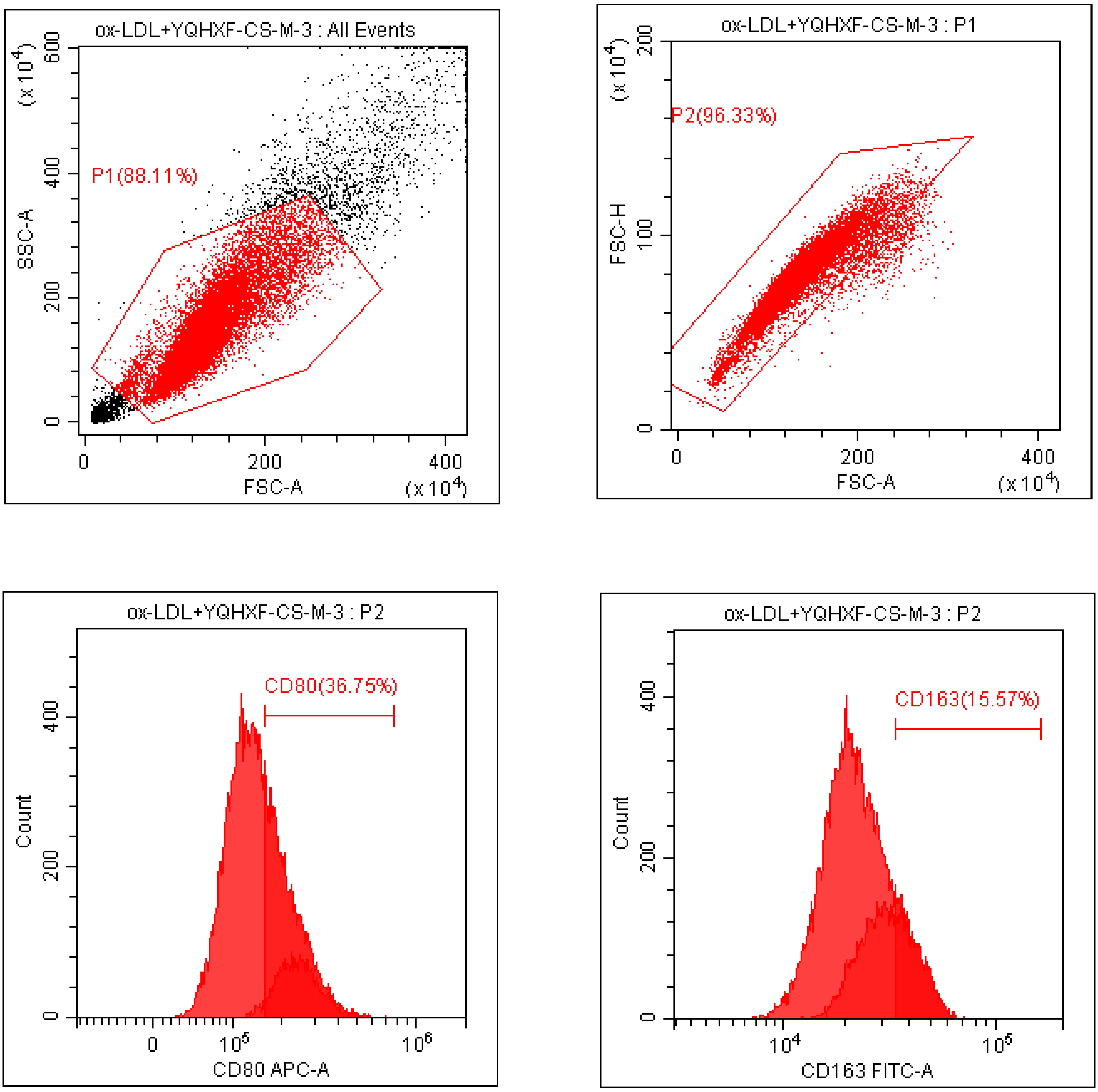

Figure S5 BLANK CD86/CD206

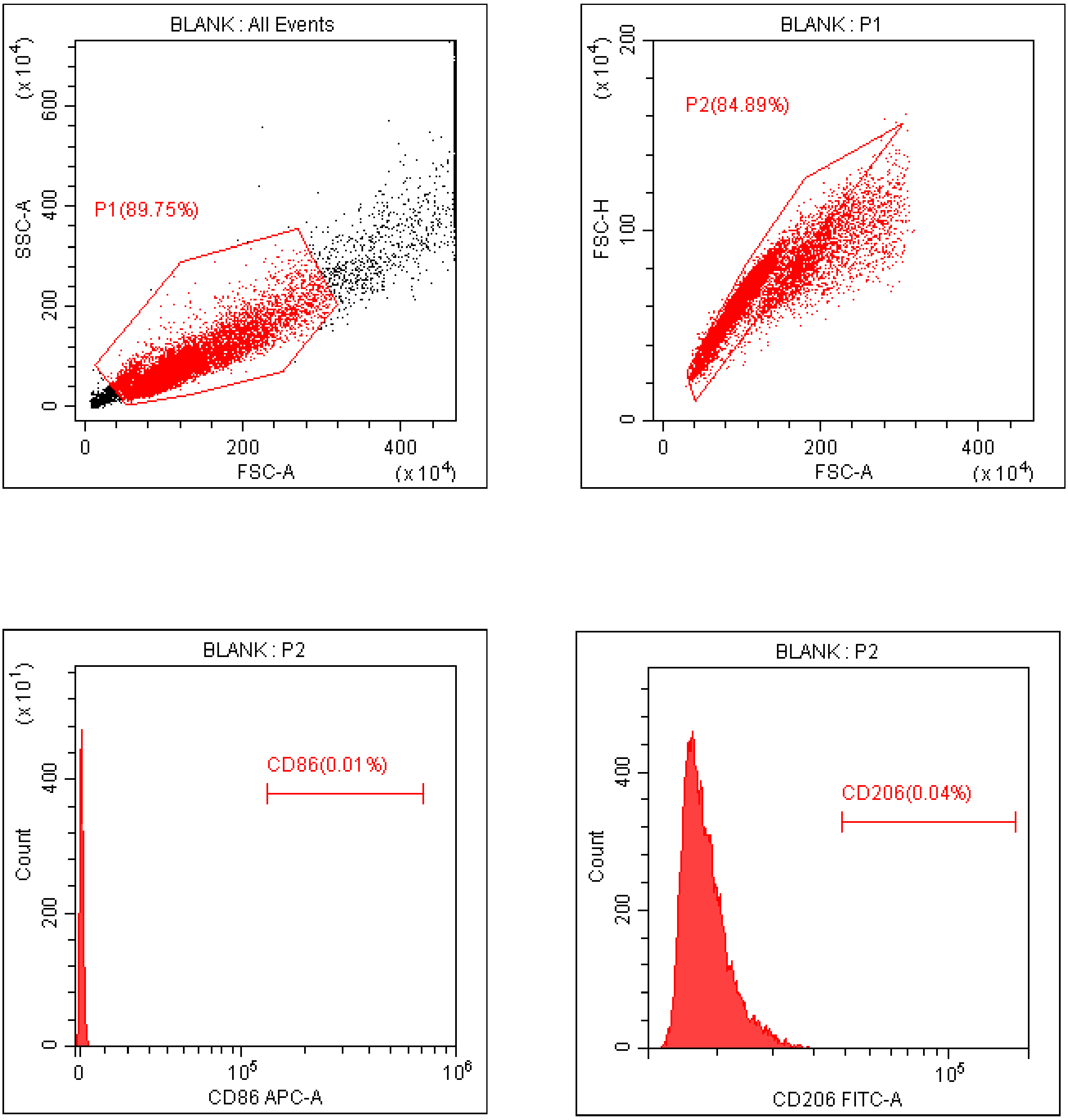

Figure S5 CD86

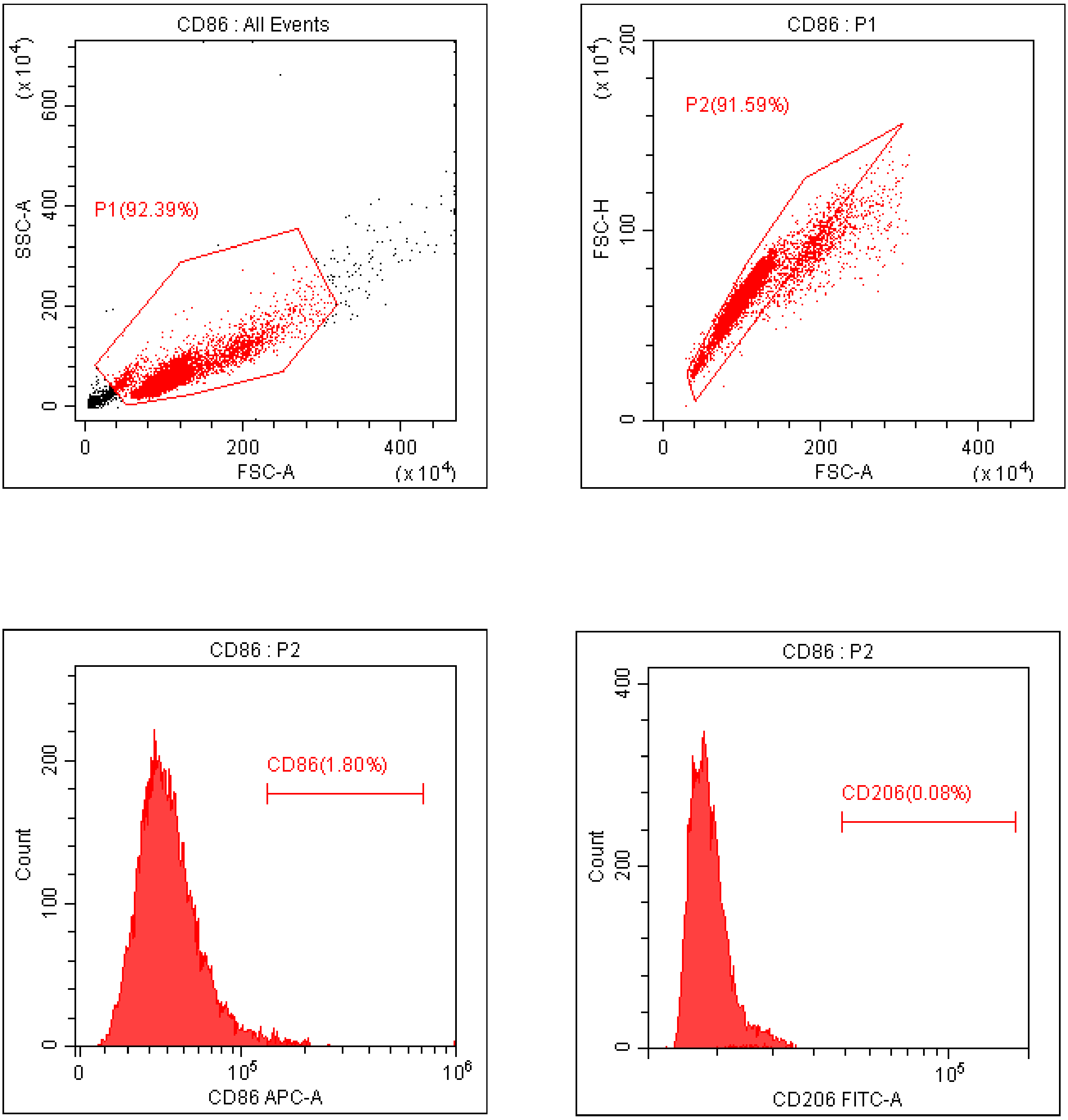

Figure S5 CD206

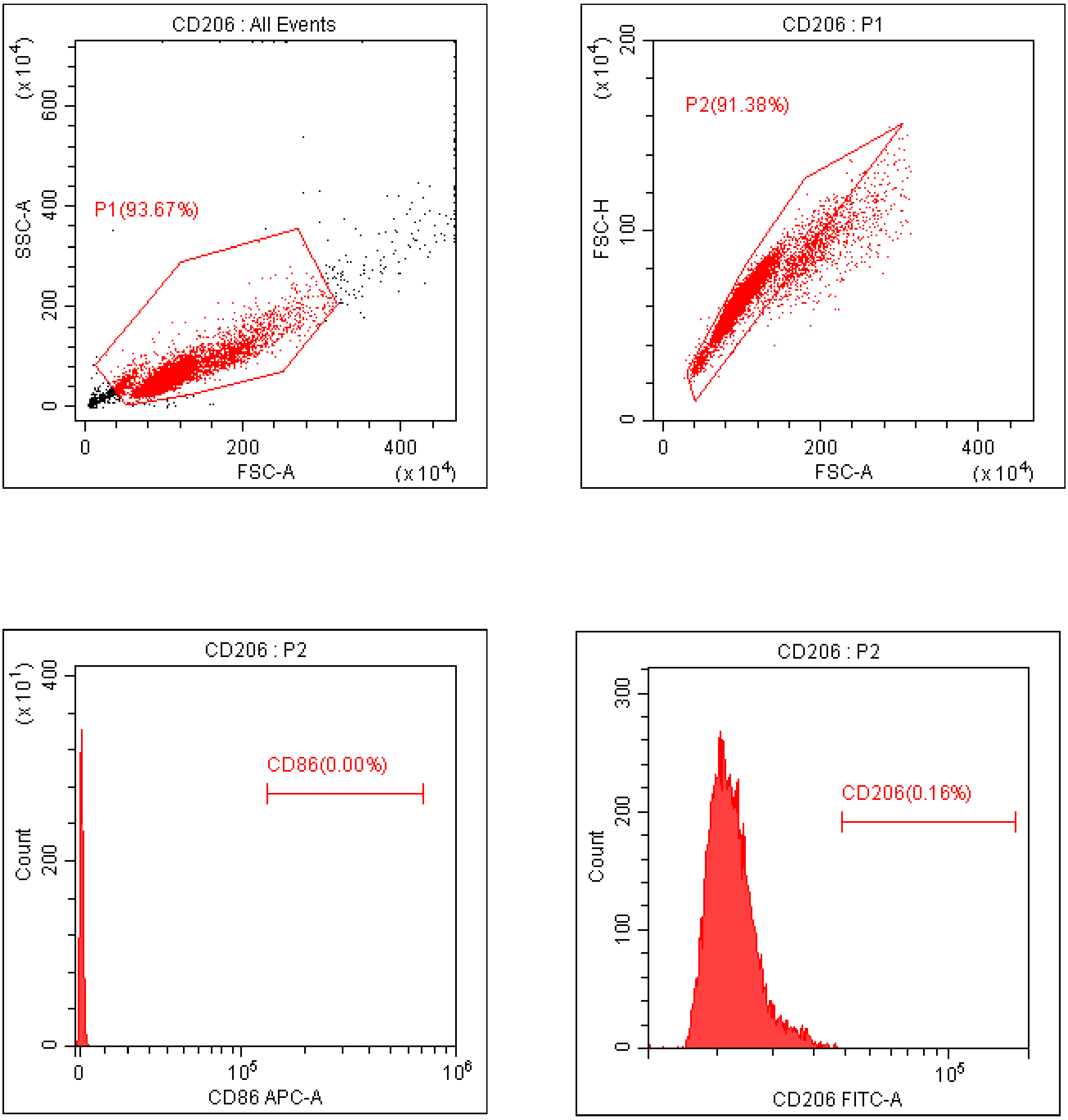

Figure S5 BC-1 CD86/CD206

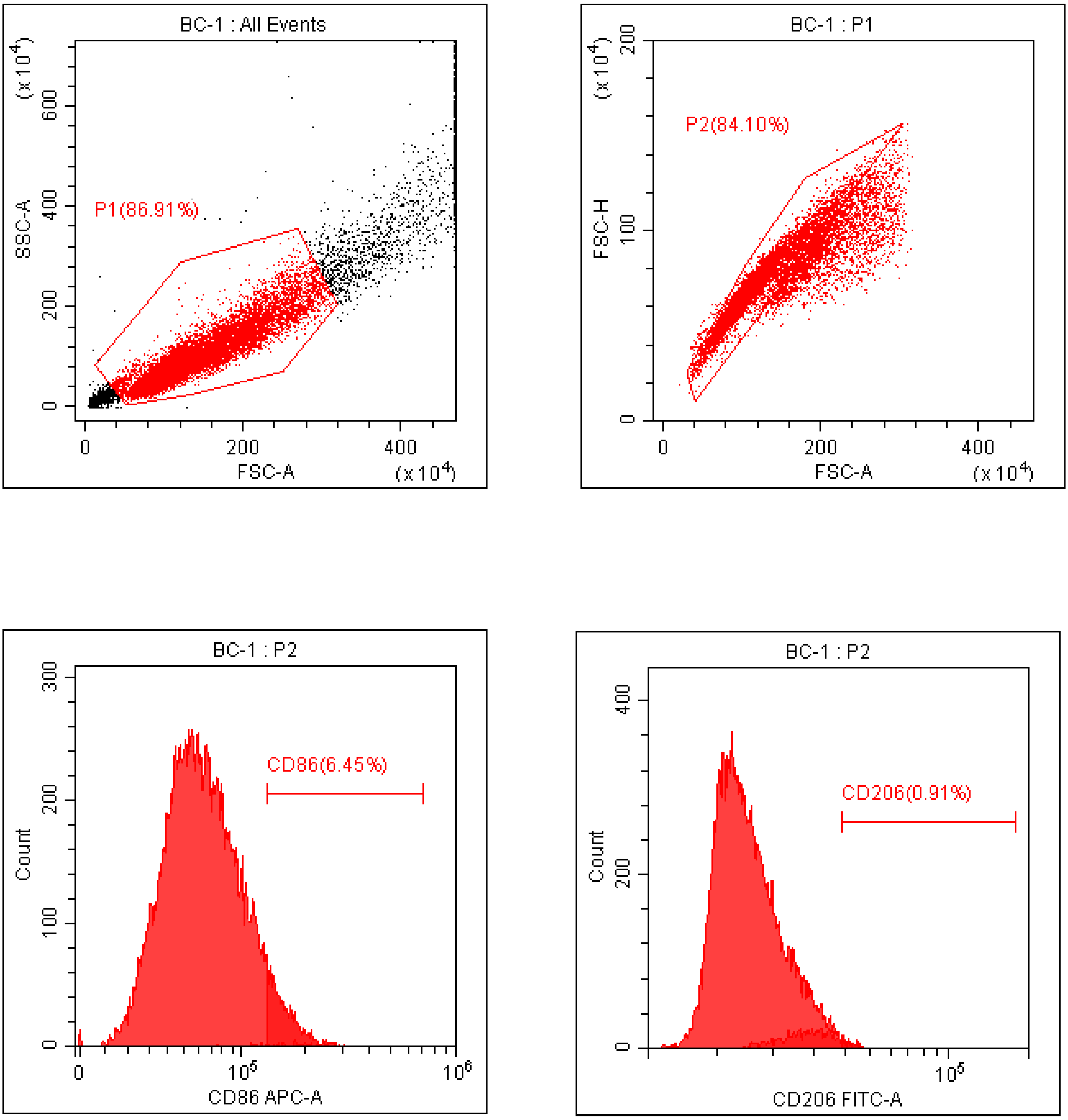

Figure S5 BC-2 CD86/CD206

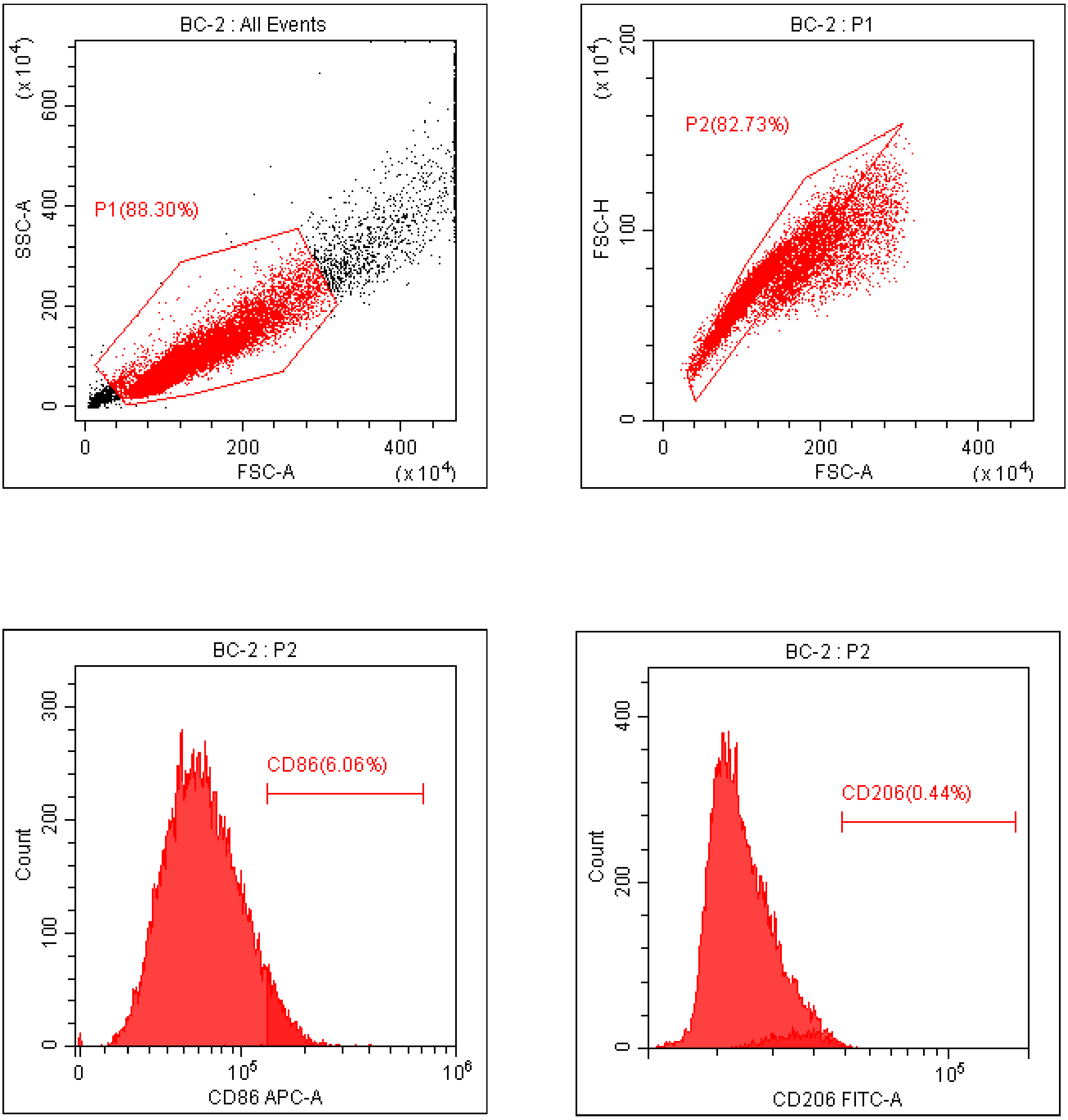

Figure S5 BC-3 CD86/CD206

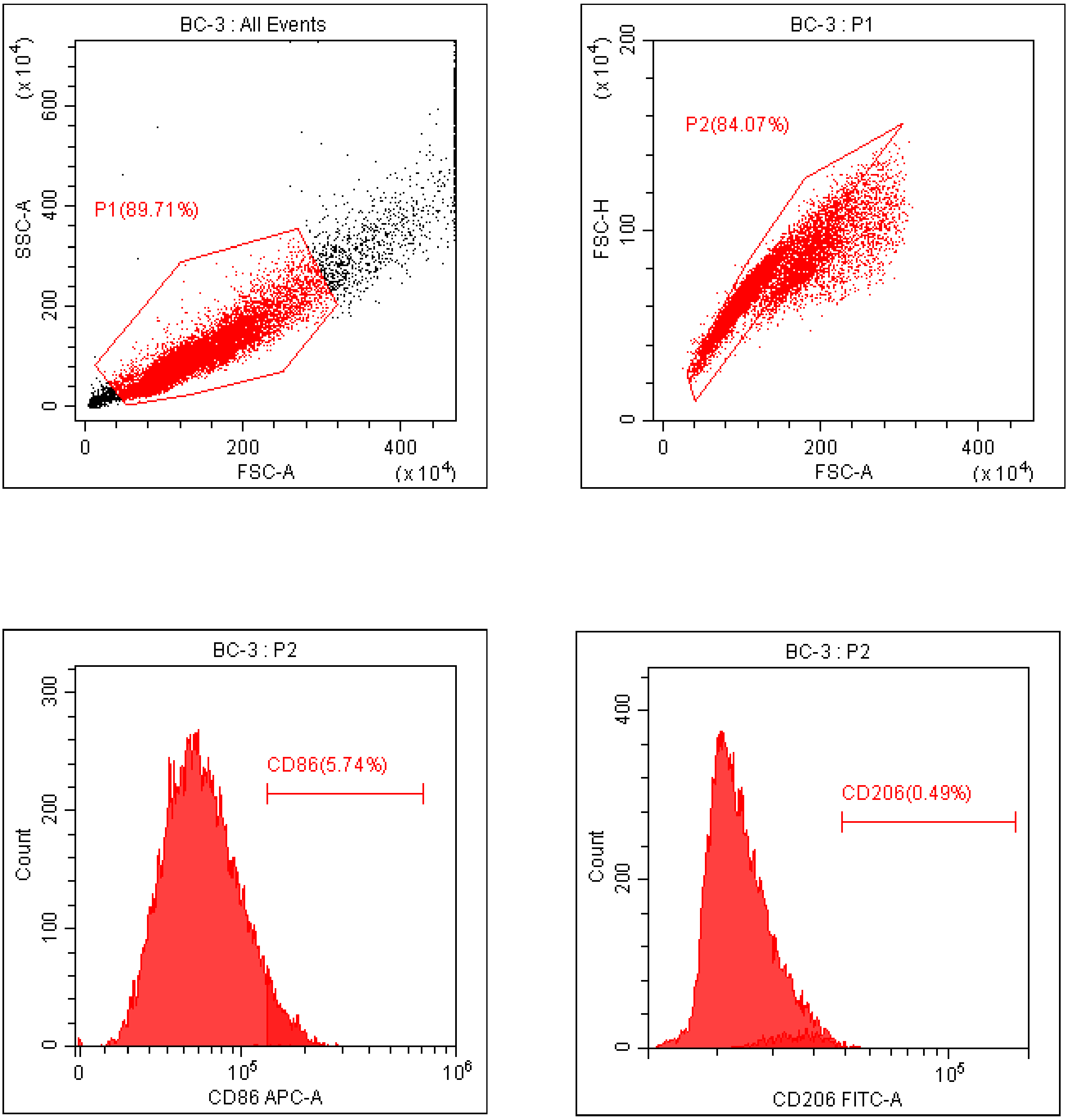

Figure S5 ox-LDL+BS-1 CD86/CD206

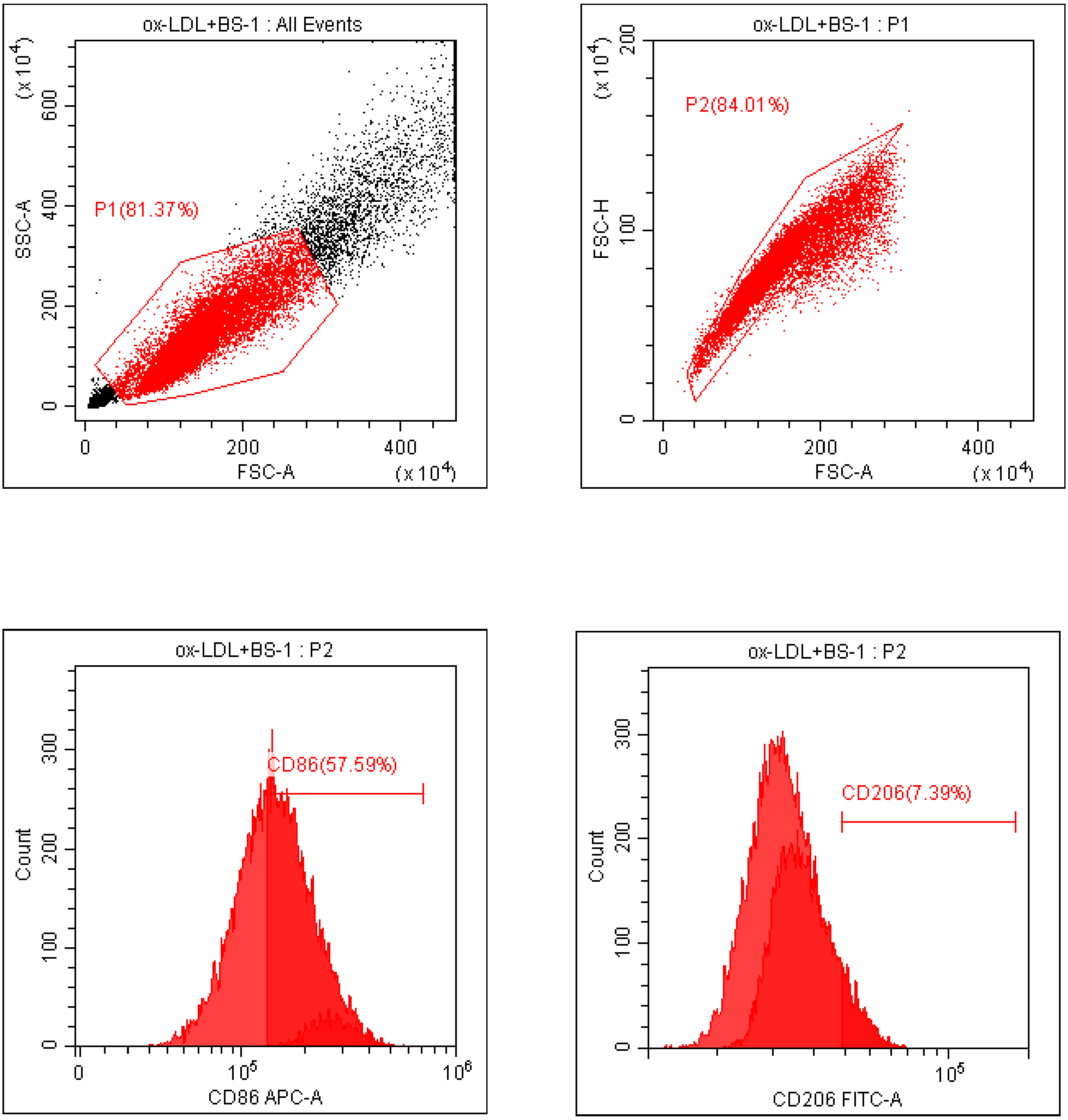

Figure S5 ox-LDL+BS-2 CD86/CD206

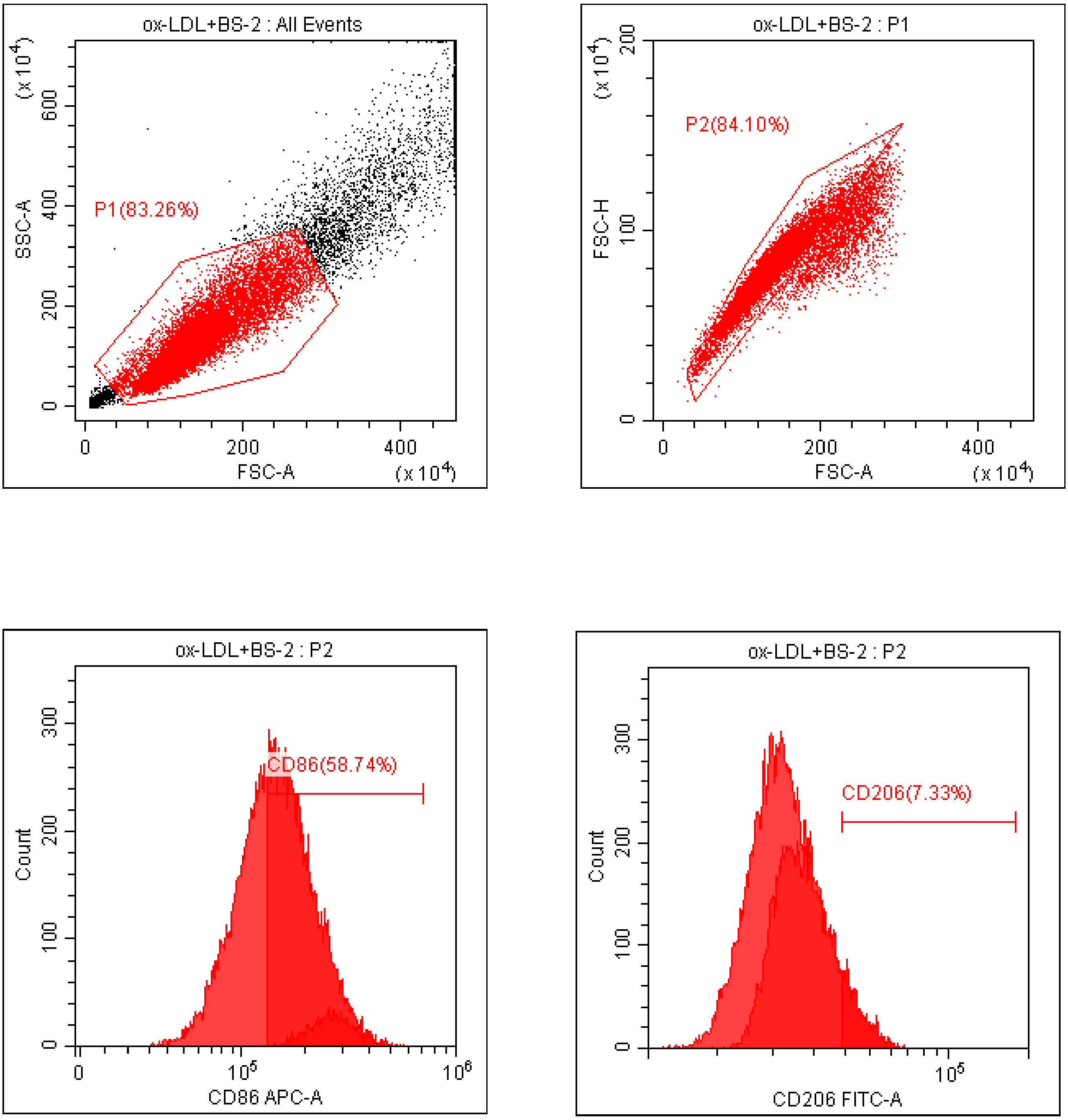

Figure S5 ox-LDL+BS-3 CD86/CD206

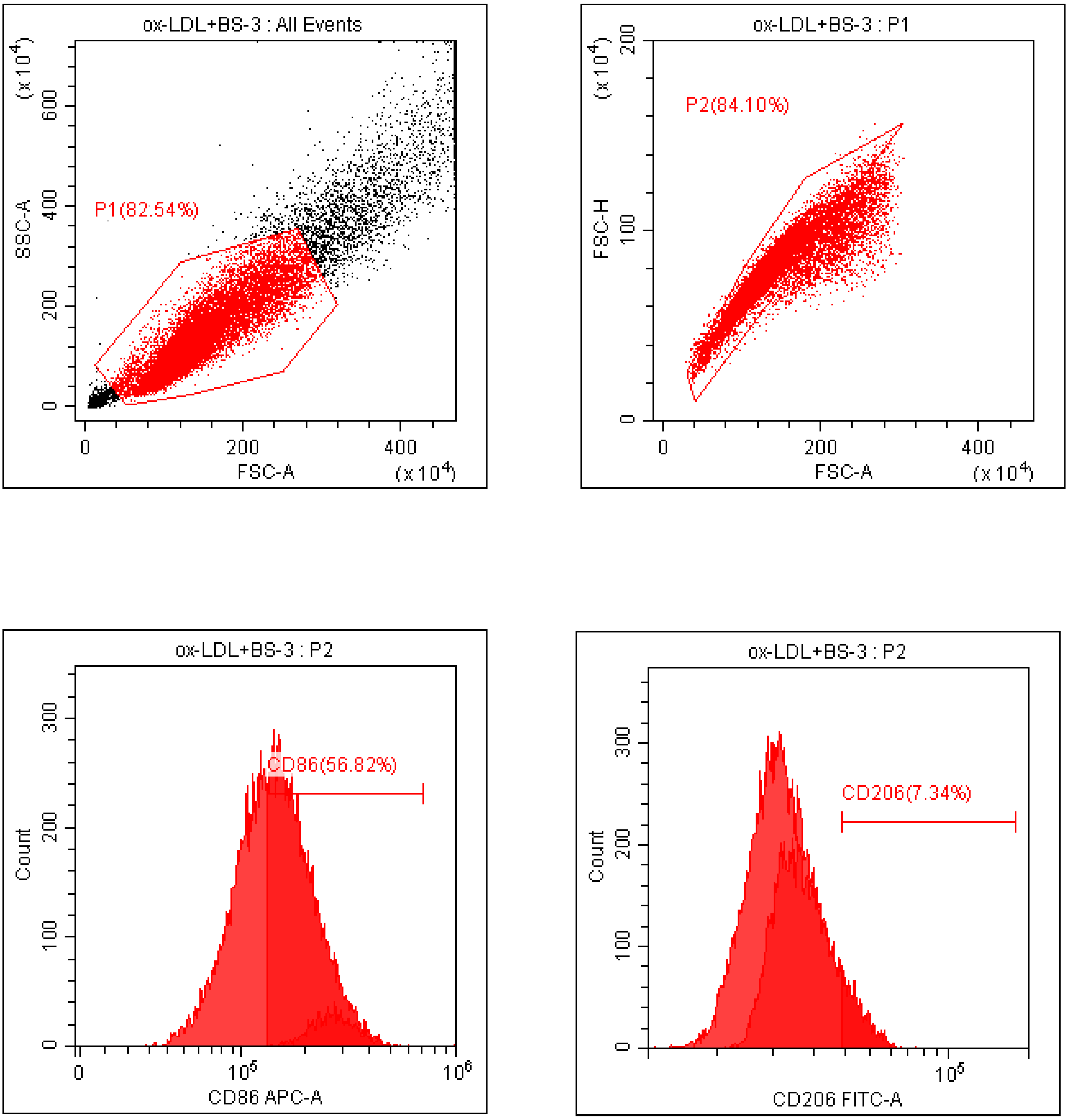

Figure S5 ox-LDL+YQHXF-CS-L-1 CD86/CD206

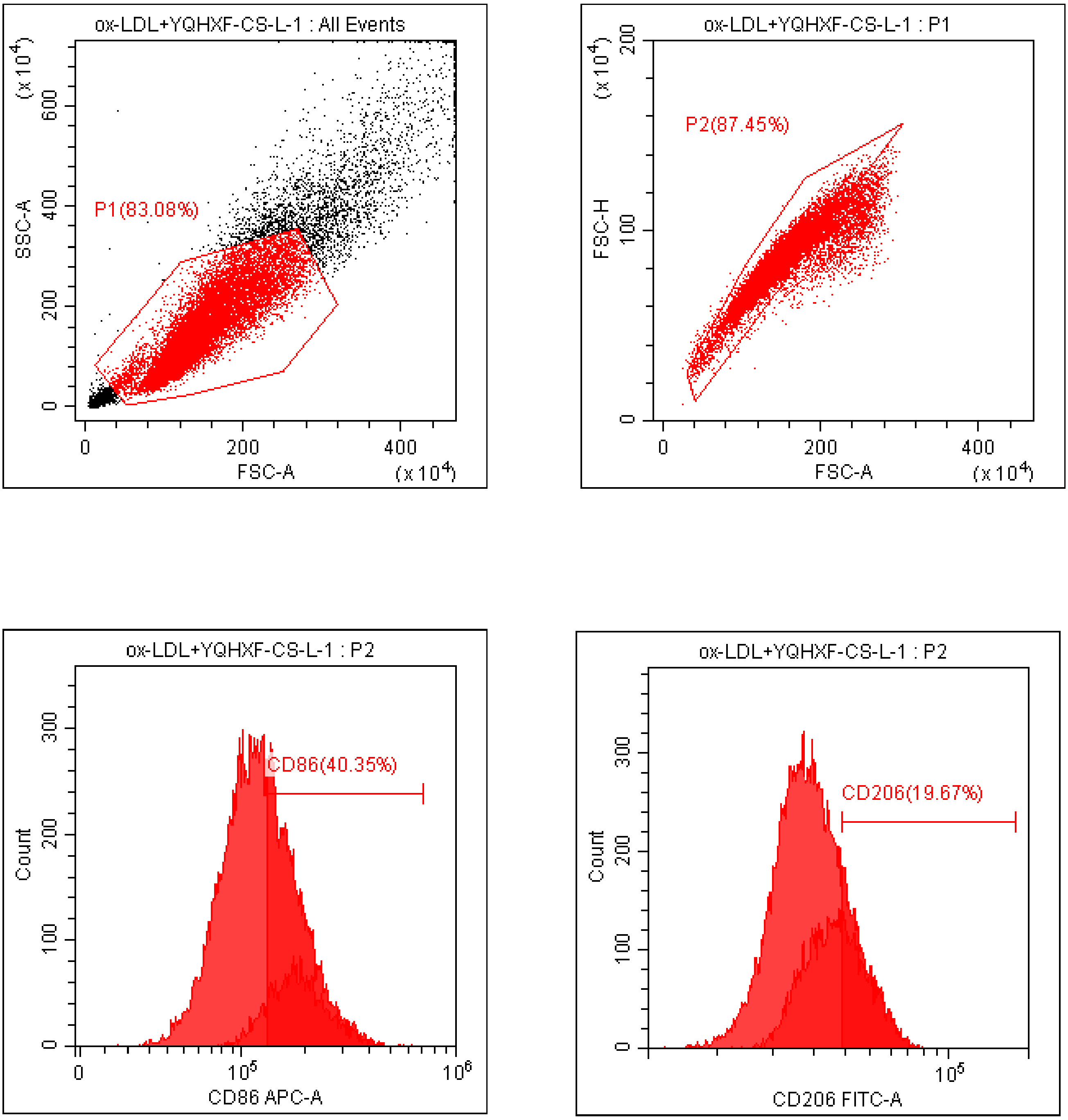

Figure S5 ox-LDL+YQHXF-CS-L-2 CD86/CD206

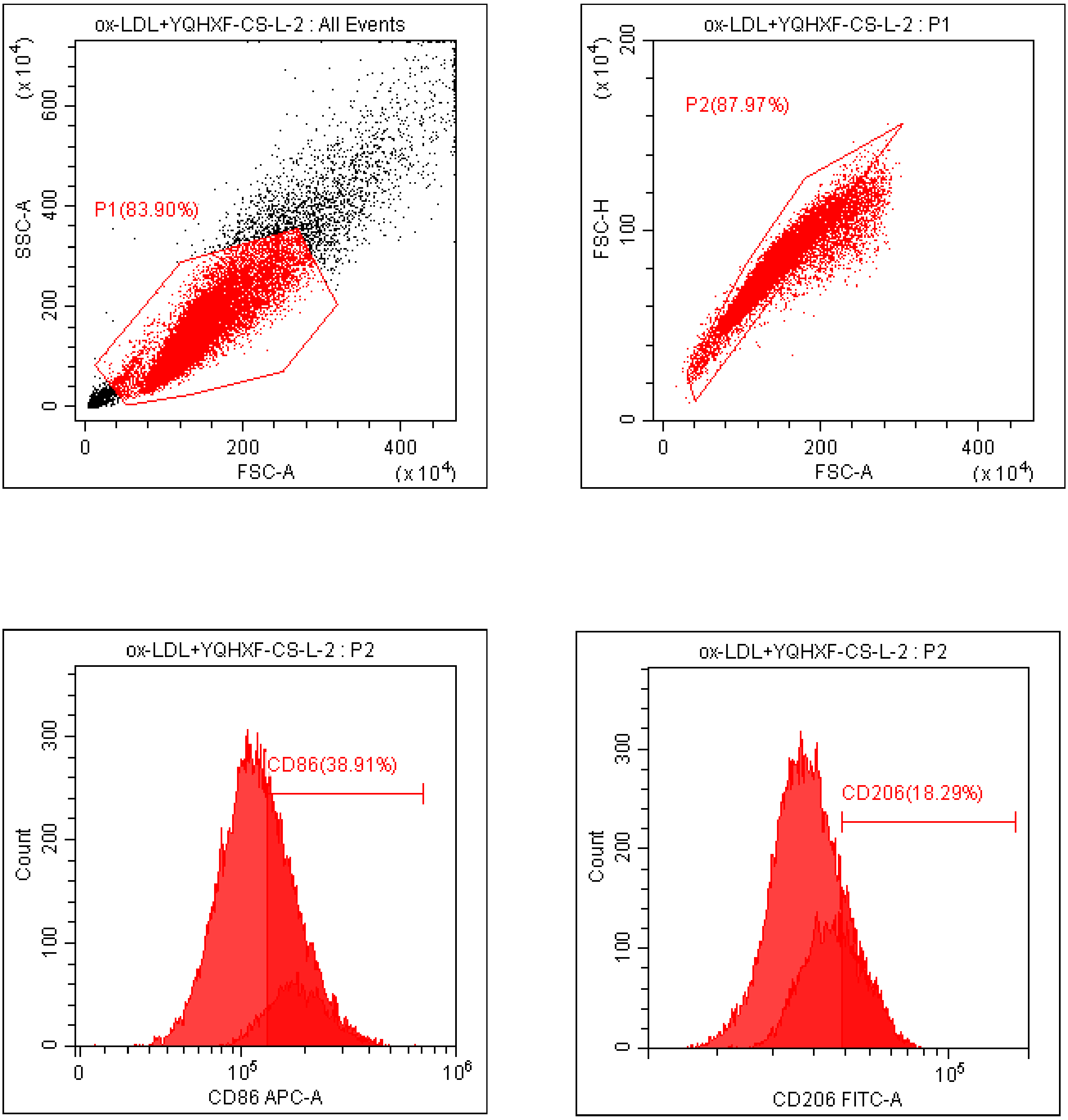

Figure S5 ox-LDL+YQHXF-CS-L-3   CD86/CD206

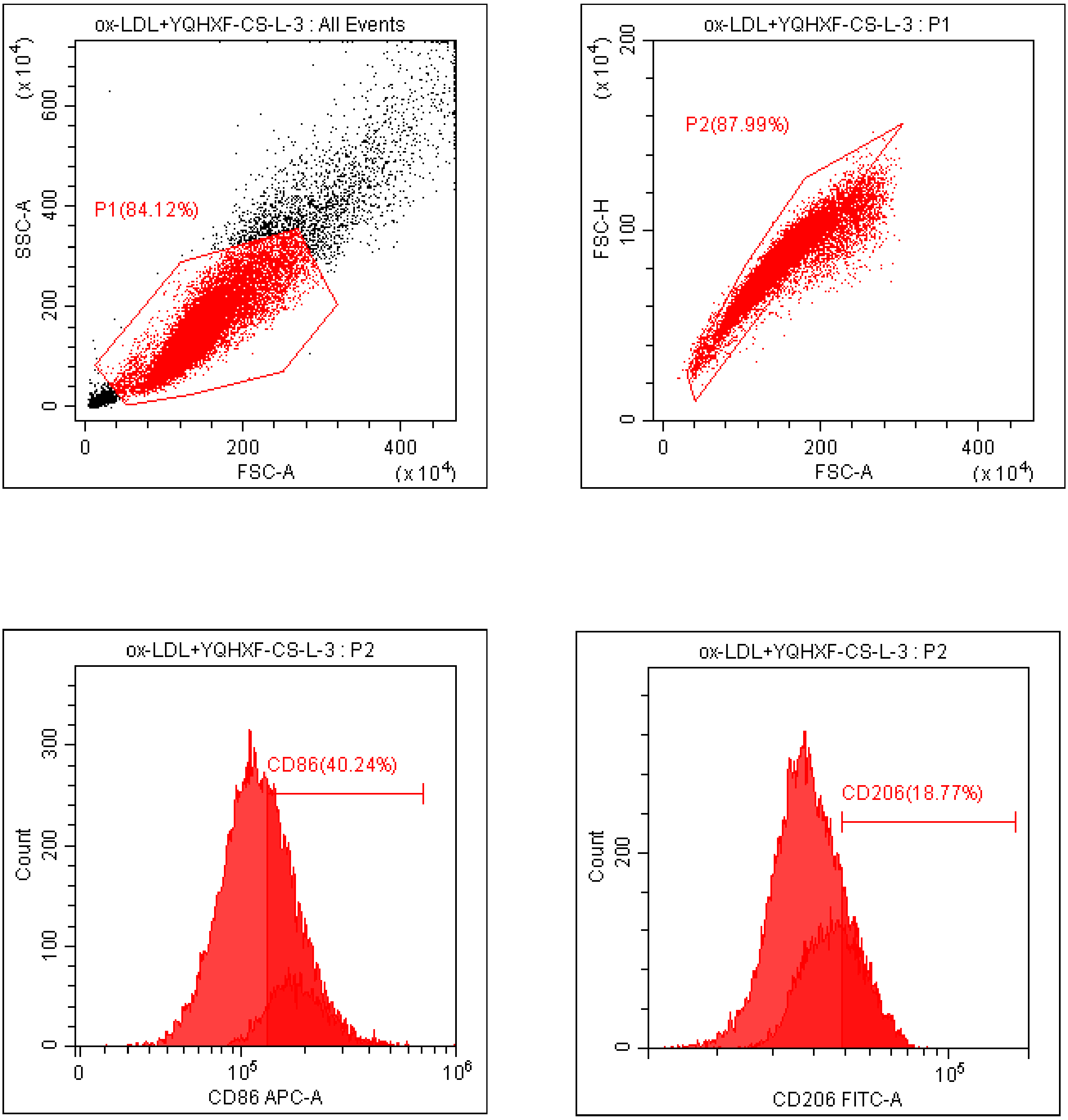

Figure S5 ox-LDL+YQHXF-CS-H-1 CD86/CD206

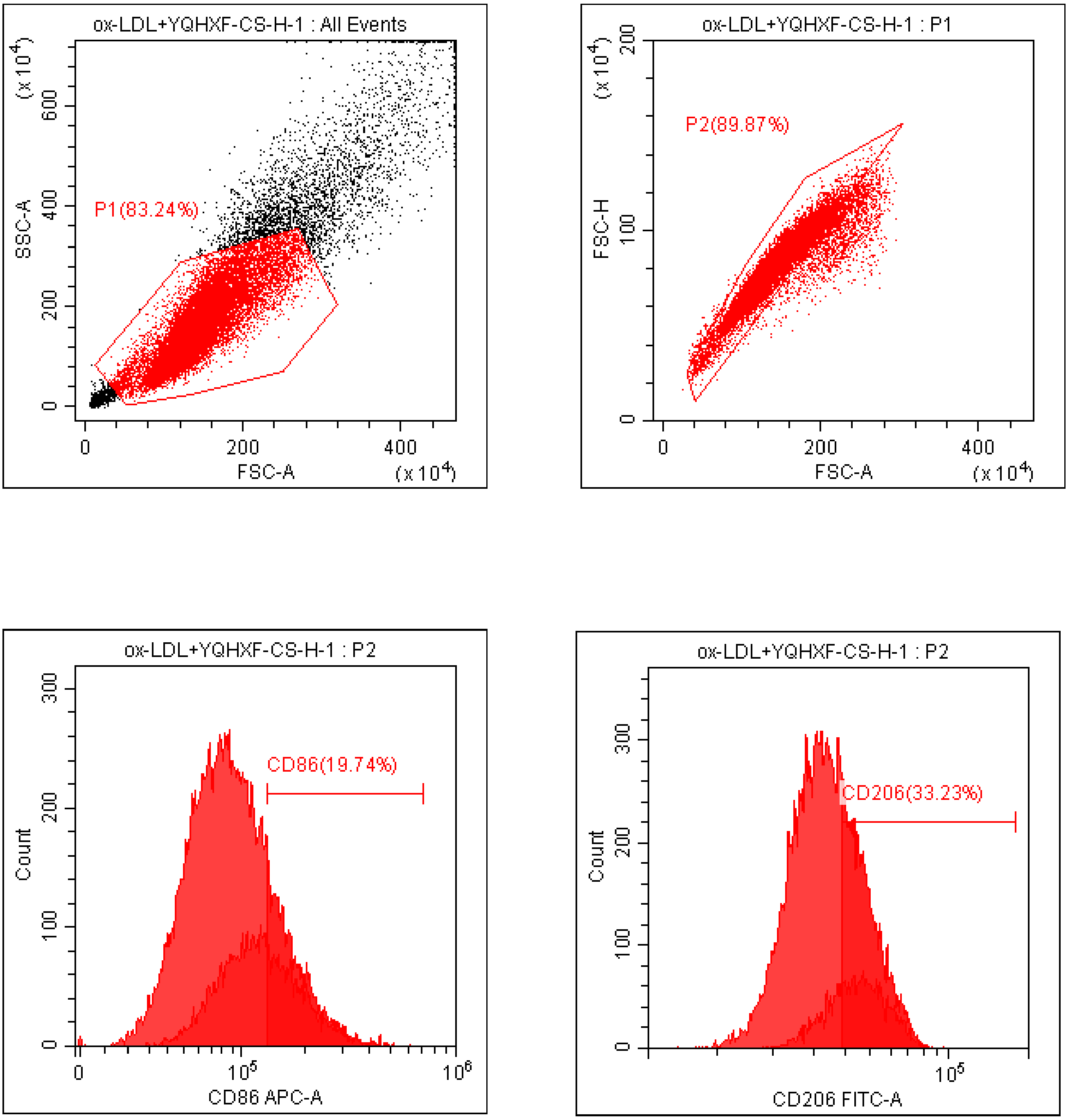

Figure S5 ox-LDL+YQHXF-CS-H-2   CD86/CD206

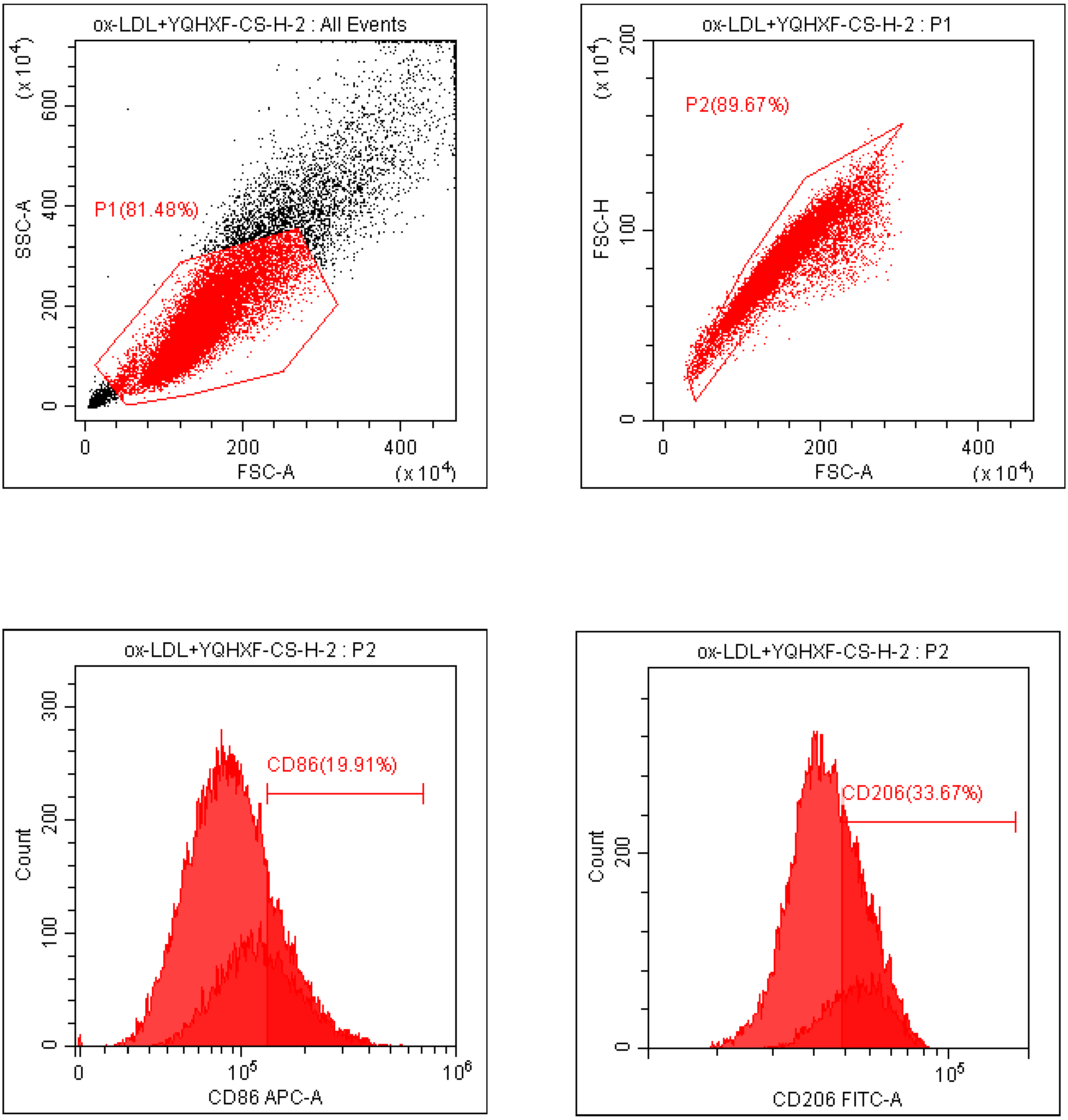

Figure S5 ox-LDL+YQHXF-CS-H-3 CD86/CD206

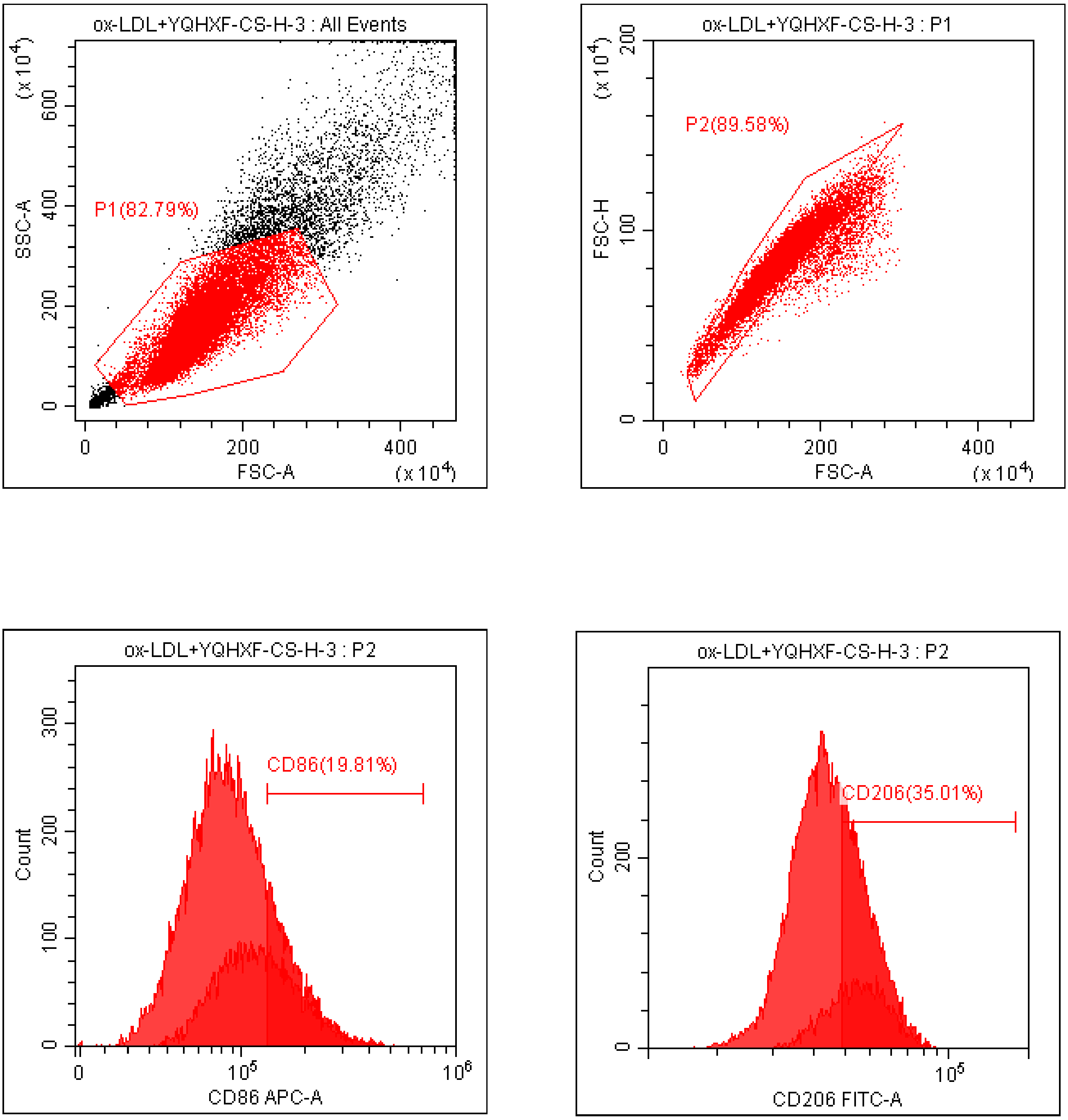

Figure S5 ox-LDL+YQHXF-CS-M-1   CD86/CD206

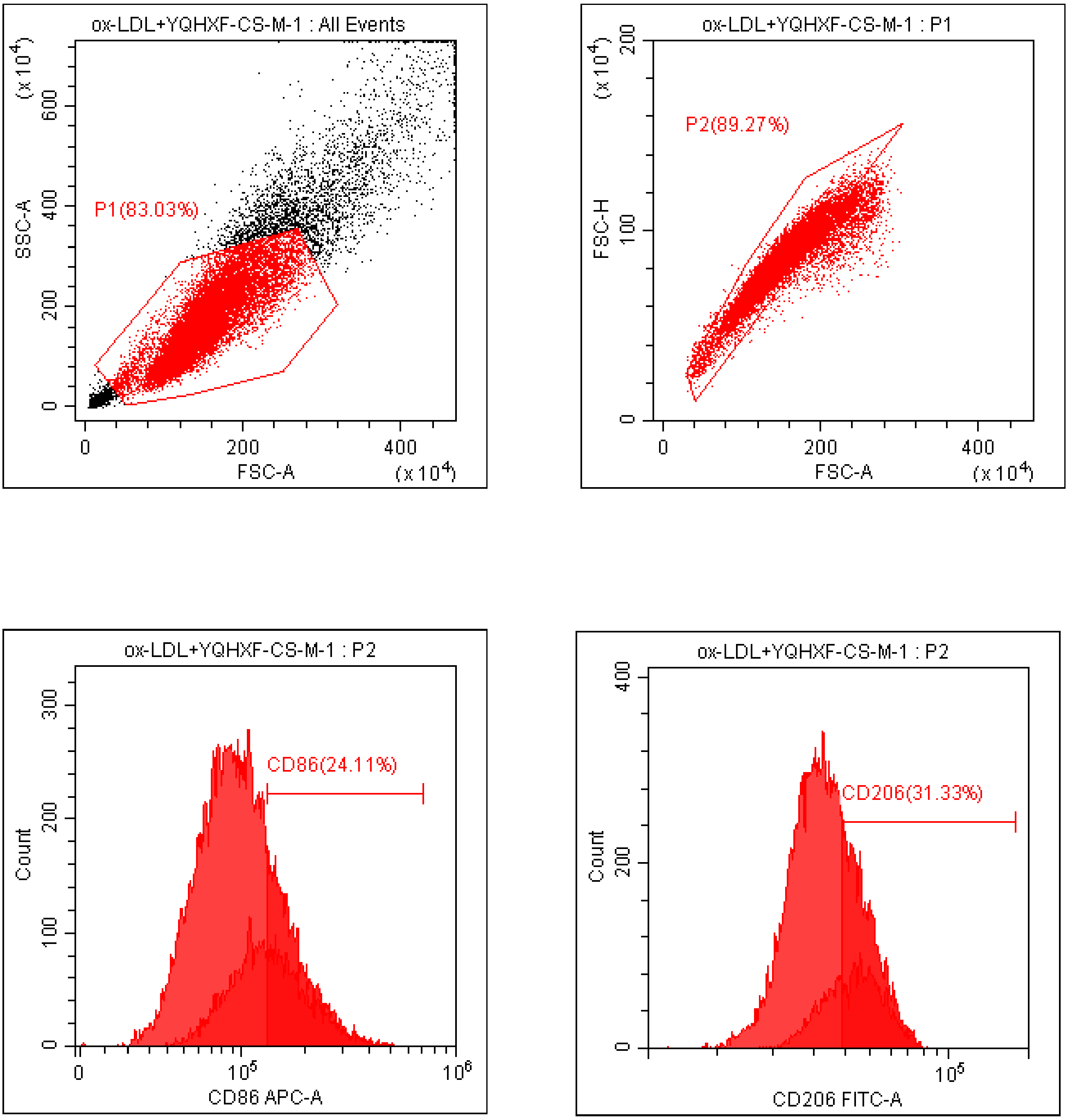

Figure S5 ox-LDL+YQHXF-CS-M-2 CD86/CD206

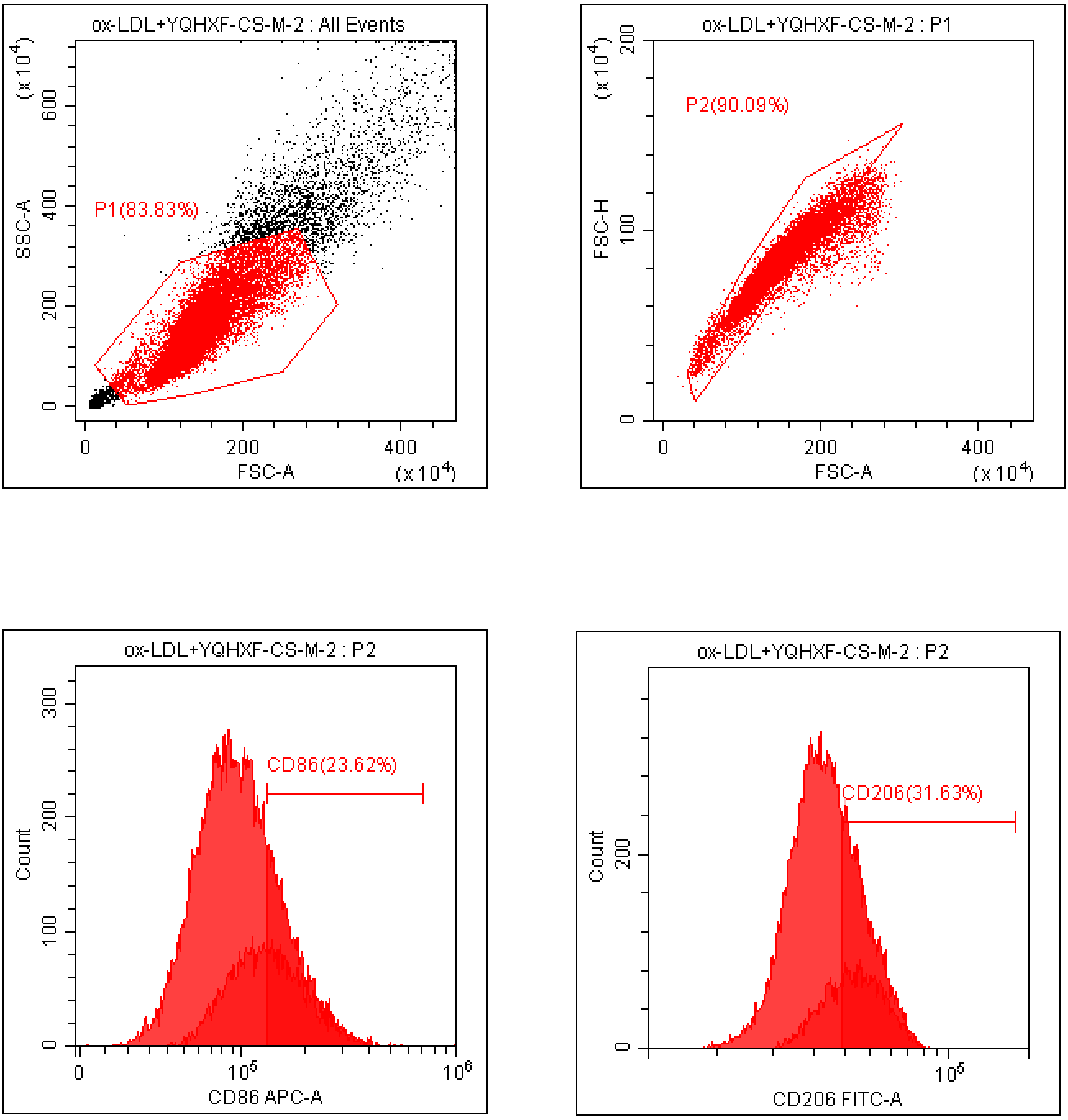

Figure S5 ox-LDL+YQHXF-CS-M-3 CD86/CD206

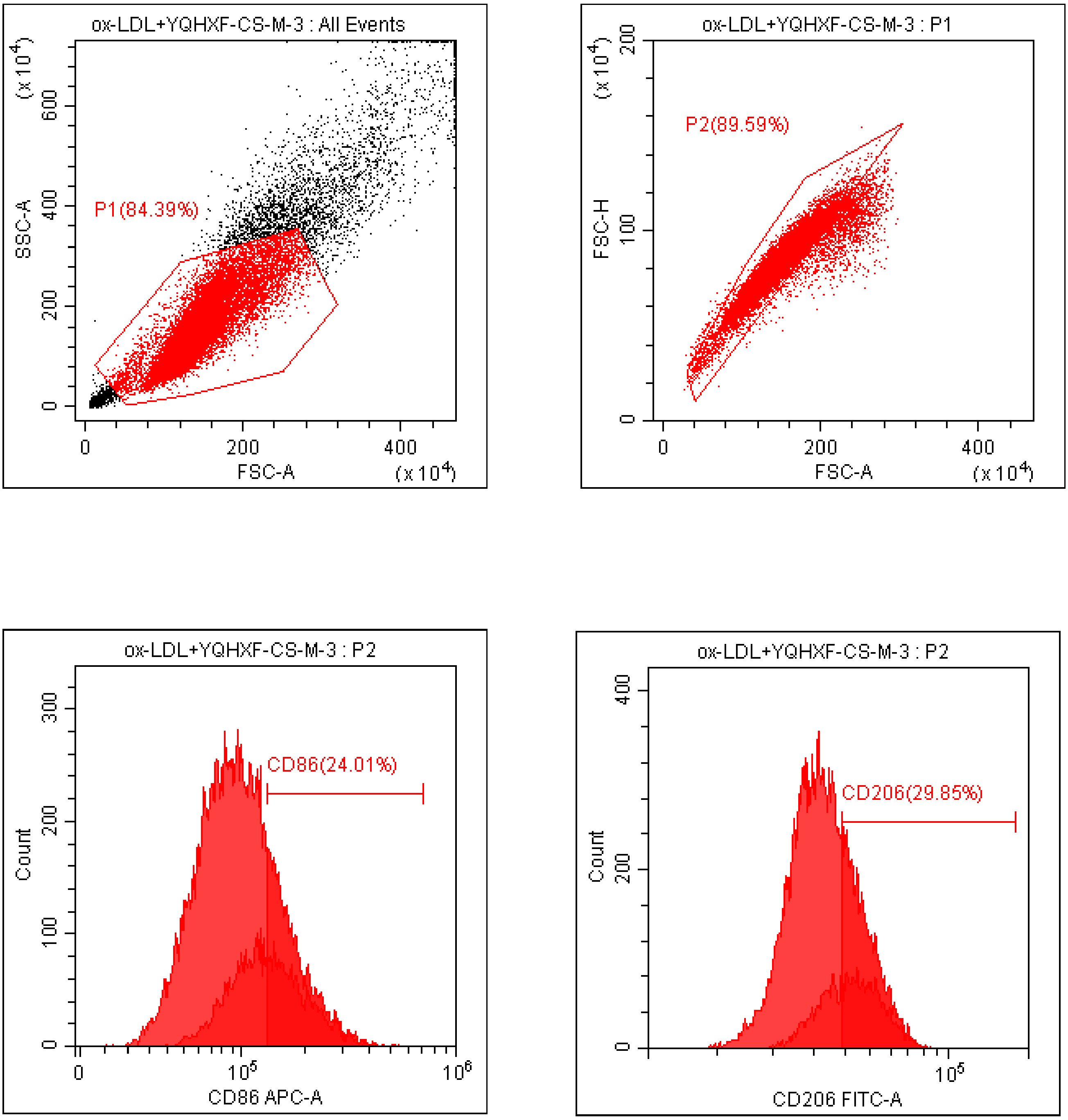

(3) The raw data of flow cytometry in Figure S6

Figure S6 BLANK CD80/CD163

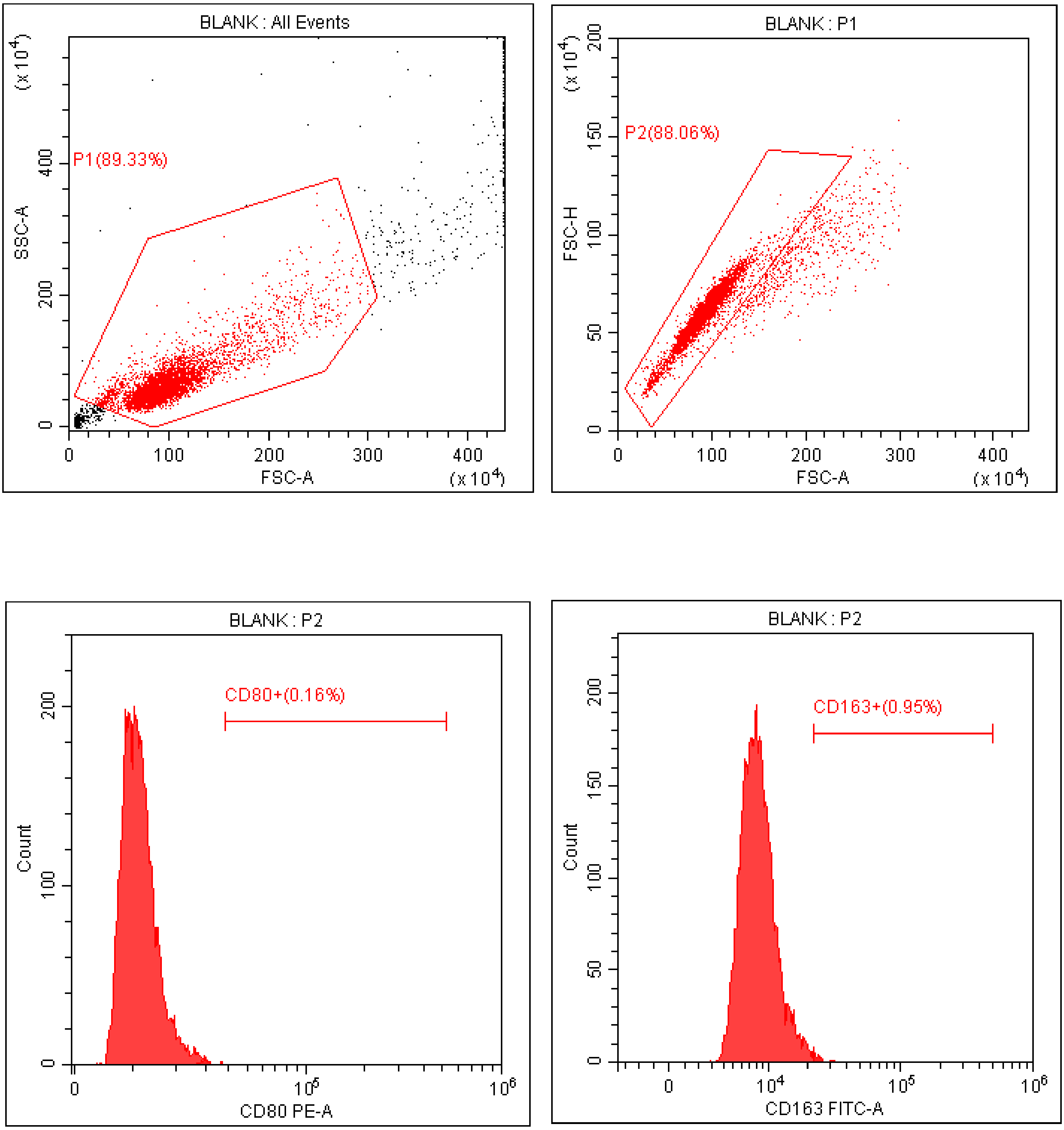

Figure S6 CD80

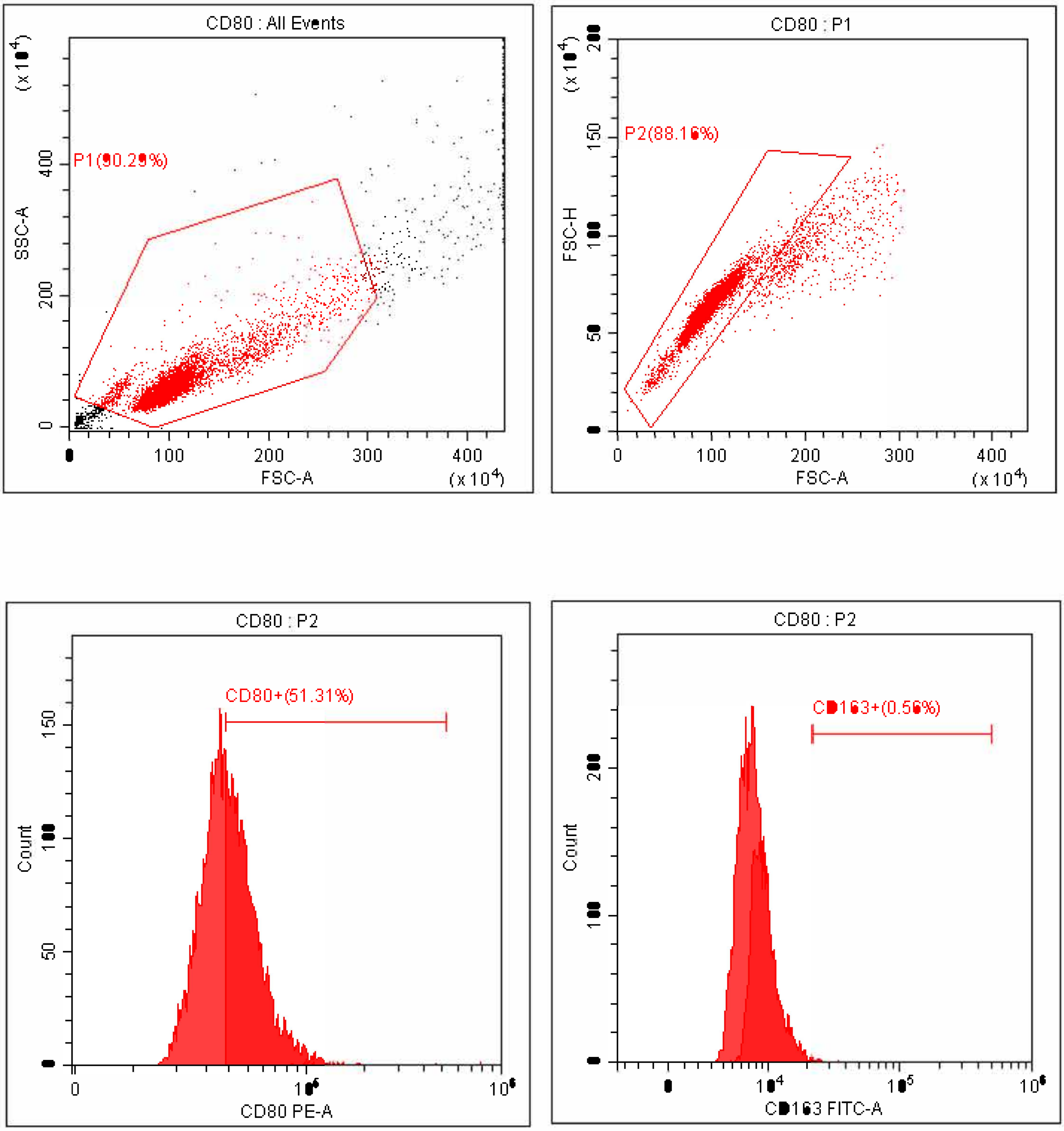

Figure S6 CD163

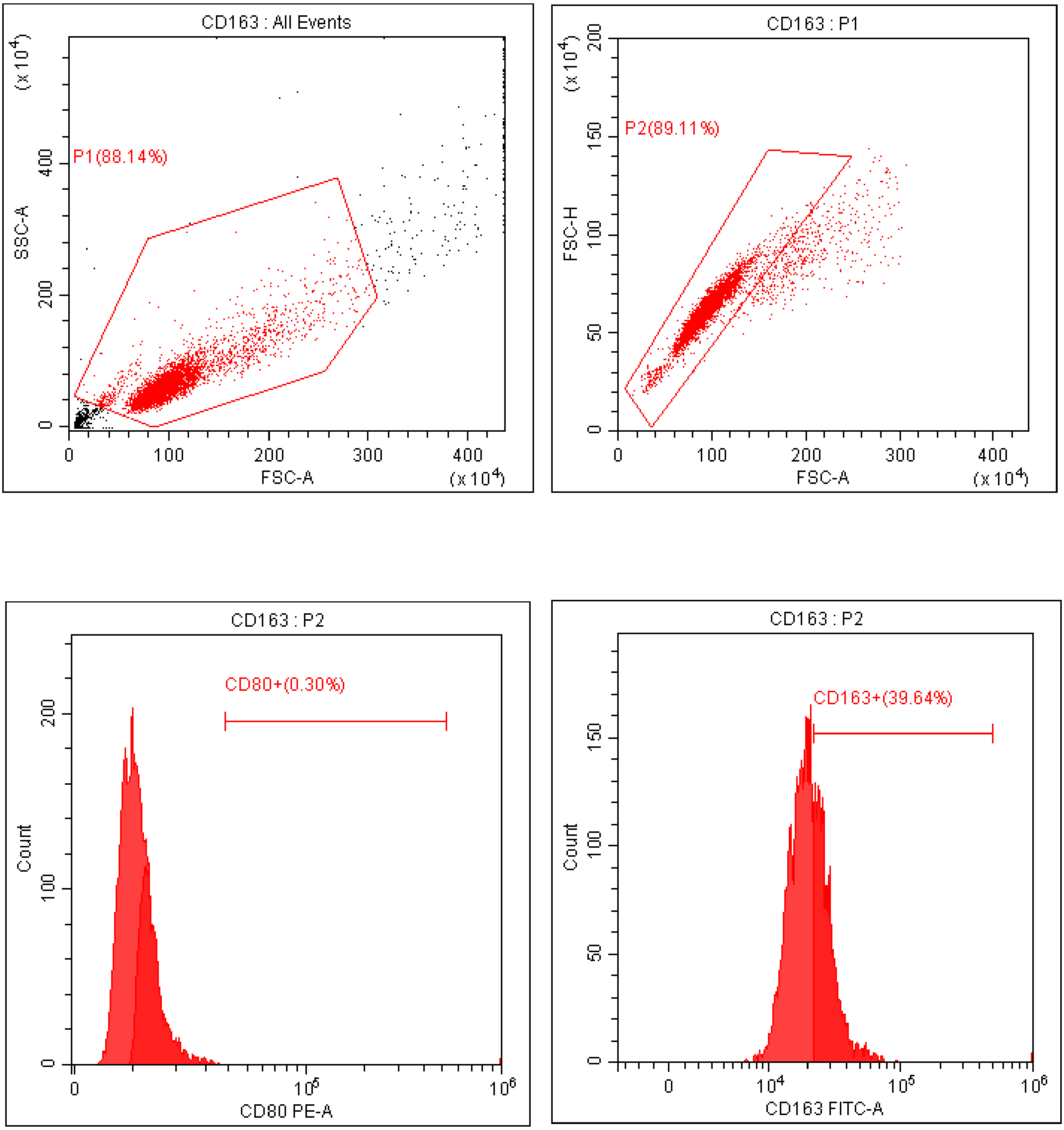

Figure S6 siRNA-PPAR γ +ox-LDL+BS-1 CD80/CD163

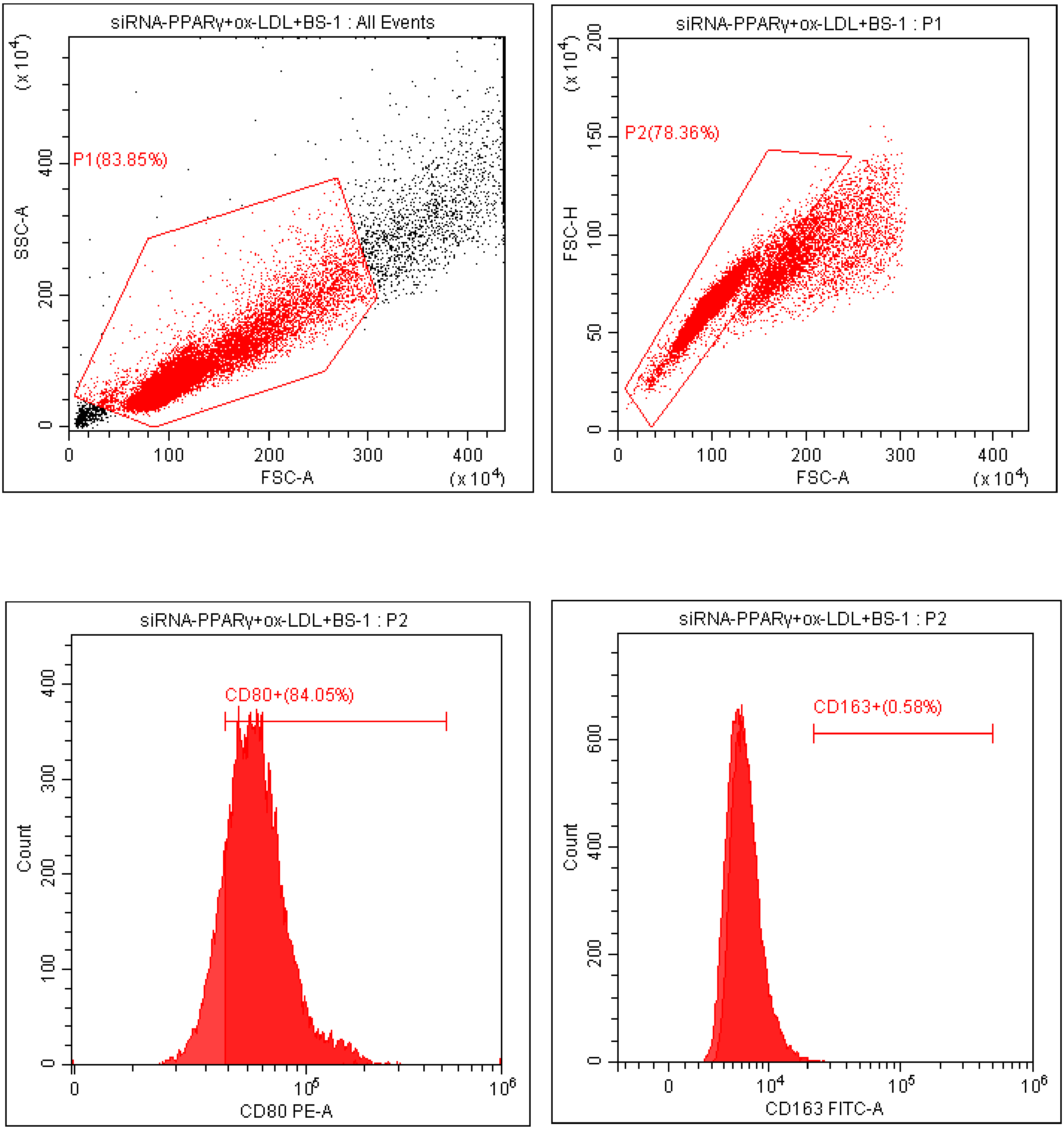

Figure S6 siRNA-PPAR γ +ox-LDL+BS-2 CD80/CD163

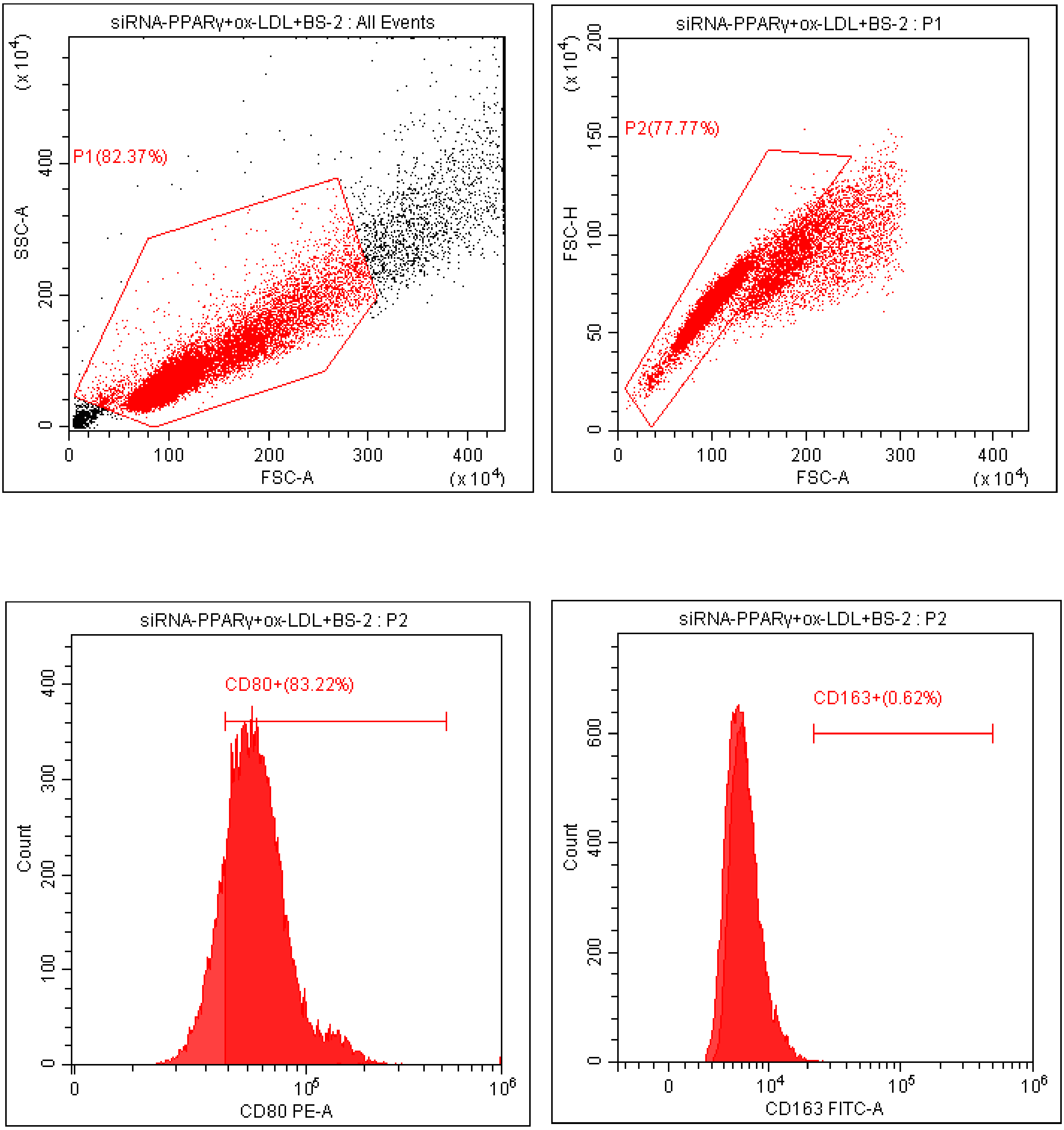

Figure S6 siRNA-PPAR γ +ox-LDL+BS-3 CD80/CD163

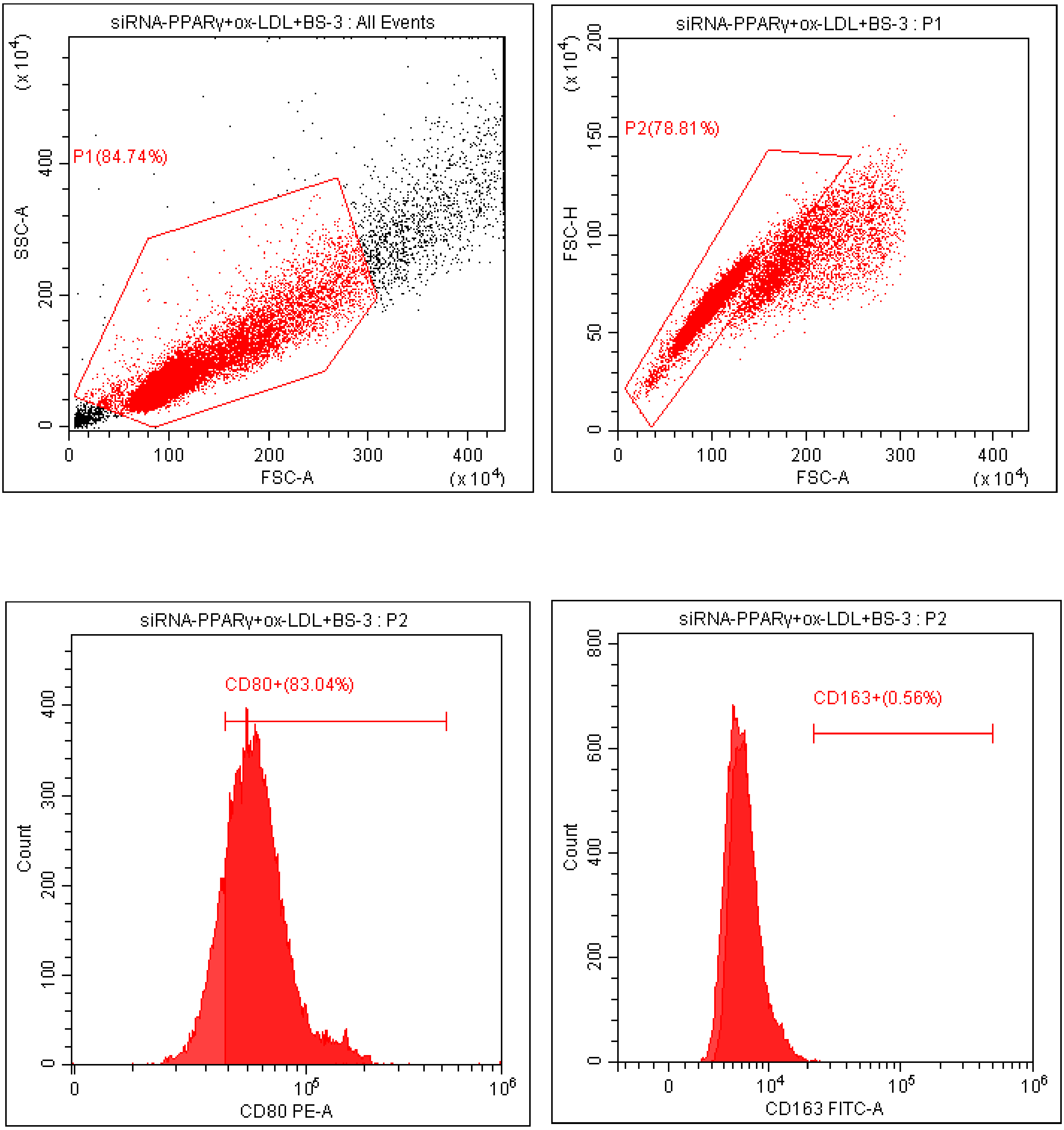

Figure S6 siRNA-PPAR γ -1 CD80/CD163

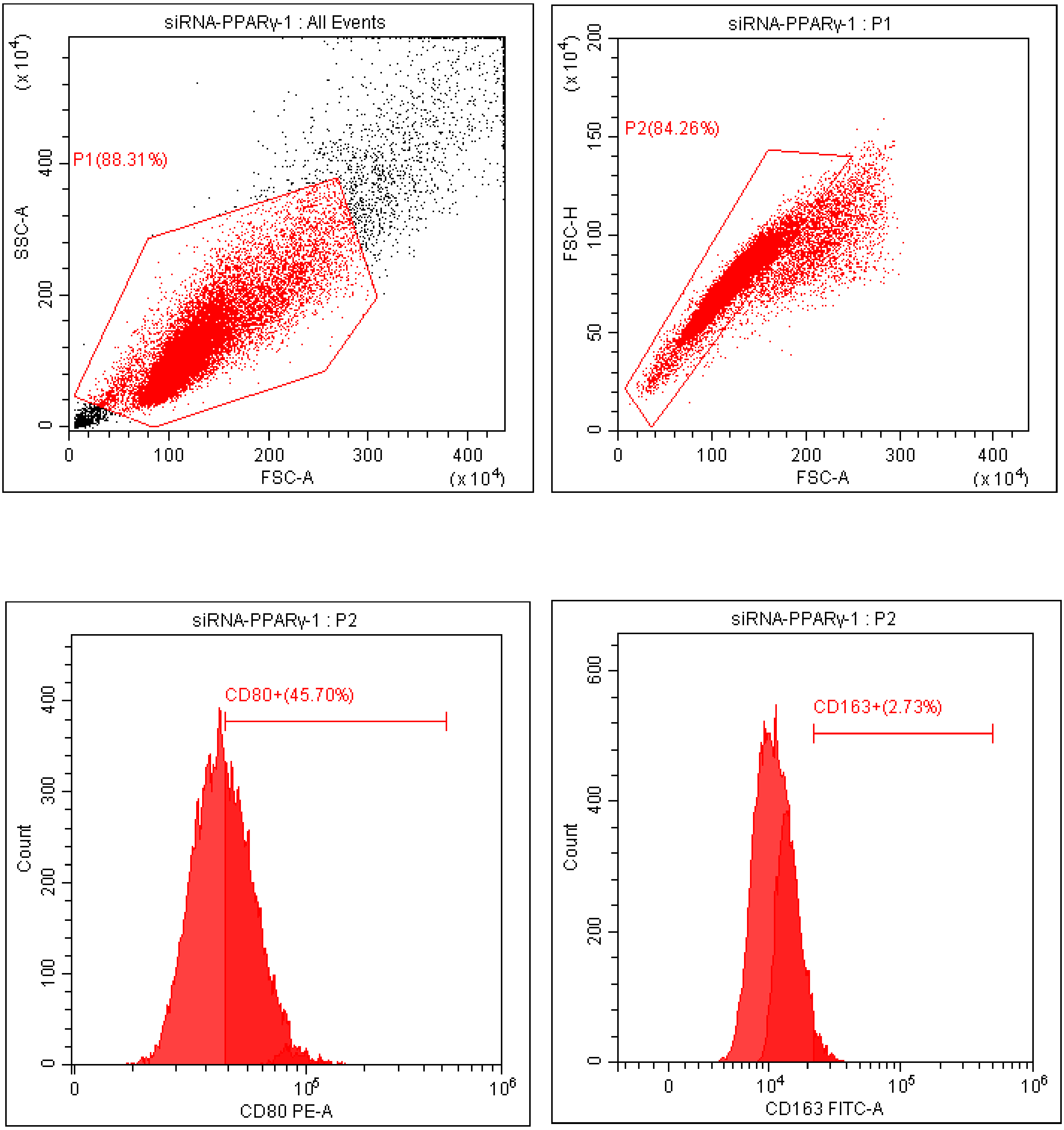

Figure S6 siRNA-PPAR γ -2 CD80/CD163

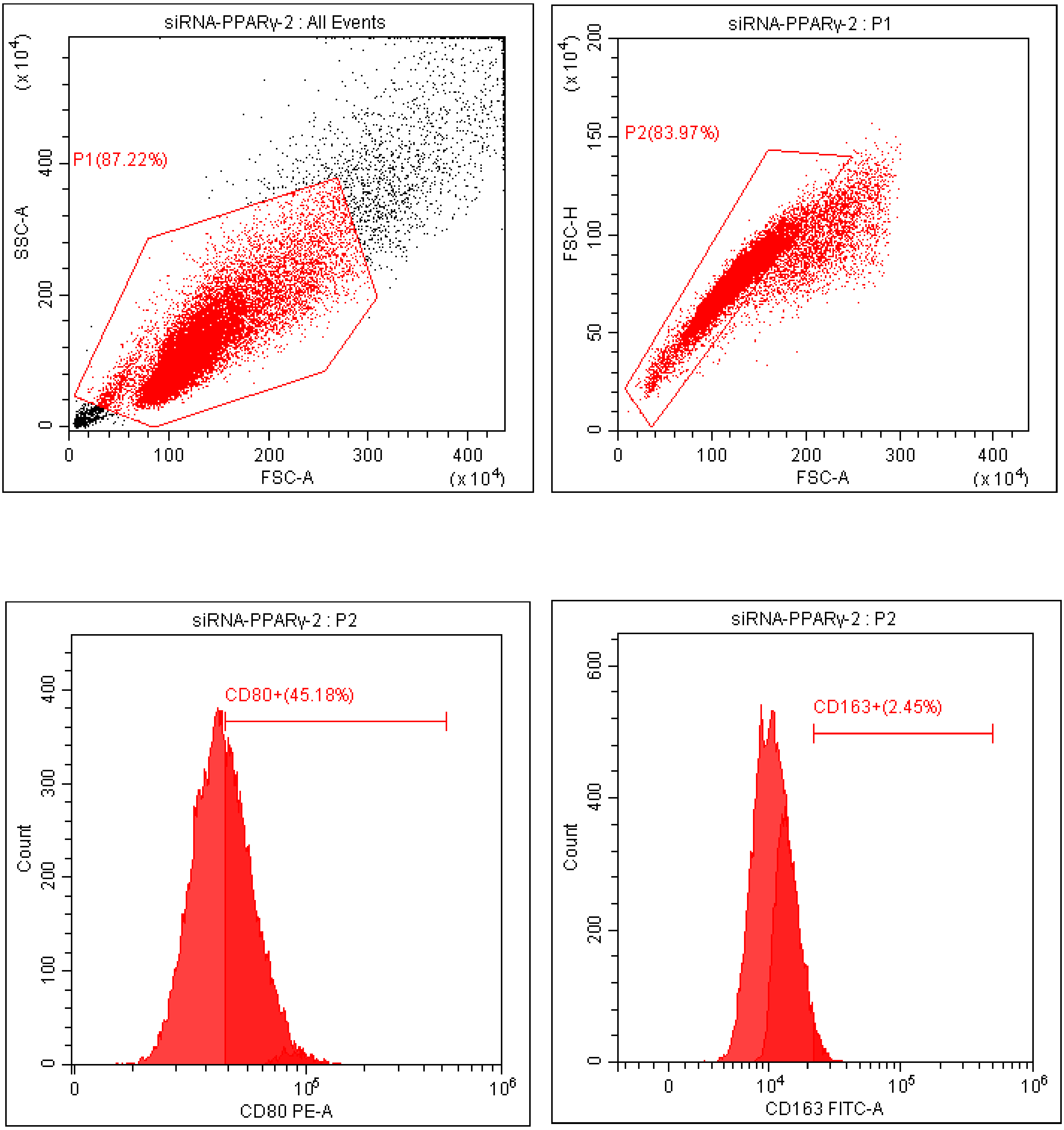

Figure S6 siRNA-PPAR γ -3 CD80/CD163

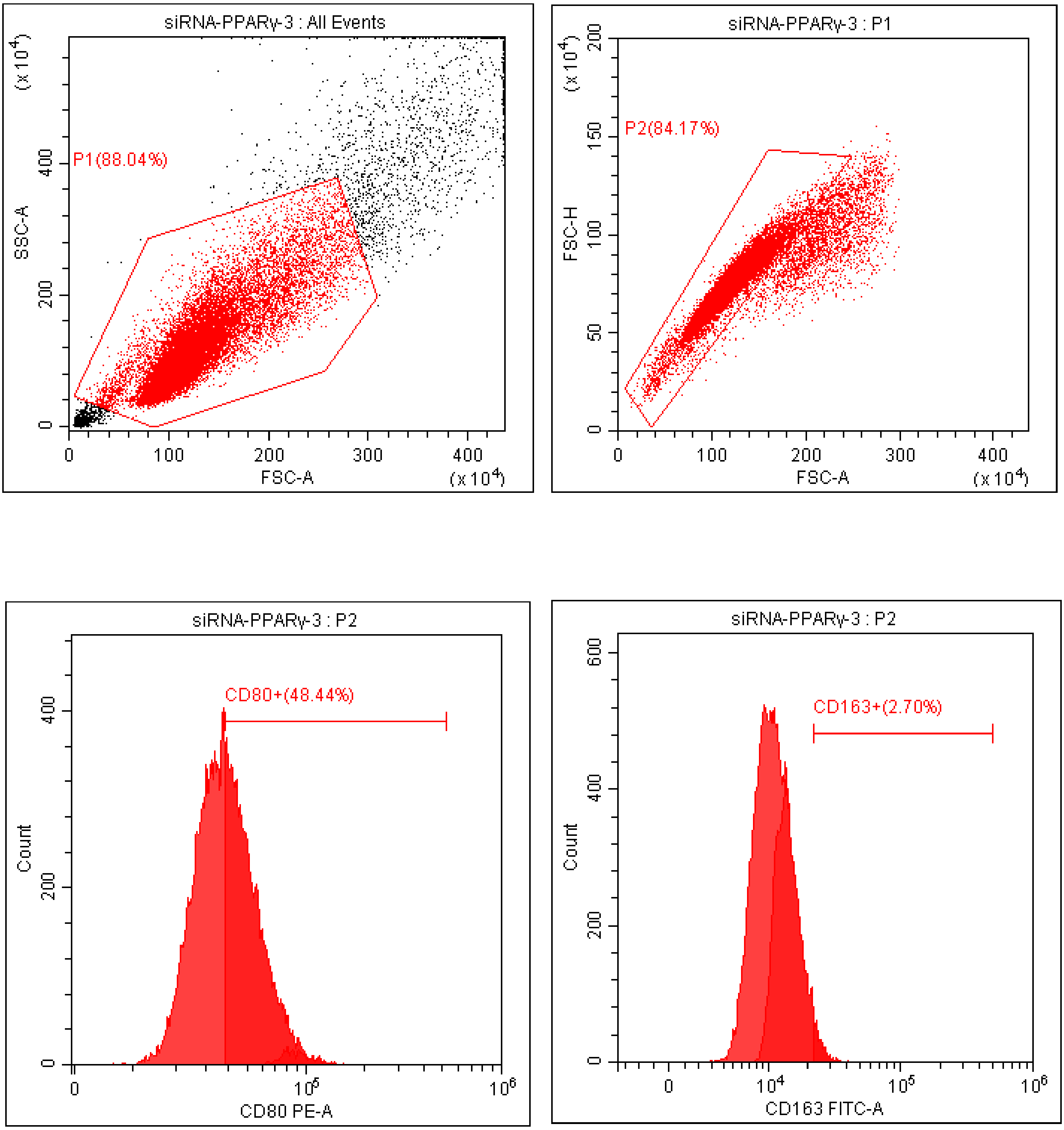

Figure S6 siRNA-PPAR γ +ox-LDL+YQHXF-CS-1 CD80/CD163

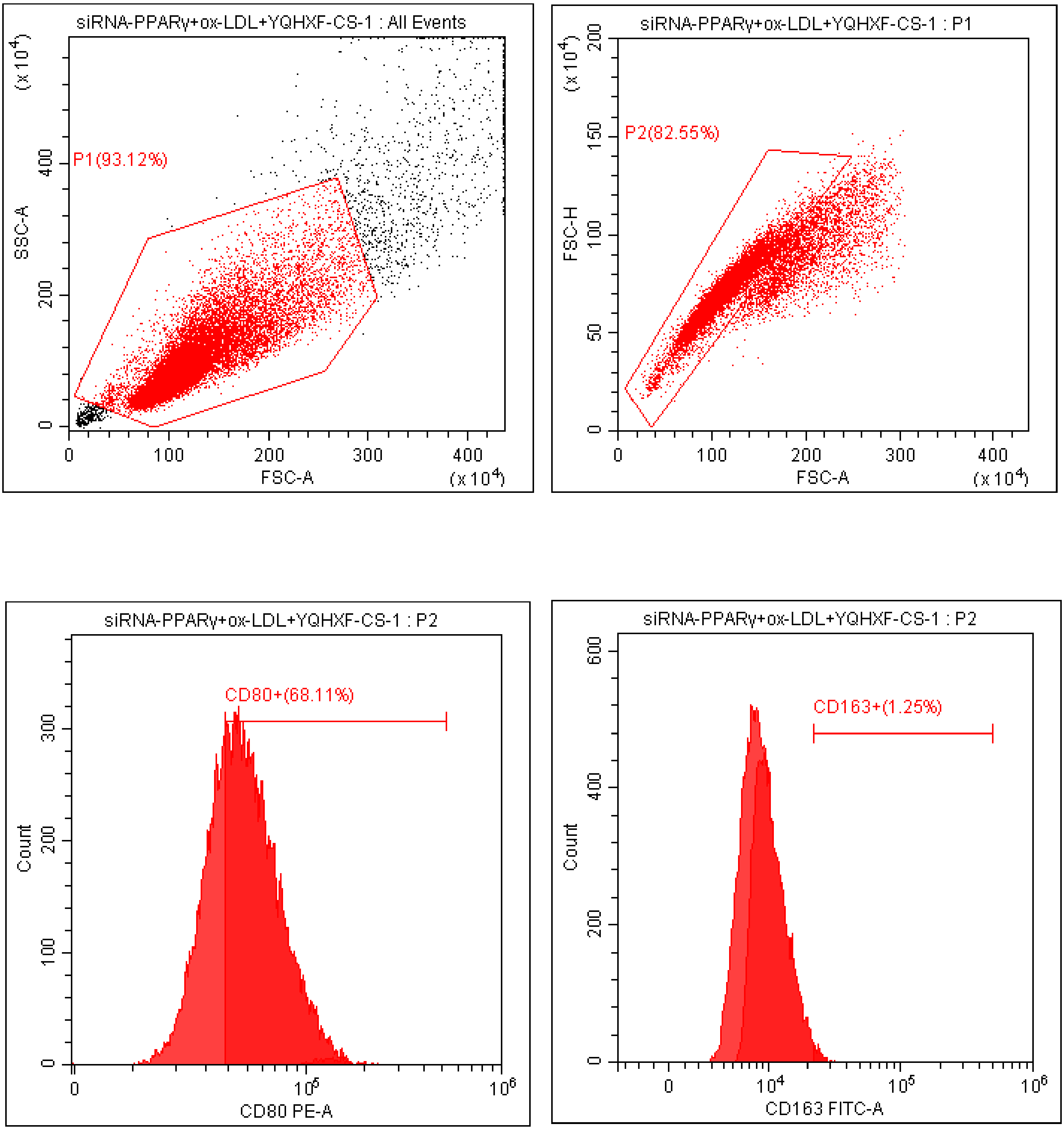

Figure S6 siRNA-PPAR γ +ox-LDL+YQHXF-CS-2 CD80/CD163

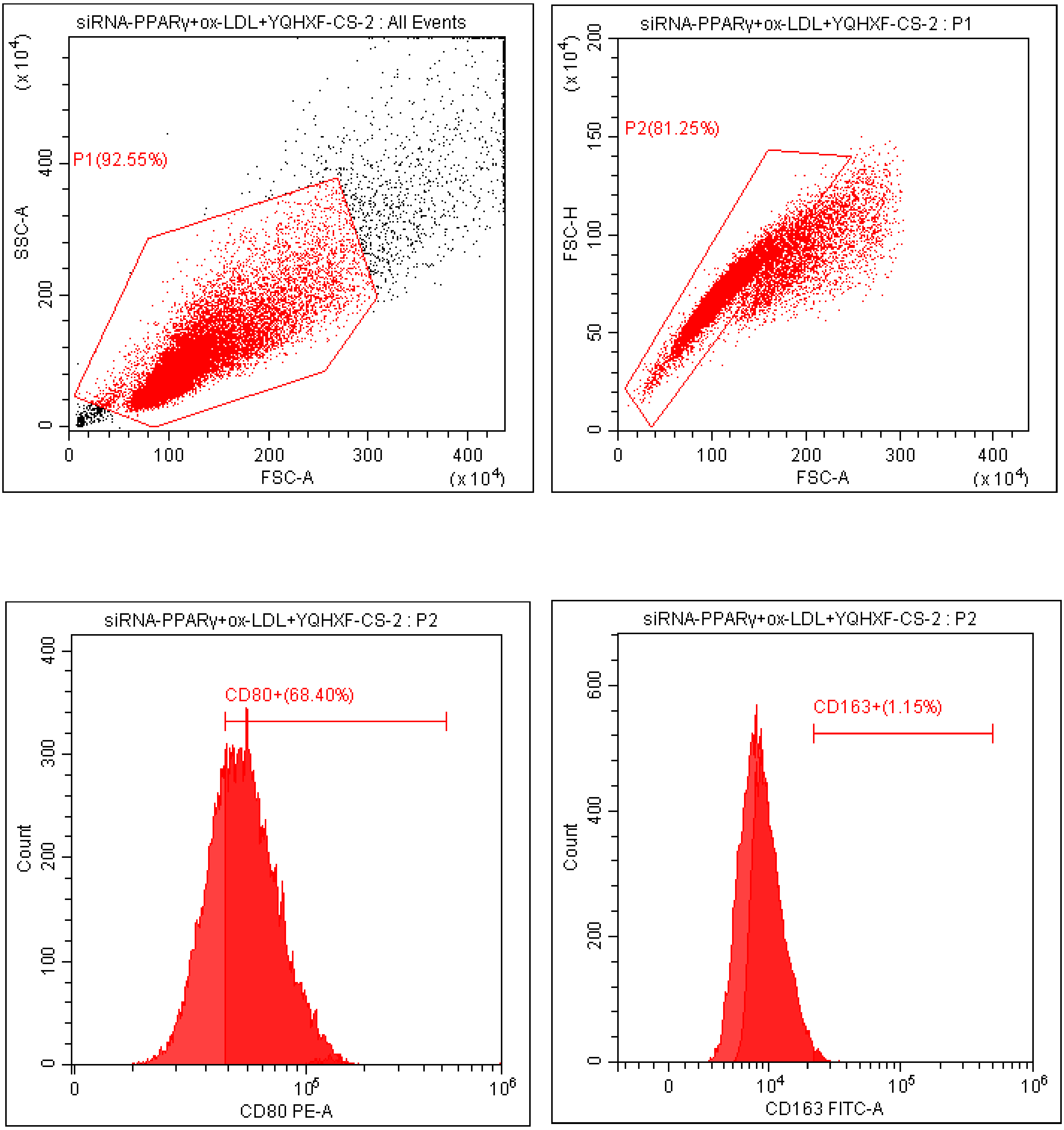

Figure S6 siRNA-PPAR γ +ox-LDL+YQHXF-CS-3 CD80/CD163

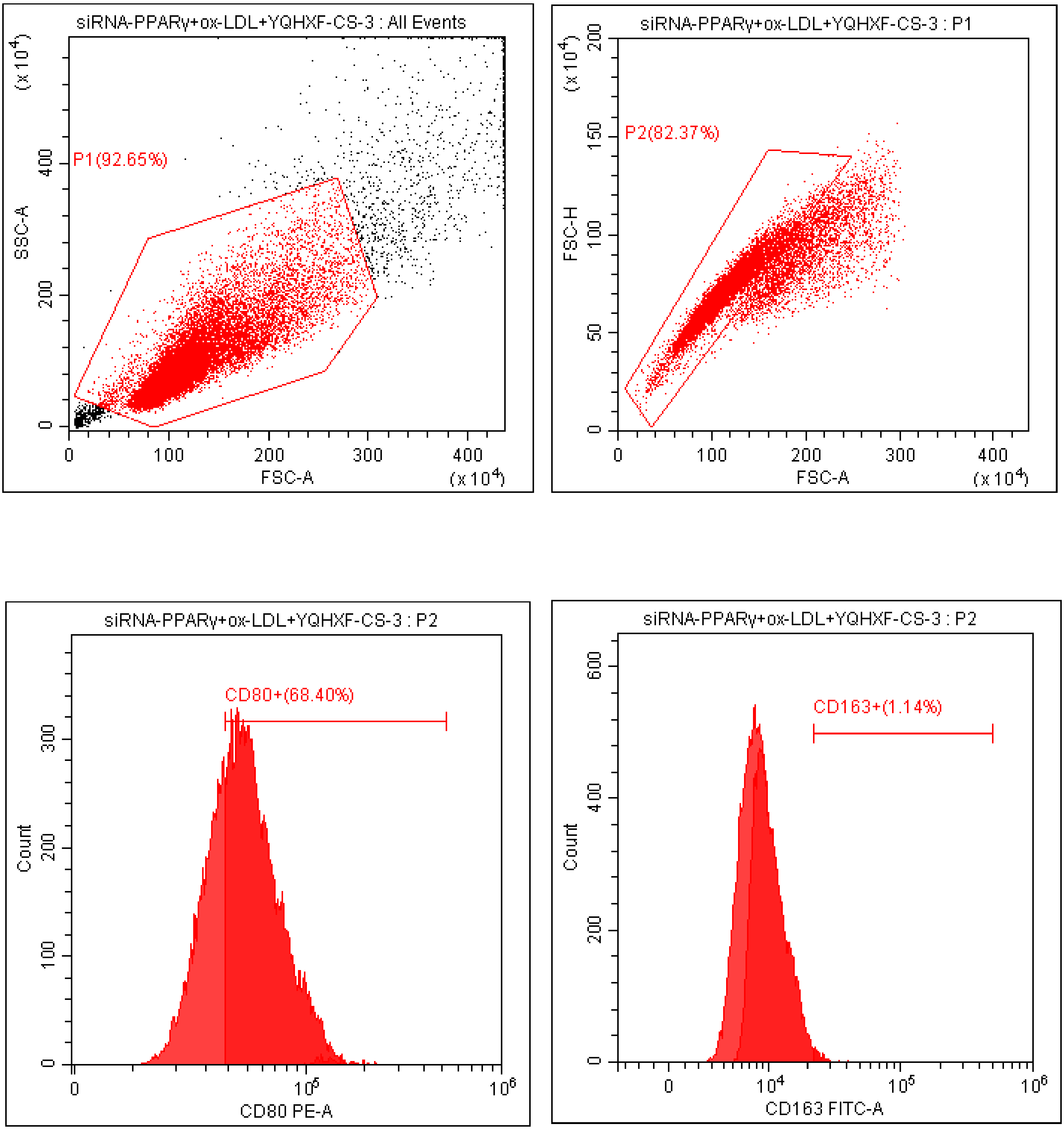

Figure S6 siRNA-NC+ox-LDL+BS-1 CD80/CD163

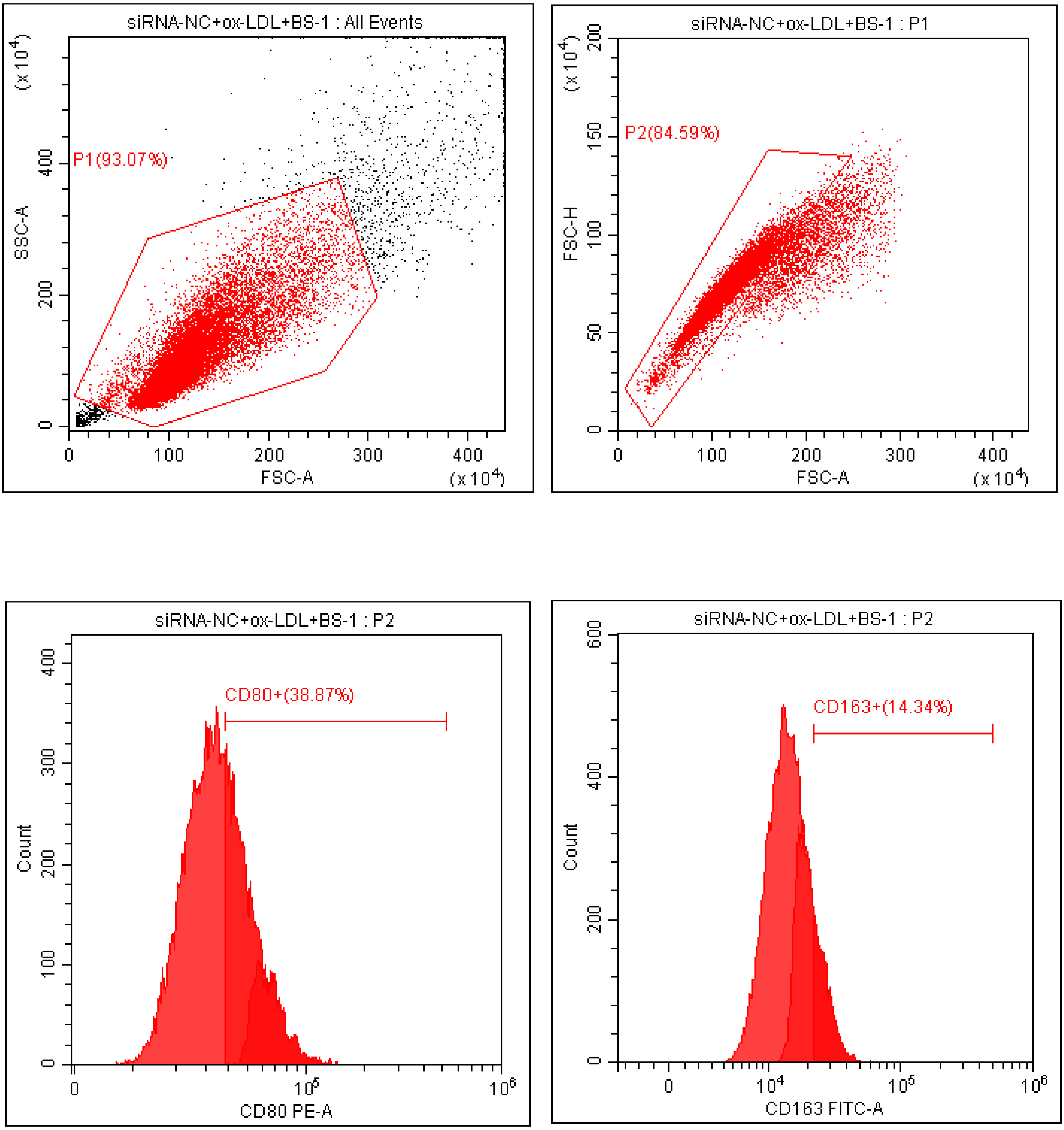

Figure S6 siRNA-NC+ox-LDL+BS-2 CD80/CD163

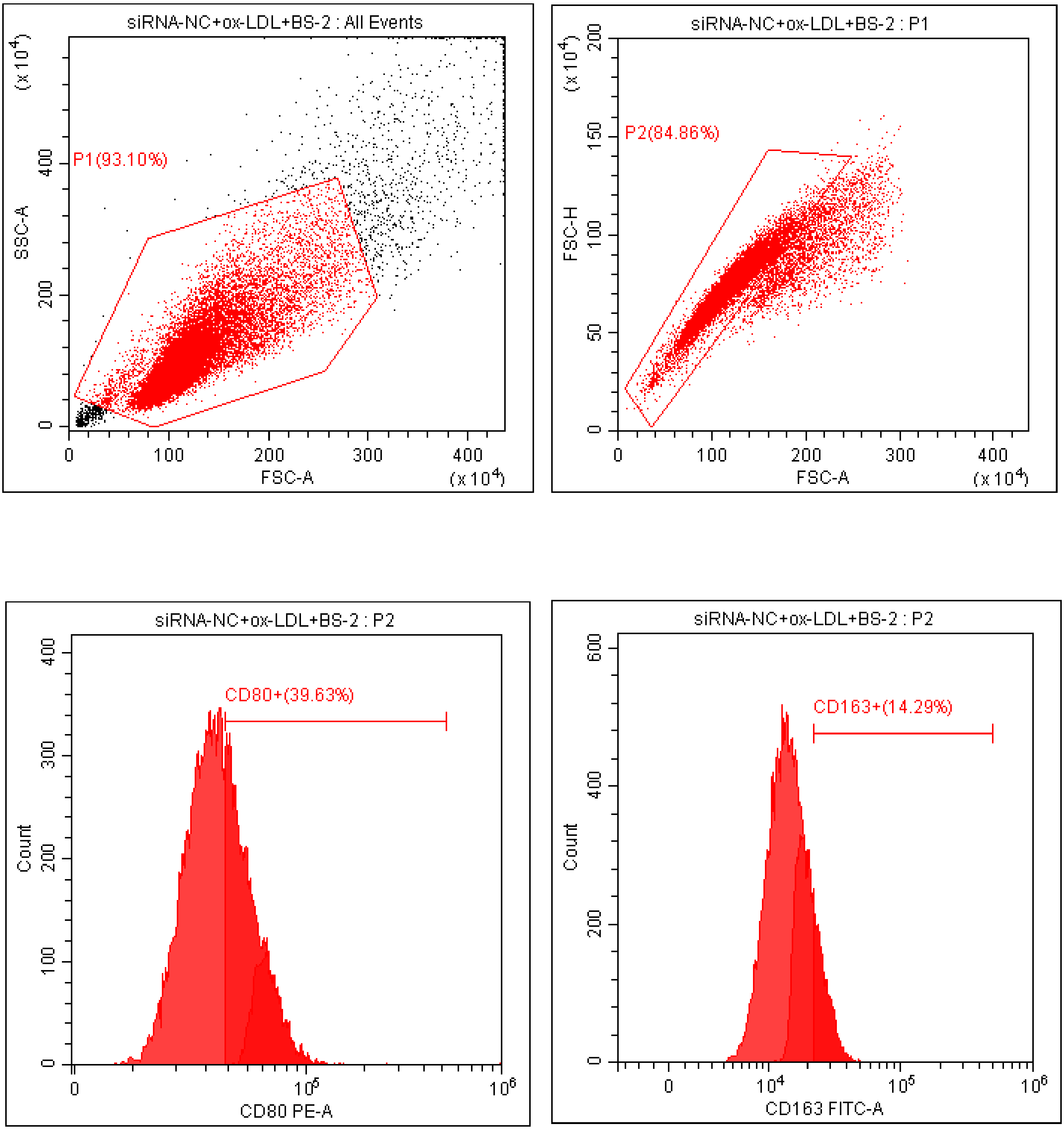

Figure S6 siRNA-NC+ox-LDL+BS-3 CD80/CD163

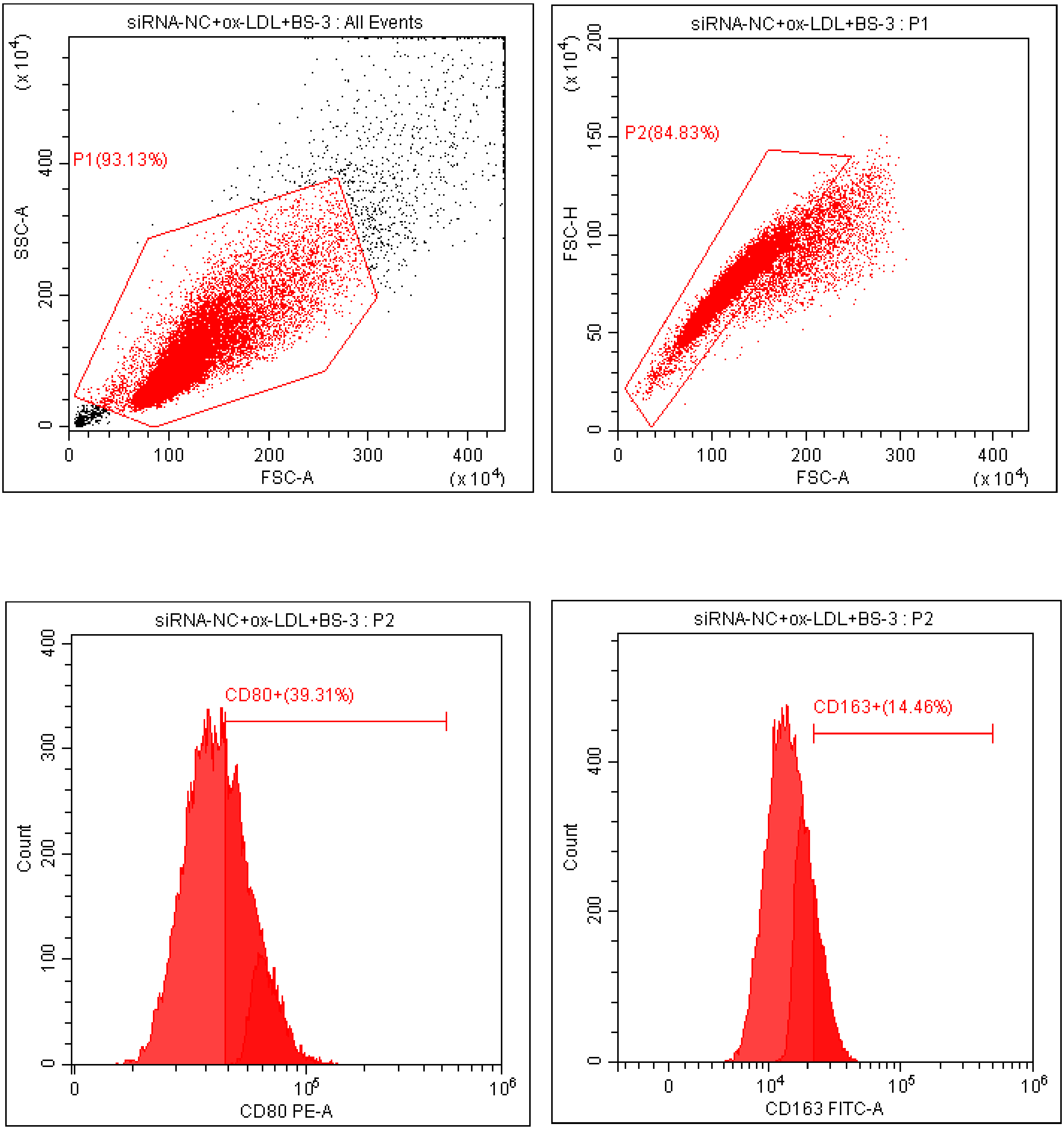

Figure S6 siRNA-NC+ox-LDL+YQHXF-CS-1 CD80/CD163

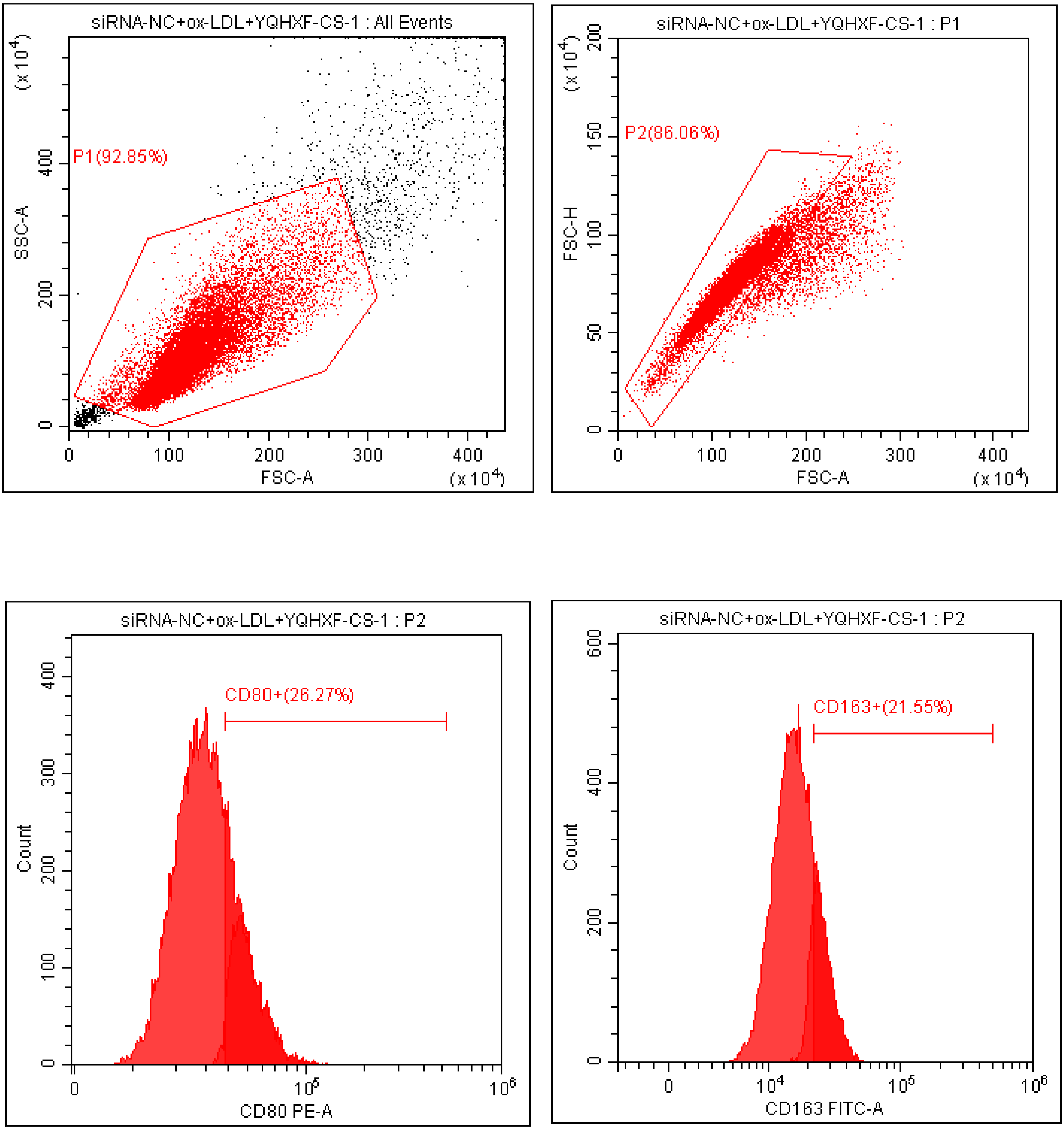

Figure S6 siRNA-NC+ox-LDL+YQHXF-CS-2 CD80/CD163

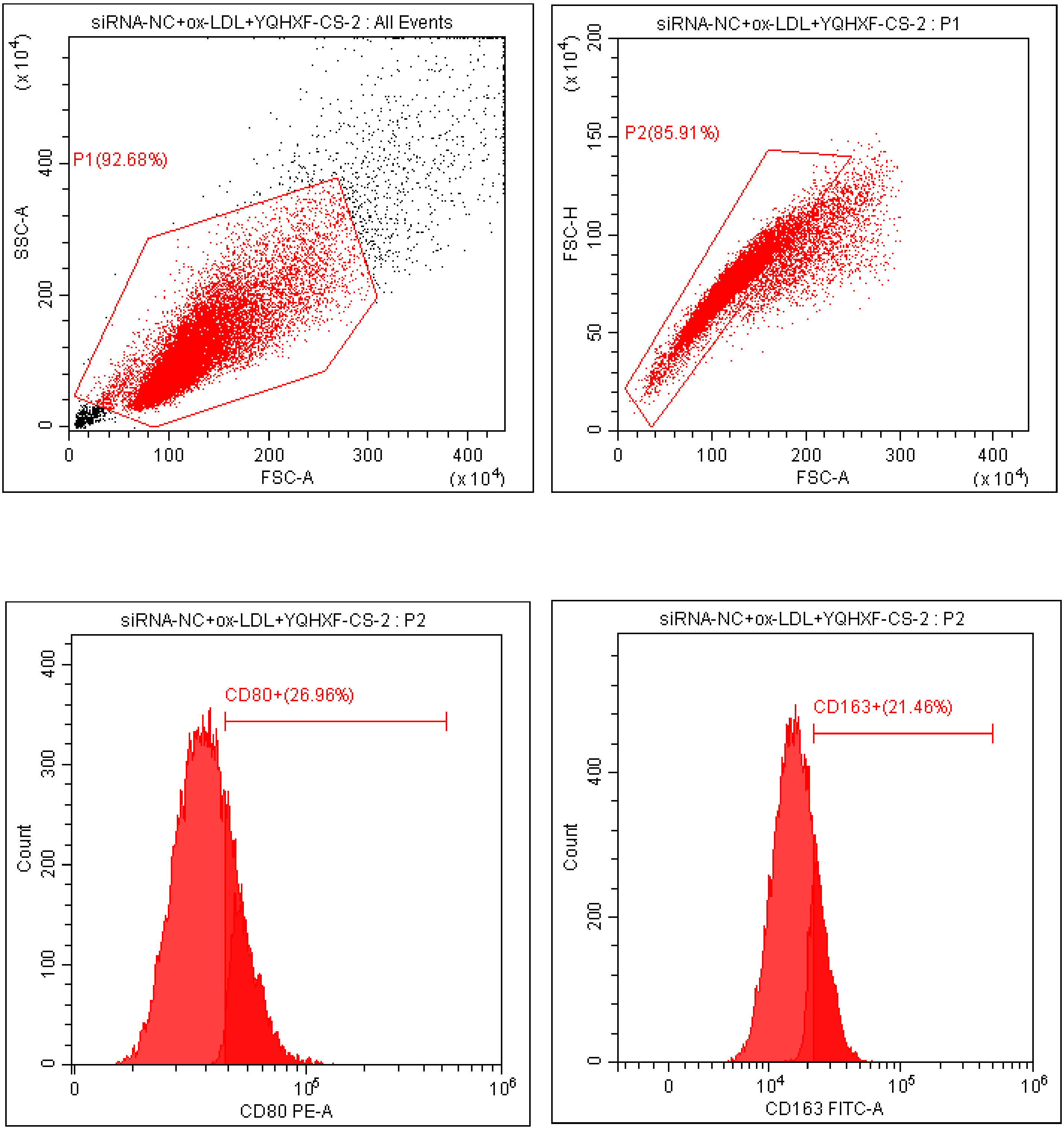

Figure S6 siRNA-NC+ox-LDL+YQHXF-CS-3 CD80/CD163

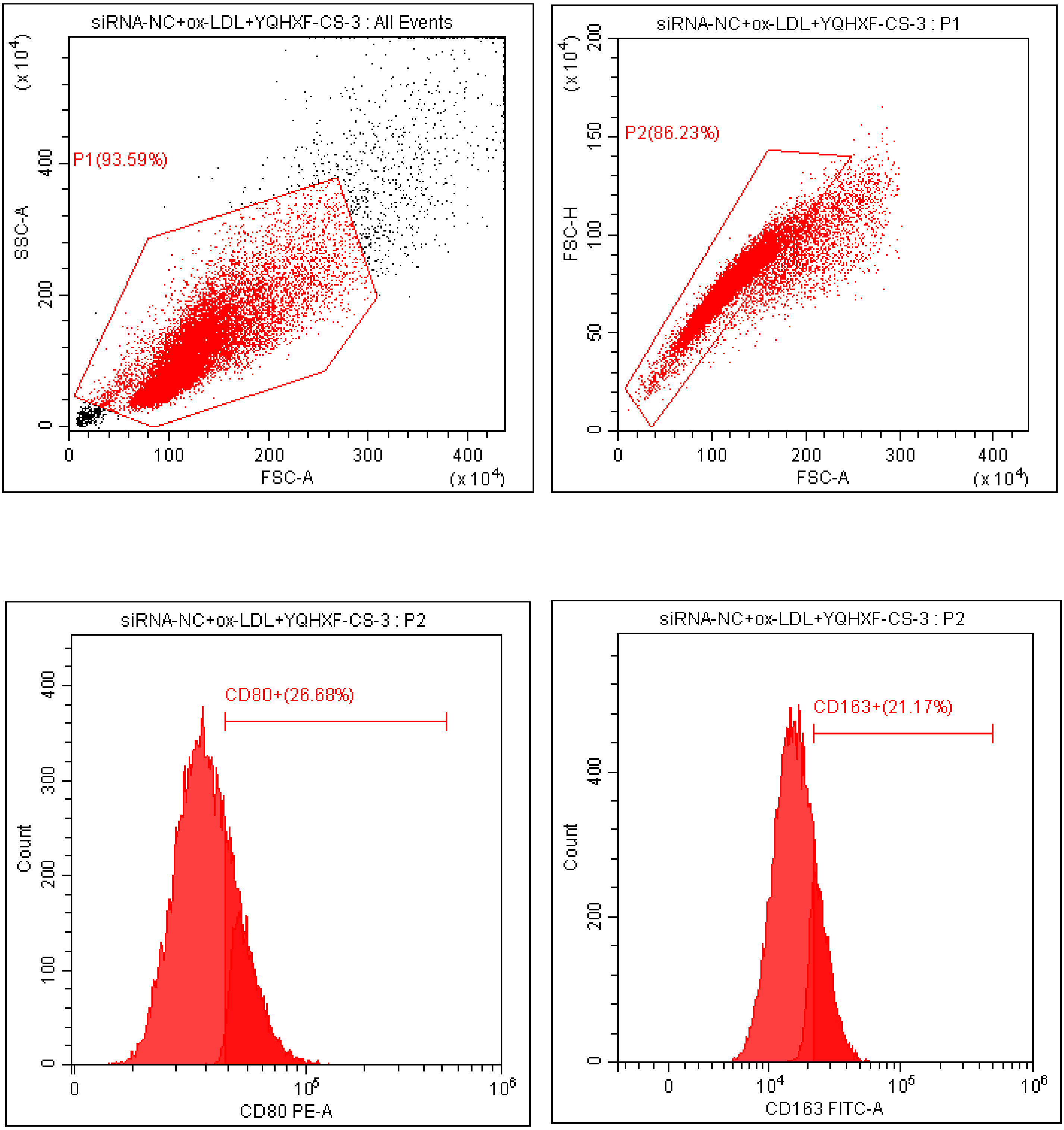

Figure S6 siRNA-NC-1 CD80/CD163

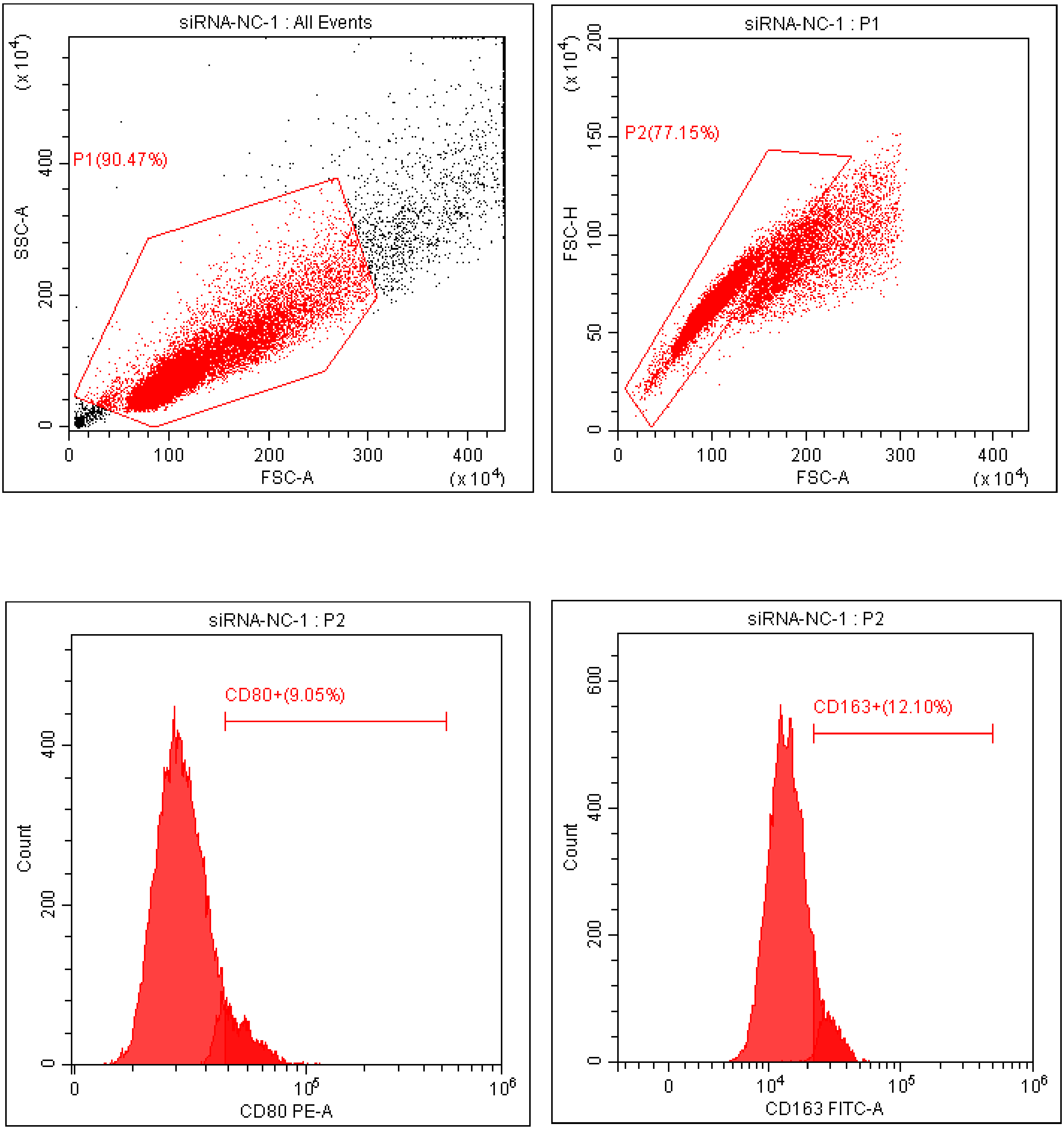

Figure S6 siRNA-NC-2 CD80/CD163

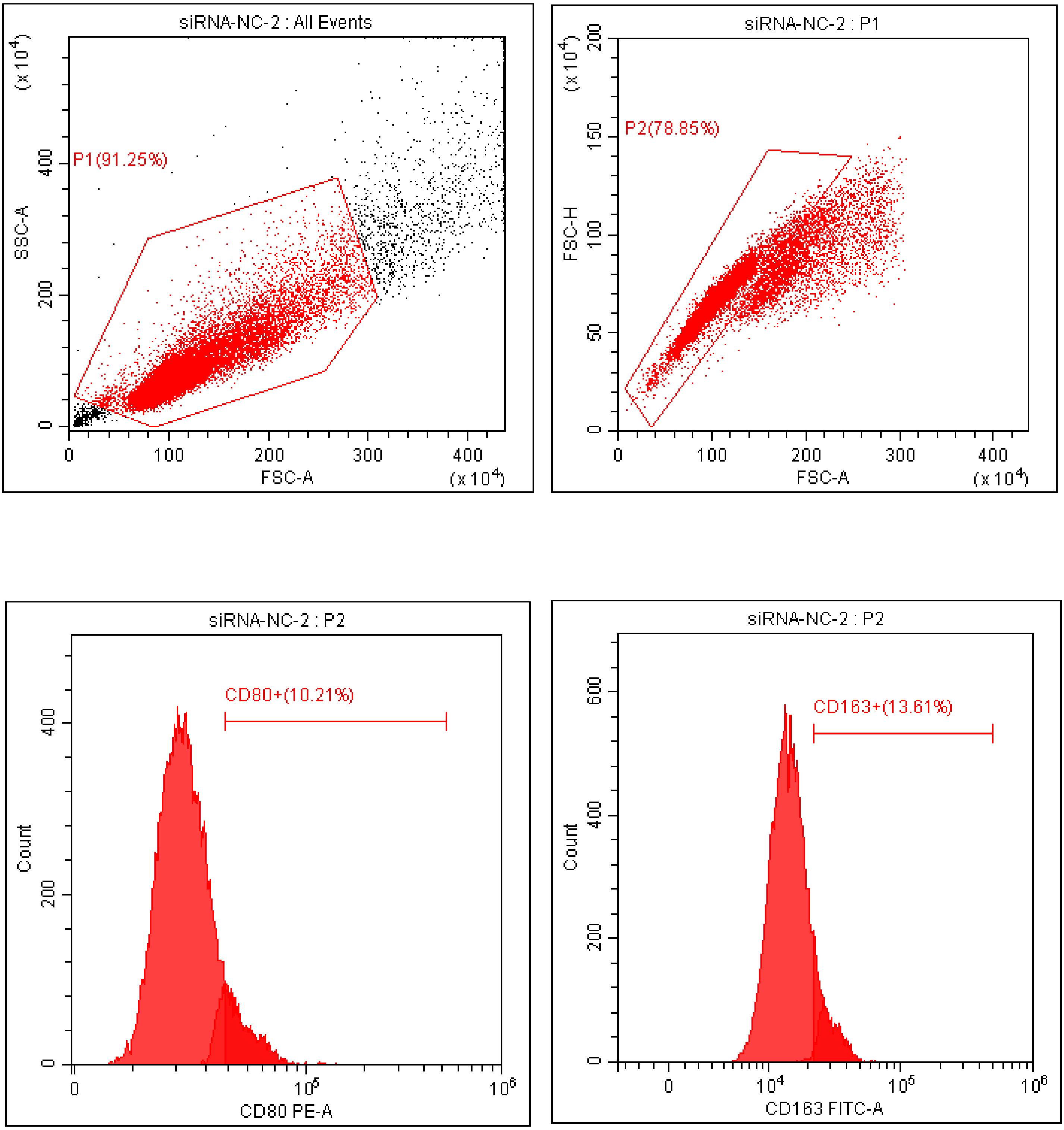

Figure S6 siRNA-NC-3 CD80/CD163

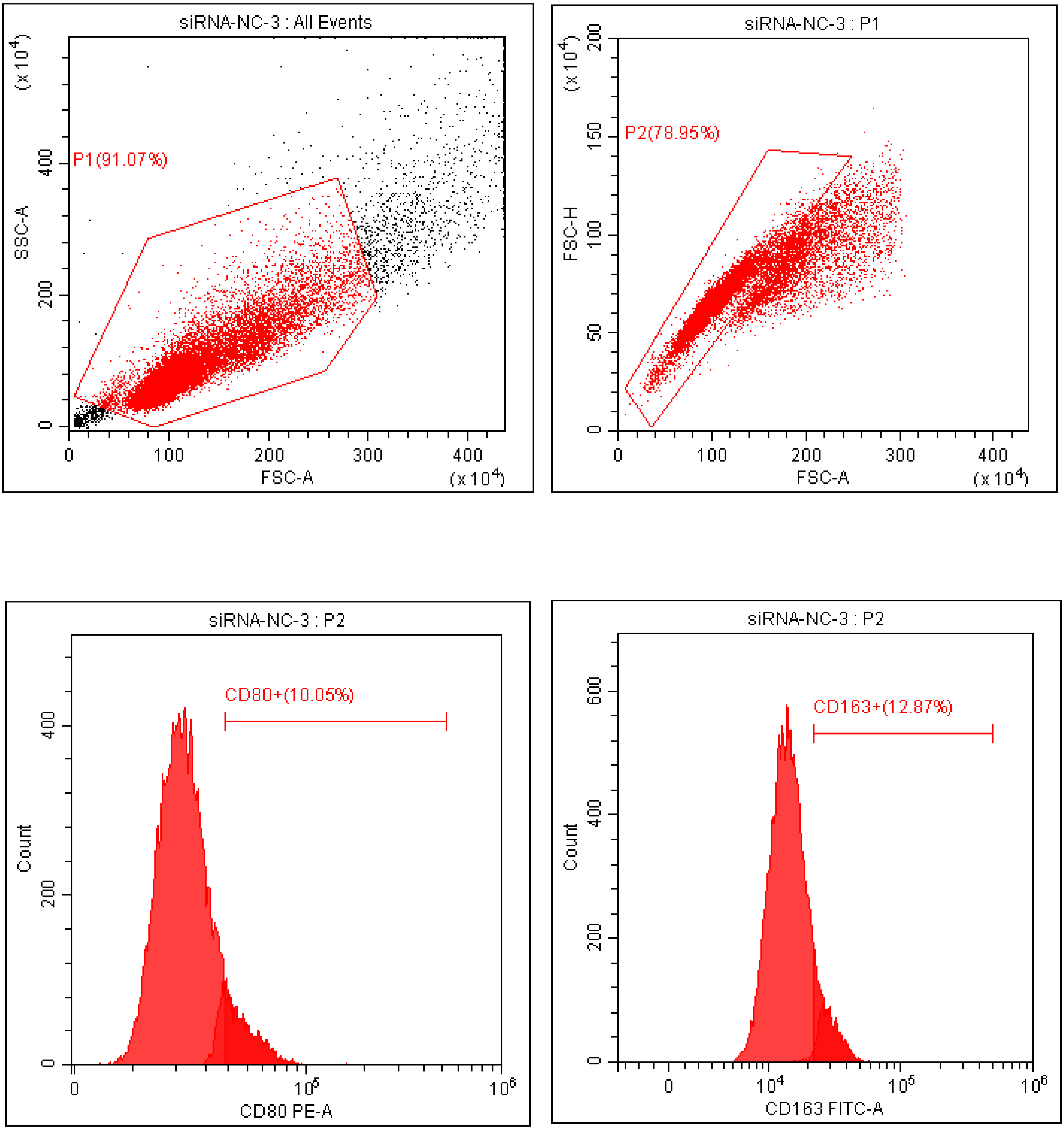

Figure S6 BLANK CD86/CD206

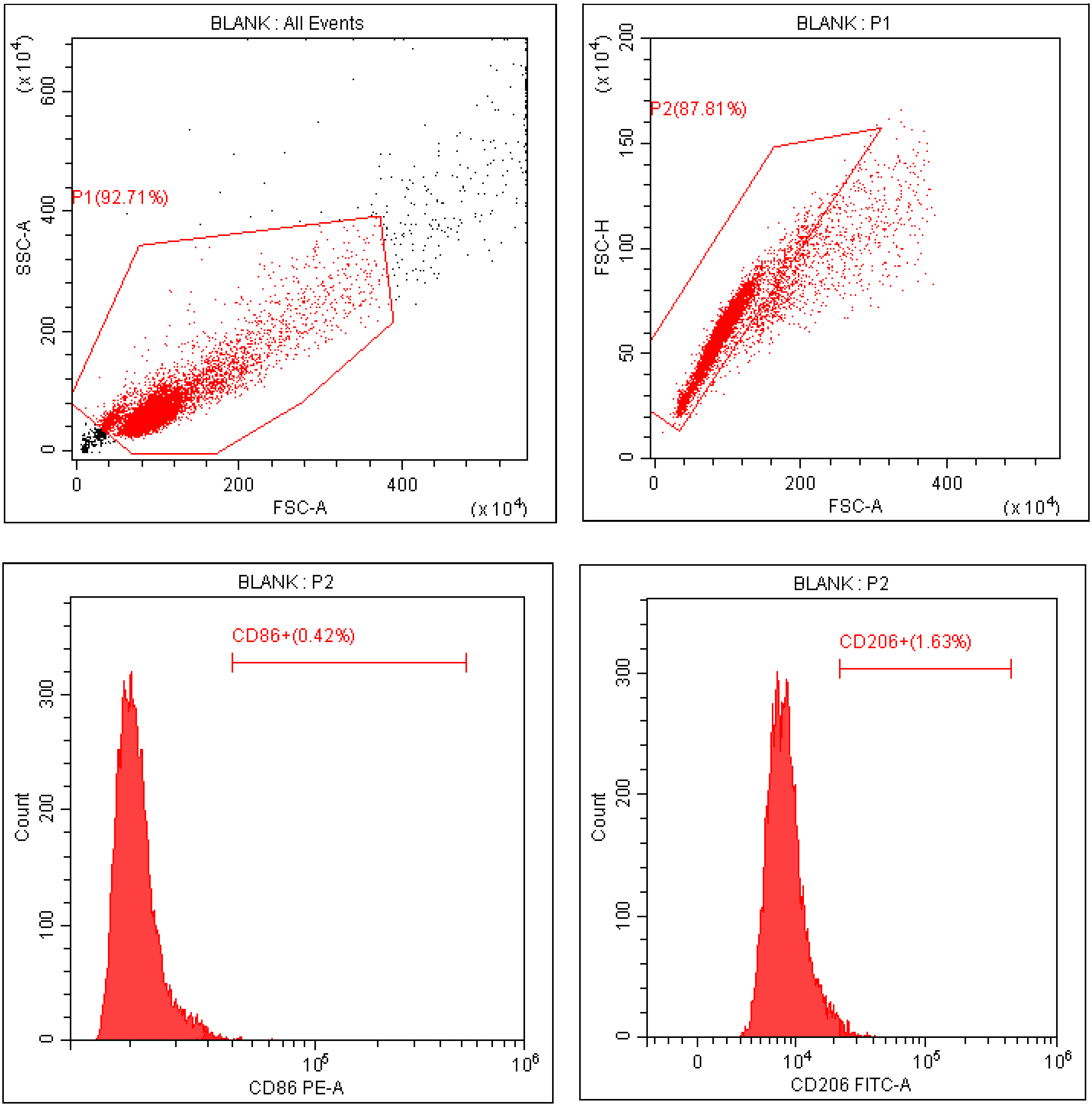

Figure S6 CD86

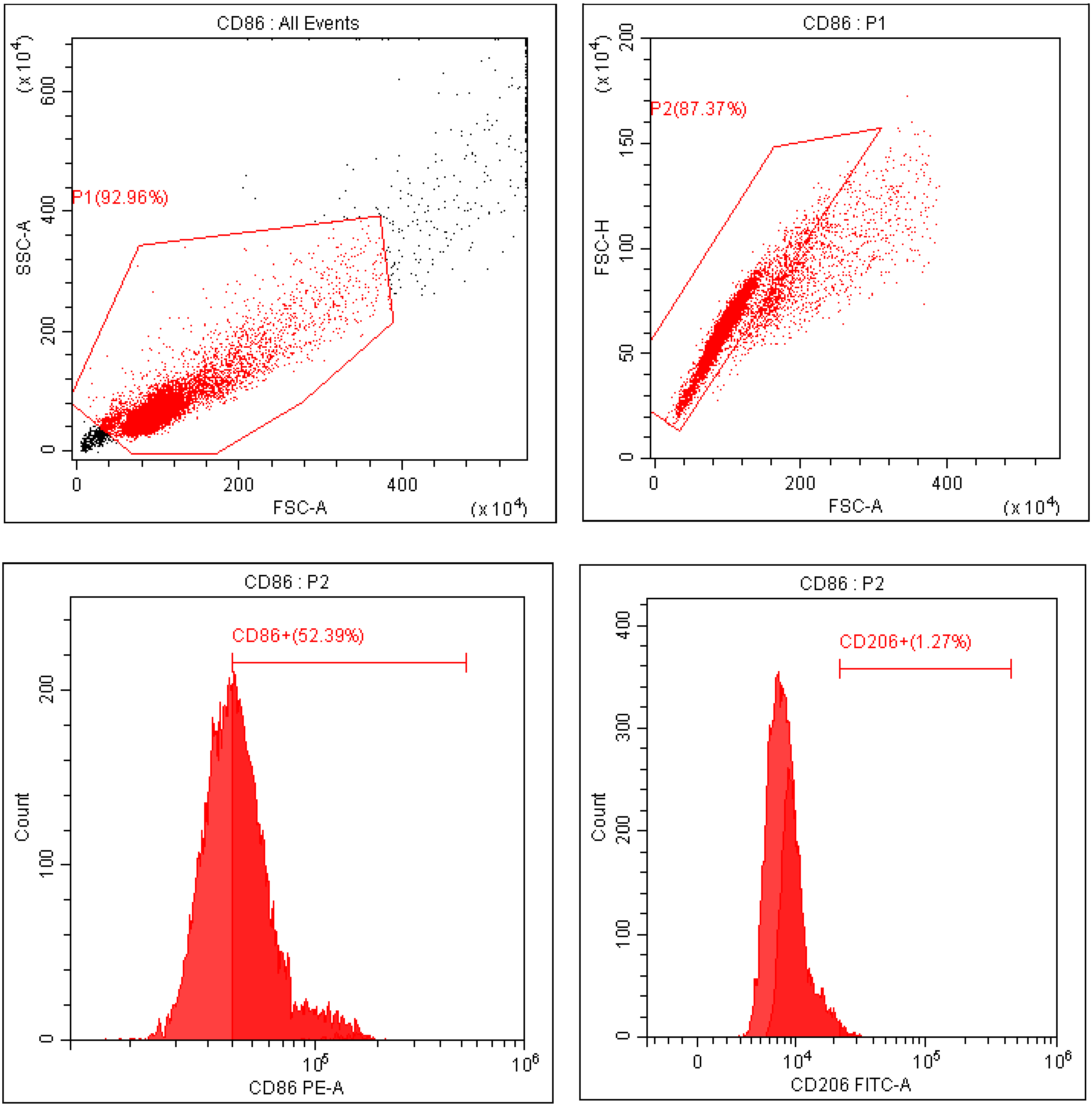

Figure S6 CD206

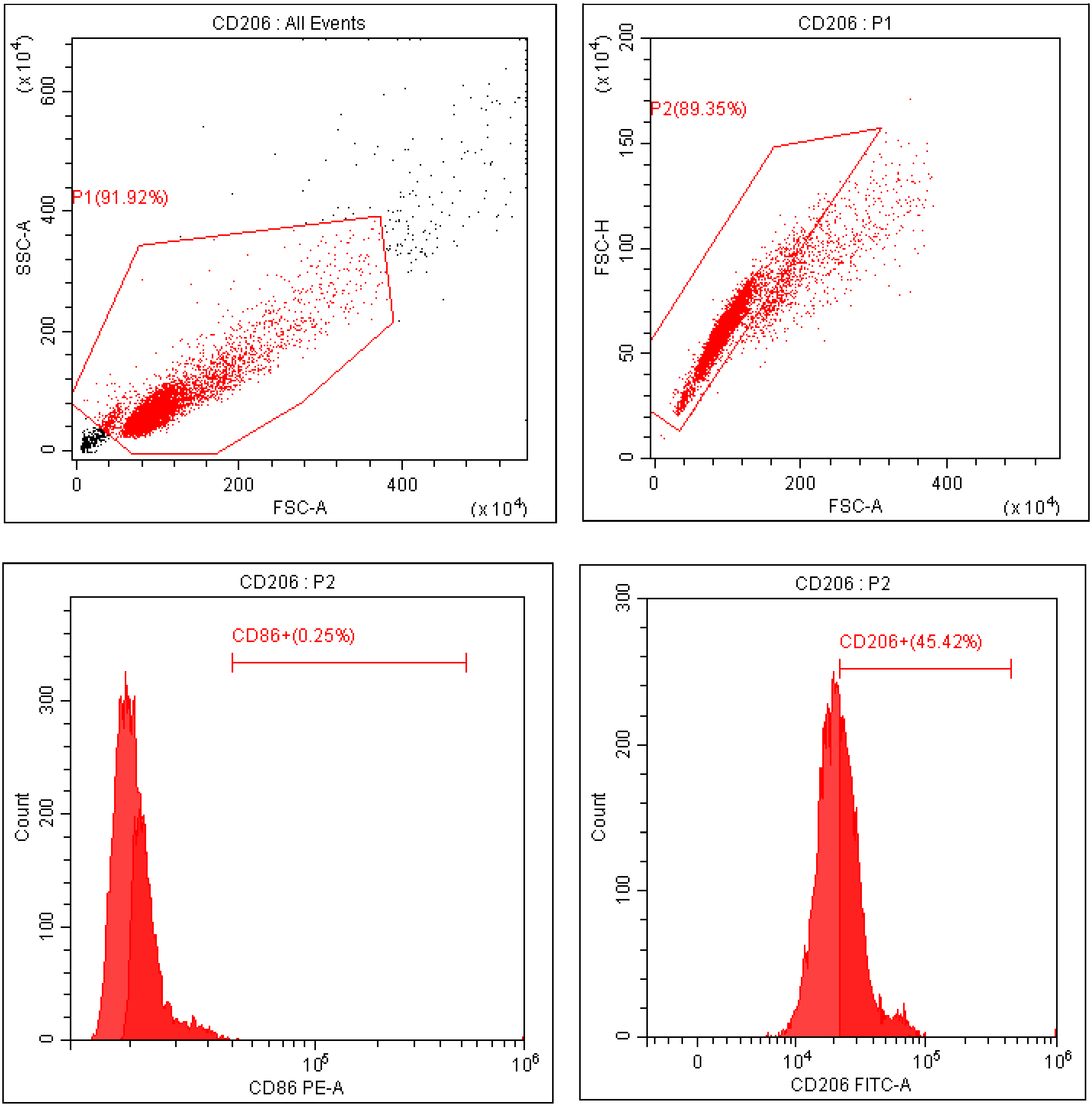

Figure S6 siRNA-PPAR γ +ox-LDL+BS-1 CD86/CD206

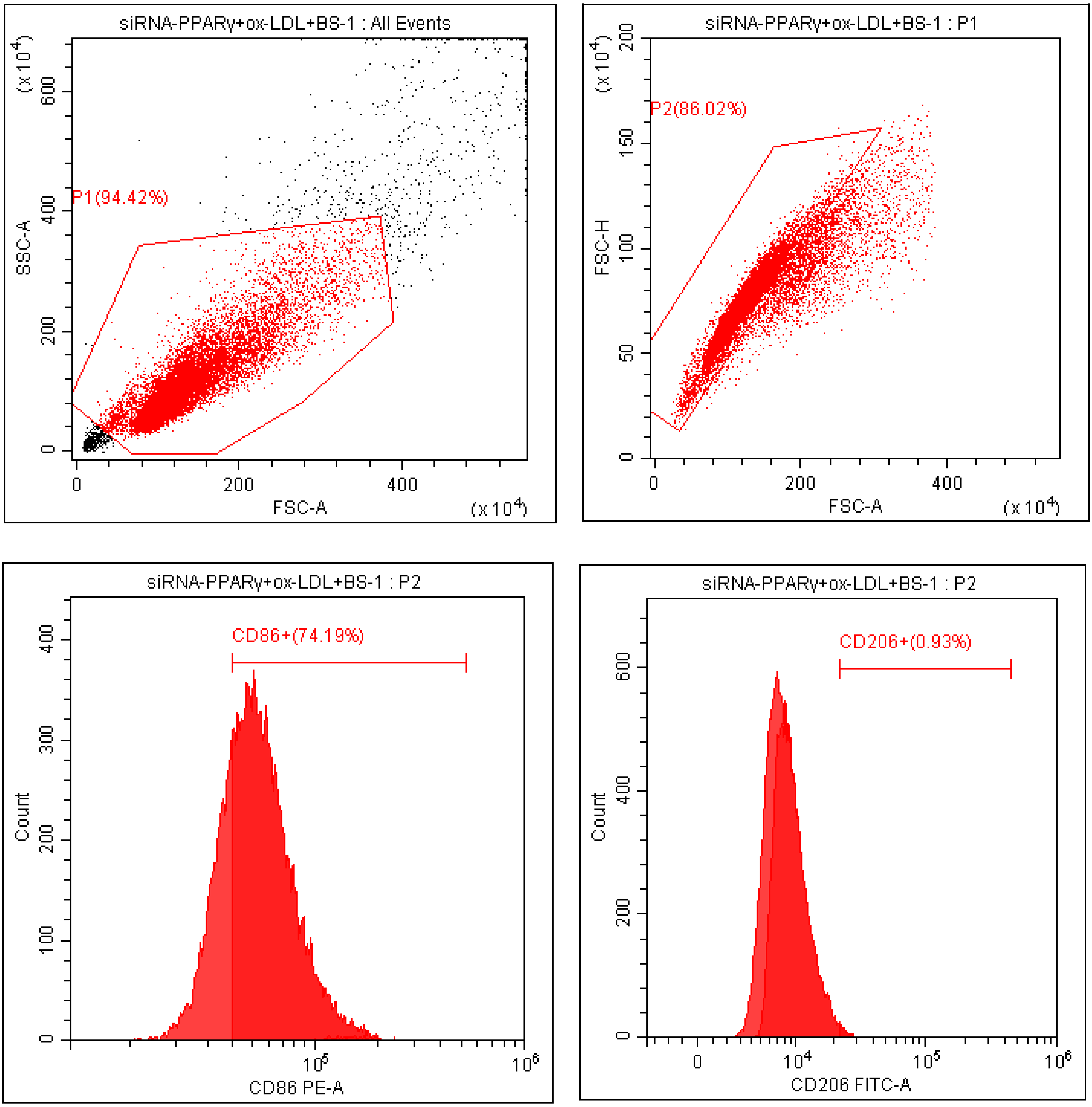

Figure S6 siRNA-PPAR γ +ox-LDL+BS-2 CD86/CD206

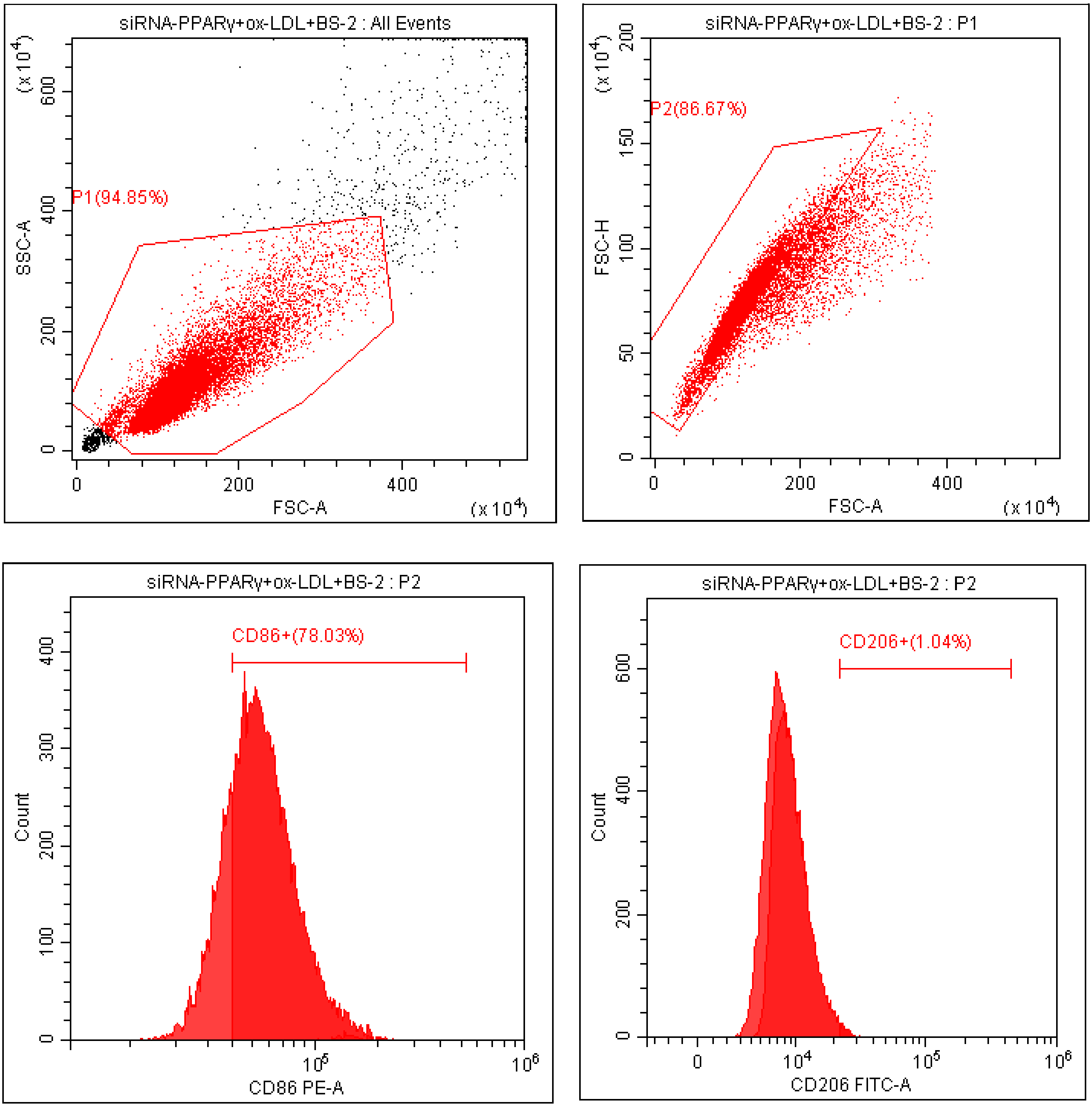

Figure S6 siRNA-PPAR γ +ox-LDL+BS-3 CD86/CD206

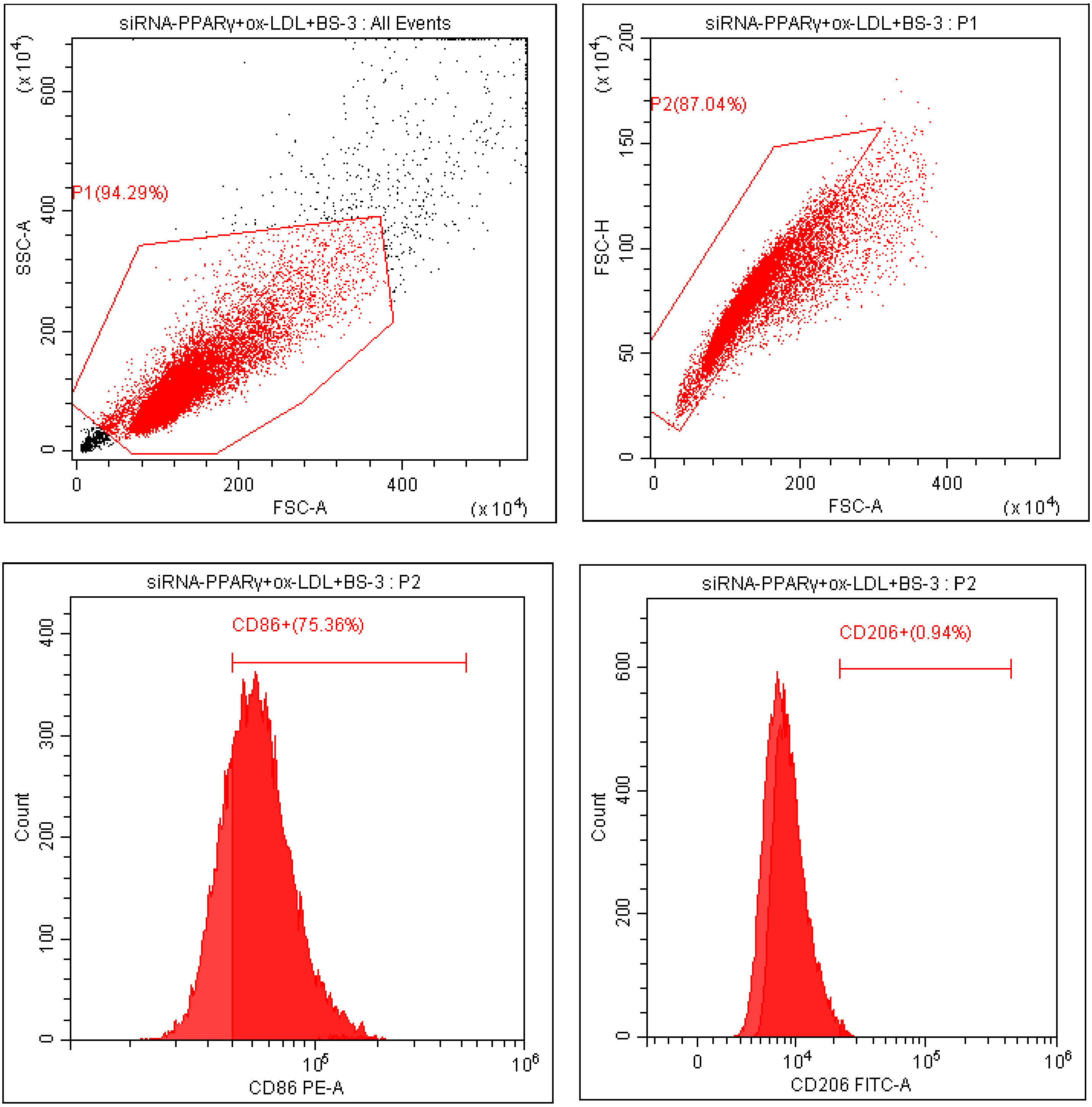

Figure S6 siRNA-PPAR γ +ox-LDL+YQHXF-CS-1 CD86/CD206

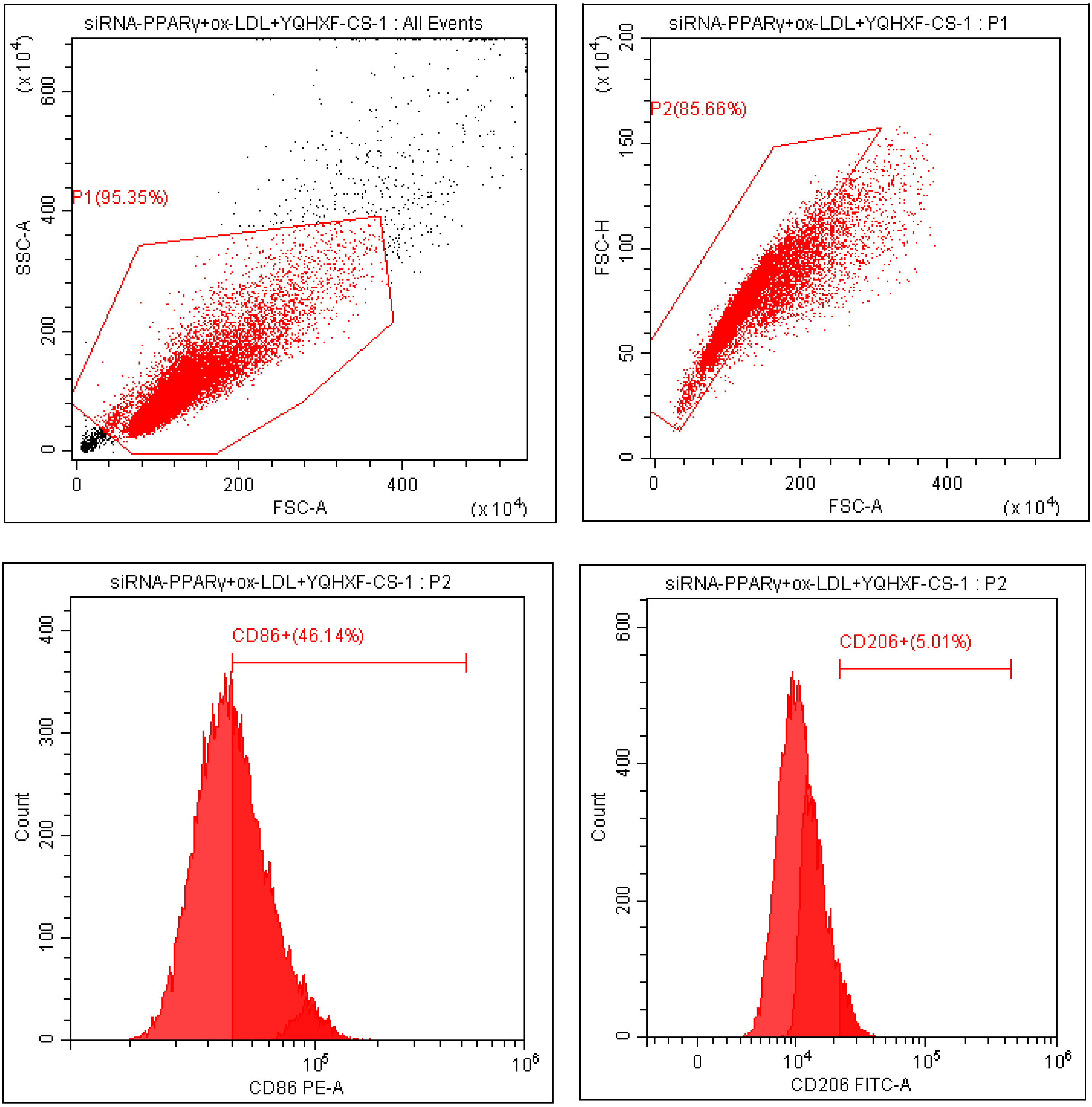

Figure S6 siRNA-PPAR γ +ox-LDL+YQHXF-CS-2 CD86/CD206

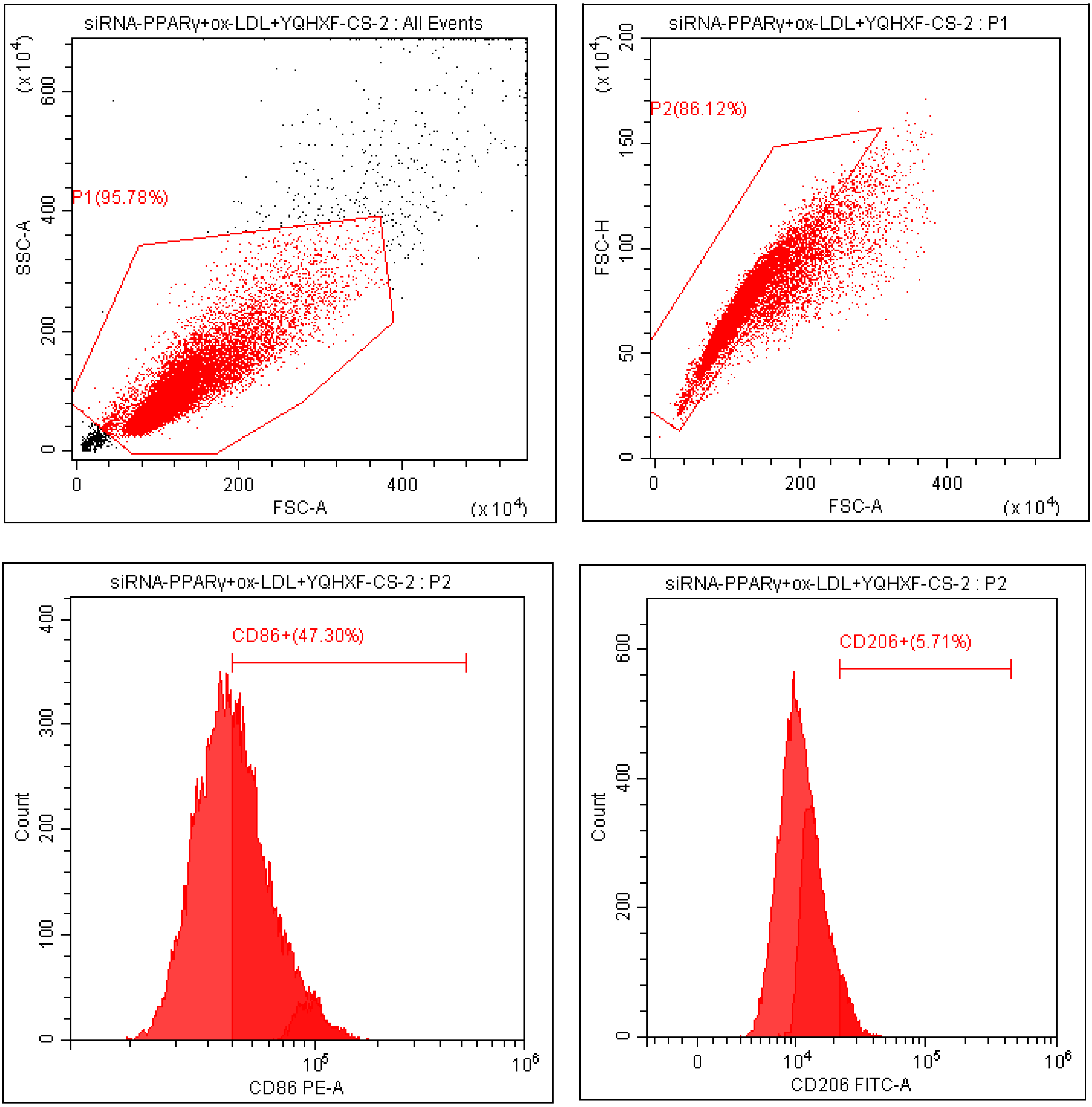

Figure S6 siRNA-PPAR γ +ox-LDL+YQHXF-CS-3 CD86/CD206

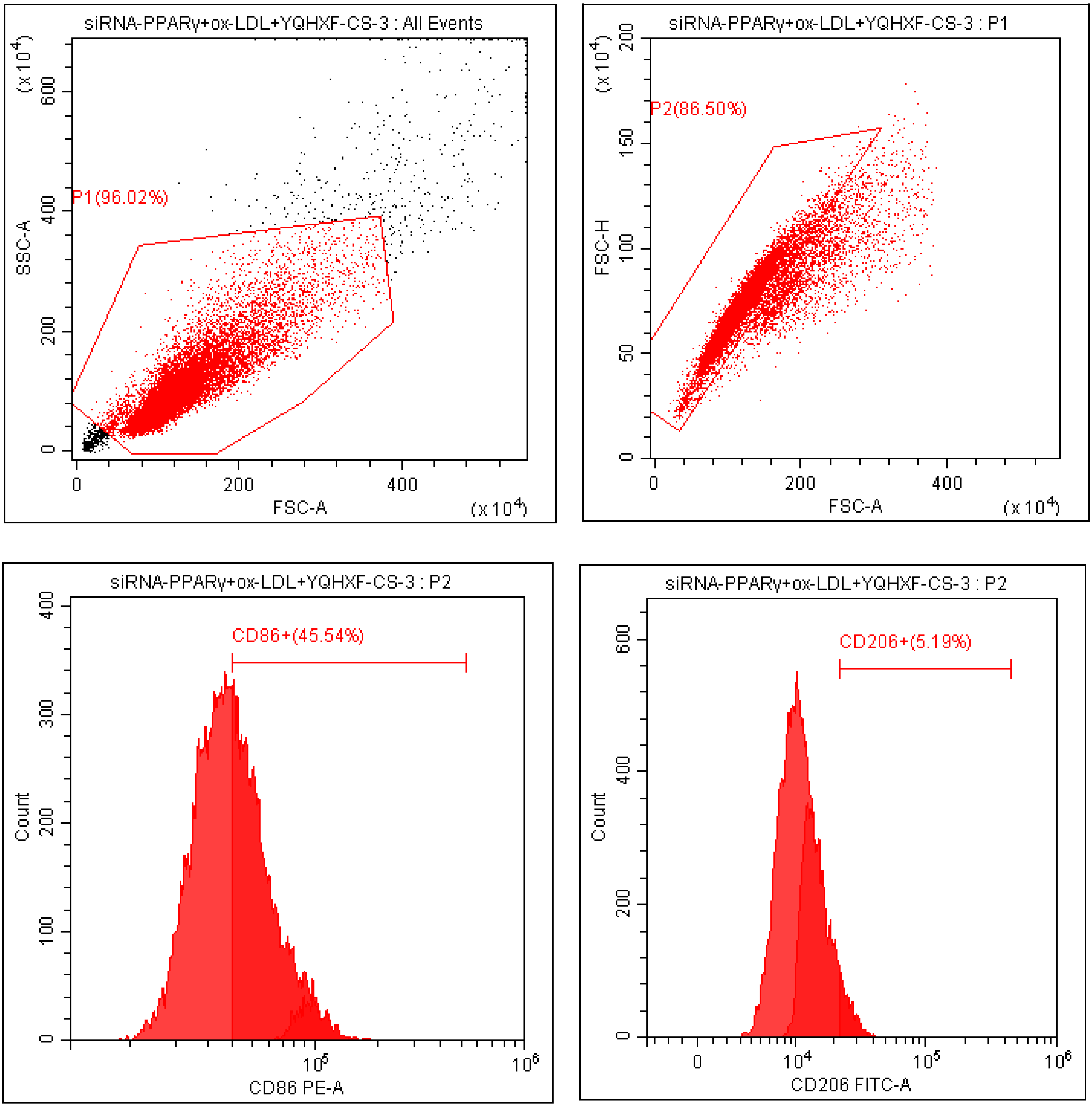

Figure S6 siRNA-NC+ox-LDL+BS-1 CD86/CD206

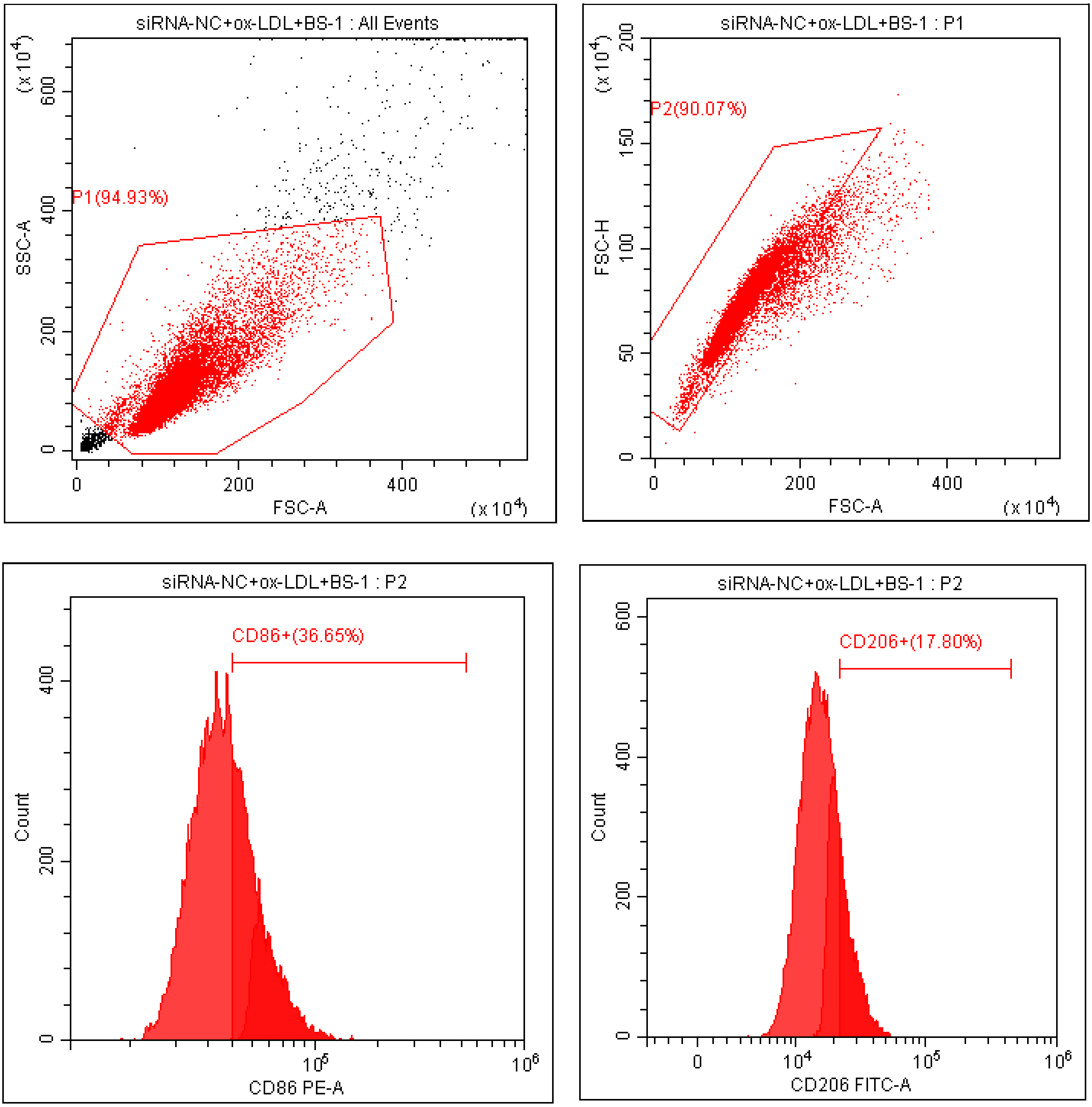

Figure S6 siRNA-NC+ox-LDL+BS-2 CD86/CD206

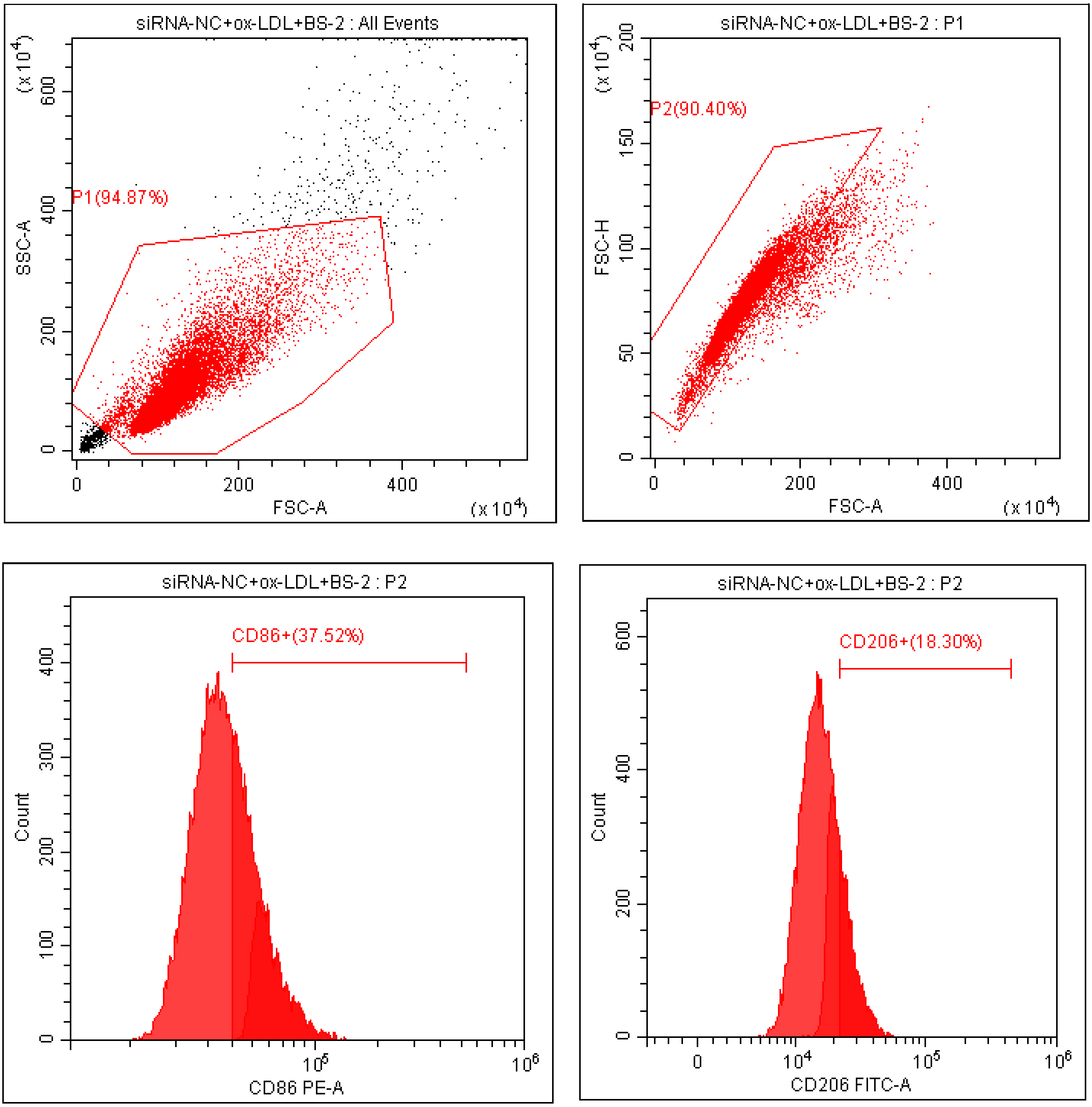

Figure S6 siRNA-NC+ox-LDL+BS-3 CD86/CD206

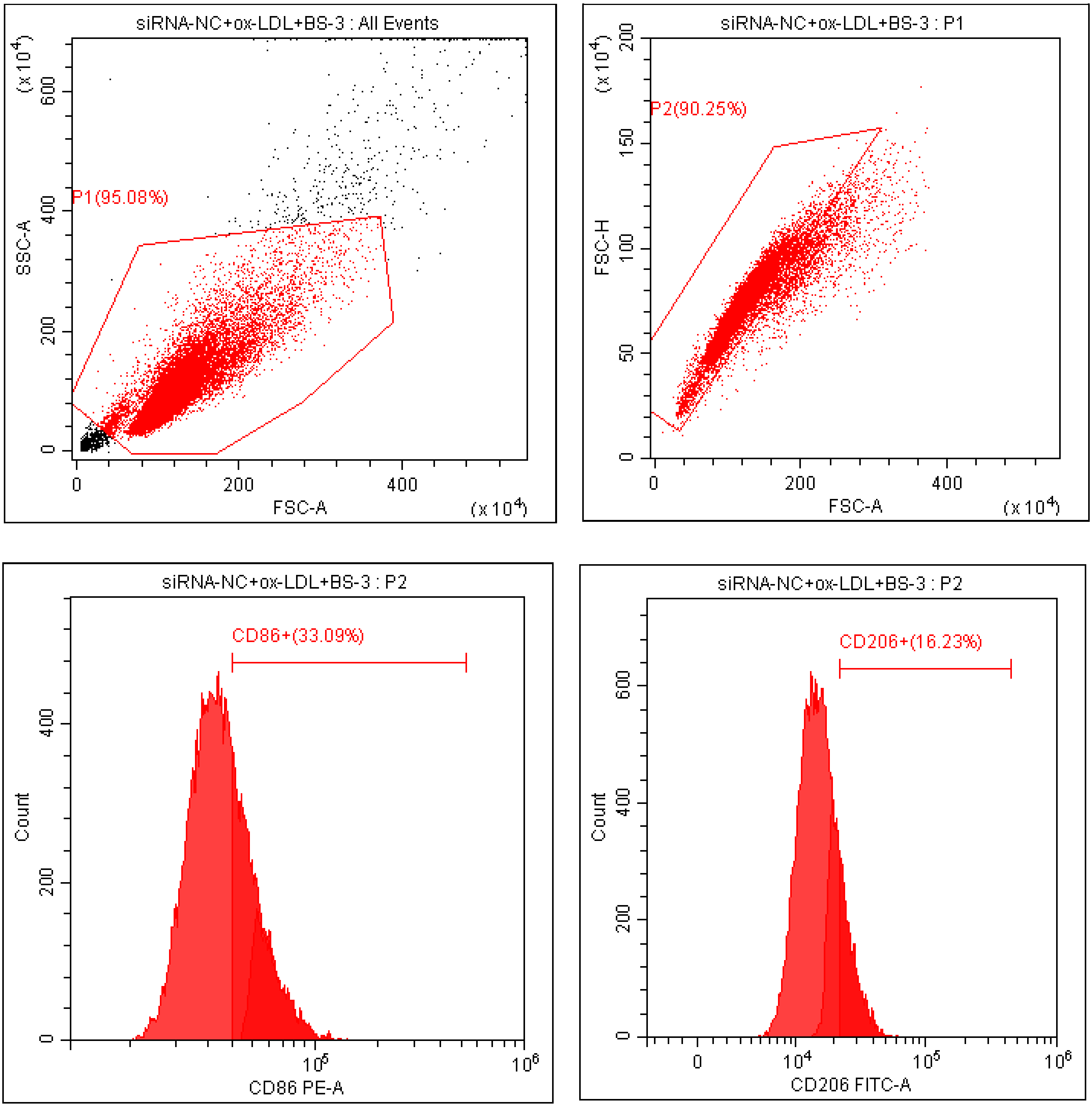

Figure S6 siRNA-PPAR γ -1 CD86/CD206

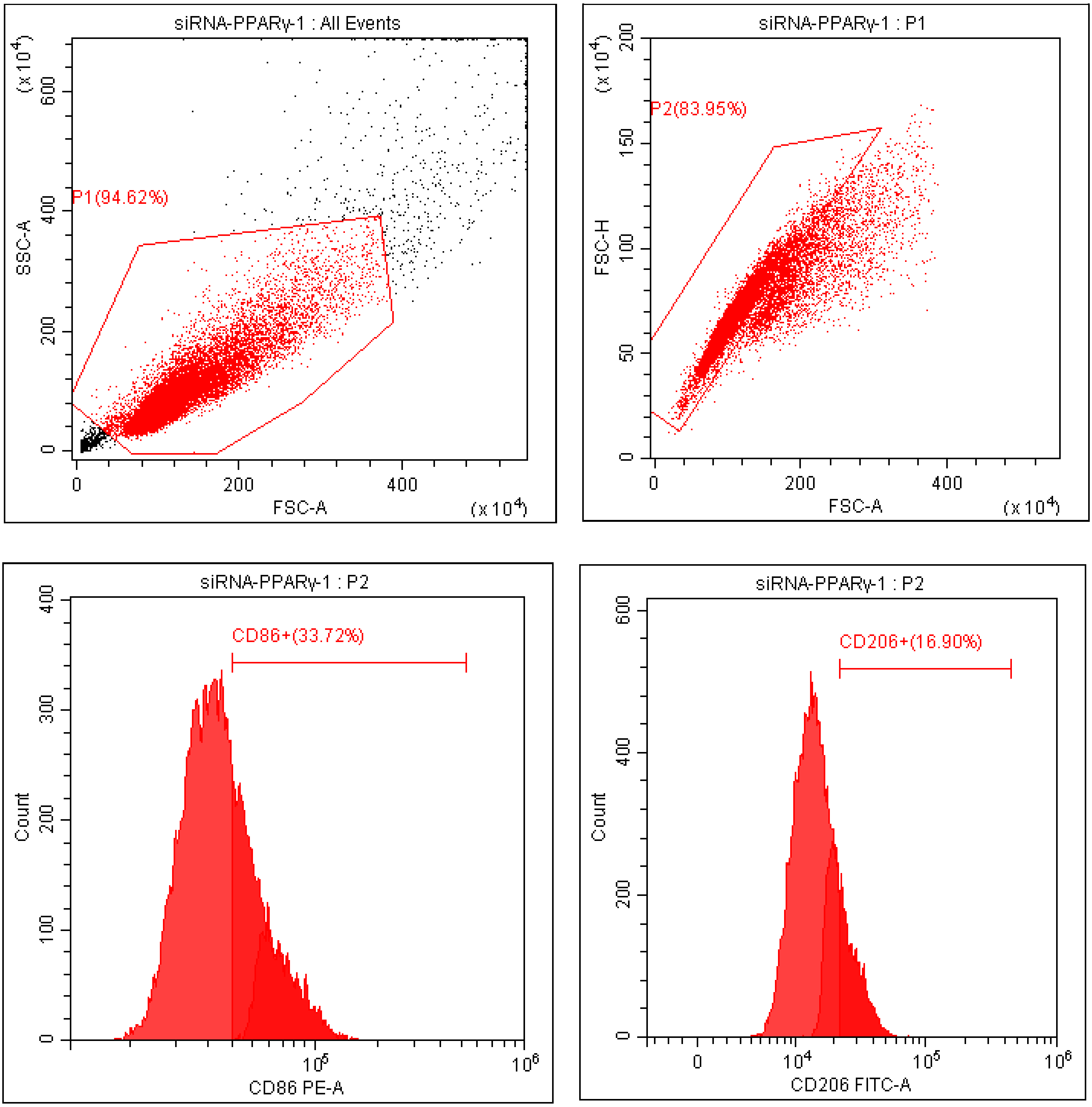

Figure S6 siRNA-PPAR γ -2 CD86/CD206

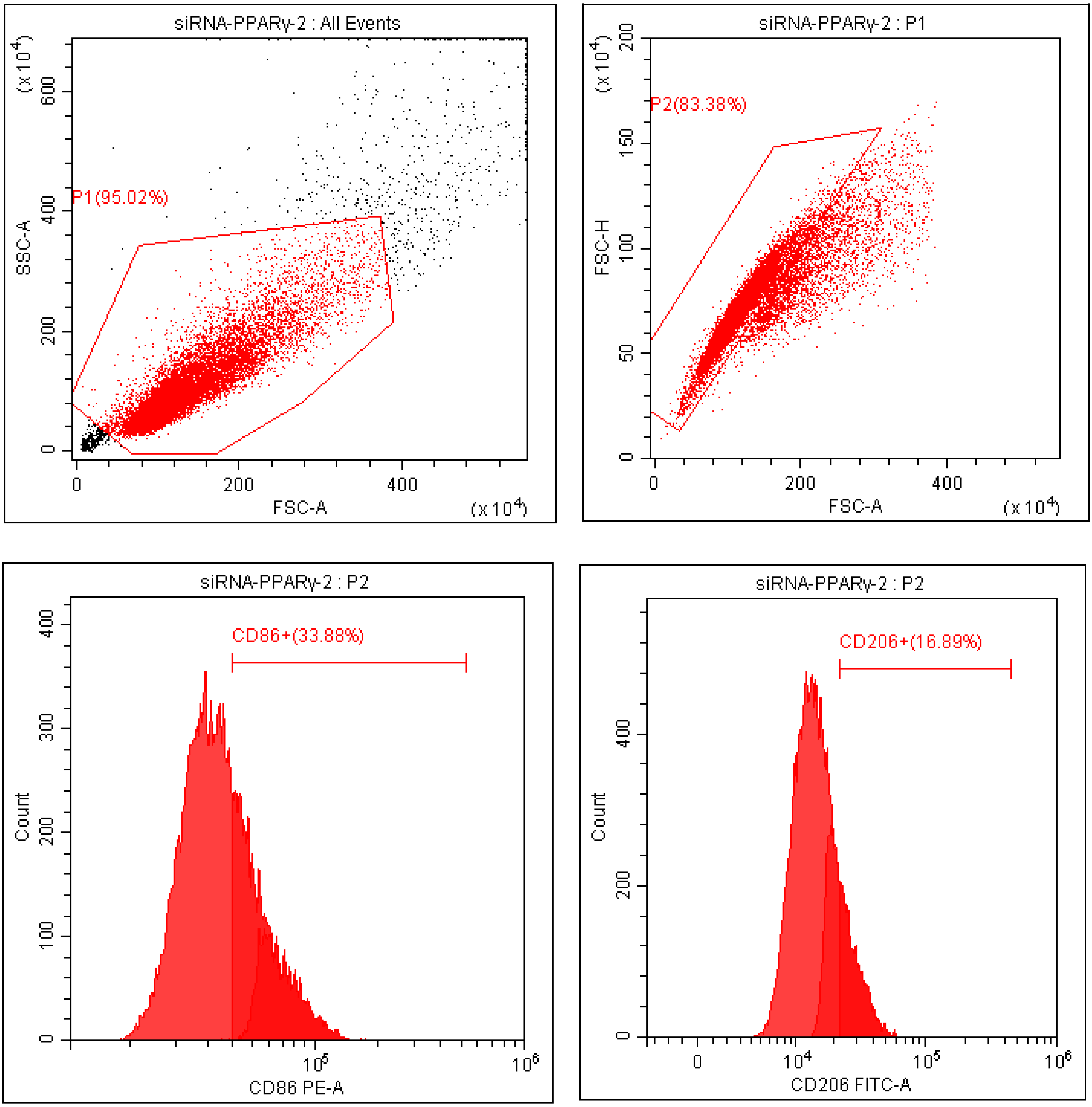

Figure S6 siRNA-PPAR γ -3 CD86/CD206

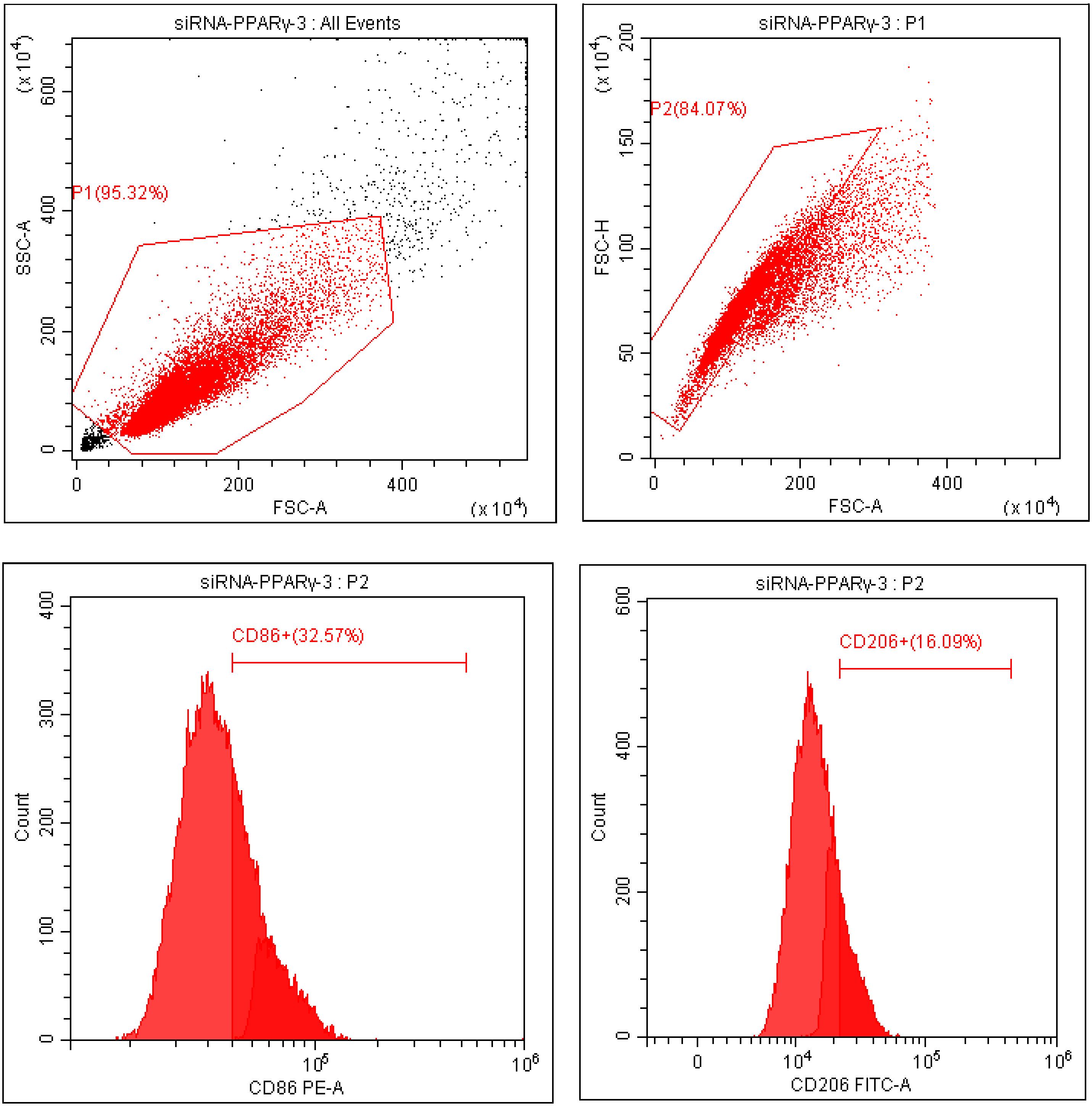

Figure S6 siRNA-NC+ox-LDL+YQHXF-CS-1 CD86/CD206

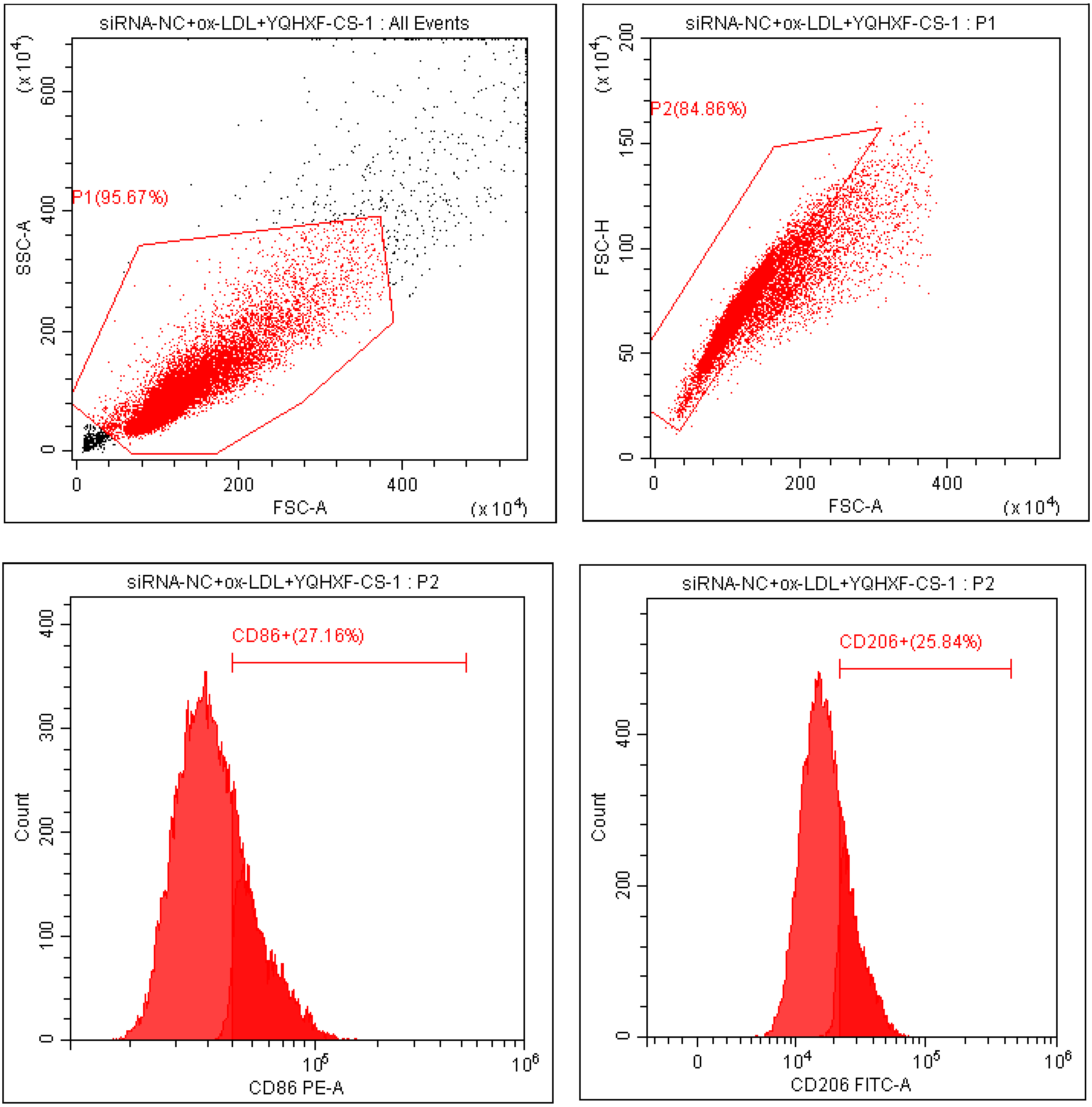

Figure S6 siRNA-NC+ox-LDL+YQHXF-CS-2 CD86/CD206

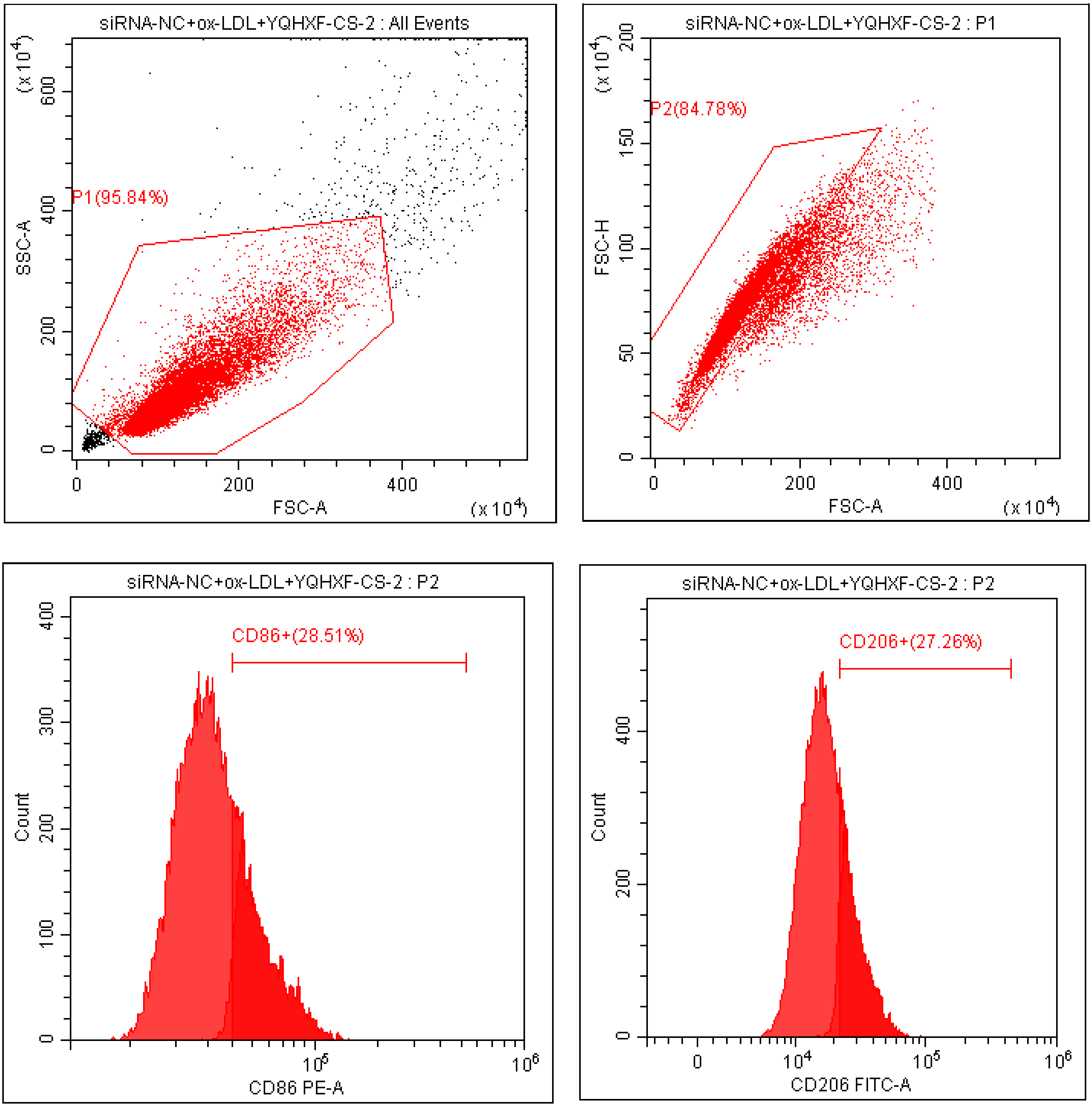

Figure S6 siRNA-NC+ox-LDL+YQHXF-CS-3 CD86/CD206

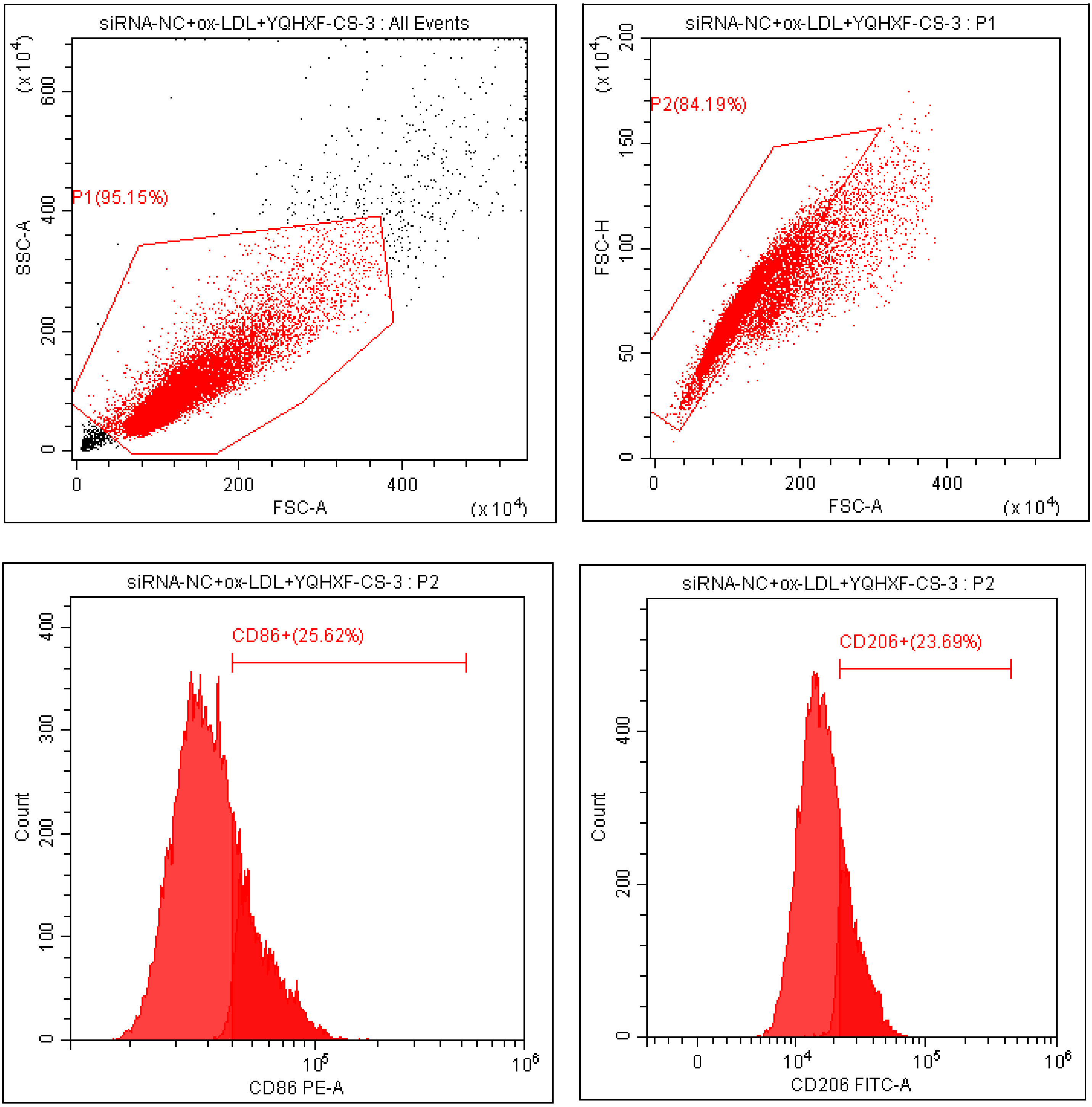

Figure S6 siRNA-NC-1 CD86/CD206

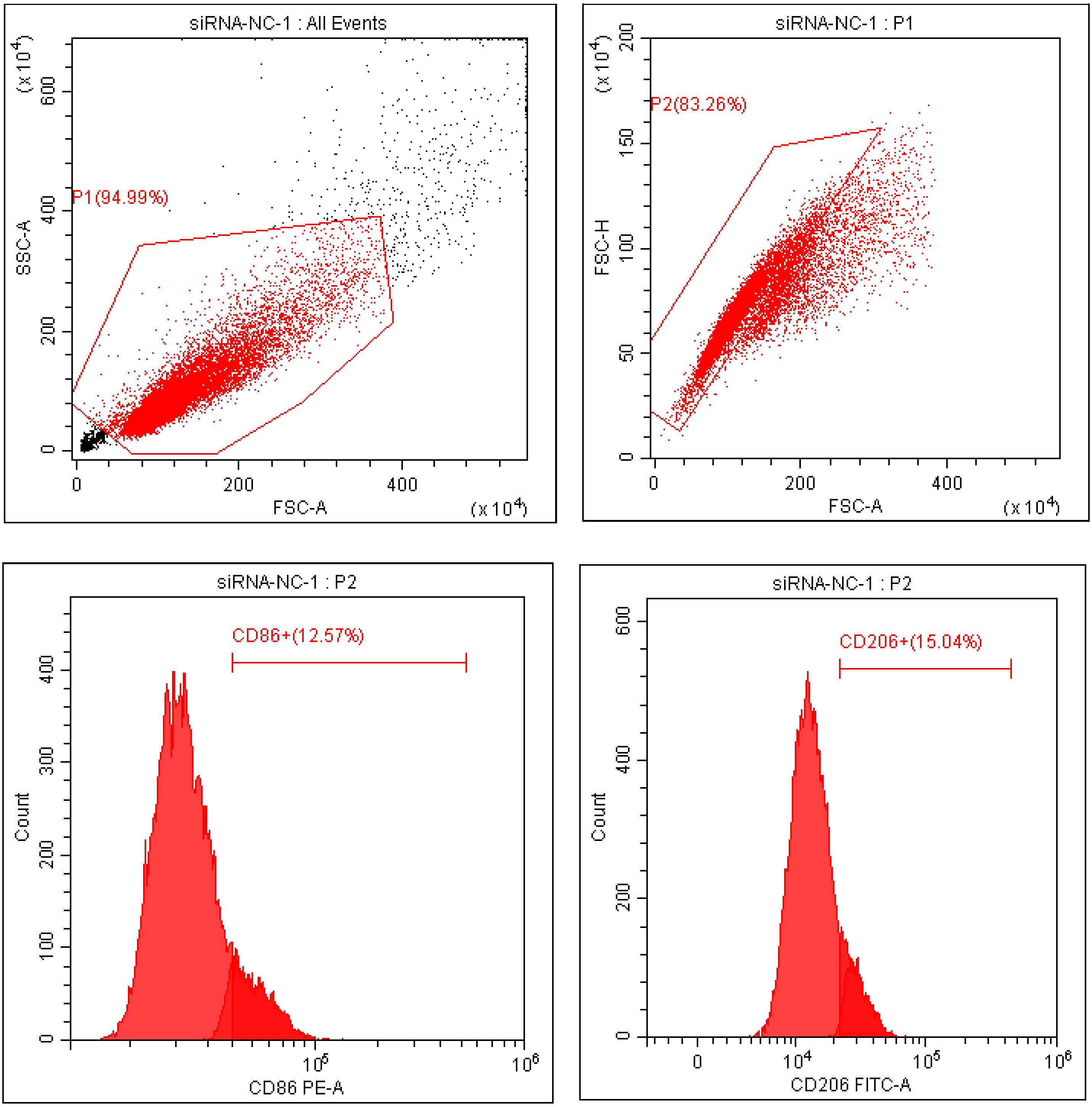

Figure S6 siRNA-NC-2 CD86/CD206

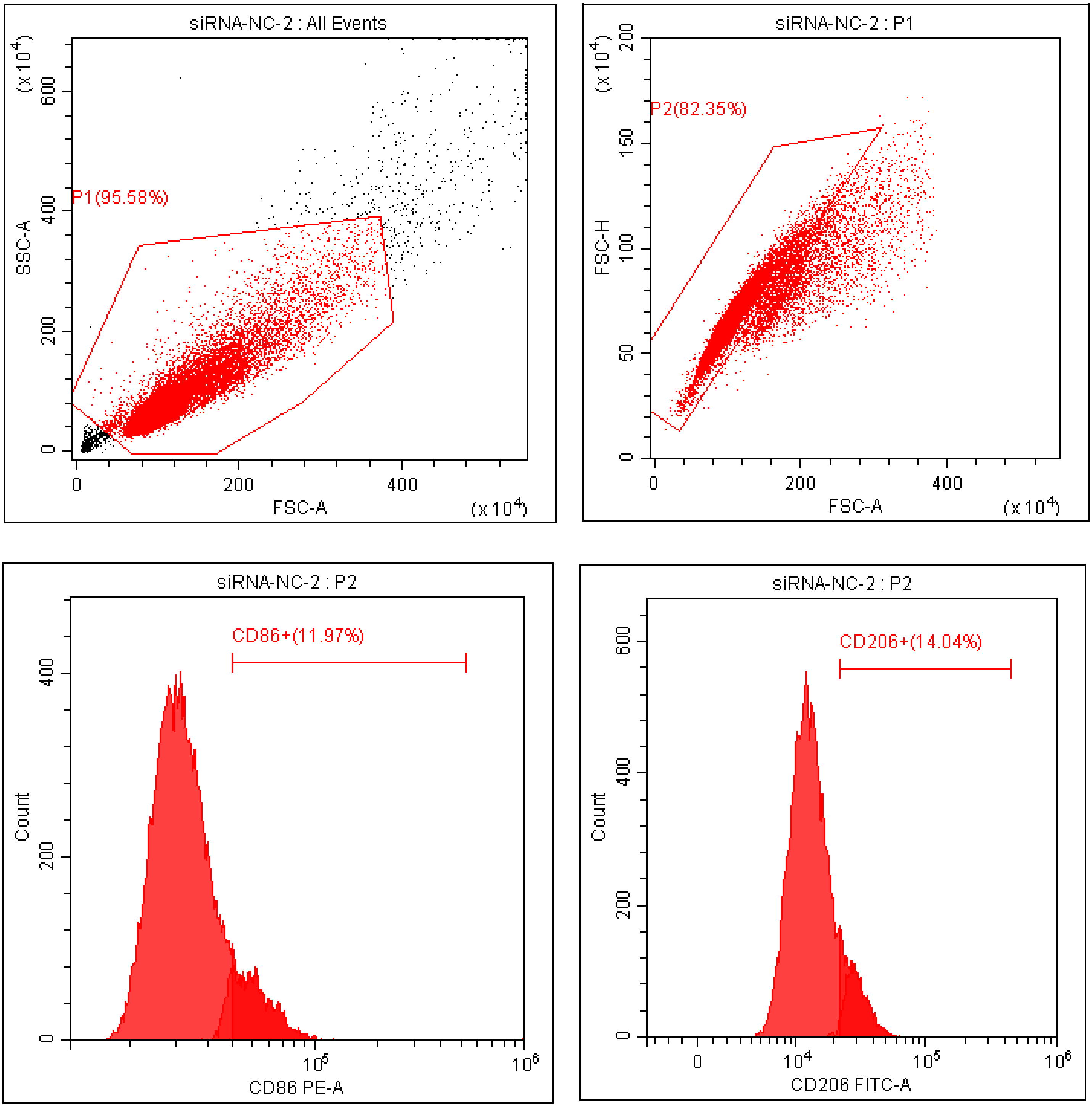

Figure S6 siRNA-NC-3 CD86/CD206

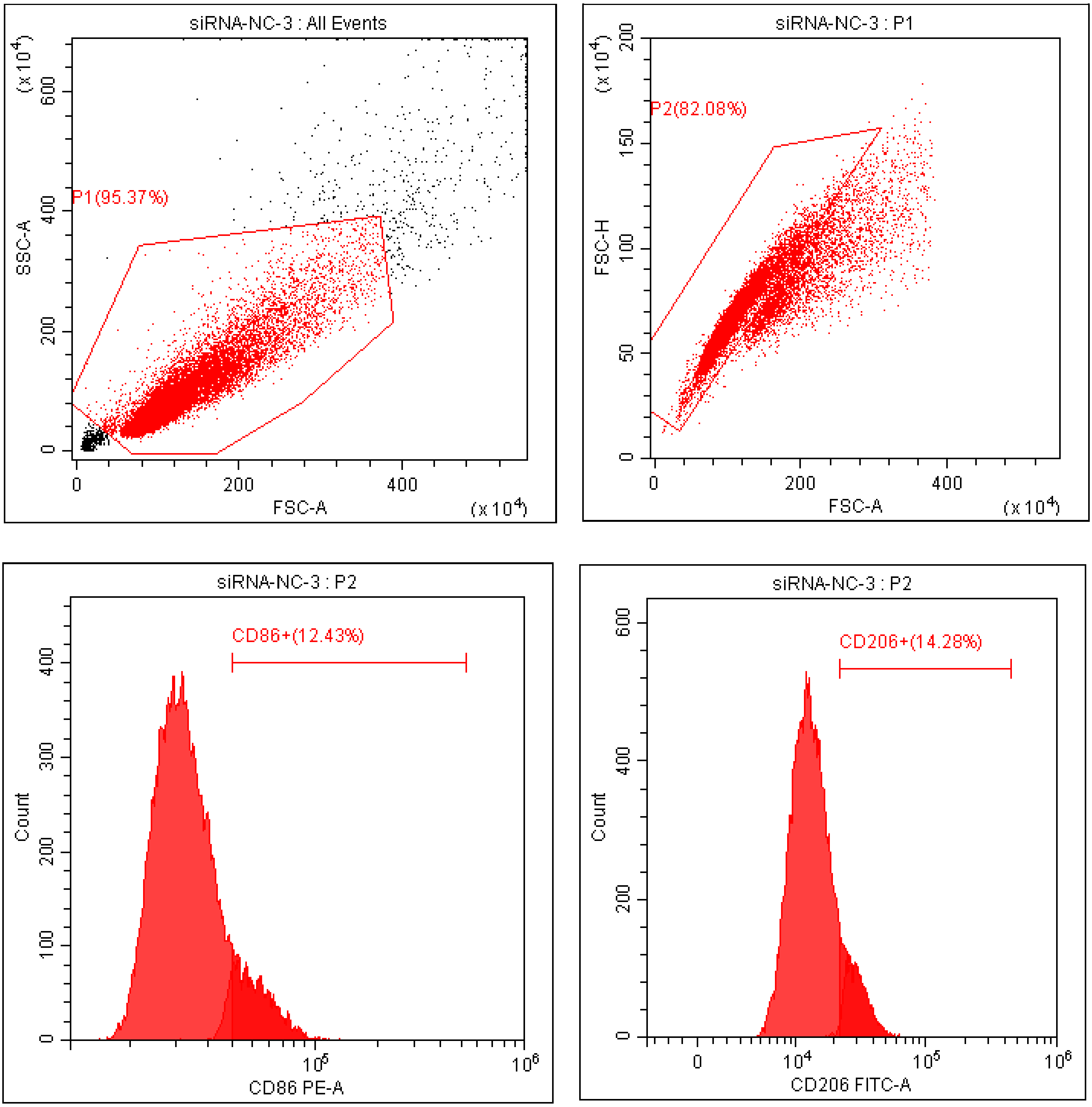

Supplement: Supplementary file 1 [file Datasheet1.pdf]
