# Supplementary material for: Yiqihuoxue formula improves atherosclerosis by regulating macrophage polarization associated with enhanced STAT6/PPARγ pathway
Source: Front Cardiovasc Med. 2026 Sep 22;13:1896890. doi: 10.3389/fcvm.2026.1896890 (PMC13638390; doi:10.3389/fcvm.2026.1896890)

Supplementary file 2. Raw images of Western blot.
Figure 3E

(1) CD80

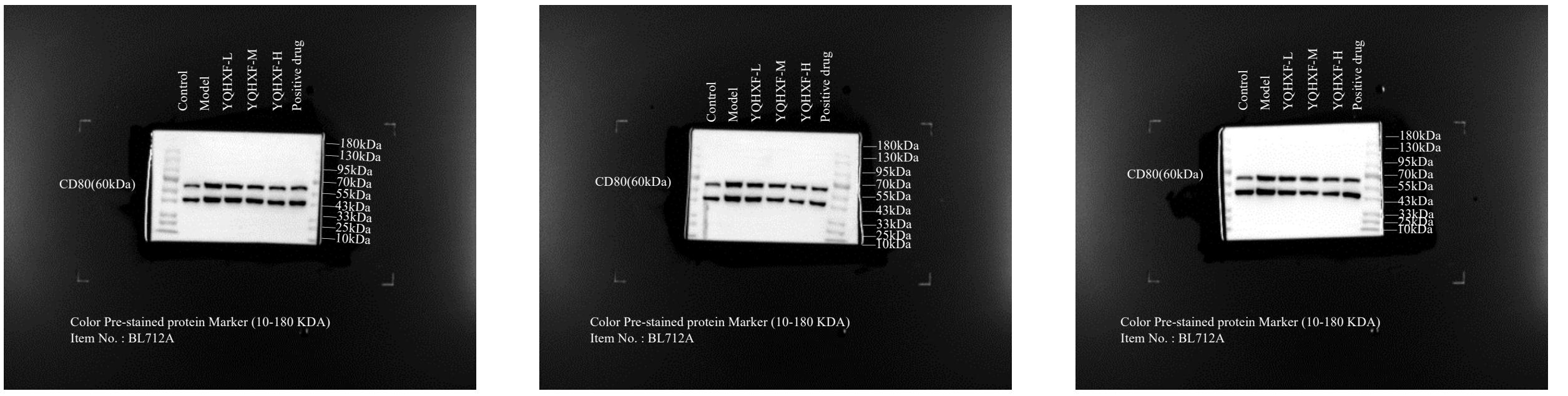

(2) CD86

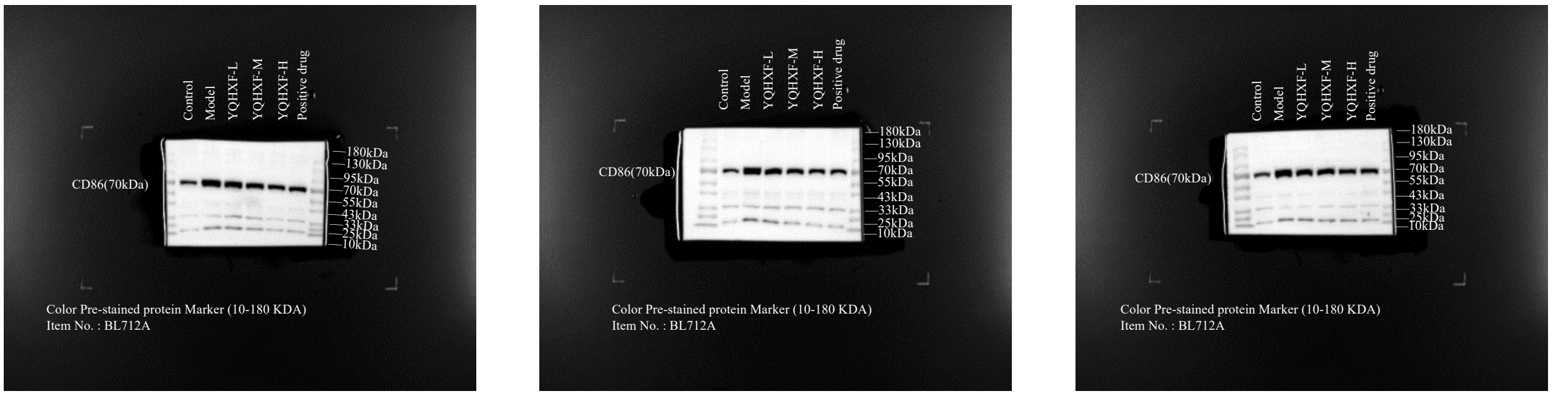

(3) CD163

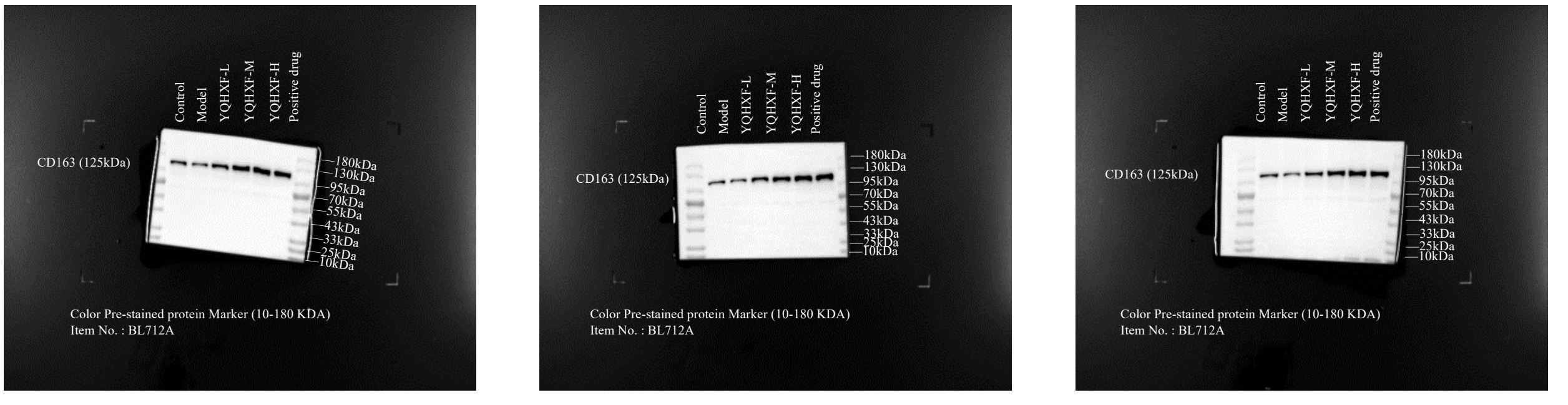

(4) CD206

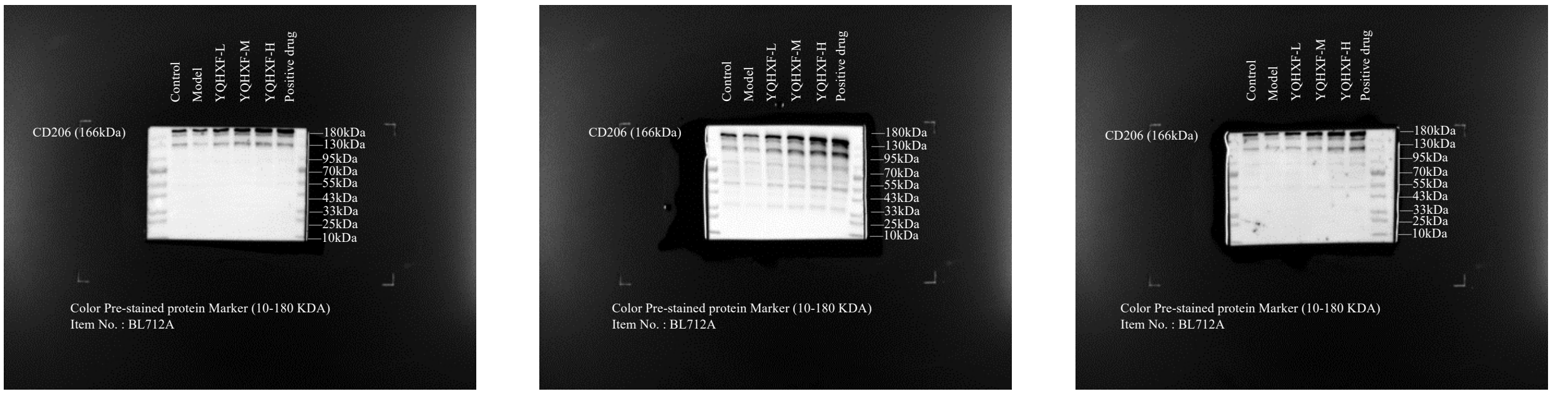

(5) β-actin

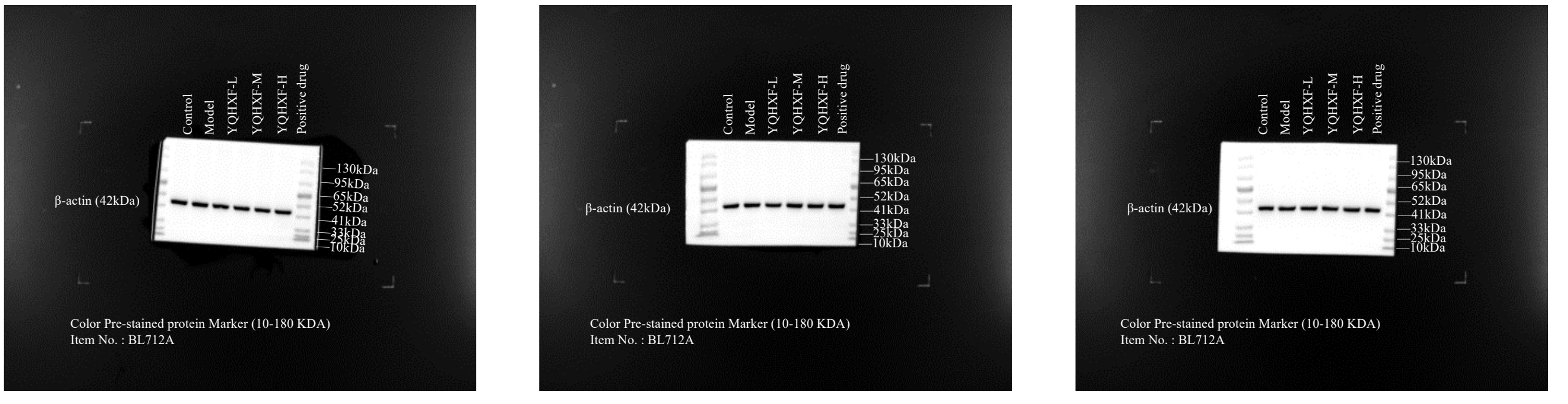

Figure 5A

(1) STAT6

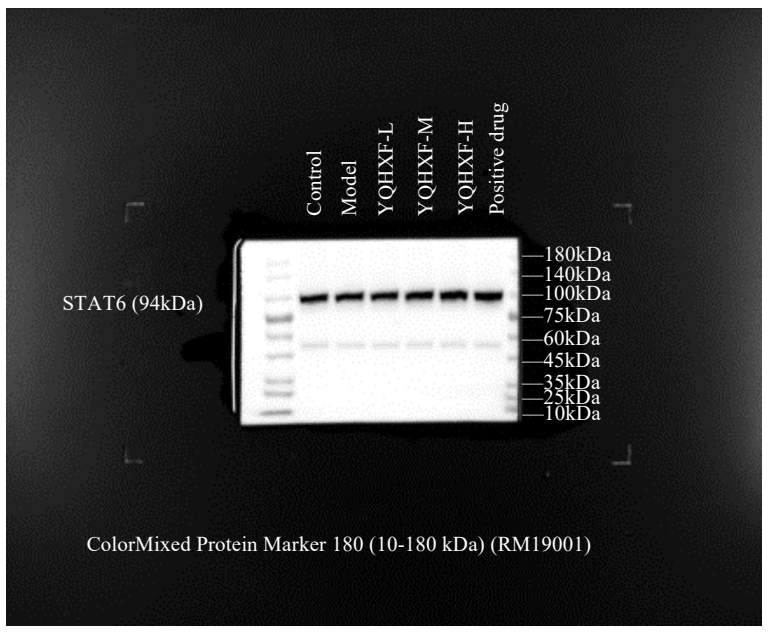

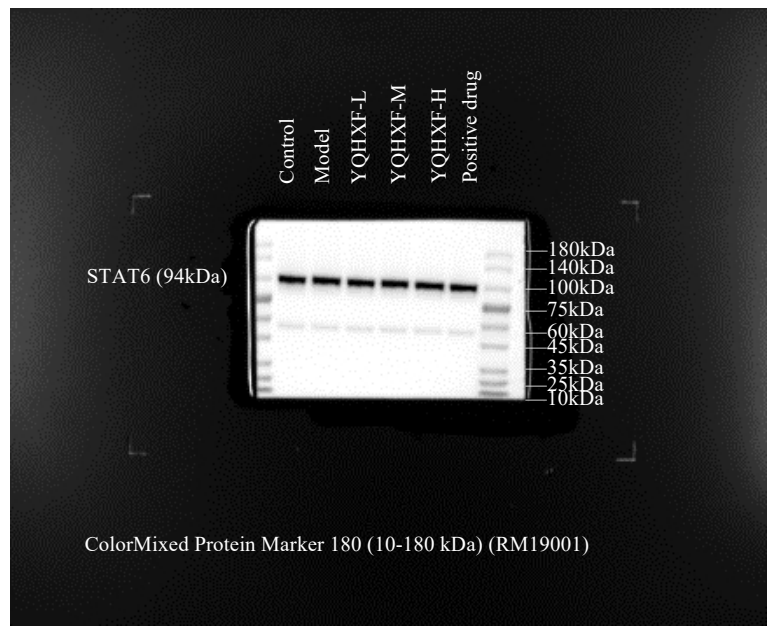

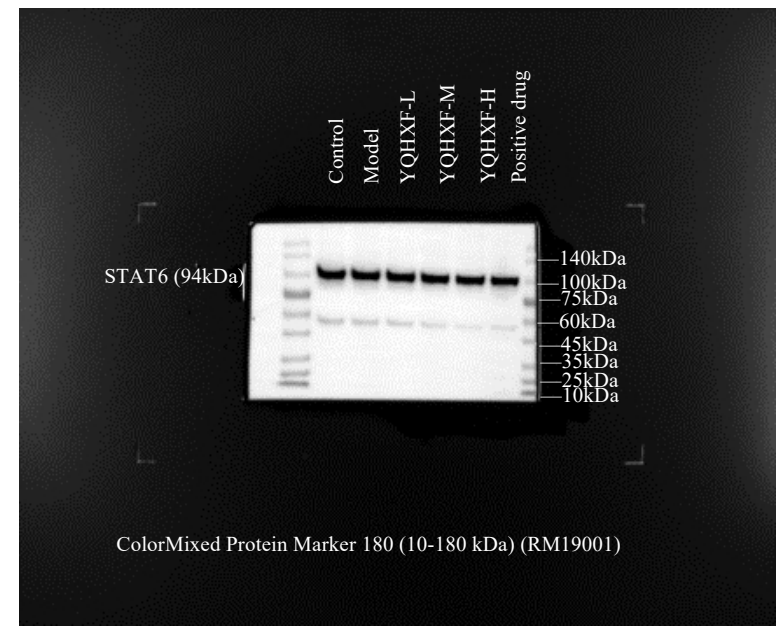

(2) p-STAT6

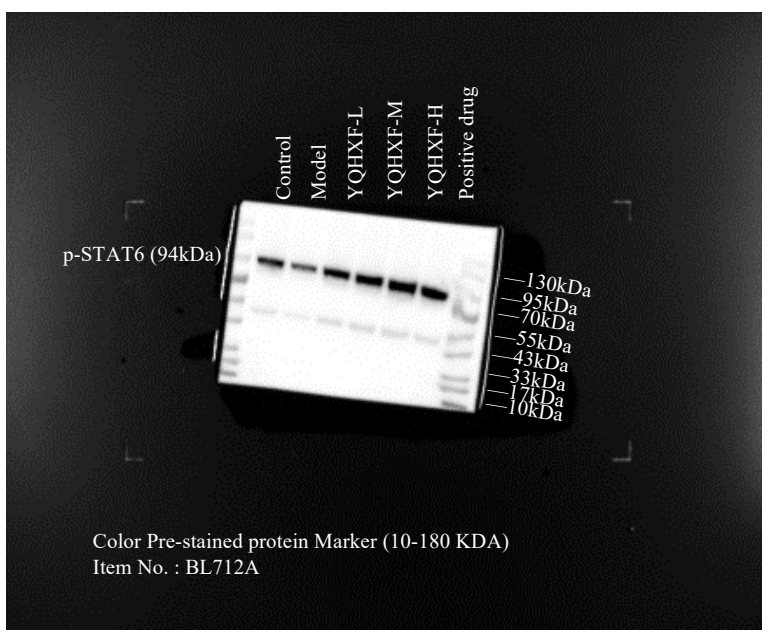

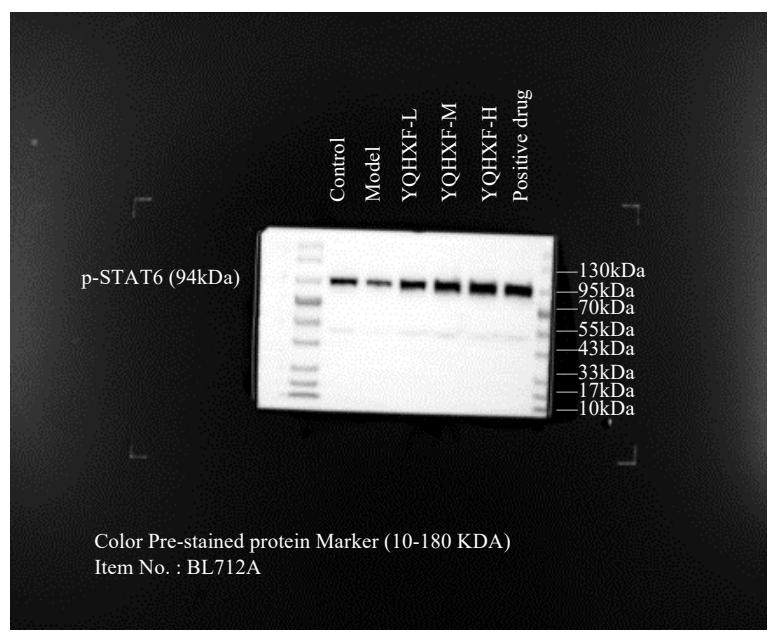

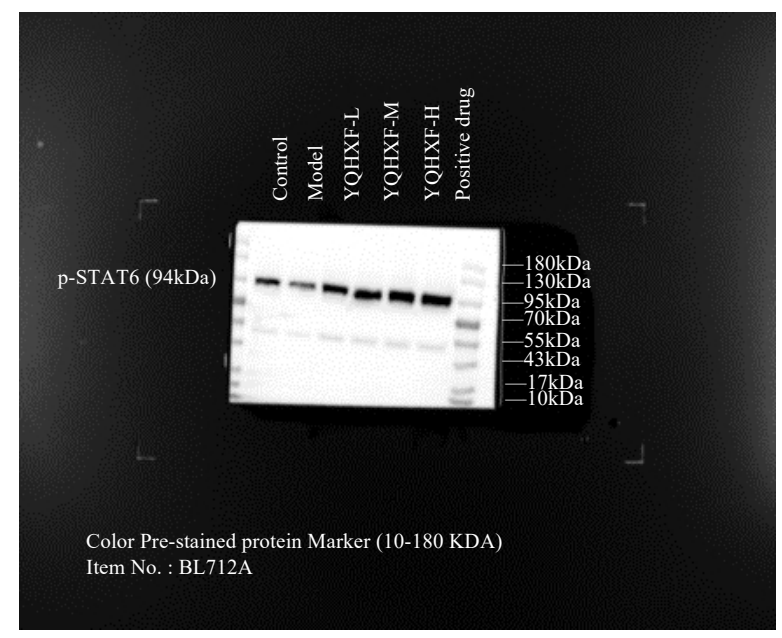

(3) PPARγ

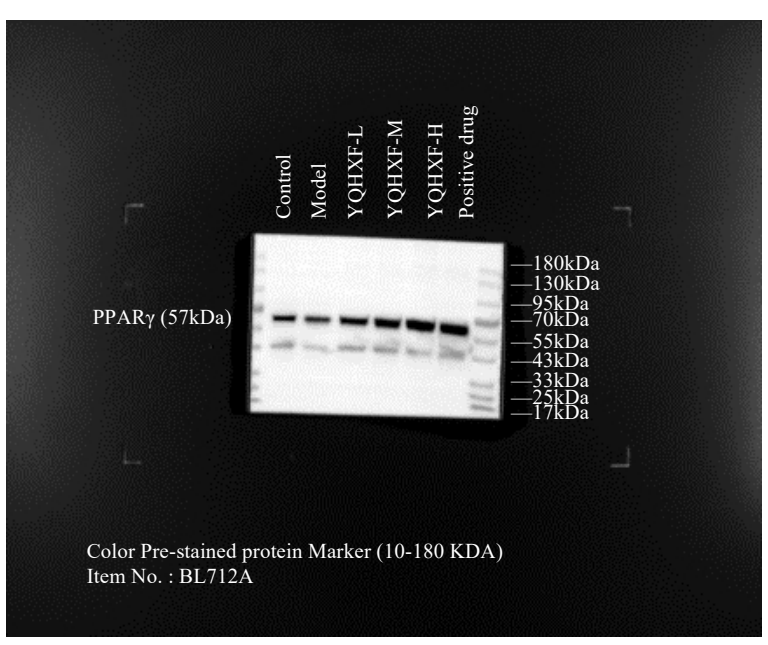

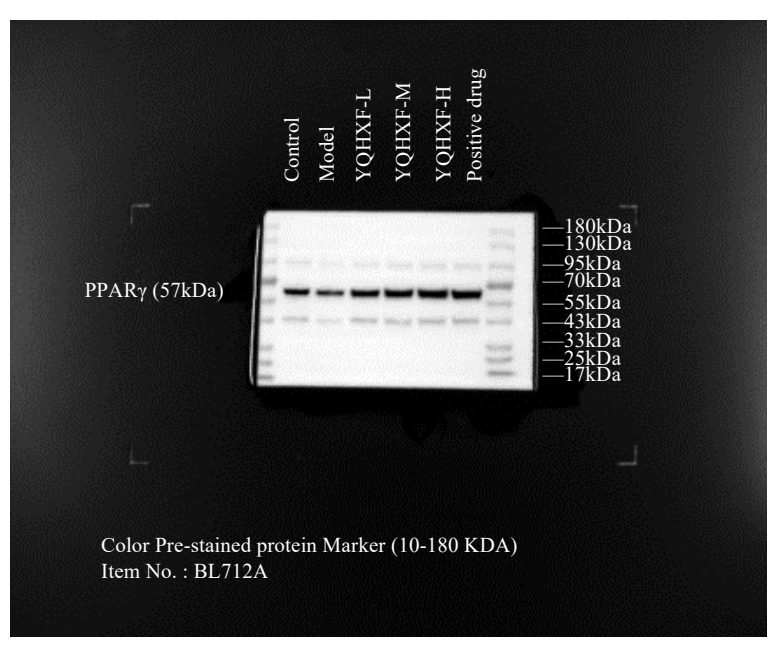

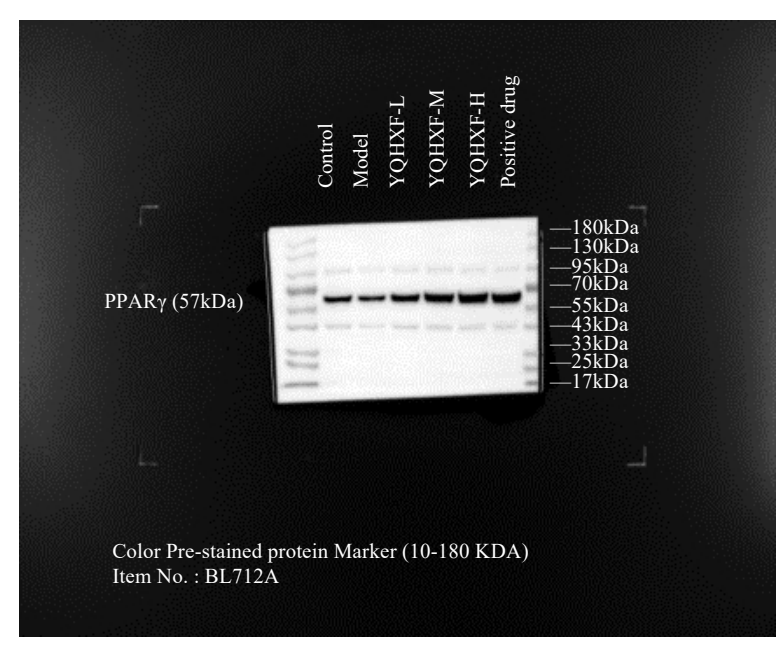

(4) β-actin

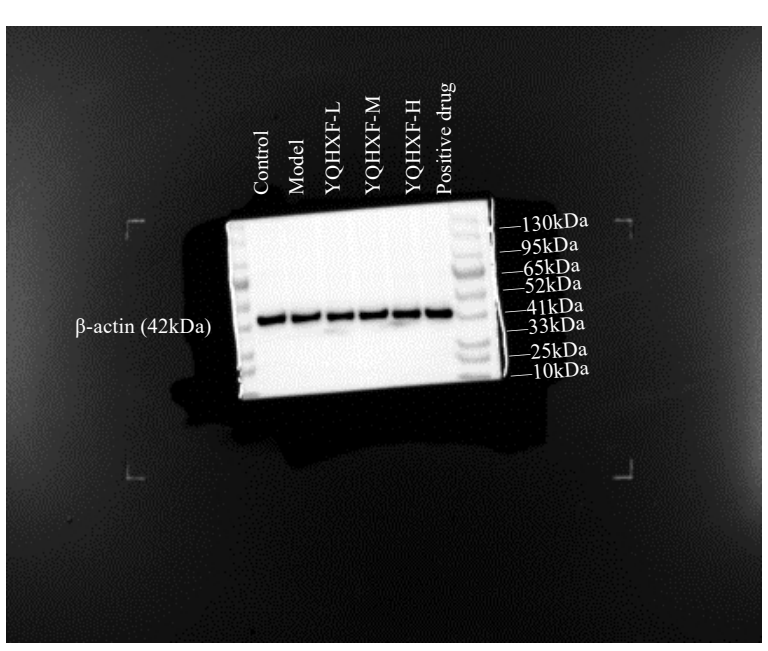

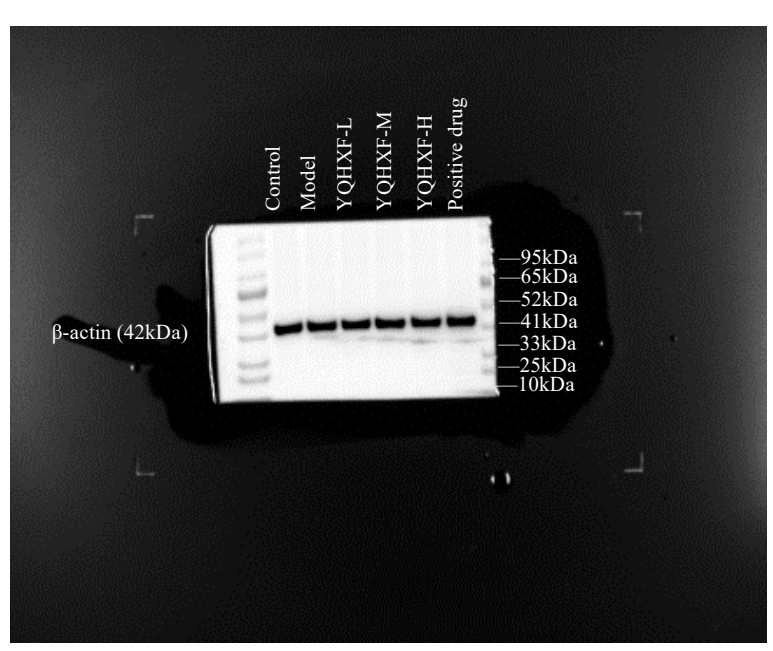

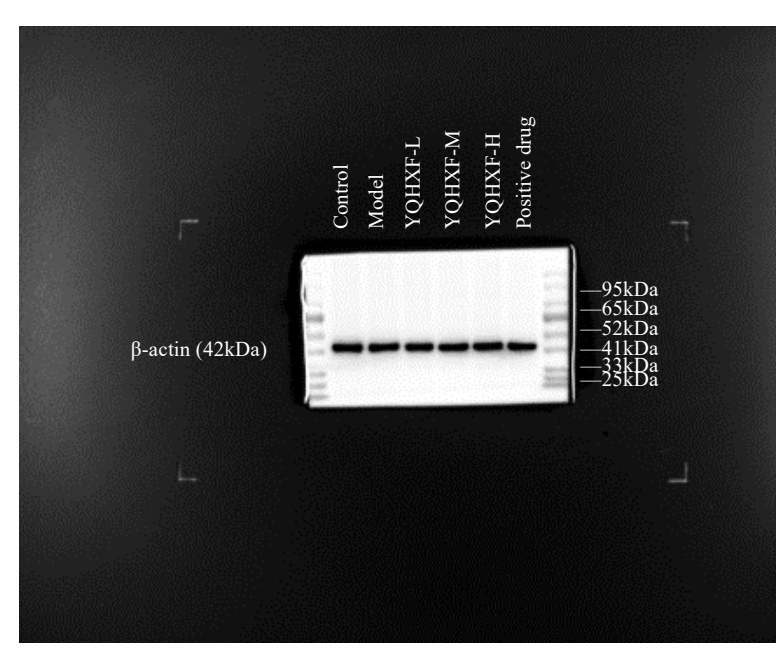

Figure 6F

(1) STAT6

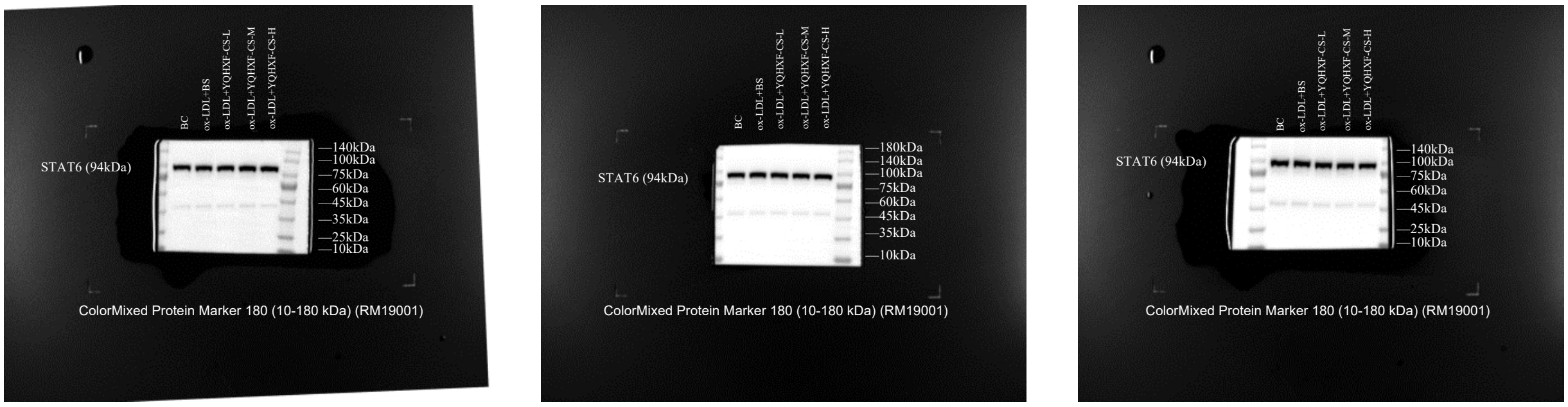

(2) p-STAT6

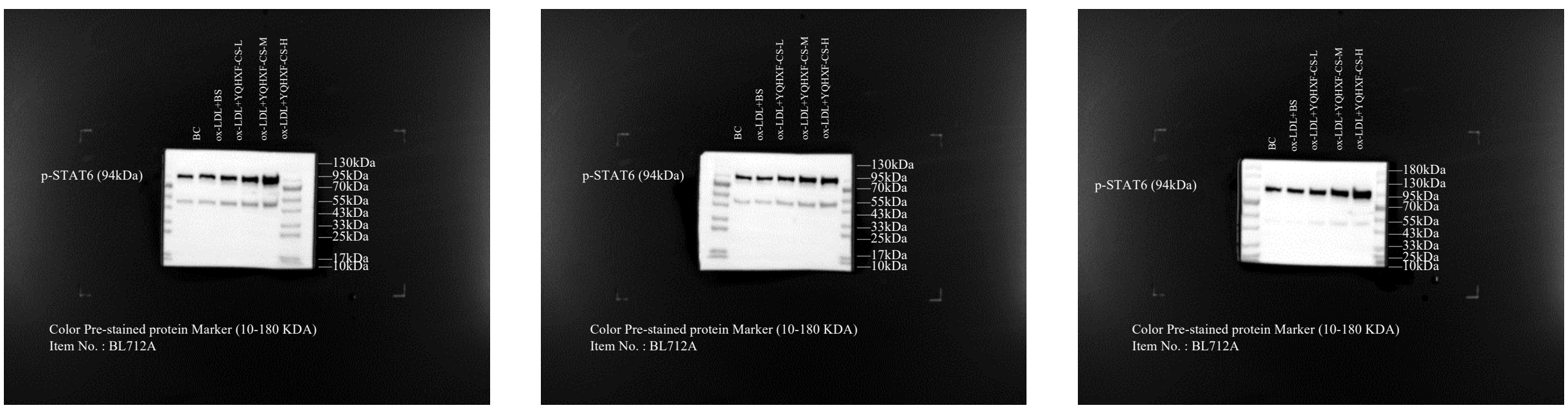

(3) PPARγ

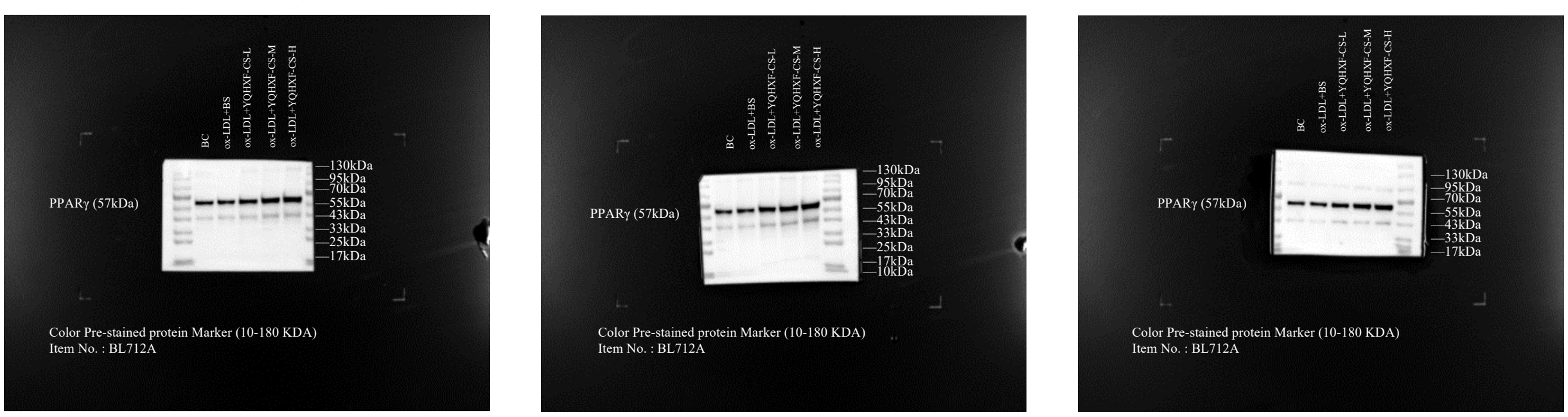

(4) β-actin

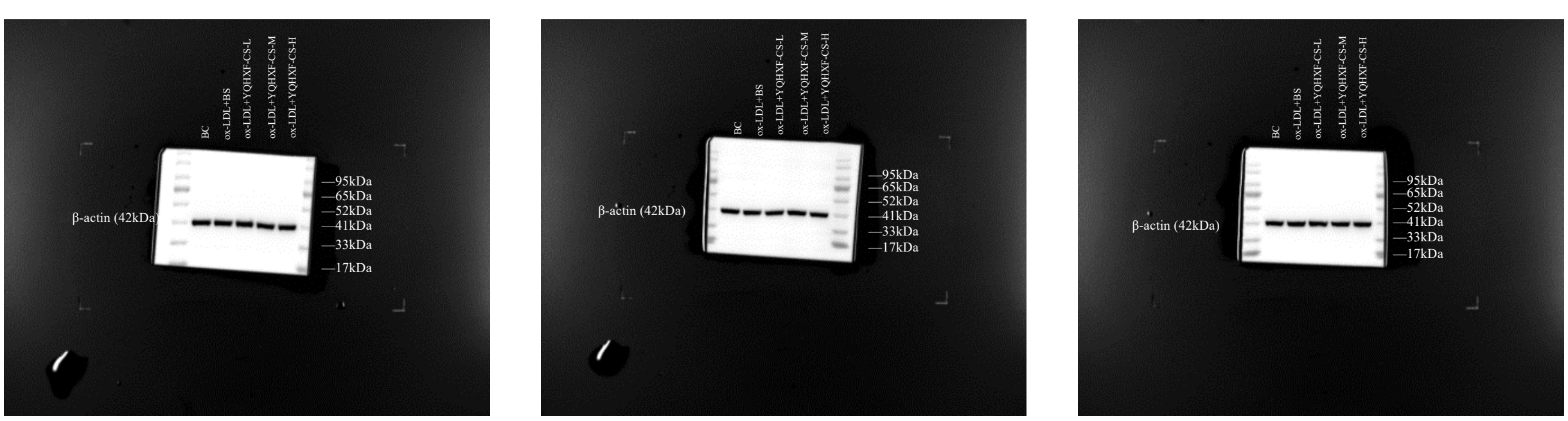

Figure 7B

(1) PPARγ

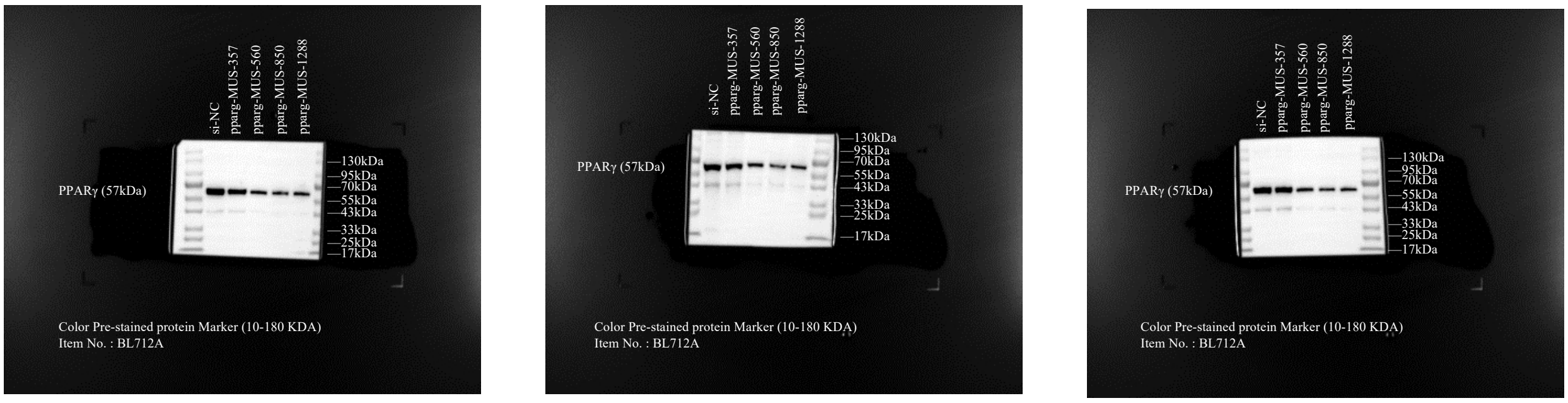

(2) β-actin

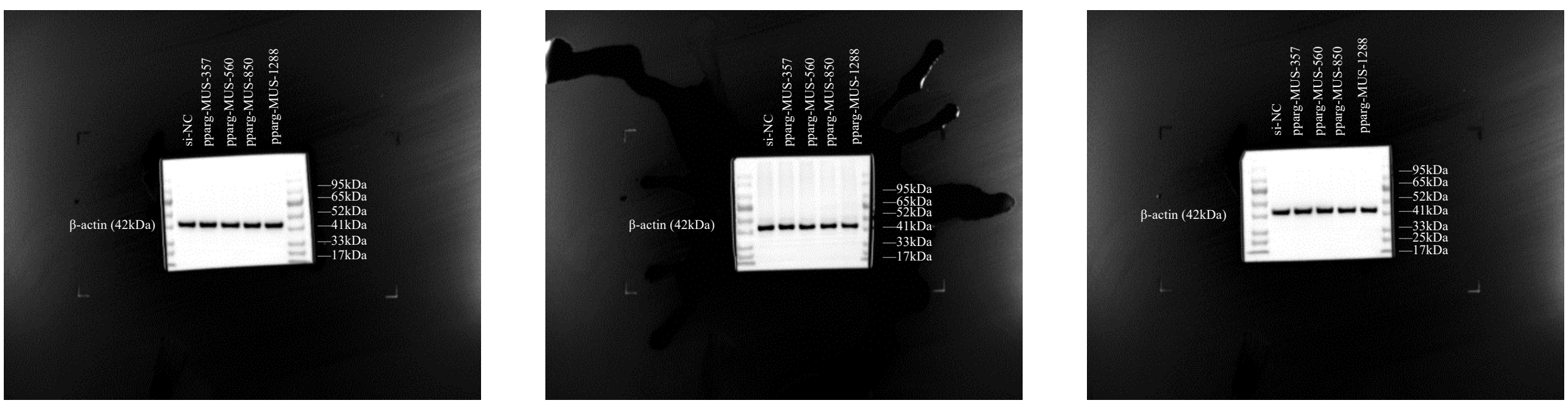

Figure 8F

(1) STAT6

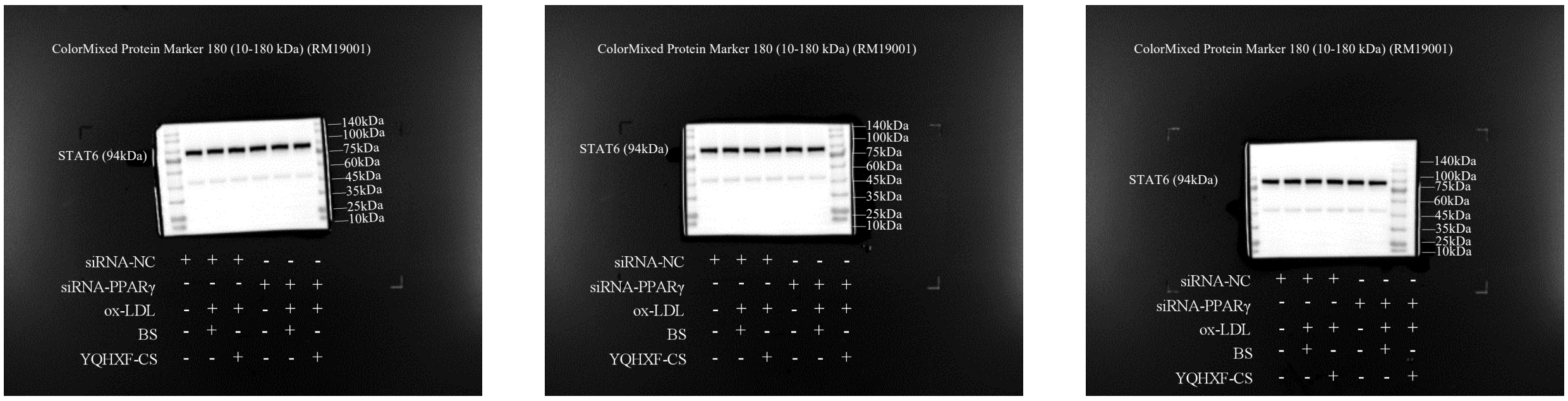

(2) p-STAT6

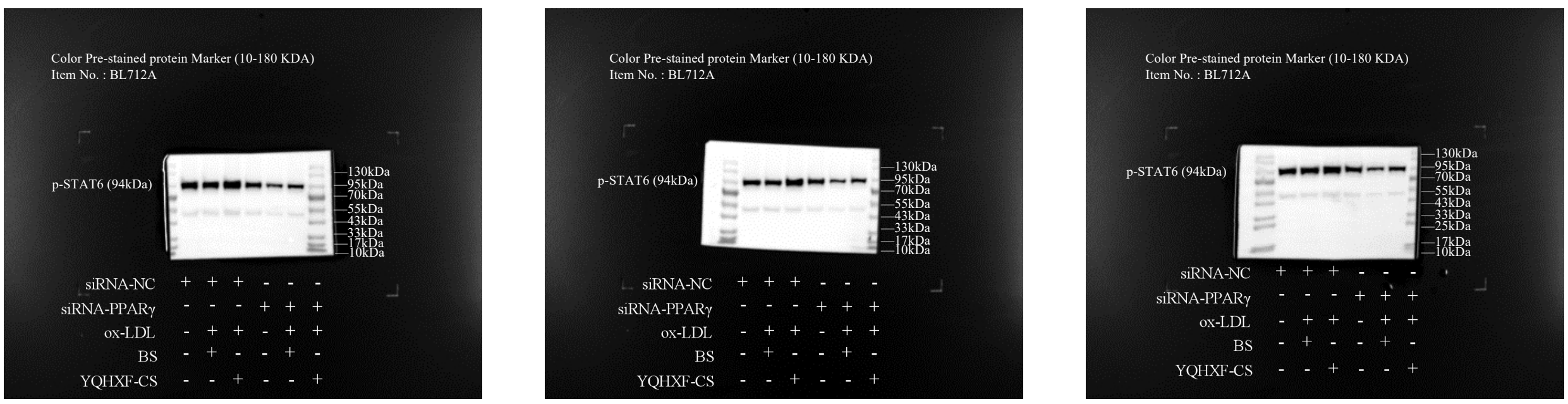

(3) PPARγ

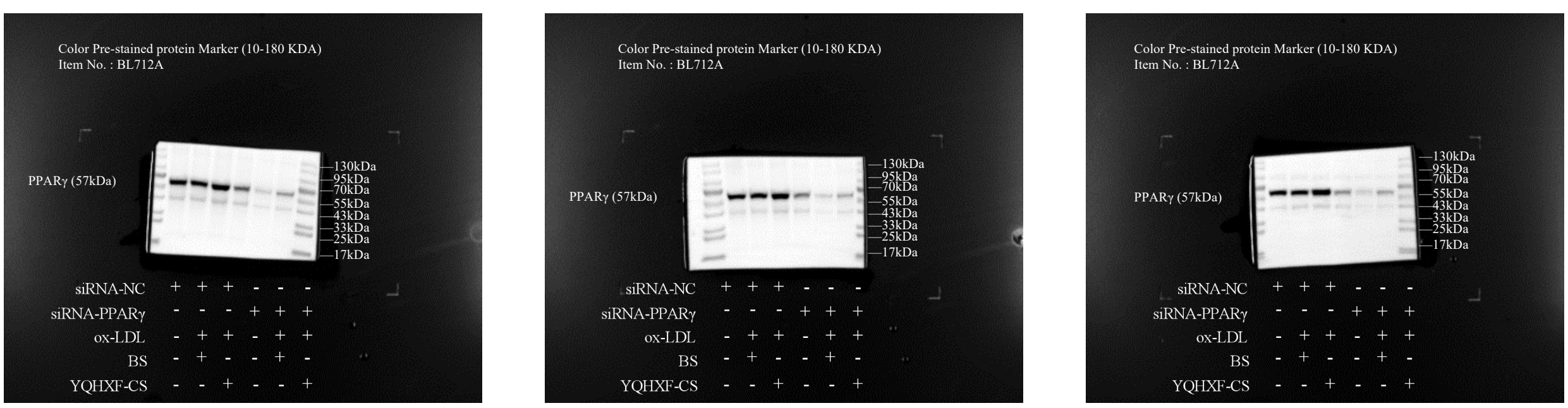

(4) β-actin

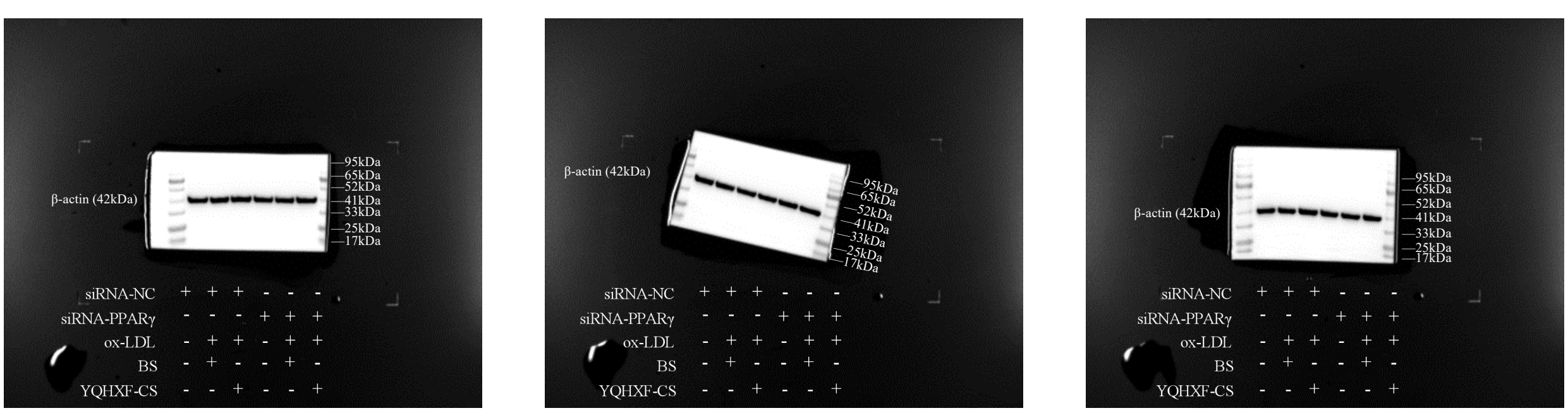

Supplement: Supplementary file 2 [file Datasheet2.pdf]
