# Supplementary material for: Yiqihuoxue formula improves atherosclerosis by regulating macrophage polarization associated with enhanced STAT6/PPARγ pathway
Source: Front Cardiovasc Med. 2026 Sep 22;13:1896890. doi: 10.3389/fcvm.2026.1896890 (PMC13638390; doi:10.3389/fcvm.2026.1896890)
Supplement: Supplementary file 3 [file Datasheet3.pdf]

Yiqihuoxue Formula improves atherosclerosis by regulating macrophage polarization through activating the STAT6/PPARγ pathway

Supplementary Material

Supplementary figures

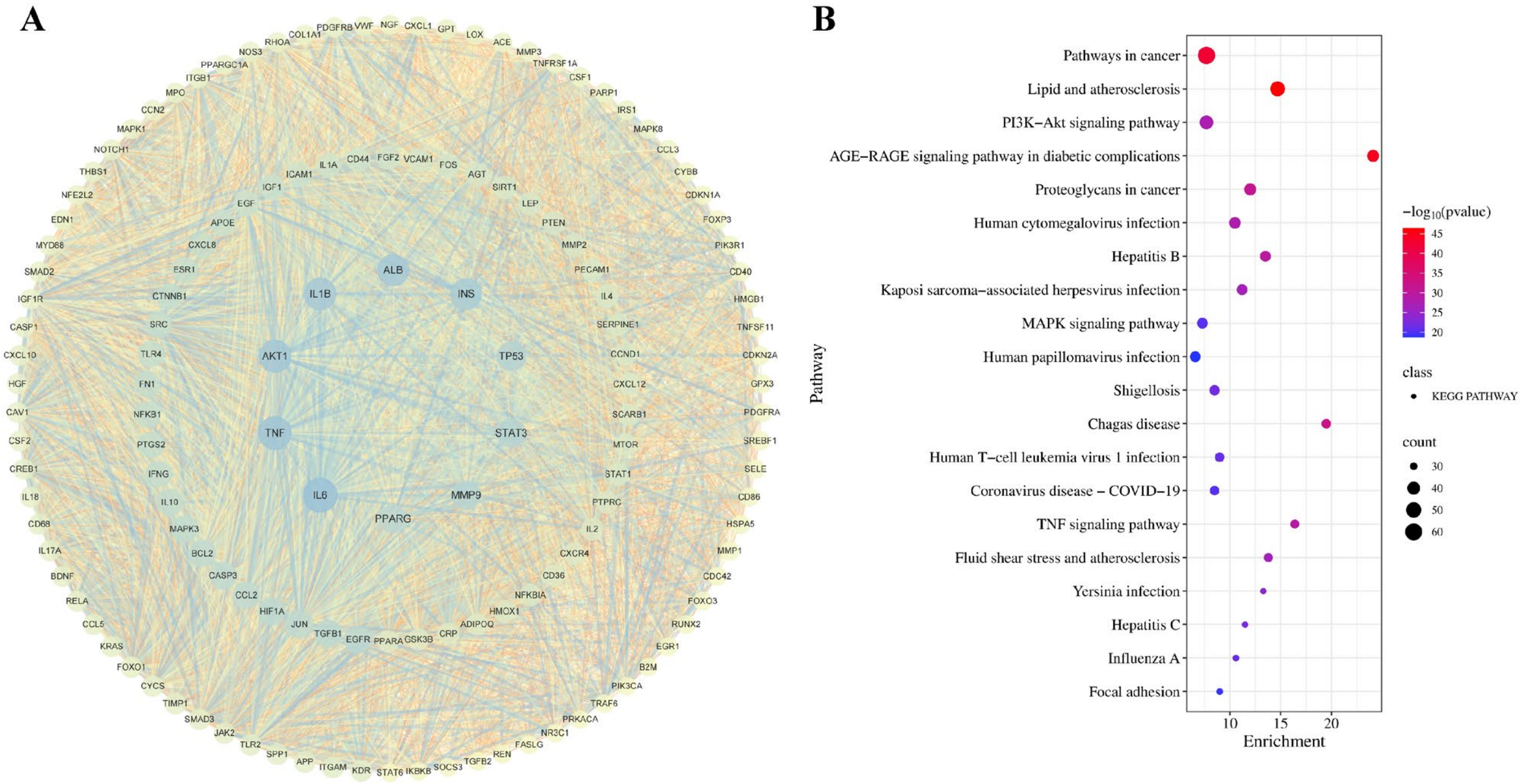

Fig. S1. A: The PPI network of the core targets of atherosclerosis. The size and color of the nodes represent the target degree values. The larger the node, the greater its degree value. B: Kyoto Encyclopedia of Genes and Genomes (KEGG) analysis of core targets of atherosclerosis.

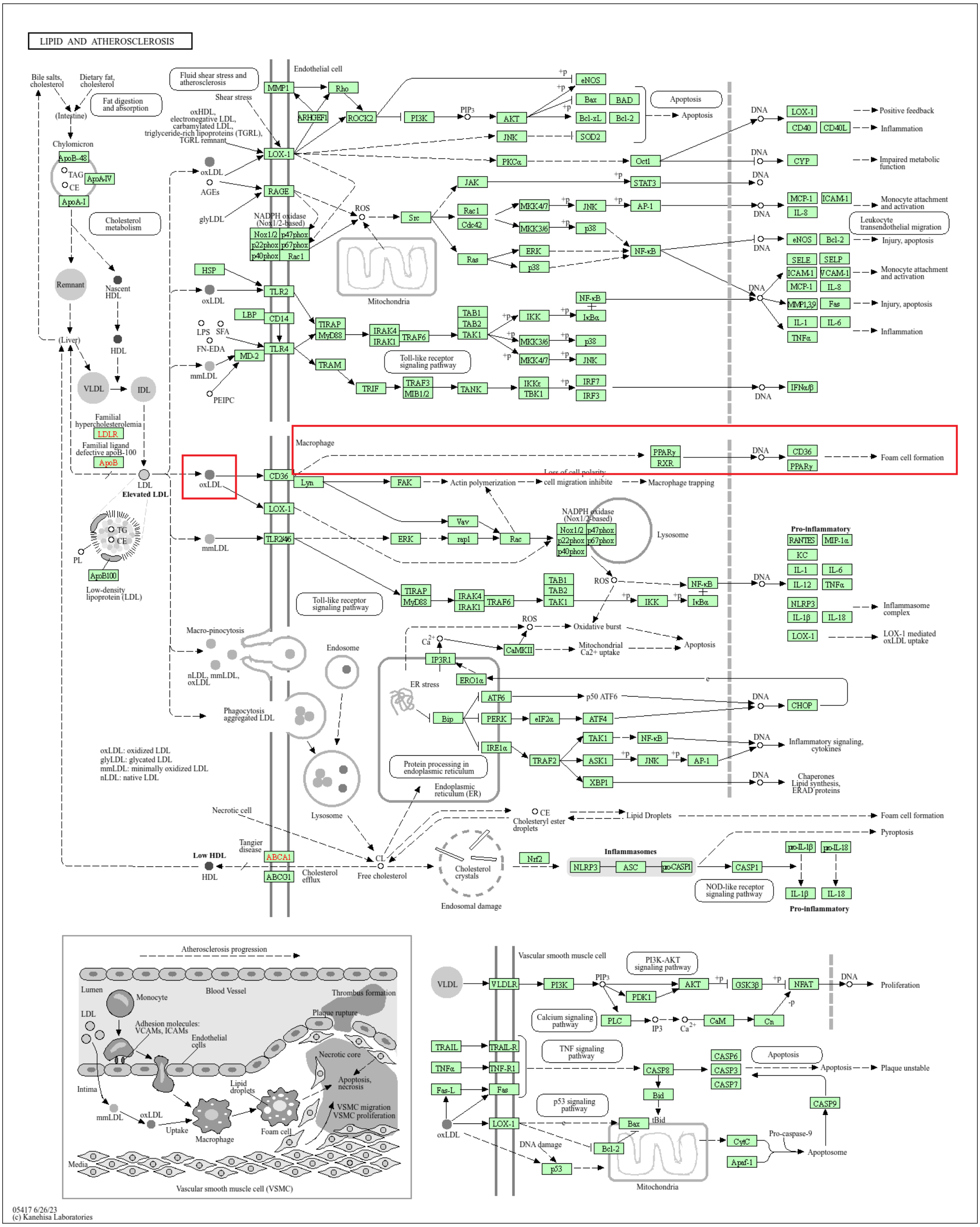

Fig. S2. Mechanistic diagram of the Lipid and atherosclerosis pathways. The red rectangle suggests that oxLDL, PPARγ, and CD36 are involved in foam cell formation in macrophages.

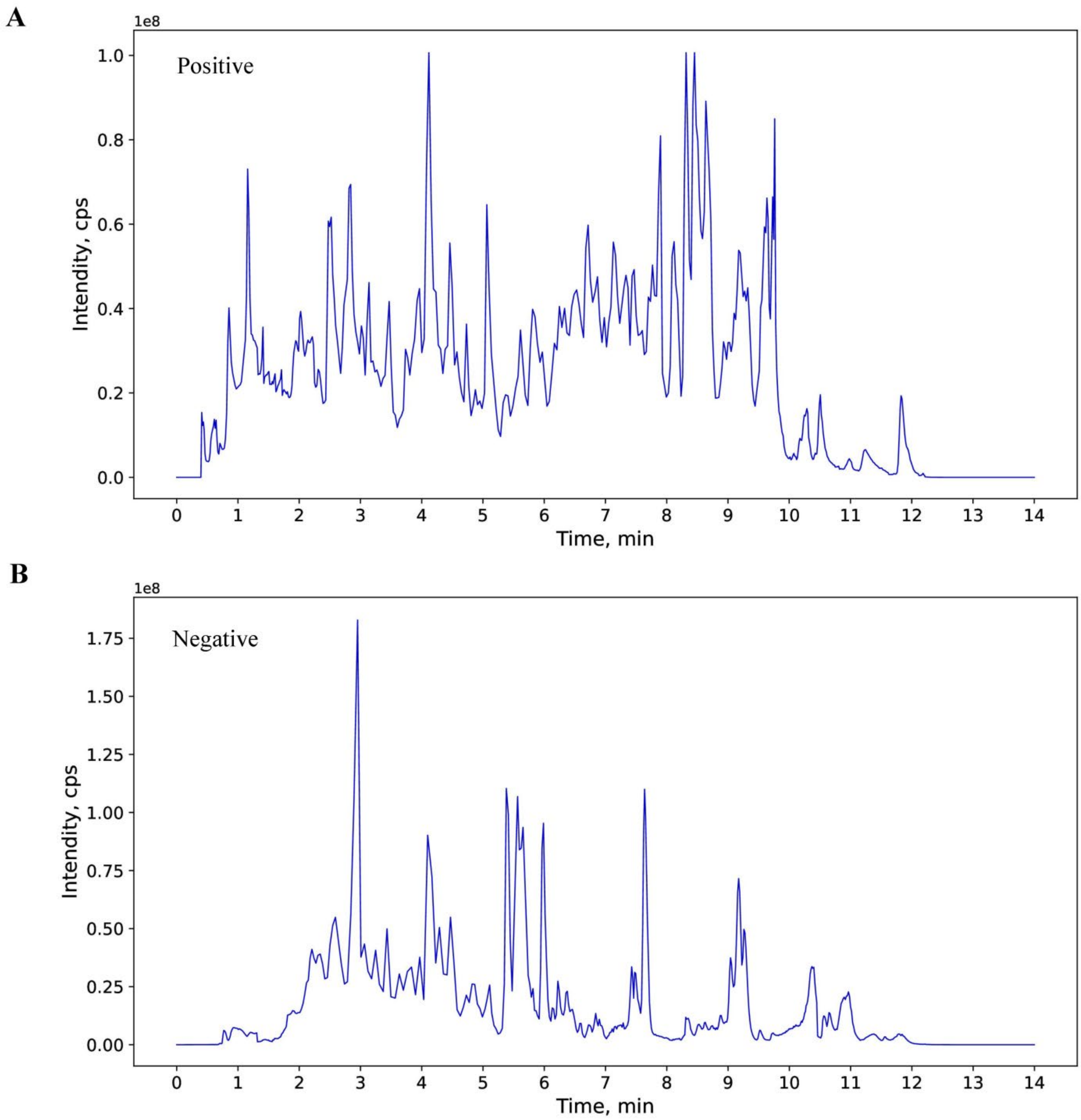

Fig. S3. Identification of the components of YQHXF-containing serum (YQHXF-CS). **A**: Total ion chromatogram (TIC) in positive ion mode; **B**: TIC in the negative ion mode.

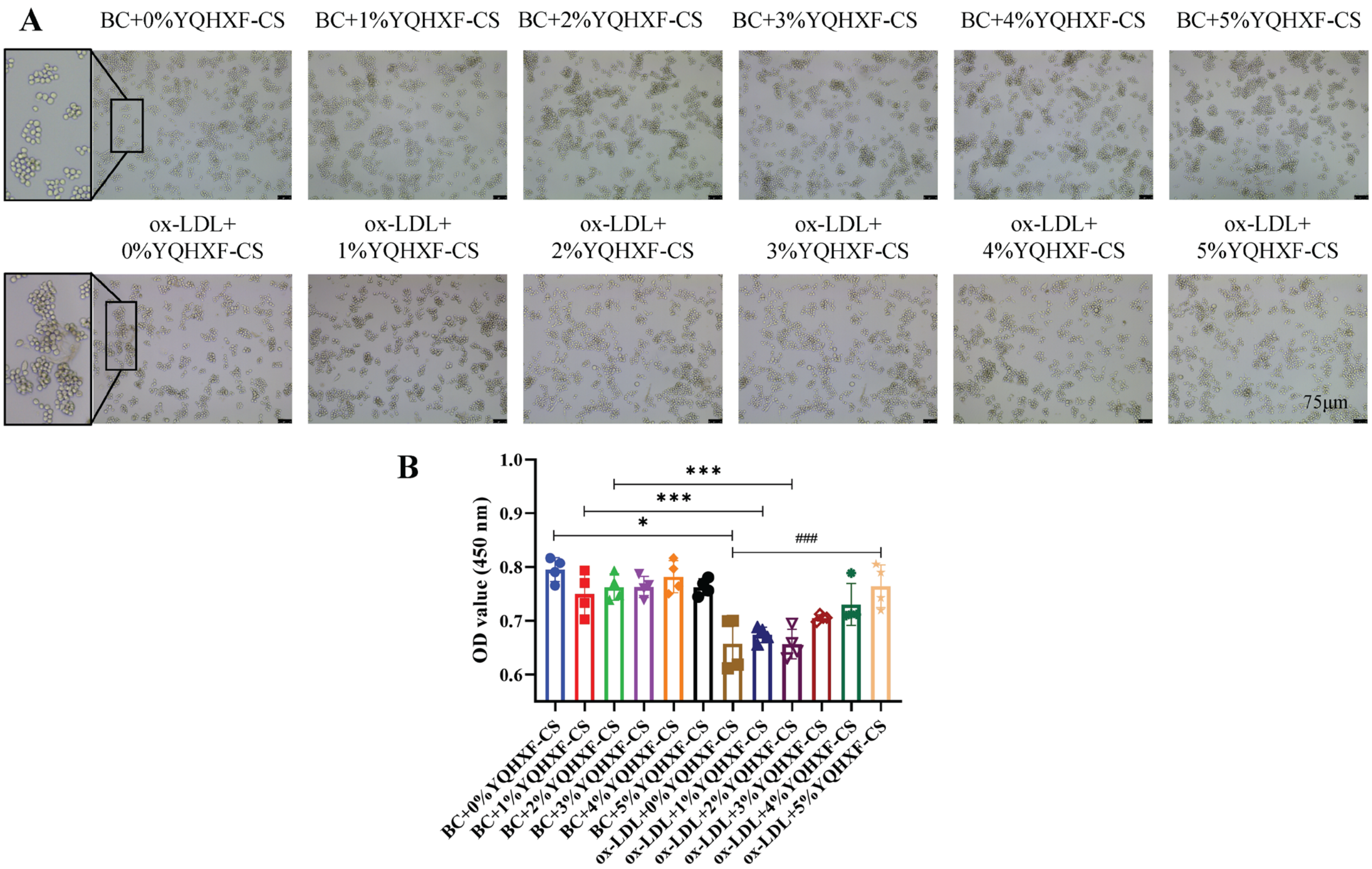

Fig. S4. RAW264.7 cells viability detected by CCK-8 assay. **A**: Morphology of RAW264.7 cells under microscope, scale bar = 75 μm; **B**: Absorbance (OD) was measured at a wavelength of 450 nm by microplate reader; higher OD values indicated stronger cell viability. $^{*}p < 0.05$, $^{***}p < 0.001$, ox-LDL-treated group *vs.* BC-treated group; $^{\#\#\#}p < 0.001$, compared with the ox-LDL+0%YQHXF-CS group. BC: Blank control; YQHXF-CS: Yiqihuoxue Formula containing serum; ox-LDL: Oxidized low-density lipoprotein.

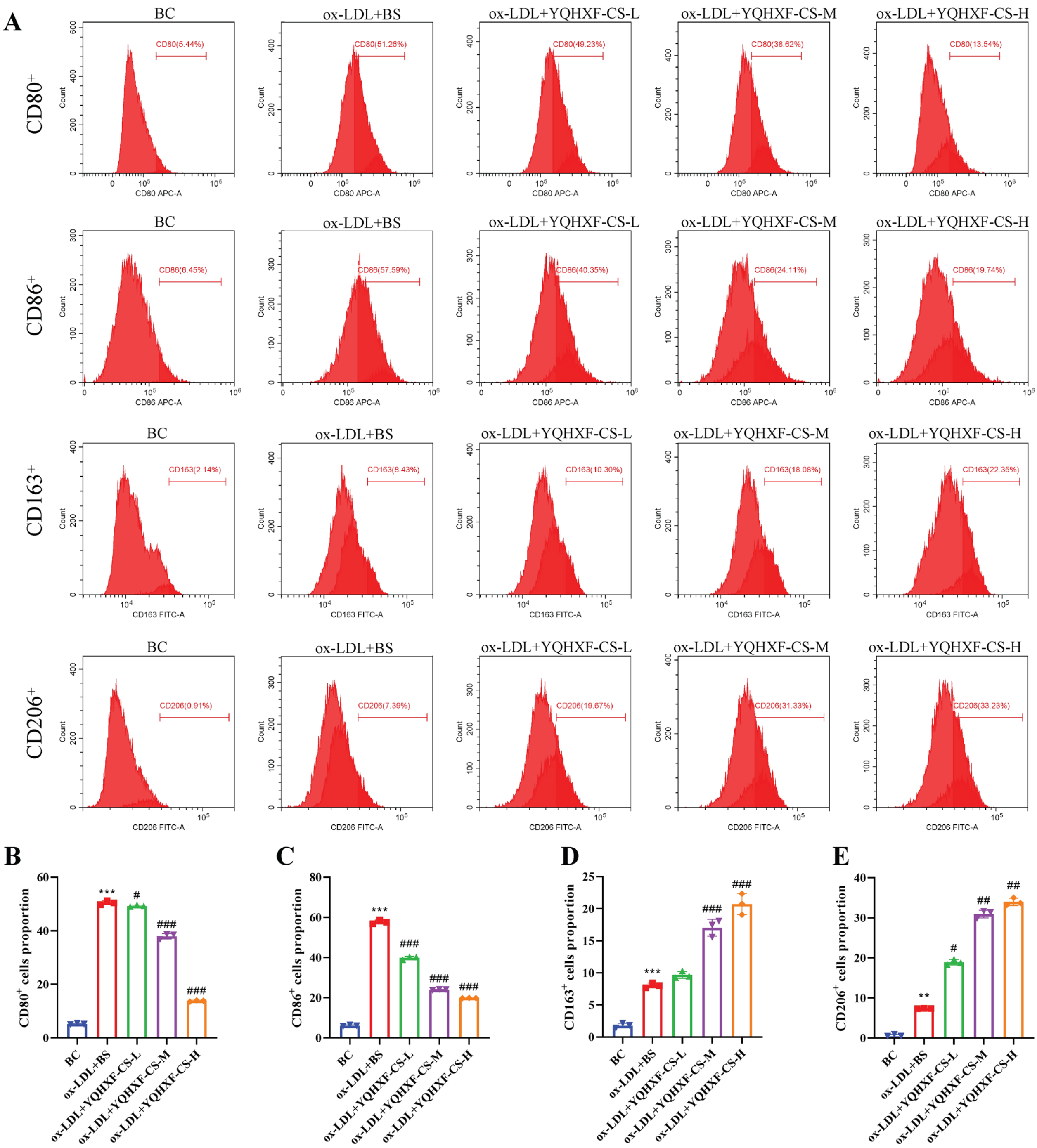

Fig. S5. The proportion of M1 macrophages (CD80$^+$, CD86$^+$) and M2 macrophages (CD163$^+$, CD206$^+$) was analyzed by flow cytometry. **A**: Representative flow cytometry analysis of macrophages. **B and C**: Flow cytometry analysis of M1 macrophages by CD80$^+$ marker and CD86$^+$ marker. **D and E**: Flow cytometry analysis of M2 macrophages by CD206$^+$ marker and CD163$^+$ marker. $^{**}p < 0.01$, $^{***}p < 0.001$ *vs.* BC group; $^{\#}p < 0.05$, $^{\#\#}p < 0.01$, $^{\#\#\#}p < 0.001$ *vs.* ox-LDL + BS group. BC: blank control; BS: blank serum; YQHXF-CS: Yiqihuoxue Formula containing serum; ox-LDL: Oxidized low-density lipoprotein.

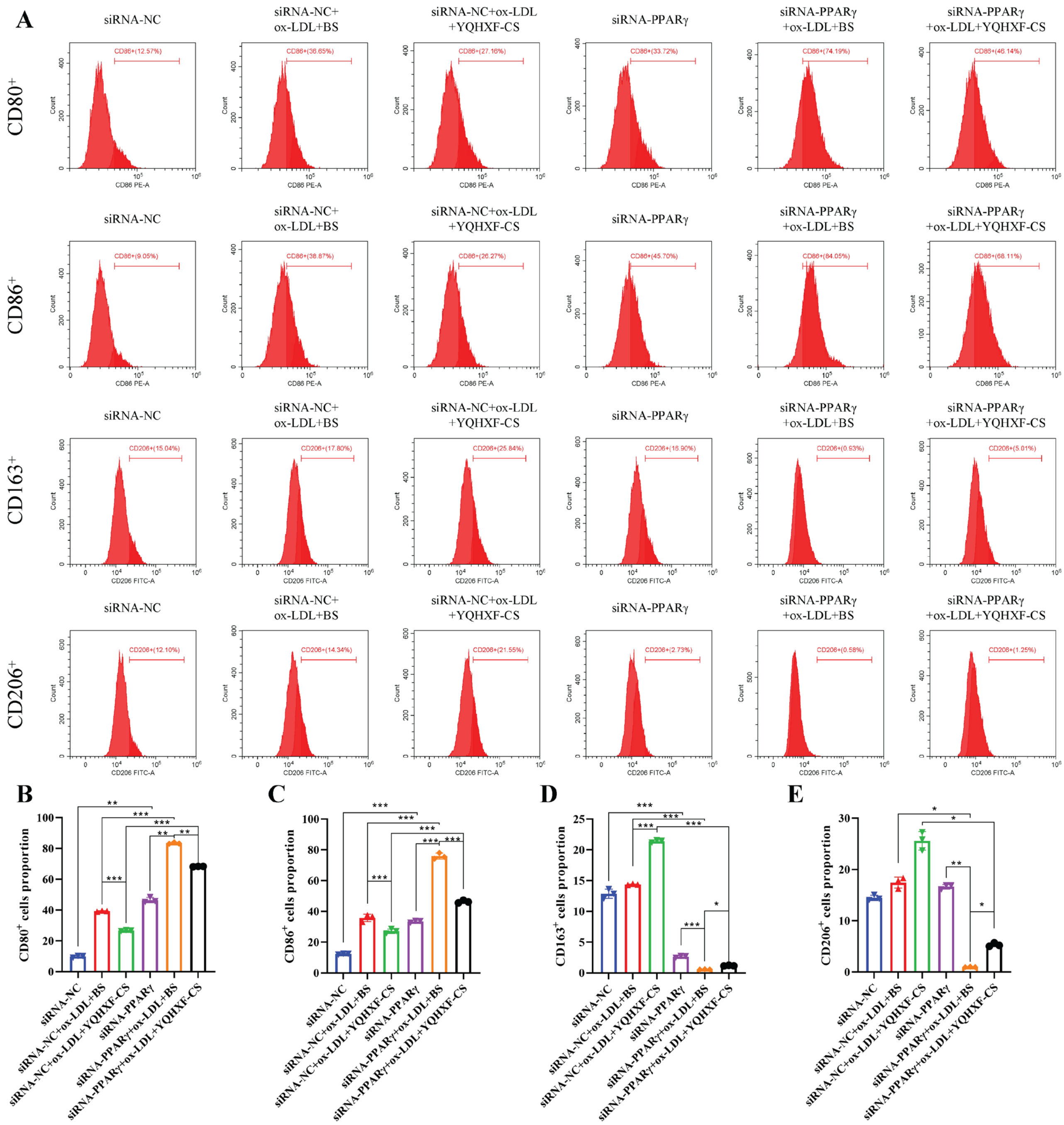

Fig. S6. The proportion of M1 macrophages ($CD80^+$, $CD86^+$) and M2 macrophages ($CD163^+$, $CD206^+$) was analyzed by flow cytometry. **A**: Representative flow cytometry analysis of macrophages. **B and C**: Flow cytometry analysis of M1 macrophages by $CD80^+$ marker and $CD86^+$ marker. **D and E**: Flow cytometry analysis of M2 macrophages by $CD206^+$ marker and $CD163^+$ marker. $^{*}p < 0.05$, $^{**}p < 0.01$, $^{***}p < 0.001$. BS: blank serum; YQHXF-CS: Yiqihuoxue Formula containing serum; ox-LDL: Oxidized low-density lipoprotein.

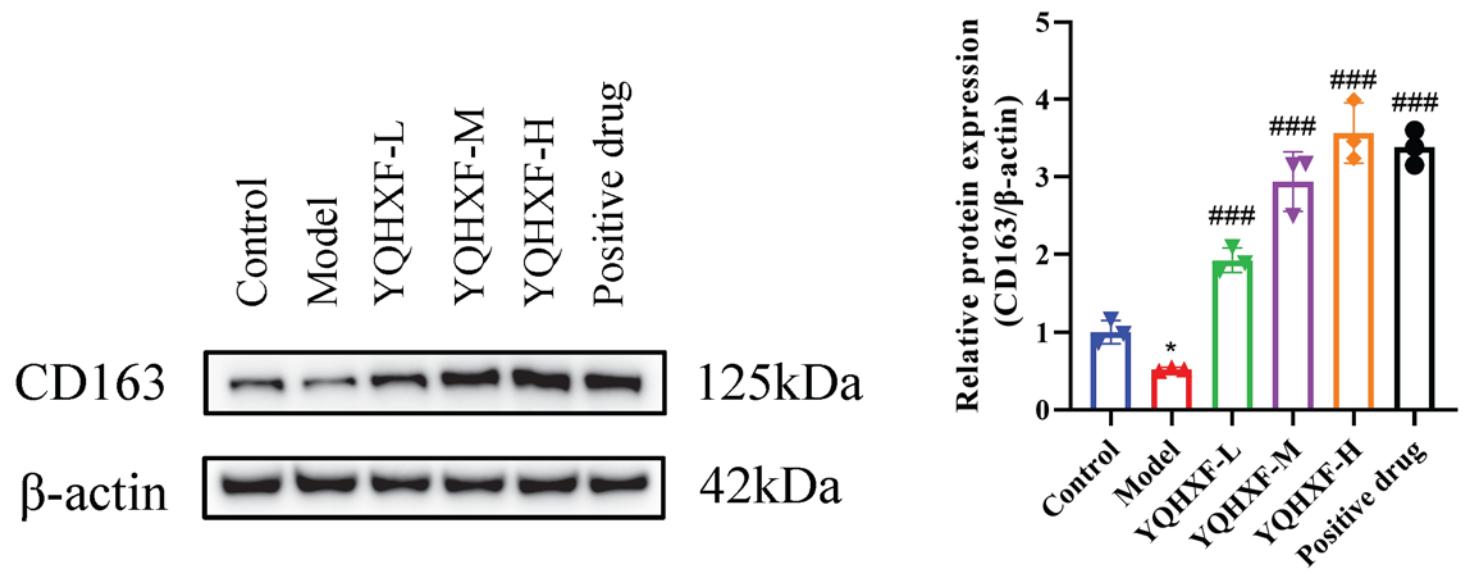

Fig. S7. Representative pictures and quantitative analysis of CD206 protein expression detected by Western blot; β-actin was used to normalize the data. Values are expressed as mean ± SD. $^{*}p < 0.05$, compared with the control group; $^{###}p < 0.001$, compared with the model group. YQHXF: Yiqihuoxue Formula.
